# Assessing risk of bias of cohort studies with large language models

**DOI:** 10.1017/rsm.2025.10028

**Published:** 2025-08-07

**Authors:** Danni Xia, Honghao Lai, Weilong Zhao, Jiajie Huang, Jiayi Liu, Ziying Ye, Jianing Liu, Mingyao Sun, Liangying Hou, Bei Pan, Long Ge

**Affiliations:** 1 Department of Health Policy and Management, School of Public Health, Lanzhou University, Lanzhou, China; 2 Evidence-Based Social Science Research Center, School of Public Health, Lanzhou University, Lanzhou, China; 3 College of Nursing, Gansu University of Chinese Medicine, Lanzhou, China; 4 School of Nursing, Peking University, Beijing, China; 5 Department of Health Research Methods, Evidence, and Impact, McMaster University, Hamilton, Ontario, Canada; 6 Evidence-Based Medicine Center, School of Basic Medical Sciences, Lanzhou University, Lanzhou, China; 7 Key Laboratory of Evidence Based Medicine of Gansu Province, Lanzhou, China

**Keywords:** cohort studies, large language models, risk of bias, systematic reviews

## Abstract

This study aims to explore the feasibility and accuracy of utilizing large language models (LLMs) to assess the risk of bias (ROB) in cohort studies. We conducted a pilot and feasibility study in 30 cohort studies randomly selected from reference lists of published Cochrane reviews. We developed a structured prompt to guide the ChatGPT-4o, Moonshot-v1-128k, and DeepSeek-V3 to assess the ROB of each cohort twice. We used the ROB results assessed by three evidence-based medicine experts as the gold standard, and then we evaluated the accuracy of LLMs by calculating the correct assessment rate, sensitivity, specificity, and *F*1 scores for overall and item-specific levels. The consistency of the overall and item-specific assessment results was evaluated using Cohen’s kappa (κ) and prevalence-adjusted bias-adjusted kappa. Efficiency was estimated by the mean assessment time required. This study assessed three LLMs (ChatGPT-4o, Moonshot-v1-128k, and DeepSeek-V3) and revealed distinct performance across eight assessment items. Overall accuracy was comparable (80.8%–83.3%). Moonshot-v1-128k showed superior sensitivity in population selection (0.92 versus ChatGPT-4o’s 0.55, *P* < 0.001). In terms of *F*1 scores, Moonshot-v1-128k led in population selection (*F* = 0.80 versus ChatGPT-4o’s 0.67, *P* = 0.004). ChatGPT-4o demonstrated the highest consistency (mean κ = 96.5%), with perfect agreement (100%) in outcome confidence. ChatGPT-4o was 97.3% faster per article (32.8 seconds versus 20 minutes manually) and outperformed Moonshot-v1-128k and DeepSeek-V3 by 47–50% in processing speed. The efficient and accurate assessment of ROB in cohort studies by ChatGPT-4o, Moonshot-v1-128k, and DeepSeek-V3 highlights the potential of LLMs to enhance the systematic review process.

## Highlights

### What is already known?

- Systematic reviews synthesize and evaluate existing research, guiding clinical decision-making and providing information for health guidelines. The assessment of risk of bias (ROB) is a critical step in this process.
- Currently, large language models (LLMs) have demonstrated exceptional capabilities in understanding and generating human-like text. With the support of advanced machine learning algorithms and vast datasets, these models have the potential to revolutionize the creation of systematic reviews.

### What is new?

- The study provides novel evidence that LLMs can accurately and efficiently assess the ROB in cohort studies, demonstrating their potential to improve the systematic review process.
- ChatGPT-4o showed superior efficiency and comparable accuracy in assessing ROB compared to Moonshot-v1-128k, highlighting the role of LLMs in reducing the time burden for systematic review tasks.
- The use of LLMs for ROB assessment in cohort studies offers a promising alternative to traditional methods, potentially streamlining systematic reviews in the future.

### Potential impact for RSM readers

- ROB assessment is not only critical but also highly time-consuming to assess. This approach can effectively handle and analyze large amounts of data, potentially improving the efficiency of systematic reviews of nonrandomized studies.

## 1 Introduction

Systematic reviews are integral to evidence-based decision-making in public health and clinical practice. They systematically synthesize existing research evidence, elucidate the strengths and limitations of studies, and offer a scientific foundation for the development of clinical guidelines and health policies.^1^ ^,^ ^2^ However, the process is resource-intensive, requiring substantial time and specialized training.^3^ ^–^ ^5^ Additionally, the heterogeneity in the quality of the available literature, such as unclear descriptions or missing information in some studies, presents significant challenges to ensuring consistent quality assessments when conducted independently by two reviewers.^6^ ^–^ ^8^

Risk-of-bias (ROB) assessment is a pivotal step in the development of systematic reviews, as it helps determine the credibility and reliability of the findings.^9^ Recent advancements in machine learning and large language models (LLMs) have transformed the ROB assessment process, mitigating the labor-intensive demands of systematic reviews and meta-analyses.^10^ ^,^ ^11^ LLMs exhibit exceptional capabilities in processing and analyzing extensive biomedical data,^12^ providing an innovative perspective for automating and enhancing the accuracy and efficiency of ROB assessments.^13^

In 2023, a pioneering study explored the use of ChatGPT and Claude for assessing ROB in randomized controlled trials (RCTs),^14^ reporting high levels of accuracy and consistency. However, the application of artificial intelligence (AI) in systematic reviews remains predominantly focused on RCTs, with less attention given to non-RCTs.

This study aimed to assess the feasibility and performance of LLMs in assisting with ROB assessment in cohort studies.

## 2 Methods

**Figure 1:**
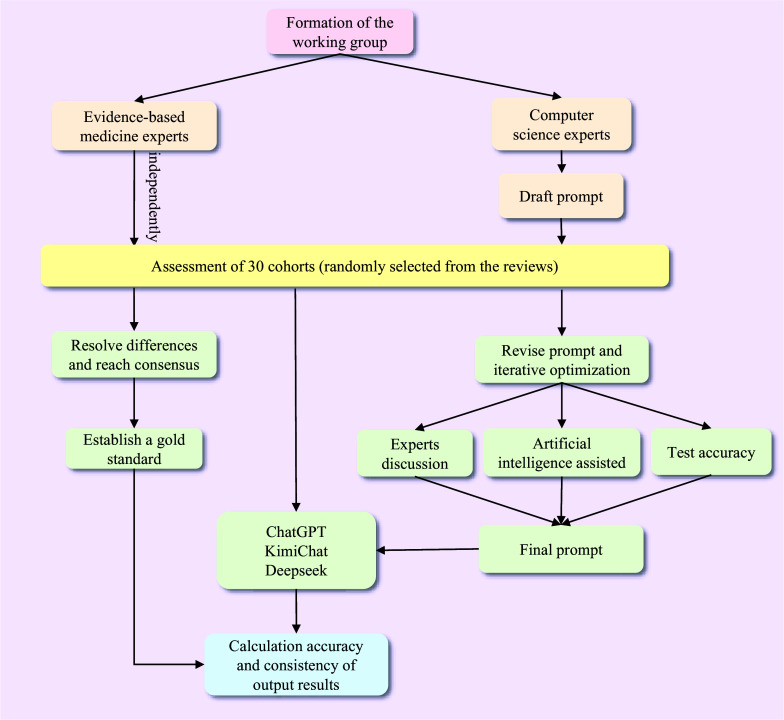
Flow diagram of the main study process.

The research team comprised three senior experts in evidence-based medicine methodology (B.P., L.H., and L.G.) and two computer science specialists (J.H. and W.Z.), adhering to the TRIPOD-LLM guideline.^15^ The Medical Ethics Review Committee of Lanzhou University’s School of Public Health exempted the study from requiring approval, as all data were derived from published studies. Figure 1 illustrates the entire process.

### 2.1 Sample selection

We searched the Cochrane Library database using the keywords “Cohort Analysis,” “Historical Cohort Studies,” and “Cohort Studies.” We selected 57 reviews published between January 1, 2020, and October 8, 2023 (Appendix 1 of the Supplementary Material), with no language restrictions. We excluded withdrawn publications, unavailable full texts, duplicates, and noncohort studies. To facilitate study management and ensure unbiased selection, we assigned a unique identification code to each cohort study included in the reviews and then randomly selected 30 studies using computer-generated sampling.

### 2.2 Prompt development

We used a modified version of the Newcastle-Ottawa Scale (NOS) to assess the ROB of cohort studies,^16^ which included “Population Selection,” “Exposure Confidence,” “Outcome Baseline,” “Variable Adjustment,” “Prognostic Assessment,” “Outcome Confidence,” “Follow-up Adequacy,” “Intervention Similarity,” and we answered each item as to be definitely/probably yes (low ROB) and definitely/probably no (high ROB), based on a detailed assessment criterion (Appendix 2 of the Supplementary Material). We drafted structured prompts including the assessment criterion, output format of the assessment results, and examples of the final output. We iteratively optimized the prompts through expert discussion and AI assistance until the instructions could be efficiently and correctly evaluated. Box 1 contains parts of the prompts. The complete prompts is in Supplementary Material (Appendix 3 of the Supplementary Material).

**Figure:**
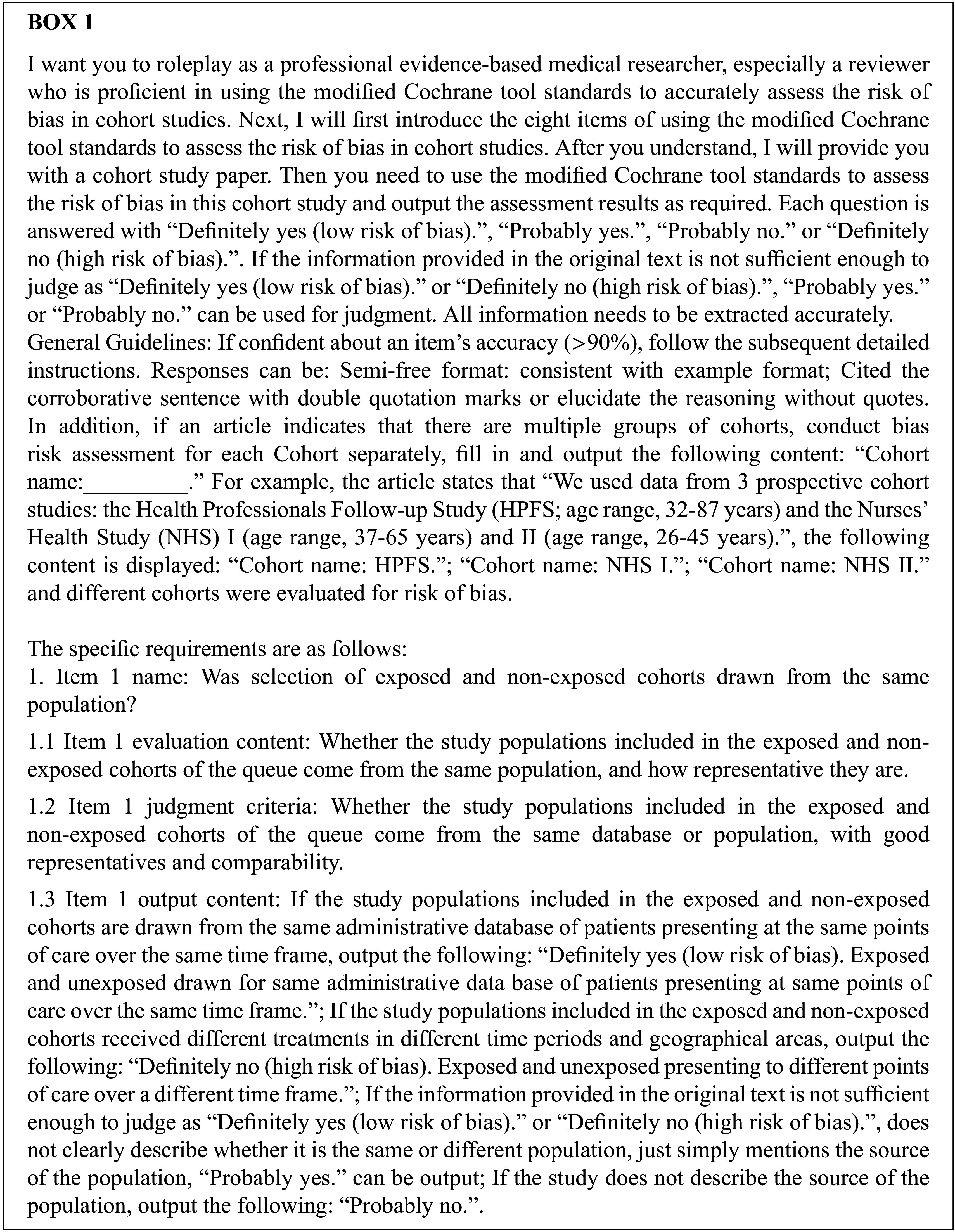

### 2.3 LLM assessment implementation

We employed three LLMs in this study: ChatGPT-4o (OpenAI), Moonshot-v1-128k (Moonshot AI), and DeepSeek-V3 (DeepSeek AI). In the figures, these models are referred to as “ChatGPT,” “Kimichat,” and “DeepSeek,” respectively, to indicate the specific applications or services used. All three models support long-context processing (up to 128k tokens) and allow for direct uploading of PDF files. The temperature parameter was set to 0.2 across all models to ensure stable and consistent outputs during ROB assessments. For each item, the model was required to select one of four assessment options based on the information provided in the research article: “definitely yes,” “probably yes,” “probably no,” and “definitely no.” In cases where insufficient information was available to make a definitive judgment, the model could select either “probably yes” or “probably no” in accordance with the instructions. For studies with multiple cohorts, ROB assessments were conducted for each individual cohort group. The results of these assessments were then outputted in a standardized format. To assess outputs’ consistency, we repeated the assessment process twice for the same cohort study using a new chat window. Each output result was recorded accurately; in addition, we recorded the time of each operation from the submission of the material to the completion of the result.

### 2.4 Gold standard establishment

Three methodologists (B.P., L.H., and L.G.) collaboratively read 30 cohort studies and assessed the ROB using the modified tool. After the preliminary assessment, the reviewers compared the assessment results from LLMs to those from the Cochrane review to identify their consistency. For studies with inconsistent results, reviewers discussed the reasons and re-examined original texts to enhance objectivity and consistency. Ultimately, they reached a consensus to establish a gold standard for each cohort.

### 2.5 Data analysis

We conducted data analysis using R version 4.3.2.^17^ We categorized responses of “definitely yes” or “probably yes” as “low risk” (negative outcome), and those of “definitely no” or “probably no” as “high risk” (positive outcome). To compare the overall correctness rates among the LLMs, we calculated the rate difference (RD) with 95% confidence intervals (CIs). A *P* value of <0.05 was considered statistically significant.

#### 2.5.1 Accuracy

The performance of ChatGPT-4o, Moonshot-v1-128k, and DeepSeek-V3 in the RoB assessment was evaluated at both overall and item-specific levels. Standard classification metrics were used to quantify accuracy, including correct assessment rate, sensitivity, and specificity. To compare correct assessment rates between the two models, we calculated RDs with corresponding 95% CIs. True positives (TPs) and true negatives (TNs) were determined based on the gold standard, while false positives (FPs) and false negatives (FNs) represented deviations from this standard. For item-specific evaluation, we used the F1 score, which is the harmonic mean of sensitivity and precision (positive predictive value).

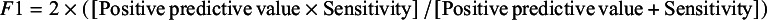



#### 2.5.2 Consistency

To evaluate the consistency of LLMs’ assessments, we focused on the stability of their outputs when the same PDF was submitted to the same LLM consecutively, we used Cohen’s kappa (κ) statistic.^18^ The observed agreement (Po) and expected agreement (Pe) were used to compute the kappa value. Additionally, the prevalence-adjusted and bias-adjusted kappa (PABAK) was calculated to mitigate the influence of prevalence and evaluator bias, ensuring that consistency assessments were unaffected by human factors.

#### 2.5.3 Efficiency

Assessment efficiency is measured by the total time from text upload to item assessment completion. The study ensures a global network bandwidth of 100 Mbps for upload and processing.

## 3 Results

### 3.1 Prompt for LLMs

We developed a prompt (Appendix 3 of the Supplementary Material) that required ChatGPT-4o, Moonshot-v1-128k, and DeepSeek-V3 to understand and apply the eight items of the modified tool to assess the ROB in cohort studies. The output results are shown in Appendix 4 of the Supplementary Material.

### 3.2 Characteristics of the cohort studies

We randomly selected 30 cohort studies that spanned multiple research fields,^19^ ^–^ ^48^ including COVID-19 (*n* = 4), primary breast cancer (*n* = 4), acute appendicitis (*n* = 3), and diabetic retinopathy (*n* = 3). Others included mild cognitive impairment or dementia, diarrhea, preschool autism spectrum disorder, prognosis after epileptic seizures, the relationship between smoking cessation and cardiovascular or mental health, tuberculosis, and the use of health services in low- and middle-income countries. The majority of the publications were from 2015 and beyond (*n* = 18).

### 3.3 Accuracy

**Figure 2:**
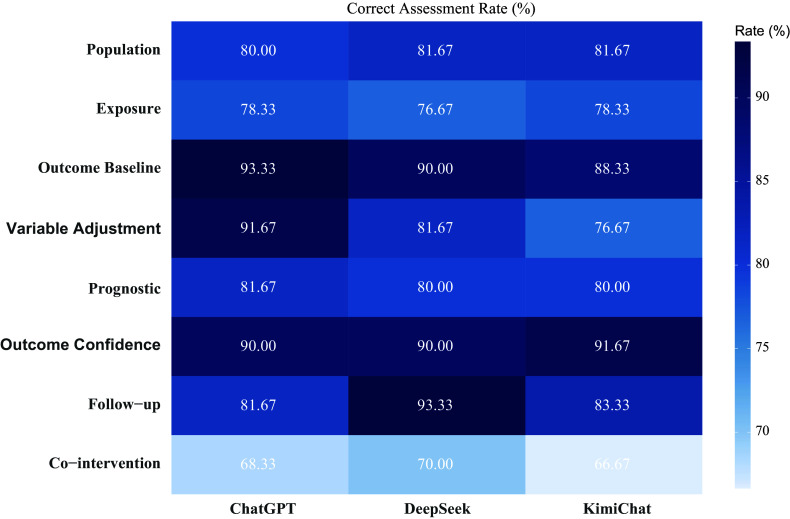
Heatmap of accuracy assessment rates.

The complete assessment results are presented in Supplementary Tables S1 and S2. As shown in Figure 2 and Table 1, among the eight assessment items, the three LLMs (ChatGPT-4o, Moonshot-v1-128k, and DeepSeek-V3) exhibited comparable overall accuracy. The average correct assessment rates are 83.13% (95% CI: 79.79%–86.47%) for ChatGPT-4o, 83.04% (95% CI: 79.68%–86.40%) for DeepSeek-V3, and 80.83% (95% CI: 77.31%–84.35%) for Moonshot-v1-128k. The differences in overall correct assessment rates among the three models did not reach statistical significance (ChatGPT-4o versus Moonshot-v1-128k: RD = 2.3%, 95% CI: −0.38% to 4.98%, *P* = 0.64; ChatGPT-4o versus DeepSeek-V3: RD = 0.09%, 95% CI: −0.83% to 1.01%, *P* = 0.98). Performance heterogeneity was observed across items, with peak accuracy in outcome baseline (ChatGPT-4o: 93.33%) and follow-up adequacy (DeepSeek-V3: 93.33%), while all models underperformed in co-intervention similarity (ChatGPT-4o: 68.33%; Moonshot-v1-128k: 66.67%; DeepSeek-V3: 70.00%). ChatGPT-4o demonstrated superior performance in variable adjustment with a correct assessment rate of 91.67%, significantly outperforming both Moonshot-v1-128k (76.67%; RD = 15%; 95% CI: 5%–25%; *P* = 0.02) and DeepSeek-V3 (81.67%; RD = 10%; 95% CI: 1%–19%; *P* = 0.03). The difference between DeepSeek-V3 and Moonshot-v1-128k did not reach statistical significance (RD = 5%; 95% CI: −4% to 14%; *P* = 0.28).

**Figure 3:**
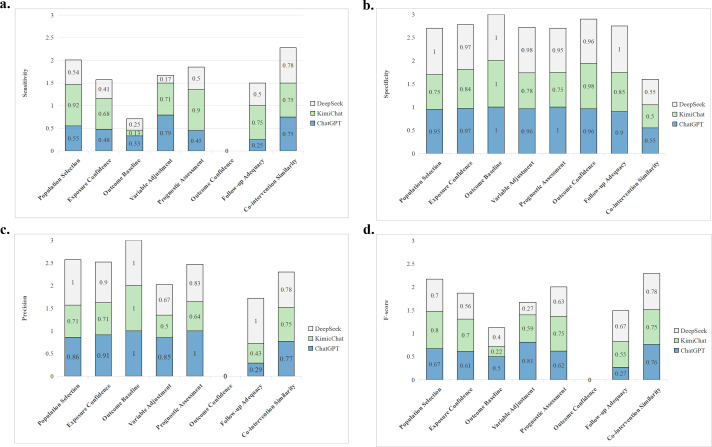
Performance comparison of the assessment.

The models exhibited substantial variation in sensitivity across assessment items (Figure 3). Moonshot-v1-128k demonstrated superior recall in population selection (0.92) compared to both ChatGPT-4o (0.55; RD = 0.37, 95% CI: 0.25–0.49, *P* < 0.001) and DeepSeek-V3 (0.54; RD = 0.38, 95% CI: 0.26–0.50, *P* < 0.001). This pattern persisted in prognostic assessment, where Moonshot-v1-128k maintained significantly higher sensitivity (0.90) versus ChatGPT-4o (0.45; RD = 0.45, 95% CI: 0.32–0.58, *P* < 0.001) and DeepSeek-V3 (0.50; RD = 0.40, 95% CI: 0.27–0.53, *P* < 0.001).

ChatGPT-4o and DeepSeek-V3 showed consistently high specificity, exceeding 95% in six of eight assessment items. In contrast, Moonshot-v1-128k demonstrated significantly lower specificity in prognostic assessment (0.75) compared to both ChatGPT-4o (1.00; RD = 0.25, 95% CI: 0.12–0.37, *P* < 0.001) and DeepSeek-V3 (0.95; RD = 0.20, 95% CI: 0.07–0.33, *P* = 0.003). This performance gap was even more pronounced in exposure confidence assessments, where ChatGPT-4o (0.97; RD = 0.13, 95% CI: 0.04–0.22, *P* = 0.006) and DeepSeek-V3 (97.37%; RD = 0.13, 95% CI: 0.04–0.23, *P* = 0.005) substantially outperformed Moonshot-v1-128k (0.84).

DeepSeek-V3 achieved perfect precision (1.00) in three assessment items (population selection, outcome baseline, and follow-up adequacy) but showed complete failure in outcome confidence (0.00).

The comparative *F*-score analysis revealed distinct model-specific competencies across different assessment items. Moonshot-v1-128k demonstrated superior performance in population selection with an F-score of 0.80 (95% CI: 0.72–0.86), significantly outperforming ChatGPT-4o’s 0.67 (95% CI: 0.58–0.74; RD = 0.13, *P* = 0.004). Similarly, in prognostic assessment, Moonshot-v1-128k maintained an advantage (*F*-score = 0.75, 95% CI: 0.67–0.82) over DeepSeek-V3 (*F*-score = 0.625, 95% CI: 0.54–0.70; RD = 0.125, *P* = 0.032). Conversely, ChatGPT-4o exhibited significantly stronger performance in variable adjustment (*F*-score = 0.81, 95% CI: 0.74–0.87) compared to Moonshot-v1-128k (*F*-score = 0.59, 95% CI: 0.50–0.67; RD = 0.22, *P* < 0.001). DeepSeek-V3 showed comparable but nonsignificant performance in co-intervention similarity (*F*-score = 0.775, 95% CI: 0.70–0.84) relative to ChatGPT-4o (*F*-score = 0.76, 95% CI: 0.68–0.82; RD = 0.015, *P* = 0.082).

**Table 1:** Accuracy of assessments

Item	ChatGPT			KimiChat			DeepSeek		
				Correct			Correct			Correct
	No. of	No. of	assessment	No. of	No. of	assessment	No. of	No. of	assessment
	correct	total	rate	correct	total	rate	correct	total	rate
	assessments	assessments	(95% CI)	assessments	assessments	(95% CI)	assessments	assessments	(95% CI)
Population selection	48	60	80.00% (68.22%–88.27%)	49	60	81.67% (70.12%–89.38%)	49	60	81.67% (70.12%–89.38%)
Exposure confidence	47	60	78.33% (66.52%–86.78%)	47	60	78.33% (66.52%–86.78%)	46	60	76.67% (64.85%–85.41%)
Outcome baseline	56	60	93.33% (83.79%–97.49%)	53	60	88.33% (77.83%–94.23%)	54	60	90.00% (79.49%–95.38%)
Variable adjustment	55	60	91.67% (81.61%–96.43%)	46	60	76.67% (64.85%–85.41%)	49	60	81.67% (69.61%–89.77%)
Prognostic assessment	49	60	81.67% (69.61%–89.77%)	48	60	80.00% (68.22%–88.27%)	48	60	80.00% (68.22%–88.27%)
Outcome confidence	54	60	90.00% (79.49%–95.38%)	55	60	91.67% (81.61%–96.43%)	54	60	90.00% (79.49%–95.38%)
Follow-up adequacy	49	60	81.67% (69.61%–89.77%)	50	60	83.33% (72.07%–90.72%)	56	60	93.33% (83.79%–97.49%)
Co-intervention similarity	41	60	68.33% (55.86%–78.66%)	40	60	66.67% (54.14%–77.21%)	42	60	70.00% (57.72%–80.07%)
Overall	399	480	83.13% (79.79%–86.47%)	388	480	80.83% (77.31%–84.35%)	398	480	83.04% (79.68%–86.40%)

### 3.4 Consistency

ChatGPT-4o demonstrated superior interrater reliability compared to both Moonshot-v1-128k and DeepSeek-V3, with statistically significant risk differences observed across all items (ChatGPT-4o mean Cohen’s κ = 96.52%; ChatGPT-4o versus Moonshot-v1-128k: RD = 3.42%, 95% CI: 1.15%–5.69%, *P* = 0.004; ChatGPT-4o versus DeepSeek-V3: RD = 5.49%, 95% CI: 3.22%–7.76%, *P* < 0.001; Table 2). The strongest agreement was achieved in outcome baseline and outcome confidence items, where ChatGPT-4o and DeepSeek-V3 reached perfect agreement (κ = 100%). However, notable variability emerged in co-intervention similarity, with DeepSeek-V3 showing significantly lower agreement (RD = −27.13%, 95% CI −38.87% to −15.39%, *P* < 0.001) compared to ChatGPT-4o’s 88.42% agreement rate.

**Table 2:** Cohen’s kappa and PABAK comparison

	ChatGPT	KimiChat	DeepSeek			
	Cohen’s	Cohen’s	Cohen’s	ChatGPT	KimiChat	DeepSeek
Reviewer	kappa, %	kappa, %	kappa, %	PABAK, %	PABAK, %	PABAK, %
Population selection	100.00	96.19	96.01	100.00	93.33	96.67
Exposure confidence	95.96	88.42	91.86	93.33	80.00	93.33
Outcome baseline	100.00	95.60	100.00	100.00	93.33	100.00
Variable adjustment	96.01	92.26	87.08	93.33	86.67	90.00
Prognostic assessment	95.90	92.38	91.97	93.33	86.67	93.33
Outcome confidence	100.00	95.60	100.00	100.00	93.33	100.00
Follow-up adequacy	95.84	92.06	100.00	93.33	86.67	100.00
Co-intervention similarity	88.42	92.26	61.29	80.00	86.67	66.67
Average	96.52	93.10	91.03	94.17	88.33	92.50

The PABAK results mirrored the Cohen’s κ findings, with ChatGPT-4o maintaining superior performance (mean PABAK = 94.17%) compared to Moonshot-v1-128k (88.33%; RD = 5.84%, 95% CI: 3.21%–8.47%, *P* = 0.001), but showing no significant difference compared to DeepSeek-V3 (RD = 1.67%, 95% CI: −0.96% to 4.30%, *P* = 0.21). ChatGPT-4o and DeepSeek-V3 demonstrated perfect agreement (PABAK = 100%) for outcome confidence assessments, while the poorest performance was again observed in co-intervention similarity, particularly for DeepSeek-V3 (PABAK = 66.67%).

**Figure 4:**
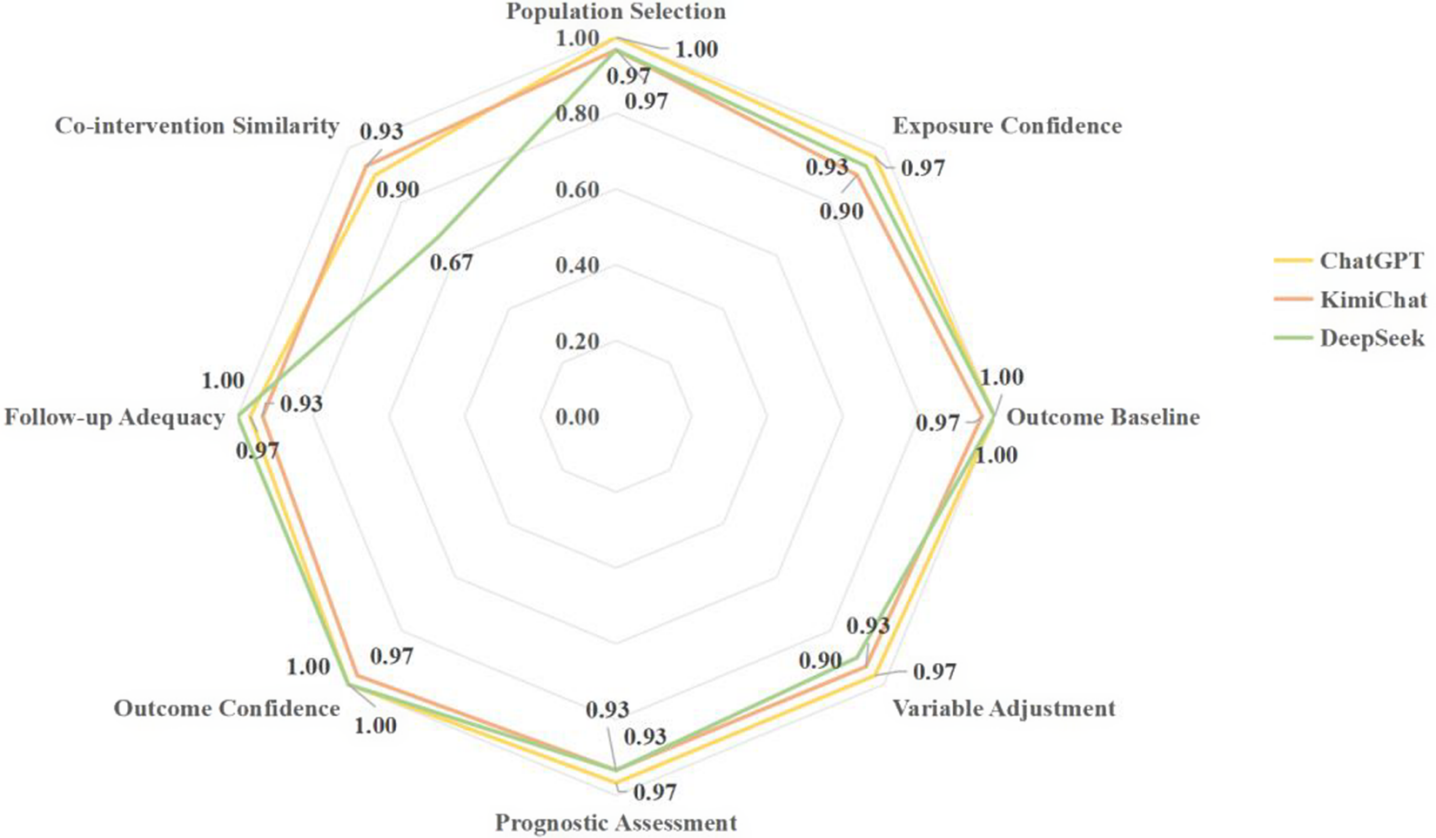
Consistent assessment rate.

As shown in Figure 4, the analysis of consistent assessment rates revealed similar model performance across different assessment items. ChatGPT-4o demonstrated the highest overall consistency (mean = 97.09%), achieving perfect agreement (100%) in population selection, outcome baseline, and outcome confidence. Its lowest consistency was observed in co-intervention similarity at 90.00%. Moonshot-v1-128k showed slightly lower but still strong performance (mean = 94.17%), with its highest consistency in outcome baseline and outcome confidence at 96.67%. The model’s most variable performance occurred in exposure confidence and co-intervention similarity, both at 90.00%. DeepSeek-V3 exhibited comparable overall consistency (mean = 93.00%) when converted to the percentage scale, with perfect scores in outcome baseline, outcome confidence, and follow-up adequacy. However, it showed substantially lower consistency in co-intervention similarity at 67.00%, representing its weakest performance area.

### 3.5 Efficiency

The assessment revealed substantial efficiency gains when using LLMs compared to traditional manual assessment methods. ChatGPT-4o demonstrated the fastest performance, completing assessments in an average of 32.8 seconds per article (range: 22–39 seconds), which is 97.3% faster than the reported 20 minutes per article for manual evaluation.^49^ Similarly, Moonshot-v1-128k (mean: 49.3 seconds) and DeepSeek-V3 (mean: 48.5 seconds) achieved 95.9% and 96.0% time savings over manual methods, respectively.

While all three LLMs significantly outperformed manual processing, ChatGPT-4o showed a clear advantage, being 50.3% faster than Moonshot-v1-128k and 47.9% faster than DeepSeek-V3. These findings highlight the transformative potential of LLMs in accelerating tasks traditionally requiring labor-intensive manual effort.

## 4 Discussion

To explore the feasibility and accuracy of utilizing LLMs to assess ROB in cohort studies, we established a structured and practical prompt framework. We demonstrated that LLMs can provide assessments that closely align with the gold standard results provided by evidence-based methodologists. Despite LLMs exhibiting good consistency, standardization, and efficiency in automating repetitive tasks, researchers may have reservations about using automated tools in systematic reviews^50^ ^,^ ^51^ and the potential need for additional verification and interpretation of results.^52^ This is due to concerns that automation could potentially deprive the evidence synthesis process of human judgment.^53^ It is crucial to emphasize that the aim is not to replace researchers’ professional knowledge and judgment entirely. Instead, they could act as a powerful auxiliary tool in the quality assessment of systematic reviews. Moonshot-v1-128k supports the upload of files (up to 50, each up to 100 MB) and accepts formats such as PDF, DOC, XLSX, PPT, TXT, and images. However, we found that when uploading multiple PDF files, Moonshot-v1-128k can only assess the ROB in studies within a single PDF file at a time, thus requiring researchers to upload PDF files repeatedly. Additionally, we found that DeepSeek-V3 may issue warnings about potential violations of usage guidelines. When this occurs, PDF files must be converted to Word before being uploaded for assessment. Although the assessment time for individual studies is short, this could affect the overall assessment time. In practice, experienced researchers spend an average of 20 minutes per article when conducting both data extraction and RoB assessment.^49^ Systematic reviews typically involve dozens to hundreds of articles, and tasks like assessing ROB are usually done independently by two researchers. Shortening the time for ROB assessment can improve review efficiency.

The lowest correct assessment rate for LLMs in ROB assessments was observed in the item of intervention similarity, with both assessments failing to exceed 70%. When using LLMs to assess ROB in cohort studies, the accuracy of the assessment may be reduced if the original text is reported vaguely. This is because the assessment capabilities of LLMs largely depend on the quality and completeness of the original text. If the original text is unclear or insufficient, LLMs may fail to accurately assess bias in the study. Experts often use their professional knowledge, experience, and additional sources to interpret and evaluate unclear information. In contrast, LLMs lack this expertise and complex contextual processing, assessing based on predefined algorithms and training data without human-like reasoning or information-seeking.

As the first study using the modified NOS tool to explore the feasibility of applying LLMs to the assessment of ROB in cohorts, this research investigated various aspects of LLMs technology, including accuracy, consistency, and efficiency. Our findings suggest integrating LLMs with human review in systematic reviews. LLMs can serve as initial screeners for cohort ROB assessments, with human reviewers validating LLM outputs. Comparing LLM and human assessments on a subset of studies can identify inconsistencies and refine criteria. An iterative feedback loop, where human experts review LLM assessments and provide feedback to fine-tune prompts, can enhance LLM performance over time. This approach combines LLM efficiency with human expertise, improving accuracy and efficiency in systematic reviews.

However, the study is not without limitations. First, there may be some degree of subjectivity in the assessment criteria. Although the modified Cochrane tool provides guidelines for assessment, in certain circumstances, assessors may need to make judgments of “probably yes” or “probably no” based on insufficient information, which could introduce subjectivity. Second, executing this instruction requires the assessor to have professional medical research knowledge and a deep understanding of the modified tool to ensure the accuracy and consistency of the assessment. Third, LLMs may not fully consider the context and environmental factors of the research, which could have significant impacts on the interpretation of the results and the assessment of ROB. Fourth, the extent to which LLMs can benefit researchers in practical uses has not yet been rigorously assessed. Fifth, the sample size of 30 cohort studies is relatively small, which may limit the generalizability of our findings. Although these studies cover a range of different items, a larger sample size would provide a more robust basis for assessing the feasibility and accuracy of utilizing LLMs to assess ROB. Future research should consider expanding the sample size to include a more diverse and extensive range of cohort studies to enhance the validity and reliability of the results. Sixth, while our gold standard assessments benefited from rigorous consensus among three methodologists, the lack of formal interrater reliability statistics may affect the interpretability of model-versus-human comparisons. Future studies could strengthen validity testing by incorporating both consensus judgments and independent ratings with reliability metrics. We proposed this feasibility study to assess the practical utility of LLMs in accelerating the synthesis of biomedical evidence.

## 5 Conclusions

Our research found that the ROB assessment in cohort studies, DeepSeek-V3, ChatGPT-4o and Moonshot-v1-128k, through its machine learning technologies, can efficiently handle and analyze large amounts of data, significantly simplifying mechanical and standardized tasks within the ROB assessment process. LLMs assist human reviewers in conducting more in-depth and accurate assessments. It can automatically review literature and provide preliminary assessment results and judgment reasons regarding ROB, helping human reviewers quickly identify potential issues, thereby enhancing the overall accuracy and efficiency of the assessment.

LLMs continuously optimize themselves to meet the needs of future scientific research. At the same time, it works closely with human researchers to jointly promote the development and innovation of research methods.

## Supporting information

Xia et al. supplementary material Xia et al. supplementary material

## Data Availability

All data generated or analyzed during this study are included in this published article and its supplementary information files. Additional details or specific datasets from the analysis can be made available by the corresponding author upon reasonable request.

## Supplementary material

To view supplementary material for this article, please visit http://doi.org/10.1017/rsm.2025.10028.
