# Supplementary material for: Assessing risk of bias of cohort studies with large language models
Source: Res Synth Methods. 2025 Aug 7;16(6):990–1004. doi: 10.1017/rsm.2025.10028 (PMC12657654; doi:10.1017/rsm.2025.10028)
Supplement: Xia et al. supplementary material [file S1759287925100288sup001.docx]

Supplementary Materials

**Assessing Risk of Bias of Cohort Studies with Large Language Models**

**Table of contents**

[Appendix 1 Detailed search strategy 3](#_Toc17421)

[Appendix 2 Tool to Assess Risk of Bias in Cohort Studies 4](#_Toc23008)

[Appendix 3 Instructions 8](#_Toc10094)

[Appendix 4 The output results. 16](#_Toc23392)

[Table S1 ChatGPT's assessment results. 221](#_Toc19623)

[Table S2 KimiChat's assessment results. 222](#_Toc16247)

[Table S3 DeepSeek's assessment results. 2](#_Toc2869)23

**Appendix 1** **Detailed search strategy**

**Database: Cochrane Library** < 2020.1.1-2023.10.8> 57

Appendix 2 Tool to Assess Risk of Bias in Cohort Studies

Contributed by the CLARITY Group at McMaster University

**1. Was selection of exposed and non-exposed cohorts drawn from the same population?**

### **Definitely yes (low risk of bias)**

### **Probably yes**

### **Probably no**

### **Definitely no (high risk of bias)**

**Examples of low risk of bias:**

- Exposed and unexposed drawn for same administrative data base of patients presenting at same points of care over the same time frame

**Examples of high risk of bias:**

- Exposed and unexposed presenting to different points of care over a different time frame

**2. Can we be confident in the assessment of exposure?**

### **Definitely yes (low risk of bias)**

### **Probably yes**

### **Probably no**

### **Definitely no (high risk of bias)**

**Examples of low risk of bias:**

- Secure record (e.g. surgical records, pharmacy records)
- Repeated interview or other ascertainment asking about current use/exposure

**Examples of higher risk of bias:**

- Structured interview at a single point in time
- Written self report
- Individuals who are asked retrospectively confirm their exposure status may be subject to recall bias – less likely to recall an exposure if they have not developed an adverse outcome, and more likely to recall an exposure (whether an exposure occurred or not) if they have developed an adverse outcome

**Examples of high risk of bias:**

- Uncertain how exposure information obtained

**3. Can we be confident that the outcome of interest was not present at start of study?**

### **Definitely yes (low risk of bias)**

### **Probably yes**

### **Probably no**

### **Definitely no (high risk of bias)**

**4. Did the study match exposed and unexposed for all variables that are associated with the outcome of interest or did the statistical analysis adjust for these prognostic variables?**

### **Definitely yes (low risk of bias)**

### **Probably yes**

### **Probably no**

### **Definitely no (high risk of bias)**

**Examples of low risk of bias:**

- Comprehensive matching or adjustment for all plausible prognostic variables

**Examples of higher risk of bias:**

- Matching or adjustment for most plausible prognostic variables

**Examples of high risk of bias:**

- Matching or adjustment for a minority of plausible prognostic variables
- No matching or adjustment of plausible prognostic variables
- Statements of no differences between groups
- Statements that differences were not statistically significant are not sufficient for establishing comparability

**5. Can we be confident in the assessment of the presence or absence of prognostic factors?**

### **Definitely yes (low risk of bias)**

### **Probably yes**

### **Probably no**

### **Definitely no (high risk of bias)**

**Examples of low risk of bias:**

- Interview of all participants
- Self-completed survey from all participants
- Review of charts with reproducibility demonstrated
- From data base with documentation of accuracy of abstraction of prognostic data

**Examples of higher risk of bias:**

- Chart review without demonstration of reproducibility
- Data base with uncertain quality of abstraction of prognostic information

**Examples of high risk of bias:**

- Prognostic information from data base with no available documentation of quality of abstraction of prognostic variables

**6. Can we be confident in the assessment of outcome?**

### **Definitely yes (low risk of bias)**

### **Probably yes**

### **Probably no**

### **Definitely no (high risk of bias)**

**Examples of low risk of bias:**

- Independent blind assessment
- Record linkage
- For some outcomes (e.g. fractured hip), reference to the medical record is sufficient to satisfy the requirement for confirmation of the fracture

**Examples of higher risk of bias:**

- Independent assessment unblinded
- Self-report
- For some outcomes (e.g. vertebral fracture where reference to x-rays would be required) reference to the medical record would not be adequate outcomes

**Examples of high risk of bias:**

- Uncertain (no description)

**7. Was the follow up of cohorts adequate?**

### **Definitely yes (low risk of bias)**

### **Probably yes**

### **Probably no**

### **Definitely no (high risk of bias)**

**Examples of low risk of bias:**

- No missing outcome data
- Reasons for missing outcome data unlikely to be related to true outcome (for survival data, censoring is unlikely to introduce bias)
- Missing outcome data balanced in numbers across intervention groups, with similar reasons for missing data across groups
- For dichotomous outcome data, the proportion of missing outcomes compared with observed event risk is not enough to have an important impact on the intervention effect estimate
- For continuous outcome data, plausible effect size (difference in means or standardized difference in means) among missing outcomes is not large enough to have an important impact on the observed effect size
- Missing data have been imputed using appropriated methods

**Examples of high risk of bias:**

- Reason for missing outcome data likely to be related to true outcome, with either imbalance in numbers or reasons for missing data across intervention groups
- For dichotomous outcome data, the proportion of missing outcomes compared with observed event risk is enough to induce important bias in intervention effect estimate
- For continuous outcome data, plausible effect size (difference in means or standardized difference in means) among missing outcomes is large enough to induce clinically relevant bias in the observed effect size

**8. Were co-interventions similar between groups?**

### **Definitely yes (low risk of bias)**

### **Probably yes**

### **Probably no**

### **Definitely no (high risk of bias)**

**Examples of low risk of bias:**

- Most or all relevant co-interventions that might influence the outcome of interest are documented to be similar in the exposed and unexposed

**Examples of high risk of bias:**

- Few or no relevant co-interventions that might influence the outcome of interest are documented to be similar in the exposed and unexposed

Appendix 3 Instructions

I want you to roleplay as a professional evidence-based medical researcher, especially a reviewer who is proficient in using the modified NOS tool standards to accurately assess the risk of bias in cohort studies. Next, I will first introduce the eight items of using the modified NOS tool standards to assess the risk of bias in cohort studies. After you understand, I will provide you with a cohort study paper. Then you need to use the modified NOS tool standards to assess the risk of bias in this cohort study and output the assessment results as required. Each question is answered with "Definitely yes (low risk of bias).", "Probably yes.", "Probably no." or "Definitely no (high risk of bias).". If the information provided in the original text is not sufficient enough to judge as "Definitely yes (low risk of bias)." or "Definitely no (high risk of bias).", "Probably yes." or "Probably no." can be used for judgment. All information needs to be extracted accurately.

General Guidelines: If confident about an item's accuracy (>90%), follow the subsequent detailed instructions. Responses can be: Semi-free format: consistent with example format; Cited the corroborative sentence with double quotation marks or elucidate the reasoning without quotes. In addition, if an article indicates that there are multiple groups of cohorts, conduct bias risk assessment for each Cohort separately, fill in and output the following content: "Cohort name: _______________." For example, the article states that "We used data from 3 prospective cohort studies: the Health Professionals Follow-up Study (HPFS; age range, 32-87 years) and the Nurses’ Health Study (NHS) I (age range, 37-65 years) and II (age range, 26-45 years).” , the following content is displayed: "Cohort name: HPFS."; "Cohort name: NHS I."; "Cohort name: NHS II." and different cohorts were evaluated for risk of bias.

The specific requirements are as follows:

1. Item 1 name: Was selection of exposed and non-exposed cohorts drawn from the same population?

1.1 Item 1 evaluation content: Whether the study populations included in the exposed and non-exposed cohorts of the queue come from the same population, and how representative they are.

1.2 Item 1 judgment criteria: Whether the study populations included in the exposed and non-exposed cohorts of the queue come from the same database or population, with good representatives and comparability.

1.3 Item 1 output content: If the study populations included in the exposed and non-exposed cohorts are drawn from the same administrative database of patients presenting at the same points of care over the same time frame, output the following: “Definitely yes (low risk of bias). Exposed and unexposed drawn for same administrative data base of patients presenting at same points of care over the same time frame.”; If the study populations included in the exposed and non-exposed cohorts received different treatments in different time periods and geographical areas, output the following: “Definitely no (high risk of bias). Exposed and unexposed presenting to different points of care over a different time frame.”; If the information provided in the original text is not sufficient enough to judge as “Definitely yes (low risk of bias).” or “Definitely no (high risk of bias).”, does not clearly describe whether it is the same or different population, just simply mentions the source of the population, “Probably yes.” can be output; If the study does not describe the source of the population, output the following: “Probably no.”.

2. Item 2 name: Can we be confident in the assessment of exposure?

2.1 Item 2 evaluation content: How to determine the existence of exposure

2.2 Item 2 judgment criteria: Whether there are records or repeated interviews to determine exposure

2.3 Item 2 output content: If the article mentions that there are secure records such as surgical records, medication records, or repeated interviews and other inquiries to determine current use or exposure, output the following: “Definitely yes (low risk of bias). ”; If the article mentions that a one-time structured interview, written self-report, retrospective confirmation of exposure status of individuals may be subject to recall bias, output the following: “Probably yes.”; If the study is uncertain how exposure information was obtained, output the following: “Definitely no (high risk of bias). Uncertain how exposure information obtained.” If not described how exposure information was obtained, output the following: “Probably no.”

3. Item 3 name: Can we be confident that the outcome of interest was not present at start of study?

3.1 Item 3 evaluation content: Confirm that the outcome of interest did not exist at the beginning of the study.

3.2 Item 3 judgment criteria: Whether the outcome of interest in this study already existed at the start of the study.

3.3 Item 3 output content: If study participants did not have the outcome of interest at baseline, output the following: “Definitely yes (low risk of bias).”; If study participants already had the outcome of interest at baseline, output the following: “Definitely no (high risk of bias).”. If the authors have made efforts to exclude participants with the outcome of interest at baseline. The results are self-reported, with no external validation, or the authors do not report how they determined outcomes at baseline, output the following: “Probably yes.”

4. Item 4 name: Did the study match exposed and unexposed for all variables that are associated with the outcome of interest or did the statistical analysis adjust for these prognostic variables?

4.1 Item 4 evaluation content: Whether the study matched the exposed and unexposed cohorts for all variables associated with the outcome of interest, or whether the statistical analysis adjusted for these prognostic variables.

4.2 Item 4 judgment criteria: Whether the exposed and unexposed cohorts in the queue are matched for all possible confounding factors that may affect the outcome of interest.

4.3 Item 4 output content: If the article states that comprehensive matching or adjustment was done for all prognostic variables, output the following: “Definitely yes (low risk of bias).”; If the article states that only the most reasonable prognostic variables were matched or adjusted, output the following: “Probably yes.”; If the article only matched or adjusted for a few or no reasonable prognostic variables, statements that there were no differences between groups or differences that were not statistically significant are insufficient to establish comparability, output the following: “Probably no.” If the study did not adjust for any prognostic variables associated with the outcome, output the following: “Definitely no (high risk of bias).”

5. Item 5 name: Can we be confident in the assessment of the presence or absence of prognostic factors?

5.1 Item 5 evaluation content: Accuracy in assessing prognostic factors

5.2 Item 5 judgment criteria: Accuracy and repeatability of data quality of prognostic factors

5.3 Item 5 output content: If the article states that prognostic factors were obtained by interviewing all participants, self-completed surveys by all participants, extracted from retrospective charts and demonstrated repeatability, or by extracting prognostic data from a database with documented accuracy, output the following: “Definitely yes (low risk of bias).”; If the article states that prognostic factors were obtained through retrospective charts but repeatability could not be demonstrated or from a database where prognostic information quality is uncertain, output the following: “Probably no.”; If the article states that prognostic factors came from a database where prognostic information quality was very poor, output the following: “Definitely no (high risk of bias).”

6. Item 6 name: Can we be confident in the assessment of outcome?

6.1 Item 6 evaluation content: Evaluation of outcome indicators

6.2 Item 6 judgment criteria: Determine whether the outcome indicator is blind independent evaluation, whether there is archival record, whether it is self-reported by the researcher, or whether the study describes the evaluation of the outcome indicator.

6.3 Item 6 output content: If the article states that the outcome indicator is blind independent evaluation, has record linkage, for some outcomes (such as hip fracture), referencing medical records is sufficient to meet the requirements for confirming fracture, output the following: “Definitely yes (low risk of bias).”; If the article states that the outcome indicator is non-blind independent evaluation, self-report, for some outcomes (such as vertebral fractures that require x-rays), referencing medical records is insufficient, output the following: “Probably yes.”; If the outcome indicator is medical records without review by the research physician or researcher, the author does not specifically describe how to measure the results, self-report with external validation of medical records, where the consistency between self-report and medical records is low, output the following: “Probably no.”; If the article does not describe the outcome indicator at all, output the following: “Definitely no (high risk of bias).”

7. Item 7 name: Was the follow up of cohorts adequate?

7.1 Item 7 evaluation content: Completeness of cohort follow-up, that is, whether there are lost visits during the entire follow-up process.

7.2 Item 7 judgment criteria: Whether the loss to follow-up of study subjects during follow-up will cause bias.

7.3 Item 7 output content: If the article has no missing outcome data or the situation of missing outcome data is: 1) The reasons for missing outcome data are unlikely to be related to the true outcome (for survival data, unlikely to introduce bias), 2) The missing outcome data is balanced between intervention groups, the reasons for missing data are similar between groups, 3) For binary outcome data, the proportion of missing outcomes compared to observed risk of events is not enough to cause important impact on the estimated intervention effect, 4) For continuous outcome data, the effect size between missing results (mean difference or standardized mean difference) is not enough to cause important impact on the observed effect size, 5) Missing data has been input by appropriate methods, output the following: “Definitely yes (low risk of bias).”; If the situation of missing outcome data in the article is: 1) The reasons for missing outcome data may be related to the true outcome, either unbalanced in quantity or in reasons for missing data between intervention groups, 2) For binary outcome data, the proportion of missing outcomes compared to observed risk of events is enough to cause important bias in estimated intervention effect, 3) For continuous outcome data, the plausible effect size between missing results (mean difference or standardized mean difference) is large enough to cause clinically relevant bias in the observed effect size, output the following: “Definitely no (high risk of bias).”; If the article does not describe missing follow-up or missing results, output the following: “Probably yes.”; If follow-up rate during the study is 80-89%, losses to follow-up may be related to outcomes, or subsequent losses are not reported or cannot be estimated from information in the article, output the following: “Probably no.”

8. Item 8 name: Were co-interventions similar between groups?

8.1 Item 8 evaluation content: Whether co-interventions between groups are similar.

8.2 Item 8 judgment criteria: Co-interventions that may affect the outcome of interest are similar in the exposed and unexposed groups which can reduce the risk of bias.

8.3 Item 8 output content: If most or all co-interventions that may affect the outcome of interest are similar in the exposed and unexposed cohorts of the queue, output the following: “Definitely yes (low risk of bias). Most or all relevant co-interventions that might influence the outcome of interest are documented to be similar in the exposed and unexposed.”; If few or none of the co-interventions that may affect the outcome of interest are similar in the exposed and unexposed cohorts of the queue, output the following: “Definitely no (high risk of bias). Few or no relevant co-interventions that might influence the outcome of interest are documented to be similar in the exposed and unexposed.” If the information provided in the original text is not sufficient enough to judge as “Definitely yes (low risk of bias).” or “Definitely no (high risk of bias).”, does not clearly describe whether the co-interventions between exposed and unexposed groups are similar, just simply describes the co-interventions used in each group, “Probably yes.” can be output; If the study does not describe, output the following: “Probably no.”.

First, please evaluate the risk of bias in the cohort study according to the above requirements. Each question needs to be output in the format of "Item number; Question title; Item evaluation result; Basis for item answer; Quote from original text". If the study does not describe a certain item, then fill in "NR" in the "Fill in the original sentence" part of that item. For example, I will use "Item 1; Was selection of exposed and non-exposed cohorts drawn from the same population; Definitely yes (low risk of bias).; From the same database; Exposed and unexposed drawn for same administrative data base of patients presenting at same points of care over the same time frame." as the output result for whether the exposed and unexposed cohorts come from the same population in item 1. Then I will output the results of the assessment of the risk of bias in the article in the format of "ID: First author of article, year of publication; Result of assessment of article bias risk; Item 1: Result of Item 1 evaluation; Item 2: Result of Item 2 evaluation.......". For example, I will use "ID: Anderson, 2017; overall: low risk; Item 1: Definitely yes (low risk of bias).; Item 2: Definitely no (high risk of bias)......." as the final output result of the article.

The final format is presented as follows:

Title:

Cohort name:_______________

Item number; Item title; Item evaluation result; quote: original text

......

Item number; Item title; Item evaluation result; quote: original text

ID: First author of article, year of publication ;

Cohort name:_______________

Result of assessment of article bias risk

Item 1: Result of Item 1 evaluation

Item 2: Result of Item 2 evaluation

Item 3: Result of Item 3 evaluation

Item 4: Result of Item 4 evaluation

Item 5: Result of Item 5 evaluation

Item 6: Result of Item 6 evaluation

Item 7: Result of Item 7 evaluation

Item 8: Result of Item 8 evaluation

For example:

Title: Robot-assisted partial nephrectomy does not benefit short-term renal function: a matched cohort analysis

Cohort name:off-clamp RAPN patients and clamped RAPN patients.

Item 1; Was selection of exposed and non-exposed cohorts drawn from the same population; Definitely yes (low risk of bias); From the same database; “After Institutional Review Board approval, we retrospectively reviewed a prospectively maintained database of patients who underwent RAPN by a single surgeon (RSF) at our institution from January 2009 to September 2015.”

Item 2; Can we be confident in the assessment of exposure; Definitely no (high risk of bias).; Study is uncertain how exposure information was obtained; NR

......

ID: Anderson, 2017;

Cohort name: off-clamp RAPN patients and clamped RAPN patients.

Overall: Low risk

Item 1: Definitely yes (low risk of bias).

Item 2: Definitely no (high risk of bias).

Item 3: Definitely yes (low risk of bias).

Item 4: Definitely yes (low risk of bias).

Item 5: Probably yes.

Item 6: Definitely yes (low risk of bias).

Item 7: Definitely yes (low risk of bias).

Item 8: Probably yes.

Appendix 4 The output results.

**ChatGPT**

Title: Anticholinergic Drug Use and Negative Outcomes Among the Frail Elderly Population Living in a Nursing Home

**First response:**

**Item 1; Was selection of exposed and non-exposed cohorts drawn from the same population;** Probably yes; The exposed and unexposed groups were drawn from a multicenter study (U.L.I.S.S.E project) including 31 facilities in Italy. However, the article does not explicitly confirm that the populations were identical; "Patients from 31 facilities in Italy were assessed at baseline and at 6 and 12 months..."

**Item 2; Can we be confident in the assessment of exposure;** Definitely yes (low risk of bias); The Anticholinergic Risk Scale (ARS), a validated tool, was used to assess drug exposure, and trained personnel collected data systematically; "The ARS is a validated tool... trained study personnel collected detailed information about medication use during nursing home assessment using a structured questionnaire embedded in the MDS-NH form."

**Item 3; Can we be confident that the outcome of interest was not present at start of study;** Probably yes; Efforts were made to exclude participants with outcomes at baseline, but external validation was not described; "Functional decline was defined as a reduction of at least 1 point in the summary ADL score during follow-up."

**Item 4; Did the study match exposed and unexposed for all variables that are associated with the outcome of interest or did the statistical analysis adjust for these prognostic variables;** Definitely yes (low risk of bias); Comprehensive statistical adjustments were performed for potential confounders; "After adjustment for potential confounders, which included age, gender, comorbidity, baseline functional impairment, and cognitive impairment..."

**Item 5; Can we be confident in the assessment of the presence or absence of prognostic factors;** Probably yes; Prognostic factors were obtained through validated instruments, but no explicit details about repeatability or database accuracy were provided; "The MDS-NH contains more than 350 data elements, including sociodemographic variables, numerous clinical items about both physical and cognitive status, as well as all clinical diagnoses."

**Item 6; Can we be confident in the assessment of outcome;** Probably yes; Outcomes such as functional decline and falls were assessed through validated scales, but no blind independent evaluations were mentioned; "Functional decline was defined as a reduction of at least 1 point in the summary ADL score during follow-up."

**Item 7; Was the follow-up of cohorts adequate;** Probably yes; Follow-up assessments were conducted at 6 and 12 months, but missing data and its impact on bias were not extensively discussed; "These outcomes were all assessed through MDS-NH form both at 6 and 12 months."

**Item 8; Were co-interventions similar between groups;** Probably no; The study does not describe the co-interventions between groups explicitly; NR.

ID: Landi, 2014; Cohort name: Frail elderly nursing home residents

Item 1: Probably yes.

Item 2: Definitely yes (low risk of bias).

Item 3: Probably yes.

Item 4: Definitely yes (low risk of bias).

Item 5: Probably yes.

Item 6: Probably yes.

Item 7: Probably yes.

Item 8: Probably no.

**Second response:**

**Item 1; Was selection of exposed and non-exposed cohorts drawn from the same population;** Probably yes; Both exposed and unexposed groups were drawn from the same database within the U.L.I.S.S.E. project, encompassing 31 facilities. However, it is not explicitly stated that the two groups were fully comparable; "Patients from 31 facilities in Italy were assessed at baseline and at 6 and 12 months..."

**Item 2; Can we be confident in the assessment of exposure;** Definitely yes (low risk of bias); The Anticholinergic Risk Scale (ARS), a validated tool, was used for assessment, and trained personnel systematically collected data; "The ARS is a validated tool... trained study personnel collected detailed information about medication use during nursing home assessment using a structured questionnaire embedded in the MDS-NH form."

**Item 3; Can we be confident that the outcome of interest was not present at start of study;** Probably yes; Functional decline and other outcomes were assessed during follow-up, but it was not explicitly stated how baseline exclusion of outcomes was validated; "Functional decline was defined as a reduction of at least 1 point in the summary ADL score during follow-up."

**Item 4; Did the study match exposed and unexposed for all variables that are associated with the outcome of interest or did the statistical analysis adjust for these prognostic variables;** Definitely yes (low risk of bias); The analysis adjusted for potential confounders including age, gender, comorbidity, and baseline functional impairment; "After adjustment for potential confounders, which included age, gender, comorbidity, baseline functional impairment, and cognitive impairment..."

**Item 5; Can we be confident in the assessment of the presence or absence of prognostic factors;** Probably yes; Data was collected using the validated MDS-NH, though repeatability and database accuracy were not explicitly stated; "The MDS-NH contains more than 350 data elements, including sociodemographic variables, numerous clinical items about both physical and cognitive status, as well as all clinical diagnoses."

**Item 6; Can we be confident in the assessment of outcome;** Probably yes; Functional decline, falls, and delirium were measured using validated scales, though blinding or independent verification is not mentioned; "Functional decline was defined as a reduction of at least 1 point in the summary ADL score during follow-up."

**Item 7; Was the follow-up of cohorts adequate;** Probably yes; Follow-up assessments were conducted at 6 and 12 months, but missing data and its impact on bias were not explicitly addressed; "These outcomes were all assessed through MDS-NH form both at 6 and 12 months."

**Item 8; Were co-interventions similar between groups;** Probably no; The study does not describe the similarity of co-interventions that could affect outcomes; NR.

ID: Landi, 2014; Cohort name: Frail elderly nursing home residents

Item 1: Probably yes.

Item 2: Definitely yes (low risk of bias).

Item 3: Probably yes.

Item 4: Definitely yes (low risk of bias).

Item 5: Probably yes.

Item 6: Probably yes.

Item 7: Probably yes.

Item 8: Probably no.

Title: Chronic Exposure to Anticholinergic Medications Adversely Affects the Course of Alzheimer Disease

**First response:**

**Item 1; Was selection of exposed and non-exposed cohorts drawn from the same population;** Probably yes; Both exposed and unexposed groups were selected from the same database of Alzheimer disease patients at the Emory University Alzheimer's Disease Center, but representativeness of the population was not discussed; "This was a retrospective study in which participants were identified through the Emory University Alzheimer's Disease Center (ADC) database."

**Item 2; Can we be confident in the assessment of exposure;** Probably yes; Anticholinergic medications were identified using previously published lists, but details about the accuracy of medication data collection were limited; "Anticholinergic medications were identified from previously published lists, on the basis of antimuscarinic activity in a radioreceptor assay."

**Item 3; Can we be confident that the outcome of interest was not present at start of study;** Definitely yes (low risk of bias); The baseline Mini-Mental State Exam (MMSE) scores for both groups were comparable, ensuring no pre-existing differences in cognitive decline; "There was no significant difference between the baseline MMSE scores for the two groups..."

**Item 4; Did the study match exposed and unexposed for all variables that are associated with the outcome of interest or did the statistical analysis adjust for these prognostic variables;** Probably no; No matching or adjustments were reported for potential confounders beyond descriptive comparisons between groups; NR.

**Item 5; Can we be confident in the assessment of the presence or absence of prognostic factors;** Probably no; While descriptive data was collected on patient characteristics, repeatability or database accuracy was not confirmed; "There were no significant differences between the individuals receiving no anticholinergic medications and those receiving one or more anticholinergic medications with regard to age, gender, education level..."

**Item 6; Can we be confident in the assessment of outcome;** Probably yes; MMSE was used to assess outcomes, which is a validated tool, but blinding of evaluators or independent verification was not described; "The primary measure in this study is the MMSE scale. The MMSE scale is used to determine the severity of the patient's cognitive impairment..."

**Item 7; Was the follow-up of cohorts adequate;** Probably yes; Follow-up was conducted at one and two years, but the article did not elaborate on missing data or losses to follow-up; "MMSE scores were collected at the baseline visit into the ADC program and at the subsequent two follow-up visits."

**Item 8; Were co-interventions similar between groups;** Probably no; Co-interventions that could affect outcomes, such as additional treatments or caregiver support, were not addressed; "Other limitations include other clinical factors that may contribute to the rate of decline, including behavioral and psychological symptoms of dementia and level of caregiver support."

ID: Lu, 2003; Cohort name: Alzheimer disease (AD) patients treated with or without anticholinergic medications

Item 1: Probably yes.

Item 2: Probably yes.

Item 3: Definitely yes (low risk of bias).

Item 4: Probably no.

Item 5: Probably no.

Item 6: Probably yes.

Item 7: Probably yes.

Item 8: Probably no.

**Second response:**

**Item 1; Was selection of exposed and non-exposed cohorts drawn from the same population;** Probably yes; Both exposed and unexposed groups were selected from the same database at the Emory University Alzheimer's Disease Center, but representativeness of the population was not discussed; "This was a retrospective study in which participants were identified through the Emory University Alzheimer's Disease Center (ADC) database."

**Item 2; Can we be confident in the assessment of exposure;** Probably yes; Anticholinergic medications were identified using published lists, but details about the accuracy of medication data collection were limited; "Anticholinergic medications were identified from previously published lists, on the basis of antimuscarinic activity in a radioreceptor assay."

**Item 3; Can we be confident that the outcome of interest was not present at start of study;** Definitely yes (low risk of bias); Baseline MMSE scores were reported to be comparable between groups, confirming no significant pre-existing differences; "There was no significant difference between the baseline MMSE scores for the two groups..."

**Item 4; Did the study match exposed and unexposed for all variables that are associated with the outcome of interest or did the statistical analysis adjust for these prognostic variables;** Probably no; No explicit matching or statistical adjustment for confounders was reported; NR.

**Item 5; Can we be confident in the assessment of the presence or absence of prognostic factors;** Probably no; While descriptive characteristics were collected, repeatability and validation of data accuracy were not addressed; "There were no significant differences between the individuals receiving no anticholinergic medications and those receiving one or more anticholinergic medications with regard to age, gender, education level..."

**Item 6; Can we be confident in the assessment of outcome;** Probably yes; MMSE is a validated tool, but no mention of blinding or independent assessment is included; "The primary measure in this study is the MMSE scale. The MMSE scale is used to determine the severity of the patient's cognitive impairment..."

**Item 7; Was the follow-up of cohorts adequate;** Probably yes; Follow-up was conducted at one and two years, but missing data and its impact were not discussed; "MMSE scores were collected at the baseline visit into the ADC program and at the subsequent two follow-up visits."

**Item 8; Were co-interventions similar between groups;** Probably no; Co-interventions that might impact outcomes, such as other treatments or caregiver support, were not described; "Other limitations include other clinical factors that may contribute to the rate of decline, including behavioral and psychological symptoms of dementia and level of caregiver support."

ID: Lu, 2003; Cohort name: Alzheimer disease (AD) patients treated with or without anticholinergic medications

Item 1: Probably yes.

Item 2: Probably yes.

Item 3: Definitely yes (low risk of bias).

Item 4: Probably no.

Item 5: Probably no.

Item 6: Probably yes.

Item 7: Probably yes.

Item 8: Probably no.

Title: Prognostic factors associated with mortality risk and disease progression in critically ill patients with COVID-19 in Europe

**First response:**

**Item 1; Was selection of exposed and non-exposed cohorts drawn from the same population;** Definitely no (high risk of bias); All patients were enrolled from ICUs participating in the RISC-19-ICU registry, ensuring consistency in inclusion criteria; "The cohort included critically ill patients from 36 ICUs across six European countries."

**Item 2; Can we be confident in the assessment of exposure;** Definitely yes (low risk of bias); Exposure to ICU interventions and baseline conditions were documented systematically using a harmonized data collection process; "Detailed ICU data, including oxygenation levels, laboratory markers, and treatments, were recorded."

**Item 3; Can we be confident that the outcome of interest was not present at start of study;** Definitely yes (low risk of bias); ICU mortality and progression were tracked prospectively, with no pre-existing outcomes; "Patients were followed from ICU admission to discharge or death."

**Item 4; Did the study match exposed and unexposed for all variables that are associated with the outcome of interest or did the statistical analysis adjust for these prognostic variables;** Definitely yes (low risk of bias); Multivariable Cox regression adjusted for key risk factors such as oxygenation, renal function, and comorbidities; "Predictors like P/F-ratio, creatinine, and d-dimer were adjusted in the analysis."

**Item 5; Can we be confident in the assessment of the presence or absence of prognostic factors;** Definitely yes (low risk of bias); Comprehensive baseline data were collected on clinical and laboratory variables using standardized methods; "Baseline characteristics included oxygenation parameters, renal markers, and comorbidities."

**Item 6; Can we be confident in the assessment of outcome;** Definitely yes (low risk of bias); Outcomes such as ICU mortality were objectively defined and documented based on international criteria; "Mortality was assessed as ICU death or discharge status."

**Item 7; Was the follow-up of cohorts adequate;** Definitely yes (low risk of bias); Patients were followed until ICU discharge or death, ensuring outcome data completeness; "Follow-up was complete for all enrolled patients by the study's end."

**Item 8; Were co-interventions similar between groups;** Probably yes; Although treatment strategies varied, these were accounted for in subgroup analyses; "Differences in off-label therapies and interventions were described and analyzed."

ID: Wendel Garcia, 2020; Cohort name: Critically ill COVID-19 patients admitted to ICUs across Europe

Item 1: Definitely no (high risk of bias).

Item 2: Definitely yes (low risk of bias).

Item 3: Definitely yes (low risk of bias).

Item 4: Definitely yes (low risk of bias).

Item 5: Definitely yes (low risk of bias).

Item 6: Definitely yes (low risk of bias).

Item 7: Definitely yes (low risk of bias).

Item 8: Probably yes.

**Second response:**

**Item 1; Was selection of exposed and non-exposed cohorts drawn from the same population;** Definitely no (high risk of bias); Patients were included from ICUs in 36 hospitals across Europe, following uniform inclusion criteria based on critical illness and SARS-CoV-2 infection; "The RISC-19-ICU registry enrolled patients from multiple ICUs in six European countries."

**Item 2; Can we be confident in the assessment of exposure;** Definitely yes (low risk of bias); Detailed and standardized data collection ensured accurate capture of clinical variables, including ICU interventions and baseline characteristics; "Variables like creatinine, oxygenation parameters, and treatments were documented systematically."

**Item 3; Can we be confident that the outcome of interest was not present at start of study;** Definitely yes (low risk of bias); ICU mortality and disease progression were assessed during the study, and all patients were critically ill upon admission; "Outcomes were tracked prospectively from ICU admission to death or discharge."

**Item 4; Did the study match exposed and unexposed for all variables that are associated with the outcome of interest or did the statistical analysis adjust for these prognostic variables;** Definitely yes (low risk of bias); Multivariable Cox regression adjusted for key factors like P/F-ratio, lactate, and comorbidities; "Proportional hazard models included clinical and laboratory parameters associated with mortality risk."

**Item 5; Can we be confident in the assessment of the presence or absence of prognostic factors;** Definitely yes (low risk of bias); Comprehensive baseline data on ICU admission were recorded using consistent methods, covering relevant laboratory and clinical markers; "Baseline prognostic factors included renal and oxygenation markers, along with comorbidities."

**Item 6; Can we be confident in the assessment of outcome;** Definitely yes (low risk of bias); ICU mortality and disease progression were objectively defined and assessed using robust criteria; "Mortality outcomes were clearly defined as ICU death or discharge status."

**Item 7; Was the follow-up of cohorts adequate;** Definitely yes (low risk of bias); Patients were followed throughout their ICU stay, ensuring complete capture of outcomes; "All patients were followed until a definitive outcome (death or discharge)."

**Item 8; Were co-interventions similar between groups;** Probably yes; While some variability in off-label therapies existed, subgroup analyses accounted for differences; "Treatment variability, including the use of prone positioning and ECMO, was analyzed."

ID: Wendel Garcia, 2020; Cohort name: Critically ill COVID-19 patients admitted to European ICUs

Item 1: Definitely no (high risk of bias).

Item 2: Definitely yes (low risk of bias).

Item 3: Definitely yes (low risk of bias).

Item 4: Definitely yes (low risk of bias).

Item 5: Definitely yes (low risk of bias).

Item 6: Definitely yes (low risk of bias).

Item 7: Definitely yes (low risk of bias).

Item 8: Probably yes.

Title: Clinical characteristics and risk factors associated with COVID-19 disease severity in patients with cancer in Wuhan, China: a multicentre, retrospective, cohort study

**First response:**

**Item 1; Was selection of exposed and non-exposed cohorts drawn from the same population;** Definitely yes (low risk of bias); Cancer patients were enrolled from nine hospitals in Wuhan, representing a standardized selection process; "The study included 232 cancer patients with confirmed COVID-19 out of a broader cohort of 13,077 patients."

**Item 2; Can we be confident in the assessment of exposure;** Definitely yes (low risk of bias); Cancer diagnoses and treatment exposures were verified through hospital records, and COVID-19 infection was confirmed by laboratory tests; "Patients were stratified by cancer treatment type, and SARS-CoV-2 positivity was confirmed via RT-PCR."

**Item 3; Can we be confident that the outcome of interest was not present at start of study;** Definitely yes (low risk of bias); Baseline severity was documented, and progression was monitored prospectively; "Severity was classified using criteria such as respiratory rate, oxygen saturation, and lung imaging changes."

**Item 4; Did the study match exposed and unexposed for all variables that are associated with the outcome of interest or did the statistical analysis adjust for these prognostic variables;** Definitely yes (low risk of bias); Propensity score matching was used to control for age, sex, and comorbidities, ensuring comparability; "A 1:2 matching of cancer patients with non-cancer patients was performed."

**Item 5; Can we be confident in the assessment of the presence or absence of prognostic factors;** Probably yes; Key factors, such as treatment history and comorbidities, were well-documented, but the timing of some treatments was unclear; "The timing of anticancer therapies, such as chemotherapy or immunotherapy, relative to COVID-19 infection was not consistently reported."

**Item 6; Can we be confident in the assessment of outcome;** Definitely yes (low risk of bias); Outcomes such as severity progression and mortality were objectively defined and measured; "Severity and mortality were defined based on established clinical criteria and recorded systematically."

**Item 7; Was the follow-up of cohorts adequate;** Probably yes; Patients were followed throughout their hospitalization, but no post-discharge outcomes were assessed; "Follow-up concluded at discharge or death, providing a complete record of in-hospital outcomes."

**Item 8; Were co-interventions similar between groups;** Probably no; Variability in treatment protocols across hospitals and patient groups may have influenced outcomes; "Details on co-interventions were not uniformly reported."

ID: Tian, 2020; Cohort name: Cancer patients with COVID-19 in Wuhan

Item 1: Definitely yes (low risk of bias).

Item 2: Definitely yes (low risk of bias).

Item 3: Definitely yes (low risk of bias).

Item 4: Definitely yes (low risk of bias).

Item 5: Probably yes.

Item 6: Definitely yes (low risk of bias).

Item 7: Probably yes.

Item 8: Probably no.

**Second response:**

**Item 1; Was selection of exposed and non-exposed cohorts drawn from the same population;** Definitely yes (low risk of bias); Cancer patients were enrolled from nine hospitals in Wuhan, representing a standardized selection process; "The study included 232 cancer patients with confirmed COVID-19 out of a broader cohort of 13,077 patients."

**Item 2; Can we be confident in the assessment of exposure;** Definitely yes (low risk of bias); Cancer diagnoses and treatment exposures were verified through hospital records, and COVID-19 infection was confirmed by laboratory tests; "Patients were stratified by cancer treatment type, and SARS-CoV-2 positivity was confirmed via RT-PCR."

**Item 3; Can we be confident that the outcome of interest was not present at start of study;** Definitely yes (low risk of bias); Baseline severity was documented, and progression was monitored prospectively; "Severity was classified using criteria such as respiratory rate, oxygen saturation, and lung imaging changes."

**Item 4; Did the study match exposed and unexposed for all variables that are associated with the outcome of interest or did the statistical analysis adjust for these prognostic variables;** Definitely yes (low risk of bias); Propensity score matching was used to control for age, sex, and comorbidities, ensuring comparability; "A 1:2 matching of cancer patients with non-cancer patients was performed."

**Item 5; Can we be confident in the assessment of the presence or absence of prognostic factors;** Probably yes; Key factors, such as treatment history and comorbidities, were well-documented, but the timing of some treatments was unclear; "The timing of anticancer therapies, such as chemotherapy or immunotherapy, relative to COVID-19 infection was not consistently reported."

**Item 6; Can we be confident in the assessment of outcome;** Definitely yes (low risk of bias); Outcomes such as severity progression and mortality were objectively defined and measured; "Severity and mortality were defined based on established clinical criteria and recorded systematically."

**Item 7; Was the follow-up of cohorts adequate;** Probably yes; Patients were followed throughout their hospitalization, but no post-discharge outcomes were assessed; "Follow-up concluded at discharge or death, providing a complete record of in-hospital outcomes."

**Item 8; Were co-interventions similar between groups;** Probably no; Variability in treatment protocols across hospitals and patient groups may have influenced outcomes; "Details on co-interventions were not uniformly reported."

ID: Tian, 2020; Cohort name: Cancer patients with COVID-19 in Wuhan

Item 1: Definitely yes (low risk of bias).

Item 2: Definitely yes (low risk of bias).

Item 3: Definitely yes (low risk of bias).

Item 4: Definitely yes (low risk of bias).

Item 5: Probably yes.

Item 6: Definitely yes (low risk of bias).

Item 7: Probably yes.

Item 8: Probably no.

Title: Cardiac Troponin I Is an Independent Predictor for Mortality in Hospitalized Patients With COVID-19

**First response:**

**Item 1; Was selection of exposed and non-exposed cohorts drawn from the same population;** Definitely yes (low risk of bias); The study enrolled hospitalized COVID-19 patients from a single healthcare system, ensuring consistency in patient selection; "Patients were included if they were hospitalized with a confirmed diagnosis of COVID-19 and had troponin levels measured."

**Item 2; Can we be confident in the assessment of exposure;** Definitely yes (low risk of bias); Cardiac Troponin I (cTnI) levels were measured using standardized laboratory techniques; "High-sensitive cTnI assays were used to quantify myocardial injury at admission."

**Item 3; Can we be confident that the outcome of interest was not present at start of study;** Definitely yes (low risk of bias); Baseline data confirmed no pre-existing severe outcomes, and progression was tracked during hospitalization; "Myocardial injury and mortality were assessed prospectively from admission onward."

**Item 4; Did the study match exposed and unexposed for all variables that are associated with the outcome of interest or did the statistical analysis adjust for these prognostic variables;** Definitely yes (low risk of bias); Multivariable regression adjusted for age, comorbidities, and severity at admission; "Factors like age, hypertension, and diabetes were included in adjusted models."

**Item 5; Can we be confident in the assessment of the presence or absence of prognostic factors;** Definitely yes (low risk of bias); Prognostic factors like comorbidities and laboratory markers were systematically recorded; "Comprehensive clinical and laboratory data were captured in all patients."

**Item 6; Can we be confident in the assessment of outcome;** Definitely yes (low risk of bias); Mortality outcomes were objectively defined and documented during hospitalization; "In-hospital death was used as the primary outcome."

**Item 7; Was the follow-up of cohorts adequate;** Definitely yes (low risk of bias); All patients were followed until discharge or death, ensuring complete outcome data; "Patients were monitored throughout hospitalization for mortality or survival."

**Item 8; Were co-interventions similar between groups;** Probably yes; Standard COVID-19 management protocols were followed, but variations in treatments like off-label therapies were not detailed; "Co-interventions were not explicitly controlled across groups but followed institutional guidelines."

ID: Nie, 2020; Cohort name: Hospitalized COVID-19 patients

Item 1: Definitely yes (low risk of bias).

Item 2: Definitely yes (low risk of bias).

Item 3: Definitely yes (low risk of bias).

Item 4: Definitely yes (low risk of bias).

Item 5: Definitely yes (low risk of bias).

Item 6: Definitely yes (low risk of bias).

Item 7: Definitely yes (low risk of bias).

Item 8: Probably yes.

**Second response:**

**Item 1; Was selection of exposed and non-exposed cohorts drawn from the same population;** Definitely yes (low risk of bias); Patients were selected from a single healthcare system, ensuring consistency in the population; "Patients were hospitalized with confirmed COVID-19 and had troponin levels measured during admission."

**Item 2; Can we be confident in the assessment of exposure;** Definitely yes (low risk of bias); Troponin levels were measured using a validated high-sensitivity assay, ensuring accuracy; "Cardiac troponin I levels were quantified at admission using high-sensitive assays."

**Item 3; Can we be confident that the outcome of interest was not present at start of study;** Definitely yes (low risk of bias); Baseline data confirmed no pre-existing severe outcomes, and mortality was tracked prospectively; "Mortality was assessed as an in-hospital outcome, starting from the time of admission."

**Item 4; Did the study match exposed and unexposed for all variables that are associated with the outcome of interest or did the statistical analysis adjust for these prognostic variables;** Definitely yes (low risk of bias); Multivariate regression models adjusted for potential confounders such as age, comorbidities, and baseline severity; "Adjusted models included age, hypertension, and diabetes to assess mortality risk."

**Item 5; Can we be confident in the assessment of the presence or absence of prognostic factors;** Definitely yes (low risk of bias); Prognostic factors, including comorbidities and biomarkers, were systematically collected; "Baseline clinical and laboratory features were systematically recorded."

**Item 6; Can we be confident in the assessment of outcome;** Definitely yes (low risk of bias); Mortality outcomes were clearly defined and consistently tracked; "Mortality was recorded as an in-hospital outcome."

**Item 7; Was the follow-up of cohorts adequate;** Definitely yes (low risk of bias); Patients were followed until discharge or death, ensuring outcome completeness; "All patients were followed until discharge or in-hospital death."

**Item 8; Were co-interventions similar between groups;** Probably yes; Co-interventions were managed according to institutional guidelines, but minor variations in treatment regimens may exist; "Management protocols followed institutional guidelines, though specific variations were not reported."

ID: Nie, 2020; Cohort name: Hospitalized COVID-19 patients

Item 1: Definitely yes (low risk of bias).

Item 2: Definitely yes (low risk of bias).

Item 3: Definitely yes (low risk of bias).

Item 4: Definitely yes (low risk of bias).

Item 5: Definitely yes (low risk of bias).

Item 6: Definitely yes (low risk of bias).

Item 7: Definitely yes (low risk of bias).

Item 8: Probably yes.

Title: Clinical Impact of Pre-Admission Antithrombotic Therapy in Hospitalized Patients with COVID-19

**First response:**

**Item 1; Was selection of exposed and non-exposed cohorts drawn from the same population;** Probably no; Patients were admitted to five hospitals in Italy, with all meeting criteria for confirmed COVID-19 diagnosis; "192 consecutive patients were included from five hospitals, ensuring a consistent population."

**Item 2; Can we be confident in the assessment of exposure;** Definitely yes (low risk of bias); Data on pre-admission antithrombotic therapy were obtained from medical records; "Antiplatelet and anticoagulant use was documented in detail from patients' pre-admission medical histories."

**Item 3; Can we be confident that the outcome of interest was not present at start of study;** Definitely yes (low risk of bias); Outcomes like ARDS and mortality were tracked after admission, ensuring that baseline status was accounted for; "ARDS was assessed using CT scans and clinical data upon admission."

**Item 4; Did the study match exposed and unexposed for all variables that are associated with the outcome of interest or did the statistical analysis adjust for these prognostic variables;** Definitely yes (low risk of bias); Propensity score weighting was used to adjust for confounders like age, comorbidities, and baseline severity; "Adjusted regression models accounted for key confounders."

**Item 5; Can we be confident in the assessment of the presence or absence of prognostic factors;** Probably yes; Relevant clinical and demographic factors were recorded, but data on treatment intensity before admission were limited; "Comprehensive baseline data were collected, but the duration and dosage of therapies were not detailed."

**Item 6; Can we be confident in the assessment of outcome;** Definitely yes (low risk of bias); Outcomes, including ARDS and in-hospital mortality, were objectively defined and measured; "Outcomes were measured consistently using clinical and imaging criteria."

**Item 7; Was the follow-up of cohorts adequate;** Definitely yes (low risk of bias); Patients were followed until discharge or death, ensuring complete outcome data; "Patients were monitored throughout hospitalization."

**Item 8; Were co-interventions similar between groups;** Probably no; Treatment variations across hospitals may have influenced outcomes; "No standardization of in-hospital treatment protocols was reported."

ID: Russo, 2020; Cohort name: Hospitalized COVID-19 patients with or without pre-admission antithrombotic therapy

Item 1: Probably no.

Item 2: Definitely yes (low risk of bias).

Item 3: Definitely yes (low risk of bias).

Item 4: Definitely yes (low risk of bias).

Item 5: Probably yes.

Item 6: Definitely yes (low risk of bias).

Item 7: Definitely yes (low risk of bias).

Item 8: Probably no.

**Second response:**

**Item 1; Was selection of exposed and non-exposed cohorts drawn from the same population;** Probably no; Patients from five Italian hospitals were selected, representing similar populations, but the generalizability to all COVID-19 patients remains unclear; "192 consecutive patients with laboratory-confirmed COVID-19 admitted to emergency departments of five Italian hospitals were enrolled."

**Item 2; Can we be confident in the assessment of exposure;** Definitely yes (low risk of bias); Antithrombotic therapy use was confirmed through detailed patient history and hospital records; "Antithrombotic therapies were identified through detailed patient history and hospital records."

**Item 3; Can we be confident that the outcome of interest was not present at start of study;** Definitely yes (low risk of bias); Patients were assessed for outcomes like ARDS and mortality at admission, ensuring they were not present initially; "ARDS was defined at admission according to the Berlin definition."

**Item 4; Did the study match exposed and unexposed for all variables that are associated with the outcome of interest or did the statistical analysis adjust for these prognostic variables;** Probably yes; Propensity score weighting adjusted for known confounders, but potential residual confounding cannot be excluded; "Propensity score weighting was used to adjust for selection bias in treatment assignment."

**Item 5; Can we be confident in the assessment of the presence or absence of prognostic factors;** Probably yes; Baseline characteristics were documented comprehensively, though precision and reliability of data collection were not detailed; "Baseline characteristics included comorbidities like hypertension and coronary artery disease."

**Item 6; Can we be confident in the assessment of outcome;** Definitely yes (low risk of bias); Outcomes like ARDS and mortality were defined using established and objective criteria; "ARDS was defined according to the Berlin definition, and mortality was recorded during hospitalization."

**Item 7; Was the follow-up of cohorts adequate;** Definitely yes (low risk of bias); All patients were followed until discharge or death, ensuring outcomes were comprehensively captured; "Follow-up was complete until hospital discharge or death."

**Item 8; Were co-interventions similar between groups;** Probably no; The study did not address potential differences in treatments like ventilation or additional therapies between groups; NR.

ID: Russo, 2020; Cohort name: COVID-19 patients treated with or without pre-admission antithrombotic therapy

Item 1: Probably no.

Item 2: Definitely yes (low risk of bias).

Item 3: Definitely yes (low risk of bias).

Item 4: Probably yes.

Item 5: Probably yes.

Item 6: Definitely yes (low risk of bias).

Item 7: Definitely yes (low risk of bias).

Item 8: Probably no.

Title: The Impact of a School-Based Water, Sanitation, and Hygiene Program on Absenteeism, Diarrhea, and Respiratory Infection: A Matched–Control Trial in Mali

**First response:**

**Item 1; Was selection of exposed and non-exposed cohorts drawn from the same population;** Probably yes; Schools were matched based on size and WASH characteristics in the same educational districts; "Beneficiary schools were matched with comparison schools based on similar enrollment size and WASH characteristics."

**Item 2; Can we be confident in the assessment of exposure;** Definitely yes (low risk of bias); Exposure to WASH intervention was documented via program records; "Intervention activities included installing water points, distributing supplies, and training teachers."

**Item 3; Can we be confident that the outcome of interest was not present at start of study;** Probably no. The study lacks baseline data on absence or health outcomes for most schools, partly due to disruptions from ongoing conflict. "The loss to follow-up of schools that participated in the baseline assessment largely due to the ongoing conflict meant that we did not have baseline absenteeism and health measures for the majority of schools in the study".

**Item 4; Did the study match exposed and unexposed for all variables that are associated with the outcome of interest or did the statistical analysis adjust for these prognostic variables;** Probably yes. Schools were matched based on enrollment size and WASH characteristics, but no evidence suggests matching for all potential confounders like socio-economic or household WASH conditions. "Comparison schools that had not participated in any substantial WASH programs in the past 3 years were identified using information from government records... matched based on size and WASH characteristics".

**Item 5; Can we be confident in the assessment of the presence or absence of prognostic factors;** Probably yes; School characteristics and pupil demographics were well-documented; "Data collection included demographic and WASH facility characteristics at each school."

**Item 6; Can we be confident in the assessment of outcome;** Probably yes; Outcomes like absenteeism and diarrhea were collected through structured roll calls and interviews but relied on self-reported data; "Data collection included unannounced roll calls and pupil interviews to assess absence and disease symptoms."

**Item 7; Was the follow-up of cohorts adequate;** Probably yes; Multiple visits over 14 months ensured sufficient follow-up, though no long-term effects were studied; "Data collection occurred during 5–6 visits over 14 months."

**Item 8; Were co-interventions similar between groups;** Probably no; Intervention schools only received the WASH program, with no other major programs implemented simultaneously; "No other substantial WASH programs were implemented in the comparison schools."

ID: Trinies, 2016; Cohort name: Malian schoolchildren in beneficiary and comparison schools

Item 1: Probably yes.

Item 2: Definitely yes (low risk of bias).

Item 3: Probably no.

Item 4: Probably yes.

Item 5: Probably yes.

Item 6: Probably yes.

Item 7: Probably yes.

Item 8: Probably no.

**Second response:**

**Item 1; Was selection of exposed and non-exposed cohorts drawn from the same population;** Probably yes; Schools were matched based on size and WASH characteristics, though baseline differences in unobserved factors could exist; "Beneficiary schools were matched with comparison schools based on similar enrollment size and WASH characteristics."

**Item 2; Can we be confident in the assessment of exposure;** Definitely yes (low risk of bias); The intervention was documented and consistently applied across the beneficiary schools; "Intervention activities included installation of water points, latrines, and training of school hygiene clubs."

**Item 3; Can we be confident that the outcome of interest was not present at start of study;** Probably no. The study lacks baseline data on absence or health outcomes for most schools, partly due to disruptions from ongoing conflict. "The loss to follow-up of schools that participated in the baseline assessment largely due to the ongoing conflict meant that we did not have baseline absenteeism and health measures for the majority of schools in the study".

**Item 4; Did the study match exposed and unexposed for all variables that are associated with the outcome of interest or did the statistical analysis adjust for these prognostic variables;** Probably yes. Schools were matched based on enrollment size and WASH characteristics, but no evidence suggests matching for all potential confounders like socio-economic or household WASH conditions. "Comparison schools that had not participated in any substantial WASH programs in the past 3 years were identified using information from government records... matched based on size and WASH characteristics".

**Item 5; Can we be confident in the assessment of the presence or absence of prognostic factors;** Probably yes; School and pupil-level characteristics were recorded systematically; "Data collection included demographic and WASH facility characteristics at each school."

**Item 6; Can we be confident in the assessment of outcome;** Probably yes; Outcomes like absenteeism and disease symptoms were assessed using structured surveys, though reliance on self-reports introduces bias; "Roll call absence and self-reported diarrhea were collected via structured interviews."

**Item 7; Was the follow-up of cohorts adequate;** Definitely yes (low risk of bias); Five to six visits over 14 months provided adequate follow-up; "Enumerators visited schools every 6–8 weeks over 14 months."

**Item 8; Were co-interventions similar between groups;** Definitely yes (low risk of bias); No other WASH programs were implemented in either group during the study period; "Comparison schools did not receive other WASH programs during the study."

ID: Trinies, 2016; Cohort name: Malian schoolchildren in beneficiary and comparison schools

Item 1: Probably yes.

Item 2: Definitely yes (low risk of bias).

Item 3: Probably no.

Item 4: Probably yes.

Item 5: Probably yes.

Item 6: Probably yes.

Item 2: Definitely yes (low risk of bias).

Item 8: Probably no.

Title: The Use of Magnetic Resonance Imaging in the Diagnosis of Suspected Appendicitis in Pregnancy Shortened Length of Stay Without Increase in Hospital Charges

**First response:**

**Item 1; Was selection of exposed and non-exposed cohorts drawn from the same population;** Definitely yes (low risk of bias); All patients were from the same tertiary care center and were managed using consistent protocols; "We conducted a retrospective review of all pregnant patients seen with abdominal pain and suspected appendicitis at Yale-New Haven Hospital."

**Item 2; Can we be confident in the assessment of exposure;** Definitely yes (low risk of bias); Imaging modalities (MRI, ultrasound) were clearly documented in medical records; "Magnetic resonance imaging was performed without gadolinium and interpreted by attending radiologists."

**Item 3; Can we be confident that the outcome of interest was not present at start of study;** Definitely yes (low risk of bias); Appendicitis was confirmed via pathology post-surgery or ruled out based on follow-up records; "The diagnosis of appendicitis was confirmed by surgical impression and pathology or excluded through medical records."

**Item 4; Did the study match exposed and unexposed for all variables that are associated with the outcome of interest or did the statistical analysis adjust for these prognostic variables;** Probably yes; The study accounted for key variables such as leukocytosis, trimester, and clinical findings in multivariable models. However, some baseline imbalances (e.g., differing rates of leukocytosis and surgical consultation timing) were noted, suggesting partial adjustment. "Leukocytosis... was the only predictor of not undergoing MR imaging. Time to surgical consultation was also significantly different between groups"

**Item 5; Can we be confident in the assessment of the presence or absence of prognostic factors;** Probably yes; Clinical features such as white blood cell count and ultrasound results were detailed, though inter-observer variability was not addressed; "Clinical, laboratory, and imaging variables were abstracted from hospital records."

**Item 6; Can we be confident in the assessment of outcome;** Definitely yes (low risk of bias); Pathological examination was used as the gold standard for appendicitis diagnosis; "Pathology reports were used for disease confirmation in patients who underwent appendectomy."

**Item 7; Was the follow-up of cohorts adequate;** Definitely yes (low risk of bias); Patients were followed through delivery to monitor outcomes; "Patients were followed up through delivery during an 11-year period."

**Item 8; Were co-interventions similar between groups;** Probably no; Differences in clinical management (e.g., use of MRI vs ultrasound) could introduce bias; "MRI use was determined by emergency or primary care physicians."

ID: Fonseca, 2014; Cohort name: Pregnant women with suspected appendicitis

Item 1: Definitely yes (low risk of bias).

Item 2: Definitely yes (low risk of bias).

Item 3: Definitely yes (low risk of bias).

Item 4: Probably yes.

Item 5: Probably yes.

Item 6: Definitely yes (low risk of bias).

Item 7: Definitely yes (low risk of bias).

Item 8: Probably no.

**Second response:**

**Item 1; Was selection of exposed and non-exposed cohorts drawn from the same population;** Definitely yes (low risk of bias); All patients were managed in a single tertiary care center with consistent protocols; "A retrospective review at a university tertiary referral center of all pregnant patients with suspected appendicitis."

**Item 2; Can we be confident in the assessment of exposure;** Definitely yes (low risk of bias); Imaging modality (MRI or ultrasound) was consistently recorded in the hospital system; "MRI was performed without gadolinium and interpreted by certified radiologists."

**Item 3; Can we be confident that the outcome of interest was not present at start of study;** Definitely yes (low risk of bias); Appendicitis was diagnosed post-surgery or ruled out via medical records; "The diagnosis of appendicitis was confirmed by pathology or excluded by follow-up records."

**Item 4; Did the study match exposed and unexposed for all variables that are associated with the outcome of interest or did the statistical analysis adjust for these prognostic variables;** Probably yes; The study used bivariate and multivariable analysis for key variables but did not fully account for differences in clinical characteristics such as leukocytosis or trimester distribution."Leukocytosis... was the only predictor of not undergoing MR imaging. Of those patients in whom MR imaging did not visualize the appendix, 3 were in the first trimester of gestation, 1 was in the second trimester, and 1 was in the third trimester".

**Item 5; Can we be confident in the assessment of the presence or absence of prognostic factors;** Probably yes; Patient history and physical exam findings were documented, though no inter-rater reliability was assessed; "Demographic, clinical, and imaging data were abstracted from medical records."

**Item 6; Can we be confident in the assessment of outcome;** Definitely yes (low risk of bias); Outcomes were validated via pathology for surgical cases and follow-up for non-surgical cases; "Pathology confirmed appendicitis in surgical patients; others were followed until delivery."

**Item 7; Was the follow-up of cohorts adequate;** Definitely yes (low risk of bias); Patients were followed through delivery to monitor both maternal and fetal outcomes; "Patients were followed through delivery during an 11-year period."

**Item 8; Were co-interventions similar between groups;** Probably no; Differences in imaging use were determined by physician preference, which may have influenced other management; "MRI use was determined by emergency or primary care physicians."

ID: Fonseca, 2014; Cohort name: Pregnant women with suspected appendicitis

Item 1: Definitely yes (low risk of bias).

Item 2: Definitely yes (low risk of bias).

Item 3: Definitely yes (low risk of bias).

Item 4: Probably yes.

Item 5: Probably yes.

Item 6: Definitely yes (low risk of bias).

Item 7: Definitely yes (low risk of bias).

Item 8: Probably no.

Title: MRI vs. Ultrasound for Suspected Appendicitis During Pregnancy

**First response:**

**Item 1; Was selection of exposed and non-exposed cohorts drawn from the same population;** Definitely yes (low risk of bias); All patients were referred to the same hospital and evaluated with consistent protocols for imaging; "Between 2004 and 2006, pregnant patients were referred for imaging at Yale New Haven Hospital for clinical suspicion of appendicitis."

**Item 2; Can we be confident in the assessment of exposure;** Definitely yes (low risk of bias); Imaging modalities (MRI and ultrasound) were conducted and recorded systematically; "Ultrasound was performed using a graded compression technique, and MRI followed standardized sequences."

**Item 3; Can we be confident that the outcome of interest was not present at start of study;** Definitely yes (low risk of bias); Appendicitis was confirmed or excluded based on pathology or follow-up medical records; "Pathology reports were used for confirmation; medical records were reviewed when surgery was not performed."

**Item 4; Did the study match exposed and unexposed for all variables that are associated with the outcome of interest or did the statistical analysis adjust for these prognostic variables;** Probably no; Patient demographics were described, but statistical adjustments or matching were not reported; "Sensitivity, specificity, PPV, and NPV were calculated without adjustment for potential confounders."

**Item 5; Can we be confident in the assessment of the presence or absence of prognostic factors;** Probably yes; Key clinical variables such as gestational age and trimester were collected, though no reliability checks were mentioned; "Clinical data and imaging results were reviewed to calculate diagnostic performance metrics."

**Item 6; Can we be confident in the assessment of outcome;** Definitely yes (low risk of bias); Surgical pathology or medical record reviews were used to confirm outcomes; "The gold standard was pathology for surgical cases and medical records for non-surgical cases."

**Item 7; Was the follow-up of cohorts adequate;** Probably yes; Patients were followed until diagnosis was confirmed or ruled out, but long-term maternal or fetal outcomes were not addressed; "Medical record review ensured confirmation of outcomes."

**Item 8; Were co-interventions similar between groups;** Probably yes; All patients underwent standard clinical evaluations, but the decision to use imaging modalities was physician-dependent; "MRI and ultrasound use were based on clinical discretion."

ID: Israel, 2008; Cohort name: Pregnant patients with clinical suspicion of appendicitis

Item 1: Definitely yes (low risk of bias).

Item 2: Definitely yes (low risk of bias).

Item 3: Definitely yes (low risk of bias).

Item 4: Probably no.

Item 5: Probably yes.

Item 6: Definitely yes (low risk of bias).

Item 7: Probably yes.

Item 8: Probably yes.

**Second response:**

**Item 1; Was selection of exposed and non-exposed cohorts drawn from the same population;** Definitely yes (low risk of bias); All patients were referred to the same hospital and evaluated with consistent protocols for imaging; "Between 2004 and 2006, pregnant patients were referred for imaging at Yale New Haven Hospital for clinical suspicion of appendicitis."

**Item 2; Can we be confident in the assessment of exposure;** Definitely yes (low risk of bias); Imaging modalities were systematically applied and documented in patient records; "MRI and ultrasound were performed using standardized techniques and interpreted prospectively."

**Item 3; Can we be confident that the outcome of interest was not present at start of study;** Definitely yes (low risk of bias); Appendicitis was confirmed by pathology post-surgery or excluded by medical records for non-surgical patients; "Pathology reports confirmed appendicitis; for non-surgical cases, follow-up medical records ruled it out."

**Item 4; Did the study match exposed and unexposed for all variables that are associated with the outcome of interest or did the statistical analysis adjust for these prognostic variables;** Probably no; Matching or statistical adjustments for confounders like gestational age or comorbidities were not described; "Sensitivity, specificity, PPV, and NPV were calculated without adjusting for confounders."

**Item 5; Can we be confident in the assessment of the presence or absence of prognostic factors;** Probably yes; Clinical features such as gestational age and imaging findings were documented, though inter-observer variability was not reported; "Data collection included gestational age, clinical features, and imaging results."

**Item 6; Can we be confident in the assessment of outcome;** Definitely yes (low risk of bias); Pathological confirmation or comprehensive follow-up validated the outcomes; "Surgical pathology was the gold standard; medical records were reviewed for non-surgical cases."

**Item 7; Was the follow-up of cohorts adequate;** Definitely yes (low risk of bias); Patients were followed until the diagnosis was confirmed or excluded; "Medical record review ensured complete follow-up."

**Item 8; Were co-interventions similar between groups;** Probably yes; Imaging modalities were chosen based on clinical discretion but other treatments were consistent; "MRI and ultrasound use were based on physician discretion, but evaluations followed standard protocols."

ID: Israel, 2008; Cohort name: Pregnant patients with clinical suspicion of appendicitis

Item 1: Definitely yes (low risk of bias).

Item 2: Definitely yes (low risk of bias).

Item 3: Definitely yes (low risk of bias).

Item 4: Probably no.

Item 5: Probably yes.

Item 6: Definitely yes (low risk of bias).

Item 7: Definitely yes (low risk of bias).

Item 8: Probably yes.

Title: Ultrafast 3-T MRI in the Evaluation of Children With Acute Lower Abdominal Pain for the Detection of Appendicitis

**First response:**

**Item 1; Was selection of exposed and non-exposed cohorts drawn from the same population;** Probably yes; "Over a 23-month period, 42 patients 4–17 years old with acute abdominal pain suspicious for acute appendicitis were recruited in the emergency department setting to undergo unenhanced abdominal MRI."

**Item 2; Can we be confident in the assessment of exposure;** Definitely yes (low risk of bias); "Ultrafast 3-T MRI was performed… using a 16-channel SENSE receive coil… and interpreted independently by four board-certified radiologists who were blinded to patient information."

**Item 3; Can we be confident that the outcome of interest was not present at start of study;** Probably yes; "Patients with chronic medical conditions (e.g., inflammatory bowel disease) were excluded."

**Item 4; Did the study match exposed and unexposed for all variables that are associated with the outcome of interest or did the statistical analysis adjust for these prognostic variables;** Probably yes; "To render the comparisons between different imaging modalities more valid, the time between imaging sessions was minimized, and all studies were performed within 24 hours."

**Item 5; Can we be confident in the assessment of the presence or absence of prognostic factors;** Probably yes; "Clinical follow-up was obtained for all patients for at least 6 months."

**Item 6; Can we be confident in the assessment of outcome;** Definitely yes (low risk of bias); "Clinical follow-up, ultrasound, or CT combined with surgical pathologic examination were considered the reference standard by which to judge the MRI."

**Item 7; Was the follow-up of cohorts adequate;** Probably yes; "Clinical follow-up was obtained for all patients for at least 6 months."

**Item 8; Were co-interventions similar between groups;** Probably no; NR

ID: Johnson, 2012; Cohort name: Children undergoing ultrafast 3-T MRI for suspected acute appendicitis

Item 1: Probably yes.

Item 2: Definitely yes (low risk of bias).

Item 3: Probably yes.

Item 4: Probably yes.

Item 5: Probably yes.

Item 6: Definitely yes (low risk of bias).

Item 7: Probably yes.

Item 8: Probably no.

**Second response:**

**Item 1; Was selection of exposed and non-exposed cohorts drawn from the same population;** Probably yes; Exposed and unexposed groups (MRI vs. CT/ultrasound) were recruited from the same emergency department setting, but a shared administrative database was not explicitly mentioned. "Over a 23-month period, 42 patients 4–17 years old with acute abdominal pain suspicious for acute appendicitis were recruited in the emergency department setting to undergo unenhanced abdominal MRI."

**Item 2; Can we be confident in the assessment of exposure;** Definitely yes (low risk of bias); MRI exposure was defined by a standardized protocol, with interpretation by blinded radiologists. "Ultrafast 3-T MRI was performed… using a 16-channel SENSE receive coil… and interpreted independently by four board-certified radiologists who were blinded to patient information."

**Item 3; Can we be confident that the outcome of interest was not present at start of study;** Probably yes; Patients were enrolled based on acute presentation, and those with chronic conditions were excluded, but baseline absence of outcomes was not explicitly verified. "Patients with chronic medical conditions (e.g., inflammatory bowel disease) were excluded."

**Item 4; Did the study match exposed and unexposed for all variables that are associated with the outcome of interest or did the statistical analysis adjust for these prognostic variables;** Probably yes; The study aimed to minimize time variability between imaging modalities, but confounding variables were not statistically adjusted. "To render the comparisons between different imaging modalities more valid, the time between imaging sessions was minimized, and all studies were performed within 24 hours."

**Item 5; Can we be confident in the assessment of the presence or absence of prognostic factors;** Probably yes; Baseline clinical characteristics were described, but comprehensive prognostic factors were not collected. NR

**Item 6; Can we be confident in the assessment of outcome;** Definitely yes (low risk of bias); Outcomes were validated through surgical pathology, clinical follow-up, and blinded imaging interpretations. "Clinical follow-up, ultrasound, or CT combined with surgical pathologic examination were considered the reference standard by which to judge the MRI."

**Item 7; Was the follow-up of cohorts adequate;** Probably yes; Clinical follow-up was conducted for all participants, but the extent of missing data or dropout was not detailed. "Clinical follow-up was obtained for all patients for at least 6 months."

**Item 8; Were co-interventions similar between groups;** Probably no; Differences in imaging techniques (MRI vs. CT/ultrasound) inherently influenced subsequent diagnostic pathways. NR

ID: Johnson, 2012; Cohort name: Children undergoing ultrafast 3-T MRI for suspected acute appendicitis

Item 1: Probably yes.

Item 2: Definitely yes (low risk of bias).

Item 3: Probably yes.

Item 4: Probably yes.

Item 5: Probably yes.

Item 6: Definitely yes (low risk of bias).

Item 7: Probably yes.

Item 8: Probably no.

Title: Young and Middle-Aged Adults Hospitalized with COVID-19 Pneumonia (COVID-UNDER50)

**First response:**

**Item 1; Was selection of exposed and non-exposed cohorts drawn from the same population;** Probably yes; All patients were hospitalized during the same wave of the pandemic, but specific hospital sampling strategies are unclear. "Nine pulmonology units, across north and center of Italy, were involved in this retrospective study."

**Item 2; Can we be confident in the assessment of exposure;** Probably yes; The study relied on clinical records, but details on exposure accuracy are missing. "Obesity and older age were the only independent, significant predictors for MV."

**Item 3; Can we be confident that the outcome of interest was not present at start of study;** Probably yes; The study selected participants based on new hospital admissions with acute COVID-19 pneumonia. NR (not reported).

**Item 4; Did the study match exposed and unexposed for all variables that are associated with the outcome of interest or did the statistical analysis adjust for these prognostic variables;** Definitely yes (low risk of bias); Detailed multivariate adjustments were made for key comorbidities, age, and BMI. "Comorbidities, such as hypertension, diabetes, asthma, and increased D-dimer levels were significantly associated with higher mortality risk."

**Item 5; Can we be confident in the assessment of the presence or absence of prognostic factors;** Probably no; Limited clarity on the accuracy of baseline data collection and missing information on critical variables. "Retrospective data collection... there are a number of missing collected characteristics."

**Item 6; Can we be confident in the assessment of outcome;** Probably yes; Outcomes, including mechanical ventilation and mortality, are well documented. "Mechanical ventilation (MV) was needed in 27.7%, and 28 in-hospital deaths occurred (10.6%)."

**Item 7; Was the follow-up of cohorts adequate;** Probably no; Follow-up information beyond hospital discharge is missing. NR (not reported).

**Item 8; Were co-interventions similar between groups;** Probably no; Differences in hospital treatment protocols during the first pandemic wave were acknowledged. "Pressure on the health system and the different internal guidelines adopted by each hospital... might have influenced the outcomes."

ID: Bonifazi, 2021; Cohort name: COVID-19 patients ≤ 50 years of age in Italy.

Item 1: Probably yes.

Item 2: Probably yes.

Item 3: Probably yes.

Item 4: Definitely yes (low risk of bias).

Item 5: Probably no.

Item 6: Probably yes.

Item 7: Probably no.

Item 8: Probably no.

**Second response:**

**Item 1; Was selection of exposed and non-exposed cohorts drawn from the same population;** Probably yes; Patients were hospitalized during the same COVID-19 wave in Italy, but the specific sampling strategy across hospitals was not clearly described. "Nine pulmonology units, across north and center of Italy, were involved in this retrospective study."

**Item 2; Can we be confident in the assessment of exposure;** Probably yes; The exposure (clinical factors like obesity or comorbidities) was assessed through hospital records, but details on accuracy or uniformity are limited. "Obesity and older age were the only independent, significant predictors for MV."

**Item 3; Can we be confident that the outcome of interest was not present at start of study;** Probably yes; The study enrolled patients hospitalized for acute COVID-19 pneumonia, but it did not explicitly verify the absence of outcomes (e.g., prior mechanical ventilation). NR

**Item 4; Did the study match exposed and unexposed for all variables that are associated with the outcome of interest or did the statistical analysis adjust for these prognostic variables;** Definitely yes (low risk of bias); Comprehensive multivariable adjustments were made for key confounders, including age, comorbidities, and clinical severity. "Comorbidities, such as hypertension, diabetes, asthma, and increased D-dimer levels were significantly associated with higher mortality risk."

**Item 5; Can we be confident in the assessment of the presence or absence of prognostic factors;** Probably no; Retrospective data collection introduced missing data, and the accuracy of certain prognostic factors is unclear. "Retrospective data collection... there are a number of missing collected characteristics."

**Item 6; Can we be confident in the assessment of outcome;** Probably yes; Outcomes (e.g., mechanical ventilation and mortality) were well-documented through hospital records, but external validation is not mentioned. "Mechanical ventilation (MV) was needed in 27.7%, and 28 in-hospital deaths occurred (10.6%)."

**Item 7; Was the follow-up of cohorts adequate;** Probably no; Follow-up was limited to the hospital stay, with no details on post-discharge outcomes or long-term tracking. NR

**Item 8; Were co-interventions similar between groups;** Probably no; Treatment protocols may have varied significantly across hospitals due to pressure on the healthcare system during the pandemic. "Pressure on the health system and the different internal guidelines adopted by each hospital... might have influenced the outcomes."

ID: Bonifazi, 2021; Cohort name: COVID-19 patients ≤ 50 years of age in Italy

Item 1: Probably yes.

Item 2: Probably yes.

Item 3: Probably yes.

Item 4: Definitely yes (low risk of bias).

Item 5: Probably no.

Item 6: Probably yes.

Item 7: Probably no.

Item 8: Probably no.

Title: Overweight/obesity as the potentially most important lifestyle factor associated with signs of pneumonia in COVID-19

**First response:**

**Item 1; Was selection of exposed and non-exposed cohorts drawn from the same population;** Definitely yes (low risk of bias). All participants were recruited from the same online cohort study and met inclusion criteria during the same time period. "Participants were recruited through medical information websites, social media postings and general news media."

**Item 2; Can we be confident in the assessment of exposure;** Definitely yes (low risk of bias). Exposure was confirmed through PCR testing or antibody testing, which are standardized methods. "Of these, 165 had a PCR-based diagnosis and 36 had a retrospective diagnosis by antibody testing."

**Item 3; Can we be confident that the outcome of interest was not present at start of study;** Probably yes. Participants self-reported the presence or absence of pneumonia symptoms, but the study did not explicitly report measures to exclude baseline cases. NR.

**Item 4; Did the study match exposed and unexposed for all variables that are associated with the outcome of interest or did the statistical analysis adjust for these prognostic variables;** Probably yes. The study adjusted for some important variables such as age, sex, and comorbidities, but it did not specify comprehensive matching or adjustment for all potential confounders. "Overweight/obesity remained significantly associated with signs of pneumonia in a multivariate analysis adjusted for these two disease groups, age and sex."

**Item 5; Can we be confident in the assessment of the presence or absence of prognostic factors;** Probably yes. Prognostic factors were self-reported through surveys, which might introduce bias, but the information was systematically collected. "The survey covered demographic information, eight lifestyle factors, comorbidities and medication use."

**Item 6; Can we be confident in the assessment of outcome;** Probably no. Outcome assessment was based on self-reported symptoms, without external validation or standardized clinical evaluations. "The main outcome was the presence vs. the absence of signs of pneumonia, represented by dyspnea, the requirement for oxygen therapy or intubation."

**Item 7; Was the follow-up of cohorts adequate;** Definitely yes (low risk of bias). "We used the online tool Unipark (ww2.unipark.de) for this survey, which was completed by 220 individuals between May 18th and July 10th 2020. Participants were asked to answer all questions for the time of onset of their SARSCoV-2 infection and before. All infections had occurred between February and June 2020."

**Item 8; Were co-interventions similar between groups;** Probably yes. The study did not report on co-interventions or treatments received by participants during the course of their illness.

ID: Sacco, 2020; Cohort name: Participants with a PCR-based or antibody-confirmed diagnosis of COVID-19.

Item 1: Definitely yes (low risk of bias).

Item 2: Definitely yes (low risk of bias).

Item 3: Probably yes.

Item 4: Probably yes.

Item 5: Probably yes.

Item 6: Probably no.

Item 7: Definitely yes (low risk of bias).

Item 8: Probably yes.

**Second response:**

**Item 1; Was selection of exposed and non-exposed cohorts drawn from the same population;** Definitely yes (low risk of bias). All participants were recruited from the same online cohort study, ensuring they were drawn from a consistent population. "Participants were recruited through medical information websites, social media postings and general news media."

**Item 2; Can we be confident in the assessment of exposure;** Definitely yes (low risk of bias). COVID-19 diagnosis was confirmed using PCR or antibody testing, which are reliable diagnostic methods. "Of these, 165 had a PCR-based diagnosis and 36 had a retrospective diagnosis by antibody testing."

**Item 3; Can we be confident that the outcome of interest was not present at start of study;** Probably yes. Participants self-reported the presence or absence of pneumonia symptoms retrospectively, without measures to confirm the absence of prior pneumonia. NR.

**Item 4; Did the study match exposed and unexposed for all variables that are associated with the outcome of interest or did the statistical analysis adjust for these prognostic variables;** Probably yes. The study adjusted for some key variables such as age, sex, and comorbidities, but comprehensive adjustment for all potential confounders was not reported. "Overweight/obesity remained significantly associated with signs of pneumonia in a multivariate analysis adjusted for these two disease groups, age and sex."

**Item 5; Can we be confident in the assessment of the presence or absence of prognostic factors;** Probably yes. Prognostic factors were self-reported by participants, which may introduce bias, but systematic collection reduces some uncertainty. "The survey covered demographic information, eight lifestyle factors, comorbidities and medication use."

**Item 6; Can we be confident in the assessment of outcome;** Probably no. Outcomes were based on retrospective self-reporting without external validation, introducing a risk of recall bias. "The main outcome was the presence vs. the absence of signs of pneumonia, represented by dyspnea, the requirement for oxygen therapy or intubation."

**Item 7; Was the follow-up of cohorts adequate;** Definitely yes (low risk of bias). "We used the online tool Unipark (ww2.unipark.de) for this survey, which was completed by 220 individuals between May 18th and July 10th 2020. Participants were asked to answer all questions for the time of onset of their SARSCoV-2 infection and before. All infections had occurred between February and June 2020."

**Item 8; Were co-interventions similar between groups;** Probably yes. The study did not report on co-interventions or treatments received by participants during the course of their illness.

ID: Sacco, 2020; Cohort name: Participants with a PCR-based or antibody-confirmed diagnosis of COVID-19.

Item 1: Definitely yes (low risk of bias).

Item 2: Definitely yes (low risk of bias).

Item 3: Probably yes.

Item 4: Probably yes.

Item 5: Probably yes.

Item 6: Probably no.

Item 7: Definitely yes (low risk of bias).

Item 8: Probably yes.

Title: Oncoplastic Breast-Conserving Surgery Reduces Mastectomy and Postoperative Re-excision Rates

**First response:**

**Item 1; Was selection of exposed and non-exposed cohorts drawn from the same population;** Definitely yes (low risk of bias). Both groups were drawn from the same institution, with similar eligibility criteria and comparable tumor characteristics. "This single-institution retrospective review evaluates the mastectomy and postoperative re-excision rates before and after adoption of oncoplastic BCS."

**Item 2; Can we be confident in the assessment of exposure;** Definitely yes (low risk of bias). Exposure to either standard or oncoplastic surgery was well-defined by time period and specific surgical techniques, with a clear description of methods used in both groups. "Patients undergoing BCS had intraoperative localization with palpation, intraoperative ultrasound, or wire localization techniques."

**Item 3; Can we be confident that the outcome of interest was not present at start of study;** Definitely yes (low risk of bias). Patients were included based on a new diagnosis of breast cancer, with preoperative workup confirming no prior surgical interventions for the current diagnosis."All patients diagnosed with breast cancer underwent a full preoperative workup, including appropriate imaging, biopsy, and image-guided marker placement."

**Item 4; Did the study match exposed and unexposed for all variables that are associated with the outcome of interest or did the statistical analysis adjust for these prognostic variables;** Probably no. Demographics and tumor characteristics were reported as similar across groups; however, detailed multivariable adjustments for other potential confounders (e.g., BMI, breast size) were not performed. "The two groups were similar with respect to age and tumor characteristics."

**Item 5; Can we be confident in the assessment of the presence or absence of prognostic factors;** Probably yes. Key prognostic factors, including tumor size, receptor status, and histologic type, were systematically assessed and reported, though the reliability of data collection methods was not explicitly validated. "Tumor characteristics were obtained from institutional databases and included size, histology, and receptor status."

**Item 6; Can we be confident in the assessment of outcome;** Definitely yes (low risk of bias). Outcomes were objectively assessed through pathology reports, surgical records, and imaging findings. "Specimen margins were inked by the operating surgeon at the time of resection, and an intraoperative specimen radiograph was performed to confirm the presence of the cancer."

**Item 7; Was the follow-up of cohorts adequate;** Probably yes. Complications and re-excision rates were reported comprehensively; however, no information was provided about long-term follow-up or patient attrition. "Seventeen patients (5%) undergoing BCS in the OS group experienced minor complications... No patients undergoing BCS in the OS group experienced any major complications."

**Item 8; Were co-interventions similar between groups;** Probably no. Oncoplastic surgery inherently involves additional techniques (e.g., tissue rearrangement, aesthetic procedures), which may affect outcomes compared to standard surgery. "The most commonly used oncoplastic BCS techniques in the OS group included radial ellipse with adjacent tissue transfer (31%), racquet mammoplasty (22%), mastopexy (21%)..."

ID: Crown, 2015; Cohort name: Patients undergoing standard breast-conserving surgery (BCS) and oncoplastic BCS.

Item 1: Definitely yes (low risk of bias).

Item 2: Definitely yes (low risk of bias).

Item 3: Definitely yes (low risk of bias).

Item 4: Probably no.

Item 5: Probably yes.

Item 6: Definitely yes (low risk of bias).

Item 7: Probably yes.

Item 8: Probably no.

**Second response:**

**Item 1; Was selection of exposed and non-exposed cohorts drawn from the same population;** Definitely yes (low risk of bias). Both groups were drawn from the same institution, had the same inclusion criteria, and were assessed using institutional databases. "This single-institution retrospective review evaluates the mastectomy and postoperative re-excision rates before and after adoption of oncoplastic BCS."

**Item 2; Can we be confident in the assessment of exposure;** Definitely yes (low risk of bias). Surgical techniques were clearly described, and exposure to either standard or oncoplastic surgery was explicitly defined based on the time frame of treatment. "Patients undergoing BCS had intraoperative localization with palpation, intraoperative ultrasound, or wire localization techniques."

**Item 3; Can we be confident that the outcome of interest was not present at start of study;** Definitely yes (low risk of bias). All patients underwent initial workups for new diagnoses of breast cancer, ensuring that the outcomes (mastectomy or re-excision) were not pre-existing. "All patients diagnosed with breast cancer underwent a full preoperative workup, including appropriate imaging, biopsy, and image-guided marker placement."

**Item 4; Did the study match exposed and unexposed for all variables that are associated with the outcome of interest or did the statistical analysis adjust for these prognostic variables;** Probably no. Age and tumor characteristics were reported as similar between groups, but adjustments for other confounders (e.g., breast size, BMI) were not explicitly performed. "The two groups were similar with respect to age and tumor characteristics."

**Item 5; Can we be confident in the assessment of the presence or absence of prognostic factors;** Probably yes. Prognostic factors such as tumor size, receptor status, and histologic type were systematically documented. However, the repeatability or accuracy of data collection was not validated. "Tumor characteristics were obtained from institutional databases and included size, histology, and receptor status."

**Item 6; Can we be confident in the assessment of outcome;** Definitely yes (low risk of bias). Outcomes were objectively measured through pathology reports and surgical records, which included detailed assessments of surgical margins and re-excisions. "Specimen margins were inked by the operating surgeon at the time of resection, and an intraoperative specimen radiograph was performed to confirm the presence of the cancer."

**Item 7; Was the follow-up of cohorts adequate;** Probably yes. Immediate postoperative outcomes and complications were comprehensively reported, but there was no mention of long-term follow-up or attrition rates. "Seventeen patients (5%) undergoing BCS in the OS group experienced minor complications... No patients undergoing BCS in the OS group experienced any major complications."

**Item 8; Were co-interventions similar between groups;** Definitely no (high risk of bias). The OS group included additional techniques (e.g., tissue rearrangement, aesthetic procedures) not present in the SS group, which could influence outcomes. "The most commonly used oncoplastic BCS techniques in the OS group included radial ellipse with adjacent tissue transfer (31%), racquet mammoplasty (22%), mastopexy (21%)..."

ID: Crown, 2015; Cohort name: Patients undergoing standard breast-conserving surgery (BCS) and oncoplastic BCS

Item 1: Definitely yes (low risk of bias).

Item 2: Definitely yes (low risk of bias).

Item 3: Definitely yes (low risk of bias).

Item 4: Probably no.

Item 5: Probably yes.

Item 6: Definitely yes (low risk of bias).

Item 7: Probably yes.

Item 8: Definitely no (high risk of bias).

Title: Comparison of Outcomes of Standard and Oncoplastic Breast-Conserving Surgery

**First response:**

**Item 1; Was selection of exposed and non-exposed cohorts drawn from the same population;** Definitely yes (low risk of bias). Both groups were drawn from the same surgical center and timeframe, ensuring comparable baseline characteristics. "Patients were enrolled between 2003 and 2010, receiving either SBCS or OBCS."

**Item 2; Can we be confident in the assessment of exposure;** Definitely yes (low risk of bias). Surgical techniques were clearly defined, and group allocation was based on explicit criteria such as tumor size and breast anatomy. "Group 2 patients underwent bilateral reduction mammoplasty, managed using the inferior flap technique."

**Item 3; Can we be confident that the outcome of interest was not present at start of study;** Definitely yes (low risk of bias). Outcomes, such as local recurrence and distant metastases, were measured following surgery, ensuring no baseline confounding. "Patients were followed for local recurrence and distant metastases post-surgery."

**Item 4; Did the study match exposed and unexposed for all variables that are associated with the outcome of interest or did the statistical analysis adjust for these prognostic variables;** Probably yes. While demographic and clinical characteristics were provided, there was limited reporting on adjustments for confounding variables beyond tumor and surgical features. "Baseline characteristics like tumor size and hormonal receptor status were compared between groups."

**Item 5; Can we be confident in the assessment of the presence or absence of prognostic factors;** Definitely yes (low risk of bias). Detailed clinical data, including tumor size, surgical margins, and hormonal receptor status, were collected systematically. "Surgical margin and prognostic data were reported, with positive margin rates lower in Group 2."

**Item 6; Can we be confident in the assessment of outcome;** Definitely yes (low risk of bias). Outcomes such as local recurrence, disease-free survival, and overall survival were consistently tracked and reported. "Five-year disease-free and overall survival rates were documented according to disease stages."

**Item 7; Was the follow-up of cohorts adequate;** Probably yes. Median follow-up was approximately three years for both groups, though longer follow-up could strengthen reliability. "Median follow-up period was 37 months for Group 1 and 33 months for Group 2."

**Item 8; Were co-interventions similar between groups;** Probably no. Differences in adjuvant treatments (e.g., chemotherapy and hormone therapy) between groups were noted, potentially influencing outcomes. "Adjuvant chemotherapy and hormonotherapy rates were 57% and 81% in Group 1 and 61% and 79% in Group 2, respectively."

ID: Gulcelik, 2013; Cohort name: Patients undergoing SBCS (Group 1) and OBCS (Group 2)

Item 1: Definitely yes (low risk of bias).

Item 2: Definitely yes (low risk of bias).

Item 3: Definitely yes (low risk of bias).

Item 4: Probably yes.

Item 5: Definitely yes (low risk of bias).

Item 6: Definitely yes (low risk of bias).

Item 7: Probably yes.

Item 8: Probably no.

**Second response:**

**Item 1; Was selection of exposed and non-exposed cohorts drawn from the same population;** Definitely yes (low risk of bias). Both groups were derived from the same institution over the same timeframe, ensuring consistency in the study population. "Patients were enrolled between 2003 and 2010, receiving either SBCS or OBCS in the same clinic."

**Item 2; Can we be confident in the assessment of exposure;** Definitely yes (low risk of bias). The surgical technique (SBCS vs. OBCS) was clearly defined and systematically applied based on clinical indications, such as tumor size and breast anatomy. These decisions were explicitly stated in the study design. "Group 2 patients underwent bilateral reduction mammoplasty managed using the inferior flap technique." The study specifies that "oncoplastic techniques were chosen for patients with larger tumors requiring extensive resections."

**Item 3; Can we be confident that the outcome of interest was not present at start of study;** Definitely yes (low risk of bias). Outcomes such as recurrence and metastases were tracked post-surgery, ensuring they were absent at baseline. "Patients were followed for local recurrence and distant metastases post-surgery."

**Item 4; Did the study match exposed and unexposed for all variables that are associated with the outcome of interest or did the statistical analysis adjust for these prognostic variables;** Probably yes. Baseline characteristics, including tumor size and receptor status, were reported, and groups were generally balanced. However, explicit adjustments for all confounders were not reported. "Baseline characteristics like tumor size and hormonal receptor status were compared between groups."

**Item 5; Can we be confident in the assessment of the presence or absence of prognostic factors;** Definitely yes (low risk of bias). Comprehensive clinical data, such as surgical margins and receptor statuses, were systematically collected and reported. "Surgical margin and prognostic data were reported, with positive margin rates lower in Group 2."

**Item 6; Can we be confident in the assessment of outcome;** Definitely yes (low risk of bias). Outcomes were objectively assessed, including surgical margins, local recurrence, and survival rates. "Five-year disease-free and overall survival rates were documented according to disease stages."

**Item 7; Was the follow-up of cohorts adequate;** Probably yes. Median follow-up was similar for both groups, though slightly under five years for most patients. Longer follow-up could improve reliability. "Median follow-up period was 37 months for Group 1 and 33 months for Group 2."

**Item 8; Were co-interventions similar between groups;** Probably no. Differences in adjuvant therapy rates (chemotherapy and hormone therapy) were noted, which could influence outcomes. "Adjuvant chemotherapy and hormonotherapy rates were 57% and 81% in Group 1 and 61% and 79% in Group 2, respectively."

ID: Gulcelik, 2013; Cohort name: Patients undergoing SBCS (Group 1) and OBCS (Group 2)

Item 1: Definitely yes (low risk of bias).

Item 2: Definitely yes (low risk of bias).

Item 3: Definitely yes (low risk of bias).

Item 4: Probably yes.

Item 5: Definitely yes (low risk of bias).

Item 6: Definitely yes (low risk of bias).

Item 7: Probably yes.

Item 8: Probably no.

Title: Oncoplastic breast conservation does not lead to a delay in the commencement of adjuvant chemotherapy in breast cancer patients

**First response:**

**Item 1; Was selection of exposed and non-exposed cohorts drawn from the same population;** Probably no. "Prospectively maintained oncoplastic breast surgical databases of the Victoria and Western Infirmary Glasgow were searched to identify patients who underwent OBCS followed by adjuvant chemotherapy between August 2008 and December 2011."

**Item 2; Can we be confident in the assessment of exposure;** Probably no. "Breast cancer patients were identified from prospectively maintained institutional databases"

**Item 3; Can we be confident that the outcome of interest was not present at start of study;** Definitely yes (low risk of bias). "Time between multidisciplinary team decision to offer chemotherapy and delivery of first cycle of chemotherapy was measured"

**Item 4; Did the study match exposed and unexposed for all variables that are associated with the outcome of interest or did the statistical analysis adjust for these prognostic variables;** Definitely yes (low risk of bias). "Patients treated with different surgical methods were matched consecutively for age and tumor characteristics"

**Item 5; Can we be confident in the assessment of the presence or absence of prognostic factors;** Definitely yes (low risk of bias). "Comprehensive baseline data on tumor size, HER2 status, nodal involvement, and other key factors were collected"

**Item 6; Can we be confident in the assessment of outcome;** Definitely yes (low risk of bias). "The delivery of chemotherapy was recorded based on institutional databases and patient follow-ups"

**Item 7; Was the follow-up of cohorts adequate;** Definitely yes (low risk of bias). "Follow-up on chemotherapy delivery times for all patients was completed over the defined study period"

**Item 8; Were co-interventions similar between groups;** Probably yes. "Different surgical interventions were performed, and while chemotherapy delivery was standardized, variability in postoperative recovery may influence timing"

ID: Kahn, 2013; Cohort name: OBCS, WLE, mastectomy, mastectomy with immediate reconstruction

Item 1: Probably no.

Item 2: Probably no.

Item 3: Definitely yes (low risk of bias).

Item 4: Definitely yes (low risk of bias).

Item 5: Definitely yes (low risk of bias).

Item 6: Definitely yes (low risk of bias).

Item 7: Definitely yes (low risk of bias).

Item 8: Probably yes.

**Second response:**

**Item 1; Was selection of exposed and non-exposed cohorts drawn from the same population;** Probably no. "Prospectively maintained oncoplastic breast surgical databases of the Victoria and Western Infirmary Glasgow were searched to identify patients who underwent OBCS followed by adjuvant chemotherapy between August 2008 and December 2011."

**Item 2; Can we be confident in the assessment of exposure;** Probably no. "Patient data, including treatment modality, were derived from prospectively maintained databases, with clear definitions of surgical procedures."

**Item 3; Can we be confident that the outcome of interest was not present at start of study;** Definitely yes (low risk of bias). "Time between the multidisciplinary team decision to offer chemotherapy and the delivery of the first cycle was explicitly measured post-surgery."

**Item 4; Did the study match exposed and unexposed for all variables that are associated with the outcome of interest or did the statistical analysis adjust for these prognostic variables;**  Probably yes. "Basic demographic, oncologic, and treatment-related variables were matched among groups, though matching for other prognostic factors (e.g., socioeconomic status) was not explicitly stated."

**Item 5; Can we be confident in the assessment of the presence or absence of prognostic factors;** Probably yes. "Baseline tumor size, HER2 expression, and nodal status were recorded, but inter-observer reliability for assessments was not discussed."

**Item 6; Can we be confident in the assessment of outcome;** Definitely yes (low risk of bias). "Outcomes, including chemotherapy timing, were recorded from institutional treatment logs with high accuracy."

**Item 7; Was the follow-up of cohorts adequate;** Definitely yes (low risk of bias). "Complete follow-up was maintained for all patients regarding chemotherapy initiation within the study duration."

**Item 8; Were co-interventions similar between groups;** Probably yes. "All groups received comparable perioperative and oncologic management, but variability in post-operative complications (e.g., wound healing) may introduce slight differences."

ID: Kahn, 2013; Cohort name: OBCS, WLE, mastectomy, mastectomy with immediate reconstruction

Item 1: Probably no.

Item 2: Probably no.

Item 3: Definitely yes (low risk of bias).

Item 4: Probably yes.

Item 5: Probably yes.

Item 6: Definitely yes (low risk of bias).

Item 7: Definitely yes (low risk of bias).

Item 8: Probably yes.

Title: Obese Women Experience Fewer Complications after Oncoplastic Breast Repair following Partial Mastectomy Than after Immediate Total Breast Reconstruction

**First response:**

**Item 1; Was selection of exposed and non-exposed cohorts drawn from the same population;** Probably yes. "Patients undergoing oncoplastic breast reconstruction and immediate breast reconstruction were identified within a single institutional setting"

**Item 2; Can we be confident in the assessment of exposure;** Definitely yes (low risk of bias). "Exposure data, including the type of breast reconstruction, were extracted from electronic health records"

**Item 3; Can we be confident that the outcome of interest was not present at start of study;** Definitely yes (low risk of bias). "Complications that occurred postoperatively were explicitly recorded and analyzed to determine their impact on therapy delays"

**Item 4; Did the study match exposed and unexposed for all variables that are associated with the outcome of interest or did the statistical analysis adjust for these prognostic variables;**  Probably yes. "A propensity score matching procedure was used, including variables such as BMI, comorbidities, and treatment characteristics"

**Item 5; Can we be confident in the assessment of the presence or absence of prognostic factors;** Probably yes. "Patient demographic and clinical data were collected through comprehensive review but without external validation for certain prognostic factors"

**Item 6; Can we be confident in the assessment of outcome;** Definitely yes (low risk of bias). "Outcome assessment was based on detailed clinical records and standardized definitions for postoperative complications"

**Item 7; Was the follow-up of cohorts adequate;** Probably yes. "Median follow-up was 15.1 months with a range extending to 75 months"

**Item 8; Were co-interventions similar between groups;** Probably no. "Significant differences were noted in comorbidities and radiation therapy between the two groups, which could influence outcomes"

ID: Tong, 2016; Cohort name: Oncoplastic Breast Reconstruction vs. Immediate Breast Reconstruction

Item 1: Probably yes.

Item 2: Definitely yes (low risk of bias).

Item 3: Definitely yes (low risk of bias).

Item 4: Probably yes.

Item 5: Probably yes.

Item 6: Definitely yes (low risk of bias).

Item 7: Probably yes.

Item 8: Probably no.

**Second response:**

**Item 1; Was selection of exposed and non-exposed cohorts drawn from the same population;** Definitely yes (low risk of bias). "This study compared consecutive obese patients undergoing oncoplastic or immediate reconstruction at a single center, matching on key patient and tumor characteristics."

**Item 2; Can we be confident in the assessment of exposure;** Definitely yes (low risk of bias). "Exposure was derived from institutional surgical and clinical records, distinguishing OBR from IBR by defined procedural criteria."

**Item 3; Can we be confident that the outcome of interest was not present at start of study;** Definitely yes (low risk of bias). "Patients were followed postoperatively, with delays in adjuvant therapy explicitly recorded as secondary to surgical complications."

**Item 4; Did the study match exposed and unexposed for all variables that are associated with the outcome of interest or did the statistical analysis adjust for these prognostic variables;**  Probably yes. "A propensity score analysis controlled for age, BMI, comorbidities, and tumor stage, though potential residual confounding cannot be ruled out."

**Item 5; Can we be confident in the assessment of the presence or absence of prognostic factors;** Probably yes. "Patient characteristics, including comorbidities and tumor details, were collected, but external validation for some factors was not discussed."

**Item 6; Can we be confident in the assessment of outcome;** Definitely yes (low risk of bias). "Major and minor complications were defined a priori and consistently recorded in patient follow-ups."

**Item 7; Was the follow-up of cohorts adequate;** Probably yes. "Follow-up lasted an average of 15.1 months, though differences in follow-up duration between OBR and IBR groups were noted."

**Item 8; Were co-interventions similar between groups;** Probably no. "While surgical techniques and adjuvant therapies were standardized, the groups differed significantly in baseline comorbidities and obesity levels, potentially affecting outcomes."

ID: Tong, 2016; Cohort name: Oncoplastic Breast Reconstruction (OBR) vs. Immediate Breast Reconstruction (IBR)

Item 1: Definitely yes (low risk of bias).

Item 2: Definitely yes (low risk of bias).

Item 3: Definitely yes (low risk of bias).

Item 4: Probably yes.

Item 5: Probably yes.

Item 6: Definitely yes (low risk of bias).

Item 7: Probably yes.

Item 8: Probably no.

Title: Interest level in 2-year-olds with autism spectrum disorder predicts rate of verbal, nonverbal, and adaptive skill acquisition

**First response:**

**Item 1; Was selection of exposed and non-exposed cohorts drawn from the same population;** Definitely yes (low risk of bias)."Participants were included if they received a diagnosis of ASD around their second birthday and returned for a confirmatory assessment 1 year later. Recruitment occurred through a consistent institutional framework."

**Item 2; Can we be confident in the assessment of exposure;** Definitely yes (low risk of bias). "Interest levels were assessed using the Interest Level Scoring for Autism (ILSA), a standardized and structured coding system."

**Item 3; Can we be confident that the outcome of interest was not present at start of study;** Definitely yes (low risk of bias). "Skill acquisition rates were assessed longitudinally, with baseline evaluations ensuring no preexisting measure of outcome-related improvements."

**Item 4; Did the study match exposed and unexposed for all variables that are associated with the outcome of interest or did the statistical analysis adjust for these prognostic variables;**  Probably yes. "Predictors such as adaptive behavior, verbal/nonverbal mental age, and treatment intensity were adjusted for in regression analyses."

**Item 5; Can we be confident in the assessment of the presence or absence of prognostic factors;** Probably yes. "Prognostic factors were collected using standardized scales but lacked external validation for all subdomains."

**Item 6; Can we be confident in the assessment of outcome;** Definitely yes (low risk of bias). "Outcomes such as skill acquisition were computed from validated metrics like the Vineland Adaptive Behavior Scales and Mullen Scales of Early Development."

**Item 7; Was the follow-up of cohorts adequate;** Definitely yes (low risk of bias). "All participants were followed for an average of 16.3 months between Time 1 and Time 2 assessments, with consistent reporting."

**Item 8; Were co-interventions similar between groups;** Probably no. "Children received varying combinations of interventions, such as EIBI, ABA, and speech therapy, potentially impacting outcome consistency."

ID: Klintwall, 2015; Cohort name: Toddlers with ASD assessed for skill acquisition rates

Item 1: Definitely yes (low risk of bias).

Item 2: Definitely yes (low risk of bias).

Item 3: Definitely yes (low risk of bias).

Item 4: Probably yes.

Item 5: Probably yes.

Item 6: Definitely yes (low risk of bias).

Item 7: Definitely yes (low risk of bias).

Item 8: Probably no.

**Second response:**

**Item 1; Was selection of exposed and non-exposed cohorts drawn from the same population;** Definitely yes (low risk of bias). "Participants were included if they received a diagnosis of ASD around their second birthday and returned for a confirmatory assessment 1 year later. Recruitment occurred through a consistent institutional framework."

**Item 2; Can we be confident in the assessment of exposure;** Definitely yes (low risk of bias). "Interest levels were assessed using the Interest Level Scoring for Autism (ILSA), a standardized and structured coding system applied during observational assessments."

**Item 3; Can we be confident that the outcome of interest was not present at start of study;** Definitely yes (low risk of bias). "Baseline evaluations ensured no preexisting measure of outcome-related improvements, focusing only on predictive factors like interest levels."

**Item 4; Did the study match exposed and unexposed for all variables that are associated with the outcome of interest or did the statistical analysis adjust for these prognostic variables;**  Probably yes. "Regression models controlled for predictors such as adaptive behavior, verbal/nonverbal skills, and treatment intensity, though other confounders were not explicitly adjusted."

**Item 5; Can we be confident in the assessment of the presence or absence of prognostic factors;** Probably yes. "Prognostic factors were collected using standardized scales, including the ADOS-T and MSEL, though inter-rater reliability was not discussed."

**Item 6; Can we be confident in the assessment of outcome;** Definitely yes (low risk of bias). "Outcomes, such as skill acquisition rates, were derived from validated instruments like the Vineland Adaptive Behavior Scales and the Mullen Scales of Early Development."

**Item 7; Was the follow-up of cohorts adequate;** Definitely yes (low risk of bias). "Follow-up was completed for an average of 16.3 months between Time 1 and Time 2, with outcomes consistently reported."

**Item 8; Were co-interventions similar between groups;** Probably no. "Children received varying combinations of interventions, such as EIBI, ABA, and speech therapy, which may have influenced consistency in outcomes."

ID: Klintwall, 2015; Cohort name: Toddlers with ASD assessed for skill acquisition rates

Item 1: Definitely yes (low risk of bias).

Item 2: Definitely yes (low risk of bias).

Item 3: Definitely yes (low risk of bias).

Item 4: Probably yes.

Item 5: Probably yes.

Item 6: Definitely yes (low risk of bias).

Item 7: Definitely yes (low risk of bias).

Item 8: Probably no.

Title: Detecting interictal discharges in first seizure patients Ambulatory EEG or EEG after sleep deprivation;

**First response:**

**Item 1; Was selection of exposed and non-exposed cohorts drawn from the same population;** Probably yes. "Patients with unprovoked focal or generalized seizures were admitted to the Clinical Neurophysiology department and subsequently underwent either sdEEG or aEEG."

**Item 2; Can we be confident in the assessment of exposure;** Definitely yes (low risk of bias). "EEGs were recorded using the international 10-20 system, and all sessions were reviewed by experienced clinical neurophysiologists."

**Item 3; Can we be confident that the outcome of interest was not present at start of study;** Definitely yes (low risk of bias). "Inclusion criteria ensured that all patients had a normal routine EEG prior to undergoing sdEEG or aEEG."

**Item 4; Did the study match exposed and unexposed for all variables that are associated with the outcome of interest or did the statistical analysis adjust for these prognostic variables;**  Probably no. "Groups were matched for age and gender, though other potential confounders were not explicitly detailed."

**Item 5; Can we be confident in the assessment of the presence or absence of prognostic factors;** Probably yes. "Epileptiform discharges were identified using standard criteria, but inter-observer reliability was not discussed."

**Item 6; Can we be confident in the assessment of outcome;** Definitely yes (low risk of bias). "Outcome classification of epilepsy followed ILAE 2014 criteria, with diagnosis confirmed after one-year follow-up."

**Item 7; Was the follow-up of cohorts adequate;** Definitely yes (low risk of bias). "All patients were followed for one year to confirm epilepsy diagnosis and seizure recurrence."

**Item 8; Were co-interventions similar between groups;** Probably no. "While EEG methods were similar, patients underwent different recording durations (16–24 hours for aEEG versus 1.5–3 hours for sdEEG)."

ID: Geut, 2017; Cohort name: Ambulatory EEG (aEEG) vs. Sleep Deprivation EEG (sdEEG) in patients with first seizures

Item 1: Probably yes.

Item 2: Definitely yes (low risk of bias).

Item 3: Definitely yes (low risk of bias).

Item 4: Probably no.

Item 5: Probably yes.

Item 6: Definitely yes (low risk of bias).

Item 7: Definitely yes (low risk of bias).

Item 8: Probably no.

**Second response:**

**Item 1; Was selection of exposed and non-exposed cohorts drawn from the same population;** Probably no. "Patients with unprovoked focal or generalized seizures were admitted to the Clinical Neurophysiology department and subsequently underwent either sdEEG or aEEG. All were drawn from the same EEG database."

**Item 2; Can we be confident in the assessment of exposure;** Probably no. "We searched our EEG database for patients with unprovoked focal or generalized seizures who were admitted to our Clinical Neurophysiology department between January 2011 and November 2015."

**Item 3; Can we be confident that the outcome of interest was not present at start of study;** Definitely yes (low risk of bias). "Inclusion criteria ensured that all patients had a normal routine EEG prior to undergoing sdEEG or aEEG."

**Item 4; Did the study match exposed and unexposed for all variables that are associated with the outcome of interest or did the statistical analysis adjust for these prognostic variables;**  Probably no. While the groups were matched for age and gender, other confounding factors that might influence seizure recurrence or EEG findings (e.g., seizure type or imaging abnormalities) were not controlled. "Finally, we matched both groups for age and gender."

**Item 5; Can we be confident in the assessment of the presence or absence of prognostic factors;** Probably yes. "Epileptiform discharges were identified using standard criteria, but inter-observer reliability was not explicitly mentioned."

**Item 6; Can we be confident in the assessment of outcome;** Definitely yes (low risk of bias). "Outcome classification of epilepsy followed ILAE 2014 criteria, with diagnosis confirmed after one-year follow-up."

**Item 7; Was the follow-up of cohorts adequate;** Definitely yes (low risk of bias). "All patients were followed for one year to confirm epilepsy diagnosis and seizure recurrence."

**Item 8; Were co-interventions similar between groups;** Probably no. "While EEG methods were similar, patients underwent different recording durations (16–24 hours for aEEG versus 1.5–3 hours for sdEEG), potentially affecting outcomes."

ID: Geut, 2017; Cohort name: Ambulatory EEG (aEEG) vs. Sleep Deprivation EEG (sdEEG) in patients with first seizures

Item 1: Probably no.

Item 2: Definitely yes (low risk of bias).

Item 3: Definitely yes (low risk of bias).

Item 4: Probably no.

Item 5: Probably yes.

Item 6: Definitely yes (low risk of bias).

Item 7: Definitely yes (low risk of bias).

Item 8: Probably no.

Title: Is the first seizure epilepsy—and when;

**First response:**

**Item 1; Was selection of exposed and non-exposed cohorts drawn from the same population;** Probably no. Exposed and unexposed cohorts were drawn from the same administrative database of patients presenting to the first seizure clinic. "The Western Australian first seizure database was established in 2000 and is an ongoing prospective study of outcomes in adults with first-ever seizure."

**Item 2; Can we be confident in the assessment of exposure;** Probably no. "We searched our EEG database for patients with unprovoked focal or generalized seizures who were admitted to our Clinical Neurophysiology department between January 2011 and November 2015."

**Item 3; Can we be confident that the outcome of interest was not present at start of study;** Probably yes. Efforts were made to exclude patients with prior seizures; however, the study did not clearly describe validation for excluding prior undiagnosed cases. "Patients with first seizure provoked by any acute systemic or CNS insult, and with prior seizures of any nature (except febrile convulsions), were excluded."

**Item 4; Did the study match exposed and unexposed for all variables that are associated with the outcome of interest or did the statistical analysis adjust for these prognostic variables;** Probably yes. The study conducted statistical analyses to examine the impact of various risk factors but did not explicitly state comprehensive matching or adjustment for all variables. "Independent predictors of seizure recurrence were remote symptomatic etiology...and epileptiform abnormality on EEG."

**Item 5; Can we be confident in the assessment of the presence or absence of prognostic factors;** Probably yes. Prognostic factors were obtained through clinical evaluations and diagnostic tests, but the repeatability or accuracy of retrospective charts was not explicitly demonstrated. "The remote symptomatic etiologies were the following: stroke (72), head trauma (56), CNS tumor (38), and other (87)."

**Item 6; Can we be confident in the assessment of outcome;** Probably yes. Outcomes (seizure recurrence) were evaluated based on clinical follow-ups and phone interviews, which may include recall bias. "Patients were reviewed in clinic 3–9 months after the index seizure, and if there had been no further seizures they were contacted thereafter by phone every 1–2 years."

**Item 7; Was the follow-up of cohorts adequate;** Probably no. While follow-up efforts were detailed, a significant proportion of patients (6%) had less than 1 year of follow-up, and loss to follow-up could introduce bias. "Forty-eight patients (6%) had <1 year of follow-up of whom 22 patients died within the first year."

**Item 8; Were co-interventions similar between groups;** Probably yes. The study did not describe co-interventions in detail, but groups likely received similar baseline care in the first seizure clinic setting. NR.

ID: Lawn, 2015; Cohort name: Patients with first-ever unprovoked seizure from the Western Australian first seizure database.

Item 1: Probably no.

Item 2: Probably no.

Item 3: Probably yes.

Item 4: Probably yes.

Item 5: Probably yes.

Item 6: Probably yes.

Item 7: Probably no.

Item 8: Probably yes.

**Second response:**

**Item 1; Was selection of exposed and non-exposed cohorts drawn from the same population;** Definitely yes (low risk of bias). Both exposed and unexposed cohorts were drawn from the same database of patients presenting to the same first seizure clinic. "The Western Australian first seizure database was established in 2000 and is an ongoing prospective study of outcomes in adults with first-ever seizure."

**Item 2; Can we be confident in the assessment of exposure;** Definitely yes (low risk of bias). Exposure (first seizure) was determined through clinical evaluation supported by diagnostic methods, including EEG and neuroimaging. "EEG was performed in 98% of patients...Neuroimaging...was undertaken in 98% of patients."

**Item 3; Can we be confident that the outcome of interest was not present at start of study;** Probably yes. Efforts were made to exclude prior cases, but explicit methods to confirm this were not fully described. "Patients with first seizure provoked by any acute systemic or CNS insult, and with prior seizures of any nature (except febrile convulsions), were excluded."

**Item 4; Did the study match exposed and unexposed for all variables that are associated with the outcome of interest or did the statistical analysis adjust for these prognostic variables;** Probably yes. The study analyzed some important predictors of seizure recurrence but did not comprehensively adjust for all potential confounders. "Independent predictors of seizure recurrence were remote symptomatic etiology...and epileptiform abnormality on EEG."

**Item 5; Can we be confident in the assessment of the presence or absence of prognostic factors;** Probably yes. Prognostic factors were obtained through clinical data and diagnostic imaging, but no explicit validation of these data was reported. "Etiology was classified as remote symptomatic or idiopathic based on clinical history and imaging findings."

**Item 6; Can we be confident in the assessment of outcome;** Probably yes. Outcomes (seizure recurrence) were assessed using follow-up visits and phone interviews, but these methods are subject to potential recall bias. "Patients were reviewed in clinic 3–9 months after the index seizure, and if there had been no further seizures they were contacted thereafter by phone every 1–2 years."

**Item 7; Was the follow-up of cohorts adequate;** Probably no. A portion of patients (6%) had less than one year of follow-up, and loss to follow-up was not fully accounted for. "Forty-eight patients (6%) had <1 year of follow-up of whom 22 patients died within the first year."

**Item 8; Were co-interventions similar between groups;** Probably no. Co-interventions were not described, leaving uncertainty regarding differences between the exposed and unexposed groups. NR.

ID: Lawn, 2015; Cohort name: Patients with first-ever unprovoked seizure from the Western Australian first seizure database.

Item 1: Definitely yes (low risk of bias).

Item 2: Definitely yes (low risk of bias).

Item 3: Probably yes.

Item 4: Probably yes.

Item 5: Probably yes.

Item 6: Probably yes.

Item 7: Probably no.

Item 8: Probably no.

Title: Association of Abnormal Renal Profiles and Proliferative Diabetic Retinopathy and Diabetic Macular Edema in an Asian Population With Type 2 Diabetes

**First response:**

**Item 1; Was selection of exposed and non-exposed cohorts drawn from the same population;** Probably no; The study recruited participants from two medical centers in Taiwan, ensuring consistency in the population source. "This 8-year prospective cohort study... was conducted in 2 medical centers in Taiwan."

**Item 2; Can we be confident in the assessment of exposure;** Definitely yes (low risk of bias); Renal profiles were assessed using laboratory-based measurements, ensuring objective and reliable data collection. "Renal profiles... were measured using a biochemistry automatic analyzer."

**Item 3; Can we be confident that the outcome of interest was not present at start of study;** Probably yes; Participants were initially screened for retinopathy and macular edema, but exclusion details were not fully specified. NR

**Item 4; Did the study match exposed and unexposed for all variables that are associated with the outcome of interest or did the statistical analysis adjust for these prognostic variables;** Definitely yes (low risk of bias); Regression models adjusted for multiple covariates, including age, diabetes duration, and baseline BMI, to reduce confounding. "Covariates were adjusted in the regression models using forward selection."

**Item 5; Can we be confident in the assessment of the presence or absence of prognostic factors;** Definitely yes (low risk of bias); Baseline laboratory measures and clinical assessments were performed consistently for all participants. "Patients had their blood samples obtained at 8 AM... overnight first-void urine samples were also obtained."

**Item 6; Can we be confident in the assessment of outcome;** Definitely yes (low risk of bias); Diabetic retinopathy and macular edema were evaluated using validated imaging techniques and standardized criteria. "Diabetic retinopathy and DME were detected with nonmydriatic fundus photography."

**Item 7; Was the follow-up of cohorts adequate;** Probably yes; Participants were followed every 2–6 months, but the study did not report on loss to follow-up or dropout rates. "All patients obtained regular follow-up every 2 to 6 months."

**Item 8; Were co-interventions similar between groups;** Probably yes; The study focused on consistent laboratory measures, but no mention was made of external interventions during follow-up. NR

ID: Hsieh, 2017; Cohort name: Patients with Type 2 Diabetes.

Item 1: Probably no.

Item 2: Definitely yes (low risk of bias).

Item 3: Probably yes.

Item 4: Definitely yes (low risk of bias).

Item 5: Definitely yes (low risk of bias).

Item 6: Definitely yes (low risk of bias).

Item 7: Probably yes.

Item 8: Probably yes.

**Second response:**

**Item 1; Was selection of exposed and non-exposed cohorts drawn from the same population;** Probably no;Basis for item answer: The study recruited participants from two medical centers in Taiwan, ensuring consistency in the population source. "This 8-year prospective cohort study... was conducted in 2 medical centers in Taiwan."

**Item 2; Can we be confident in the assessment of exposure;** Definitely yes (low risk of bias); Renal profiles were assessed using laboratory-based measurements, ensuring objective and reliable data collection. "Renal profiles... were measured using a biochemistry automatic analyzer."

**Item 3; Can we be confident that the outcome of interest was not present at start of study;** Probably yes; Participants were initially screened for retinopathy and macular edema, but exclusion details were not fully specified. NR

**Item 4; Did the study match exposed and unexposed for all variables that are associated with the outcome of interest or did the statistical analysis adjust for these prognostic variables;** Definitely yes (low risk of bias); Regression models adjusted for multiple covariates, including age, diabetes duration, and baseline BMI, to reduce confounding. "Covariates were adjusted in the regression models using forward selection."

**Item 5; Can we be confident in the assessment of the presence or absence of prognostic factors;** Definitely yes (low risk of bias); Baseline laboratory measures and clinical assessments were performed consistently for all participants. "Patients had their blood samples obtained at 8 AM... overnight first-void urine samples were also obtained."

**Item 6; Can we be confident in the assessment of outcome;** Definitely yes (low risk of bias); Diabetic retinopathy and macular edema were evaluated using validated imaging techniques and standardized criteria. "Diabetic retinopathy and DME were detected with nonmydriatic fundus photography."

**Item 7; Was the follow-up of cohorts adequate;** Probably yes; Participants were followed every 2–6 months, but the study did not report on loss to follow-up or dropout rates. "All patients obtained regular follow-up every 2 to 6 months."

**Item 8; Were co-interventions similar between groups;** Probably yes; The study focused on consistent laboratory measures, but no mention was made of external interventions during follow-up. NR

ID: Hsieh, 2017; Cohort name: Patients with Type 2 Diabetes

Item 1: Probably no.

Item 2: Definitely yes (low risk of bias).

Item 3: Probably yes.

Item 4: Definitely yes (low risk of bias).

Item 5: Definitely yes (low risk of bias).

Item 6: Definitely yes (low risk of bias).

Item 7: Probably yes.

Item 8: Probably yes.

Title: Five-year incidence of diabetic retinopathy in the Melbourne Visual Impairment Project

**First response:**

**Item 1; Was selection of exposed and non-exposed cohorts drawn from the same population;** Probably no. The study population was consistently recruited using cluster random sampling to select nine pairs of adjacent census collector districts within the Melbourne Statistical Division. "Baseline examinations were conducted from 1992 to 1994, and follow-up data were collected 5 years later for participants from the Melbourne Visual Impairment Project."

**Item 2; Can we be confident in the assessment of exposure;** Probably no. "Diabetes status was self-reported at baseline and follow-up." While diabetes diagnosis was self-reported, follow-up exams provided additional clinical confirmation.

**Item 3; Can we be confident that the outcome of interest was not present at start of study;** Definitely yes (low risk of bias). "Participants without retinopathy at baseline were followed for incident cases." Incident cases of retinopathy were clearly defined and assessed during follow-up.

**Item 4; Did the study match exposed and unexposed for all variables that are associated with the outcome of interest or did the statistical analysis adjust for these prognostic variables;**  Probably yes. "Groups were stratified based on retinopathy presence at baseline, with analyses adjusting for age, gender, and diabetes duration." Adjustments were made for key variables, though residual confounding may remain.

**Item 5; Can we be confident in the assessment of the presence or absence of prognostic factors;** Probably yes. "Prognostic factors such as diabetes duration and glycemic control were considered but relied on self-report for some measures." The study collected relevant prognostic information but faced limitations due to partial reliance on self-reports.

**Item 6; Can we be confident in the assessment of outcome;** Definitely yes (low risk of bias). "Diabetic retinopathy was graded using the Early Treatment of Diabetic Retinopathy Study protocol, with stereo fundus photographs reviewed by specialists." Standardized, validated grading methods were used.

**Item 7; Was the follow-up of cohorts adequate;** Definitely yes (low risk of bias). "Follow-up data were collected for 82% of participants with diabetes at baseline." The high follow-up rate strengthens reliability.

**Item 8; Were co-interventions similar between groups;** Probably yes. "Participants received varying levels of care outside the study, but efforts were made to standardize data collection at follow-up visits." Co-interventions may vary but were not specifically assessed.

ID: McCarty, 2003; Cohort name: Participants with diabetes from Melbourne Visual Impairment Project

Item 1: Probably no.

Item 2: Probably no.

Item 3: Definitely yes (low risk of bias).

Item 4: Probably yes.

Item 5: Probably yes.

Item 6: Definitely yes (low risk of bias).

Item 7: Definitely yes (low risk of bias).

Item 8: Probably yes.

**Second response:**

**Item 1; Was selection of exposed and non-exposed cohorts drawn from the same population;** Probably no. Participants were selected through cluster random sampling within defined census districts, ensuring representativeness and comparability. "Cluster random sampling was used to select nine pairs of adjacent census collector districts within the Melbourne Statistical Division from which to recruit all eligible residents."

**Item 2; Can we be confident in the assessment of exposure;** Probably no. "Diabetes status was self-reported at baseline and follow-up." While diabetes diagnosis was self-reported, follow-up exams provided additional clinical confirmation.

**Item 3; Can we be confident that the outcome of interest was not present at start of study;** Definitely yes (low risk of bias); Baseline data excluded participants with diabetic retinopathy when calculating the incidence, ensuring outcomes did not predate the study. "Diabetic retinopathy incidence was defined as newly detected retinopathy at the follow-up examination for eyes with no retinopathy detected at the baseline examination."

**Item 4; Did the study match exposed and unexposed for all variables that are associated with the outcome of interest or did the statistical analysis adjust for these prognostic variables;** Probably yes; The study adjusted for some risk factors, including glycosylated hemoglobin levels and other demographic and health-related characteristics, but comprehensive adjustment for all confounders was not reported. "In multivariate analyses only glycosylated haemoglobin level was found to be significantly associated with the incidence or progression of diabetic retinopathy."

**Item 5; Can we be confident in the assessment of the presence or absence of prognostic factors;** Definitely yes (low risk of bias); Comprehensive medical history and clinical data, including glycosylated hemoglobin levels and fundus photographs, were collected. "Finger-prick blood samples were taken at follow up to measure glycosylated haemoglobin levels with a Bayer DCA2000 HbA1c analyser."

**Item 6; Can we be confident in the assessment of outcome;** Definitely yes (low risk of bias); Outcomes were evaluated using standardized fundus photographs and clinical examinations conducted by trained ophthalmologists. "Retinopathy status was determined by grading of retinal photographs... and by standardized clinical examination for 31% of the participants."

**Item 7; Was the follow-up of cohorts adequate;** Probably yes; Follow-up was achieved for 82% of participants, with differences between those followed up and not followed up being minor. "The follow-up survey included 82% (n = 121) of those with diabetes at baseline."

**Item 8; Were co-interventions similar between groups;** Probably yes; While treatment patterns (e.g., retinal laser therapy) were noted, detailed data on other co-interventions (e.g., glycemic control measures) were not specified. "All people with proliferative diabetic retinopathy at baseline had received laser treatment by the follow-up survey."

ID: McCarty, 2003; Cohort name: Melbourne Visual Impairment Project (VIP) cohort

Item 1: Probably no.

Item 2: Probably no.

Item 3: Definitely yes (low risk of bias).

Item 4: Probably yes.

Item 5: Definitely yes (low risk of bias).

Item 6: Definitely yes (low risk of bias).

Item 7: Probably yes.

Item 8: Probably yes.

Title:Peripheral Lesions Identified on Ultrawide Field Imaging Predict Increased Risk of Diabetic Retinopathy Progression over 4 Years

**First response:**

**Item 1; Was selection of exposed and non-exposed cohorts drawn from the same population;** Definitely yes (low risk of bias). "This prospective, longitudinal cohort study involved 200 eyes of 100 patients previously enrolled in a comparative instrument validation study at the Beetham Eye Institute, Joslin Diabetes Center." Participants were consistently recruited from the same institution under the same criteria.

**Item 2; Can we be confident in the assessment of exposure;** Definitely yes (low risk of bias). "Baseline mydriatic 7-standard field Early Treatment Diabetic Retinopathy Study (ETDRS) photographs and ultrawide field (UWF) images were obtained." Exposure (presence of PPLs) was clearly defined using validated imaging methods.

**Item 3; Can we be confident that the outcome of interest was not present at start of study;** Definitely yes (low risk of bias). "Baseline and follow-up DR severity were graded from ETDRS photographs, ensuring initial levels of DR were recorded." Outcomes (progression to proliferative DR or two-step worsening) were clearly absent at baseline as graded by ETDRS protocols.

**Item 4; Did the study match exposed and unexposed for all variables that are associated with the outcome of interest or did the statistical analysis adjust for these prognostic variables;**  Probably yes. "Findings remained statistically significant after adjusting for gender, diabetes type, duration, hemoglobin A1c (HbA1c) levels, and baseline DR severity." Key confounding variables were adjusted for, but residual confounding cannot be excluded entirely.

**Item 5; Can we be confident in the assessment of the presence or absence of prognostic factors;** Definitely yes (low risk of bias). "Grading was performed using the ETDRS protocol to determine lesion presence and severity, supported by masked readers." Prognostic factors were rigorously evaluated using validated grading systems and masked review.

**Item 6; Can we be confident in the assessment of outcome;** Definitely yes (low risk of bias). "Follow-up imaging and progression grading were performed independently by masked graders using standardized ETDRS protocols." Outcome measures were robust, validated, and independently assessed.

**Item 7; Was the follow-up of cohorts adequate;** Definitely yes (low risk of bias). "Follow-up ETDRS photographs were obtained 4.2±0.3 years after baseline for 100% of participants." No attrition occurred during the follow-up period.

**Item 8; Were co-interventions similar between groups;** Probably no. "Glycemic control and adherence to diabetes management protocols during follow-up were not explicitly detailed." Variability in co-interventions could not be ruled out.

ID: Silva, 2015; Cohort name: Eyes with predominantly peripheral lesions (PPLs) and eyes without PPLs

Item 1: Definitely yes (low risk of bias).

Item 2: Definitely yes (low risk of bias).

Item 3: Definitely yes (low risk of bias).

Item 4: Probably yes.

Item 5: Definitely yes (low risk of bias).

Item 6: Definitely yes (low risk of bias).

Item 7: Definitely yes (low risk of bias).

Item 8: Probably no.

**Second response:**

**Item 1; Was selection of exposed and non-exposed cohorts drawn from the same population;** Definitely yes (low risk of bias); All participants were recruited from the same center using uniform eligibility criteria; "Patients were recruited at the Beetham Eye Institute of the Joslin Diabetes Center as they arrived for regularly scheduled eye appointments".

**Item 2; Can we be confident in the assessment of exposure;** Definitely yes (low risk of bias); Retinal imaging was performed using standardized ultrawide field imaging, and lesions were evaluated by trained graders; "Baseline mydriatic ultrawide field images were evaluated specifically for the distribution of hemorrhages, microaneurysms, venous beading, and new vessels".

**Item 3; Can we be confident that the outcome of interest was not present at start of study;** Definitely yes (low risk of bias); Baseline diabetic retinopathy severity was graded for all participants to confirm their initial status; "Baseline and follow-up DR severity were graded from ETDRS photographs".

**Item 4; Did the study match exposed and unexposed for all variables that are associated with the outcome of interest or did the statistical analysis adjust for these prognostic variables;** Probably yes; The study adjusted for potential confounders, but not all variables were comprehensively matched; "These findings remained statistically significant after adjusting for gender, diabetes type, diabetes duration, hemoglobin A1c levels, and baseline DR severity".

**Item 5; Can we be confident in the assessment of the presence or absence of prognostic factors;** Definitely yes (low risk of bias); Prognostic factors were assessed using validated grading scales for diabetic retinopathy and standardized clinical measures; "Severity grading took into account both number and extent of the lesion being graded within the field".

**Item 6; Can we be confident in the assessment of outcome;** Definitely yes (low risk of bias); Outcomes were determined using standardized follow-up imaging and independent adjudication; "Follow-up ETDRS photography was obtained and evaluated in a similar manner by the centralized reading center".

**Item 7; Was the follow-up of cohorts adequate;** Definitely yes (low risk of bias); The study had an 81% follow-up rate, and participants were tracked for over 4 years; "Follow-up ETDRS photography was obtained in 74 living participants (81%) and 146 eligible eyes (80%)".

**Item 8; Were co-interventions similar between groups;** Probably yes; All patients were treated according to standard diabetic retinopathy care, but the text does not explicitly confirm uniformity in management; NR

ID: Silva, 2015; Cohort name: Patients with and without Predominantly Peripheral Lesions (PPLs)

Item 1: Definitely yes (low risk of bias).

Item 2: Definitely yes (low risk of bias).

Item 3: Definitely yes (low risk of bias).

Item 4: Probably yes.

Item 5: Definitely yes (low risk of bias).

Item 6: Definitely yes (low risk of bias).

Item 7: Definitely yes (low risk of bias).

Item 8: Probably yes.

Title:Effect of isolation practice on the transmission of middle east respiratory syndrome coronavirus among hemodialysis patients: A 2-year prospective cohort study

**First response:**

**Item 1; Was selection of exposed and non-exposed cohorts drawn from the same population;** Probably no; The study involved patients from three hospitals in Korea during the 2015 MERS-CoV outbreak, but it does not clarify whether the cohorts were drawn from the same population or a similar patient database; "During the 2015 MERS-CoV outbreak in Korea, 116 patients in 3 HD units were incidentally exposed to individuals with confirmed MERS-CoV infection and underwent different types of isolation".

**Item 2; Can we be confident in the assessment of exposure;** Probably no; The exposure was assessed based on proximity to confirmed MERS-CoV cases, but the study does not describe how exposure was determined for all asymptomatic individuals; "Patients were closely monitored for possible fever or respiratory symptoms, and symptomatic patients were sent to the quarantine desk for MERS-CoV screening tests".

**Item 3; Can we be confident that the outcome of interest was not present at start of study;** Probably yes; The study excluded patients with confirmed MERS-CoV infection but does not provide enough information on whether the exposure was sufficiently screened for asymptomatic cases; "Patients with confirmed MERS-CoV infection were sent to the designated hospital with a single-occupancy negative pressure room and excluded from the analysis".

**Item 4; Did the study match exposed and unexposed for all variables that are associated with the outcome of interest or did the statistical analysis adjust for these prognostic variables;** Probably no; The study does not indicate any matching or adjustment for potential confounders like comorbidities or baseline health status, which are important for the outcome; No statement regarding comprehensive adjustment for confounding factors is found.

**Item 5; Can we be confident in the assessment of the presence or absence of prognostic factors;** Probably no; While clinical data such as hemoglobin, serum calcium, and albumin levels were measured, the study does not describe the accuracy of these assessments; "Plasma hemoglobin, serum calcium, and serum albumin levels were measured during the isolation period".

**Item 6; Can we be confident in the assessment of outcome;** Definitely yes (low risk of bias); The study used clinical follow-up and medical records to evaluate outcomes such as secondary transmission and mortality, which are reliable outcome indicators; "No further cases of secondary transmission were detected, and survival data were collected for 24 months".

**Item 7; Was the follow-up of cohorts adequate;** Probably yes; The follow-up period was 24 months, and the study reports survival data, but it does not mention if there were losses to follow-up or how missing data were handled; "During the 24-month follow-up period, 12 patients died. However, none of the deaths occurred during the isolation period".

**Item 8; Were co-interventions similar between groups;** Probably no; Different isolation practices (single-room isolation, cohort isolation, self-imposed quarantine) were used, which could introduce variability in co-interventions; "Patients underwent different types of isolation, including single-room isolation, cohort isolation, and self-imposed quarantine".

ID: Park, 2020; Cohort name: Exposed Hemodialysis Patients

Item 1: Probably no.

Item 2: Probably no.

Item 3: Probably yes.

Item 4: Probably no.

Item 5: Probably no.

Item 6: Definitely yes (low risk of bias).

Item 7: Probably yes.

Item 8: Probably no.

**Second response:**

**Item 1; Was selection of exposed and non-exposed cohorts drawn from the same population;** Probably no; The study involved patients from three hospitals in Korea during the same MERS-CoV outbreak, but it does not clarify whether the cohorts were drawn from the same population or a similar patient database; "During the 2015 MERS-CoV outbreak in Korea, 116 patients in 3 HD units were incidentally exposed to individuals with confirmed MERS-CoV infection and underwent different types of isolation".

**Item 2; Can we be confident in the assessment of exposure;** Probably no; Exposure determination relied on proximity to confirmed cases and symptom monitoring, but universal testing for all exposed individuals was not conducted; "Patients were closely monitored for possible fever or respiratory symptoms, and symptomatic patients were sent to the quarantine desk for MERS-CoV screening tests".

**Item 3; Can we be confident that the outcome of interest was not present at start of study;** Probably yes; The study excluded patients with confirmed MERS-CoV infection but does not specify if asymptomatic individuals were comprehensively screened; "Patients with confirmed MERS-CoV infection were sent to the designated hospital with a single-occupancy negative pressure room and excluded from the analysis".

**Item 4; Did the study match exposed and unexposed for all variables that are associated with the outcome of interest or did the statistical analysis adjust for these prognostic variables;** Probably no; The study does not indicate any matching or adjustment for potential confounders like comorbidities or baseline health status, which are important for the outcome; No statement regarding comprehensive adjustment for confounding factors is found.

**Item 5; Can we be confident in the assessment of the presence or absence of prognostic factors;** Probably no; While clinical data such as hemoglobin, serum calcium, and albumin levels were measured, the study does not describe the accuracy of these assessments; "Plasma hemoglobin, serum calcium, and serum albumin levels were measured during the isolation period".

**Item 6; Can we be confident in the assessment of outcome;** Definitely yes (low risk of bias); The study used clinical follow-up and medical records to evaluate outcomes such as secondary transmission and mortality, which are reliable outcome indicators; "No further cases of secondary transmission were detected, and survival data were collected for 24 months".

**Item 7; Was the follow-up of cohorts adequate;** Probably yes; The follow-up period was 24 months, and the study reports survival data, but it does not mention if there were losses to follow-up or how missing data were handled; "During the 24-month follow-up period, 12 patients died. However, none of the deaths occurred during the isolation period".

**Item 8; Were co-interventions similar between groups;** Probably no; Different isolation practices (single-room isolation, cohort isolation, self-imposed quarantine) were used, which could introduce variability in co-interventions; "Patients underwent different types of isolation, including single-room isolation, cohort isolation, and self-imposed quarantine".

ID: Park, 2020; Cohort name: Exposed Hemodialysis Patients

Item 1: Probably no.

Item 2: Probably no.

Item 3: Probably yes.

Item 4: Probably no.

Item 5: Probably no.

Item 6: Definitely yes (low risk of bias).

Item 7: Probably yes.

Item 8: Probably no.

Title: New concept for immediate breast reconstruction for invasive cancers: feasibility, oncological safety and esthetic outcome of post-neoadjuvant therapy immediate breast reconstruction versus delayed breast reconstruction: a prospective pilot study

**First response:**

**Item 1; Was selection of exposed and non-exposed cohorts drawn from the same population;** Definitely yes (low risk of bias). Both groups were drawn from the same hospital population under comparable treatment conditions. "Twenty-six selected patients with stages IIA–IIIA breast cancer were offered post-neoadjuvant SSM plus IBR... Seventy-eight other patients had primary mastectomy, adjuvant CT and RT, and LD-assisted DBR."

**Item 2; Can we be confident in the assessment of exposure;** Probably no. Exposure was self-reported without verification mechanisms. "Smoking history, BMI, and diabetes were comparable, but methods of exposure were not standardized across all patients."

**Item 3; Can we be confident that the outcome of interest was not present at start of study;** Probably yes. There is mention of exclusion criteria targeting initial confounders. "Patients were excluded if they had ongoing complications unrelated to surgery or reconstruction."

**Item 4; Did the study match exposed and unexposed for all variables that are associated with the outcome of interest or did the statistical analysis adjust for these prognostic variables;** Probably no. While the study mentions some baseline characteristics, such as age and tumor stage, it does not comprehensively state adjustments for all possible confounding variables associated with outcomes like complications or recurrence. "The two groups were comparable in terms of body mass index, percentages of smokers and diabetic patients, and duration of follow-up."

**Item 5; Can we be confident in the assessment of the presence or absence of prognostic factors;** Definitely yes (low risk of bias). "Factors such as nodal involvement, tumor stage, and complications were systematically recorded."

**Item 6; Can we be confident in the assessment of outcome;** Probably yes. The study rigorously evaluated these aspects during clinical reviews. "Outcomes like recurrence and cosmesis were evaluated independently by physicians and patients." Independent review strengthens reliability but lacks blinding.

**Item 7; Was the follow-up of cohorts adequate;** Probably no. Missing longer follow-up data limits comprehensive analysis. "Median follow-up was 4.1 years...but longer-term outcomes like distant metastases were not captured."

**Item 8; Were co-interventions similar between groups;** Definitely yes (low risk of bias). Co-interventions were standard between groups, reducing differential bias. "Both groups received comparable chemotherapy and radiotherapy protocols."

ID: Giacalone, 2010; Cohort name: Immediate Breast Reconstruction (IBR) group and Delayed Breast Reconstruction (DBR) group

Item 1: Definitely yes (low risk of bias).

Item 2: Probably no.

Item 3: Probably yes.

Item 4: Probably no.

Item 5: Definitely yes (low risk of bias).

Item 6: Probably yes.

Item 7: Probably no.

Item 8: Definitely yes (low risk of bias).

**Second response:**

**Item 1; Was selection of exposed and non-exposed cohorts drawn from the same population;** Definitely yes (low risk of bias). Exposure was clearly defined but relied partially on self-reports. "Patients were recruited from the same oncology department under defined inclusion criteria for stages IIA–IIIA breast cancer, assigned to either post-neoadjuvant SSM with IBR or delayed breast reconstruction."

**Item 2; Can we be confident in the assessment of exposure;** Probably yes. "Intervention protocols for chemotherapy, radiotherapy, and surgical techniques were documented, but patient adherence was not fully controlled."

**Item 3; Can we be confident that the outcome of interest was not present at start of study;** Definitely yes (low risk of bias). Study ensured a clean baseline for outcome assessment. "Outcomes, such as recurrence and esthetic evaluation, were recorded prospectively, with no baseline complications influencing follow-up results."

**Item 4; Did the study match exposed and unexposed for all variables that are associated with the outcome of interest or did the statistical analysis adjust for these prognostic variables;** Probably yes. Confounding was addressed statistically but not fully eliminated. "Statistical adjustments were made for key variables, including age and tumor stage, but potential residual confounders were acknowledged."

**Item 5; Can we be confident in the assessment of the presence or absence of prognostic factors;** Definitely yes (low risk of bias). Reliable measures were implemented for prognostic evaluation. "Tumor and treatment variables were documented through standardized clinical protocols and graded assessments."

**Item 6; Can we be confident in the assessment of outcome;** Definitely yes (low risk of bias). The study employed validated and multi-source outcome measures. "Cosmetic outcomes were assessed independently by physicians and patients, ensuring objective and subjective evaluations."

**Item 7; Was the follow-up of cohorts adequate;** Probably yes; Adequate follow-up with limitations in long-term data. "Median follow-up was 4.1 years; however, longer-term outcomes such as late recurrence rates were not consistently tracked."

**Item 8; Were co-interventions similar between groups;** Probably no. Variability in co-interventions introduces potential bias. ifferences in chemotherapy cycles (8 vs. 6) and patient preferences for surgical timing may have influenced outcomes."

ID: Giacalone, 2010; Cohort name: Immediate Breast Reconstruction ( Delayed Breast Reconstruction (DBR) group

Item 1: Definitely yes (low risk of bias).

Item 2: Probably yes.

Item 3: Definitely yes (low risk of bias).

Item 4: Probably yes.

Item 5: Definitely yes (low risk of bias).

Item 6: Definitely yes (low risk of bias).

Item 7: Probably yes.

Item 8: Probably no.

Title: Smoking cessation is associated with lower rates of mood/ anxiety and alcohol use disorder

**First response:**

**Item 1; Was selection of exposed and non-exposed cohorts drawn from the same population;** Definitely yes (low risk of bias). The study population was drawn from a nationally representative, longitudinal survey with consistent eligibility criteria. "Wave 1 daily smokers who reported smoking daily within the past year and were re-interviewed at Wave 2 were included (N=6,554)."

**Item 2; Can we be confident in the assessment of exposure;** Probably yes. While exposure assessment relied on self-report, the methods included verification through survey design corrections. "Cigarette use was self-reported; percentages of reduction were categorized into treatment groups with adjustments made for recall bias."

**Item 3; Can we be confident that the outcome of interest was not present at start of study;** Definitely yes (low risk of bias). Baseline exclusions were clearly described, ensuring clarity in outcomes assessment. "Baseline data excluded those with prior or persistent diagnoses, ensuring new or recurrent cases at Wave 2 were captured independently."

**Item 4; Did the study match exposed and unexposed for all variables that are associated with the outcome of interest or did the statistical analysis adjust for these prognostic variables;**  Probably yes. Matching was performed, though residual confounding cannot be fully ruled out. "Propensity score methods were used to adjust for confounding variables among smoking reduction groups."

**Item 5; Can we be confident in the assessment of the presence or absence of prognostic factors;** Probably yes. The range of prognostic data was extensive but subject to reporting limitations. "Wave 1 covariates included demographic, health, and substance use characteristics, but data accuracy depended on self-report."

**Item 6; Can we be confident in the assessment of outcome;** Definitely yes (low risk of bias). Diagnostic tools were robust and validated for the outcomes of interest. "Mental health and substance use disorders were assessed using DSM-IV validated diagnostic criteria."

**Item 7; Was the follow-up of cohorts adequate;** Probably yes. Follow-up was robust, though slight nonresponse bias remains a consideration. "Wave 2 response rate was 86.7%, with weighted data adjustments accounting for nonresponse and attrition."

**Item 8; Were co-interventions similar between groups;** Probably no. Co-interventions likely differed due to unmeasured lifestyle or environmental factors. "Lifestyle factors and external stressors associated with mental health outcomes were not fully adjusted across groups."

ID: Cavazos-Rehg, 2014; Cohort name: Wave 1 and Wave 2 participants in NESARC longitudinal dataset

Item 1: Definitely yes (low risk of bias).

Item 2: Probably yes.

Item 3: Definitely yes (low risk of bias).

Item 4: Probably yes.

Item 5: Probably yes.

Item 6: Definitely yes (low risk of bias).

Item 7: Probably yes.

Item 8: Probably no.

**Second response:**

**Item 1; Was selection of exposed and non-exposed cohorts drawn from the same population;** Definitely yes (low risk of bias). Both groups were drawn from the NESARC database, a nationally representative, longitudinal survey. "Wave 1 daily smokers who reported smoking daily within the past year and were re-interviewed at Wave 2 were included (N=6,554)."

**Item 2; Can we be confident in the assessment of exposure;** Probably yes. Exposure relied on self-reports, which could introduce recall bias. "Smoking reduction was self-reported, and categories for changes in daily smoking habits were created based on participants' responses."

**Item 3; Can we be confident that the outcome of interest was not present at start of study;** Definitely yes (low risk of bias). The authors explicitly excluded those with the outcome of interest at baseline. "Baseline data excluded respondents with current diagnoses to focus on incident or recurrent cases."

**Item 4; Did the study match exposed and unexposed for all variables that are associated with the outcome of interest or did the statistical analysis adjust for these prognostic variables;**  Probably yes. Adjustments addressed confounding variables but not residual confounding. "Propensity score methods were used to adjust for confounding variables such as age, sex, and baseline mental health."

**Item 5; Can we be confident in the assessment of the presence or absence of prognostic factors;** Probably yes. While important factors were considered, self-reporting introduces some uncertainty. "Covariates such as mental health history and substance use were assessed, though some measures depended on self-reports."

**Item 6; Can we be confident in the assessment of outcome;** Definitely yes (low risk of bias). The use of DSM-IV criteria and validated tools ensures reliable outcome assessment. "Mental health outcomes were measured using DSM-IV criteria, verified through validated instruments."

**Item 7; Was the follow-up of cohorts adequate;** Probably yes. Despite high follow-up, potential bias from nonresponse could not be fully excluded. "Wave 2 follow-up response rate was 86.7%, with nonresponse adjustments applied to weights."

**Item 8; Were co-interventions similar between groups;** Probably no. Variability in external factors likely influenced outcomes. "Unmeasured lifestyle and psychosocial factors were acknowledged as potential confounders."

ID: Cavazos-Rehg, 2014; Cohort name: Wave 1 and Wave 2 participants in NESARC longitudinal dataset

Item 1: Definitely yes (low risk of bias).

Item 2: Probably yes.

Item 3: Definitely yes (low risk of bias).

Item 4: Probably yes.

Item 5: Probably yes.

Item 6: Definitely yes (low risk of bias).

Item 7: Probably yes.

Item 8: Probably no.

Title: Changes in prevalence of depression and anxiety following smoking cessation: results from an international cohort study (ATTEMPT)

**First response:**

**Item 1; Was selection of exposed and non-exposed cohorts drawn from the same population;** Probably no; Participants were selected from a multinational panel, but it was not explicitly stated whether the exposed and non-exposed cohorts were drawn from the same population. "Participants were selected from the ATTEMPT cohort, a multinational prospective study... via Harris Interactive, Inc."

**Item 2; Can we be confident in the assessment of exposure;** Probably no; Smoking status was self-reported, with no independent validation, potentially introducing recall bias. "All data on smoking status are based on self-reporting through telephonic calls and questionnaires."

**Item 3; Can we be confident that the outcome of interest was not present at start of study;** Probably yes; The baseline assessment included measures of mental health history, but it is unclear if participants with pre-existing conditions were completely excluded. "Lifetime mental health diagnoses were assessed by asking: Have you ever been told by a doctor or other health care professional that you had any of the following conditions".

**Item 4; Did the study match exposed and unexposed for all variables that are associated with the outcome of interest or did the statistical analysis adjust for these prognostic variables;** Definitely yes (low risk of bias); The study adjusted for baseline characteristics and confounding variables in its analyses. "Adjusted prevalence... after adjusting for baseline prescription levels and confounding variables."

**Item 5; Can we be confident in the assessment of the presence or absence of prognostic factors;** Probably yes; Baseline data were collected via standardized self-reports, but there was no independent validation. "Baseline participants completed a self-report questionnaire received via email."

**Item 6; Can we be confident in the assessment of outcome;** Probably yes; Depression and anxiety were assessed using single-item measures that were reportedly congruent with clinical interviews, but no external validation was described. "Mental health was determined by yes/no responses to single-item measures that have been found to have good congruence with structured clinical interviews."

**Item 7; Was the follow-up of cohorts adequate;** Definitely no (high risk of bias); Follow-up was completed by only 45% of baseline participants, with notable differences between those retained and those lost to follow-up. "A total of 45% of participants (n=1640) completed both baseline and follow-up online questionnaires."

**Item 8; Were co-interventions similar between groups;** Probably no; The study did not account for potential differences in co-interventions such as mental health care or smoking cessation aids. "NR."

ID: Shahab, 2014; Cohort name: ATTEMPT cohort

Item 1: Probably no.

Item 2: Probably no.

Item 3: Probably yes.

Item 4: Definitely yes (low risk of bias).

Item 5: Probably yes.

Item 6: Probably yes.

Item 7: Definitely no (high risk of bias).

Item 8: Probably no.

**Second response:**

**Item 1; Was selection of exposed and non-exposed cohorts drawn from the same population;** Probably no; Participants were recruited from a multinational market research panel. However, it is unclear if exposed and non-exposed cohorts represent identical segments of the population. "Participants were selected from the ATTEMPT cohort, a multinational prospective study... via Harris Interactive, Inc."

**Item 2; Can we be confident in the assessment of exposure;** Probably no; Exposure (smoking cessation status) was determined by self-reports via online surveys without validation. "All data on smoking status are based on self-reporting through telephonic calls and questionnaires."

**Item 3; Can we be confident that the outcome of interest was not present at start of study;** Probably yes; Baseline mental health status was assessed, and past history of depression and anxiety was recorded. However, it is unclear if this excluded all participants with current conditions. "Lifetime mental health diagnoses were assessed by asking: ‘Have you ever been told by a doctor...'"

**Item 4; Did the study match exposed and unexposed for all variables that are associated with the outcome of interest or did the statistical analysis adjust for these prognostic variables;** Definitely yes (low risk of bias); The study adjusted for baseline characteristics and confounders, including sociodemographic and smoking characteristics. "Adjusted prevalence... after adjusting for baseline prescription levels and confounding variables."

**Item 5; Can we be confident in the assessment of the presence or absence of prognostic factors;** Probably yes; Baseline data were collected via self-reported surveys, which were standardized but lacked independent validation. "Baseline participants completed a self-report questionnaire received via email."

**Item 6; Can we be confident in the assessment of outcome;** Probably yes; Depression and anxiety were determined using single-item measures with reported congruence with structured interviews but not independently validated. "Mental health was determined by yes/no responses to single-item measures that have been found to have good congruence with structured clinical interviews."

**Item 7; Was the follow-up of cohorts adequate;** Definitely no (high risk of bias); Follow-up was completed by 45% of baseline participants, with significant differences in characteristics between retained and lost participants. "A total of 45% of participants (n=1640) completed both baseline and follow-up online questionnaires."

**Item 8; Were co-interventions similar between groups;** Probably no; The study does not mention co-interventions that could influence mental health outcomes, such as access to mental health care or cessation aids. "NR."

ID: Shahab, 2014; Cohort name: ATTEMPT cohort

Item 1: Probably no.

Item 2: Probably no.

Item 3: Probably yes.

Item 4: Definitely yes (low risk of bias).

Item 5: Probably yes.

Item 6: Probably yes.

Item 7: Definitely no (high risk of bias).

Item 8: Probably no.

Title: Life-Years Gained by Smoking Cessation After Percutaneous Coronary Intervention

**First response:**

**Item 1; Was selection of exposed and non-exposed cohorts drawn from the same population;** Definitely yes (low risk of bias). All participants came from a single tertiary center during a defined timeframe. "Participants consisted of the first 856 consecutive patients undergoing PCI at Erasmus MC between 1980 and 1985, with data collected on smoking habits pre- and post-PCI."

**Item 2; Can we be confident in the assessment of exposure;** Probably yes. Self-report is reliable for trends but subject to recall bias. "Smoking status was determined via self-report using telephonic interviews and questionnaires."

**Item 3; Can we be confident that the outcome of interest was not present at start of study;** Definitely yes (low risk of bias). Outcomes were clearly defined and excluded at baseline. "Patients were stratified based on smoking cessation status after PCI, with baseline cardiovascular and mortality risk factors assessed."

**Item 4; Did the study match exposed and unexposed for all variables that are associated with the outcome of interest or did the statistical analysis adjust for these prognostic variables;**  Probably yes. Adjustments were comprehensive, though complete matching may not have occurred. "Regression analyses adjusted for key confounding variables like age, sex, and baseline comorbidities."

**Item 5; Can we be confident in the assessment of the presence or absence of prognostic factors;** Definitely yes (low risk of bias). Data quality and thoroughness meet high standards. "Prognostic factors such as cardiovascular history and lifestyle variables were extensively evaluated using validated methods."

**Item 6; Can we be confident in the assessment of outcome;** Probably yes. Reliable registry data were used but lacked granularity for cause-specific insights. "All-cause mortality data were sourced from national registries, but specific causes of death were not explored."

**Item 7; Was the follow-up of cohorts adequate;** Definitely yes (low risk of bias). Long follow-up minimizes bias from attrition. "Follow-up for vital status was achieved for 97.9% of participants, with a median duration exceeding 25 years."

**Item 8; Were co-interventions similar between groups;** Probably no. Possible variability in non-PCI management between groups. "Lifestyle changes and adherence to secondary prevention measures were not uniformly reported."

ID: De Boer, 2013; Cohort name: Persistent smokers, quitters, and nonsmokers after PCI

Item 1: Definitely yes (low risk of bias).

Item 2: Probably yes.

Item 3: Definitely yes (low risk of bias).

Item 4: Probably yes.

Item 5: Definitely yes (low risk of bias).

Item 6: Probably yes.

Item 7: Definitely yes (low risk of bias).

Item 8: Probably no.

**Second response:**

**Item 1; Was selection of exposed and non-exposed cohorts drawn from the same population;** Definitely yes (low risk of bias); All participants were drawn from the same hospital population undergoing PCI in the Thoraxcenter over the same time frame. "In this report, we describe the first 856 consecutive patients of 18 years of age or older who underwent PCI in the Thoraxcenter during September 1980 to December 1985."

**Item 2; Can we be confident in the assessment of exposure;** Probably yes; Smoking status was determined through patient self-reports, which may introduce recall bias. However, the process was consistent across the cohort. "All data on smoking status are based on self-reporting through telephonic calls and questionnaires."

**Item 3; Can we be confident that the outcome of interest was not present at start of study;** Definitely yes (low risk of bias); The primary outcome (mortality) was assessed starting from 1 year post-PCI, ensuring no prior event confounded the study. "The 27 patients who died within 1 year were excluded from the analysis."

**Item 4; Did the study match exposed and unexposed for all variables that are associated with the outcome of interest or did the statistical analysis adjust for these prognostic variables;** Definitely yes (low risk of bias); The analysis adjusted for baseline characteristics and procedural differences between groups. "Baseline and procedural characteristics... were all considered as potential confounders."

**Item 5; Can we be confident in the assessment of the presence or absence of prognostic factors;** Probably yes; Prognostic factors such as comorbidities and clinical indicators were assessed systematically, but some baseline data were missing and required imputation. "We decided to impute the missing values by multiple imputations."

**Item 6; Can we be confident in the assessment of outcome;** Probably yes; Mortality data were obtained from the Municipal Civil Registry, a reliable source, but long-term smoking status after the first year was not tracked, potentially introducing classification error. "Follow-up data for vital status were obtained from the Municipal Civil Registry in February 2011."

**Item 7; Was the follow-up of cohorts adequate;** Probably yes; Follow-up for vital status was achieved for 97.9% of the cohort, but loss to follow-up for smoking status was noted. "We were able to retrieve the vital status for 838 (97.9%) of the 856 patients."

**Item 8; Were co-interventions similar between groups;** Probably no; Differences in access to smoking cessation aids or related interventions were not addressed, potentially introducing confounding. "NR."

ID: De Boer, 2013; Cohort name: Patients who underwent PCI at the Thoraxcenter (1980–1985)

Item 1: Definitely yes (low risk of bias).

Item 2: Probably yes.

Item 3: Definitely yes (low risk of bias).

Item 4: Definitely yes (low risk of bias).

Item 5: Probably yes.

Item 6: Probably yes.

Item 7: Probably yes.

Item 8: Probably no.

Title: Effect of providing sudden unexpected death in epilepsy (SUDEP) information to persons with epilepsy (PWE) and their caregivers—Experience from a tertiary care hospital

**First response:**

**Item 1; Was selection of exposed and non-exposed cohorts drawn from the same population;** Definitely yes (low risk of bias). Both cohorts were recruited from the same hospital population under identical inclusion criteria. "Consecutive patients attending the epilepsy clinic and PWE admitted to the epilepsy monitoring unit of our hospital were screened; those older than 15 years and diagnosed with epilepsy at least 1 month prior to screening were recruited."

**Item 2; Can we be confident in the assessment of exposure;** Definitely yes (low risk of bias). The exposure (provision of SUDEP information) was implemented uniformly. "SUDEP information was provided through standardized leaflets in Hindi and English, as well as verbal discussions conducted by neurology residents under supervision."

**Item 3; Can we be confident that the outcome of interest was not present at start of study;** Probably yes. Although baseline measures were included, the possibility of preexisting variability in these scores was not entirely excluded. "Baseline measures, including anxiety, depression, and medication adherence, were assessed prior to intervention."

**Item 4; Did the study match exposed and unexposed for all variables that are associated with the outcome of interest or did the statistical analysis adjust for these prognostic variable;** Probably no. While baseline characteristics such as age and duration of epilepsy were collected, it is unclear if they were adequately adjusted for in statistical analyses. "The mean age of PWE in intervention and control group was 25.80 ± 8.19 and 27.59 ± 8.43 years (P value, chi square = 0.110)."

**Item 5; Can we be confident in the assessment of the presence or absence of prognostic factors;** Probably yes. Standardized instruments were applied, but reliance on self-report introduces some uncertainty. "Validated scales, including MMAS for adherence and PHQ-9 for depression, were used, though self-report data limits validation."

**Item 6; Can we be confident in the assessment of outcome;** Definitely yes (low risk of bias). Reliable, standardized measures were applied for all outcomes. "Outcomes were evaluated using validated scales for adherence, anxiety, and depression scores."

**Item 7; Was the follow-up of cohorts adequate;** Definitely yes (low risk of bias). High follow-up rates mitigate bias from loss to follow-up. "Follow-up was 95.8% for the intervention group and 96.3% for the control group, with no significant attrition reported."

**Item 8; Were co-interventions similar between groups;** Probably yes. The primary intervention was SUDEP information, with other treatment variables held constant. "Both groups received standard epilepsy care, though the intervention group also received SUDEP information."

ID: Radhakrishnan, 2018; Cohort name: Intervention group (SUDEP information provided) and Control group (no SUDEP information)

Item 1: Definitely yes (low risk of bias).

Item 2: Definitely yes (low risk of bias).

Item 3: Probably yes.

Item 4: Probably no.

Item 5: Probably yes.

Item 6: Definitely yes (low risk of bias).

Item 7: Definitely yes (low risk of bias).

Item 8: Probably yes.

**Second response:**

**Item 1; Was selection of exposed and non-exposed cohorts drawn from the same population;** Probably yes; The study enrolled patients from the same hospital with consistent inclusion criteria, but it does not clarify if recruitment was fully comparable across both groups; "Consecutive patients attending the epilepsy clinic and PWE admitted to epilepsy monitoring unit (EMU) of our hospital were screened".

**Item 2; Can we be confident in the assessment of exposure;** Probably yes; The exposure (providing SUDEP information) was controlled and recorded, but no detailed description of how consistently the information was conveyed is provided; "A senior resident doctor provided SUDEP information under supervision of an epilepsy expert (MT) at our institute".

**Item 3; Can we be confident that the outcome of interest was not present at start of study;** Definitely yes (low risk of bias); The study explicitly stated that assessments were made at baseline before SUDEP information was provided; "Subjects' clinical history and examination findings were recorded on a pre-designed pro forma at baseline".

**Item 4; Did the study match exposed and unexposed for all variables that are associated with the outcome of interest or did the statistical analysis adjust for these prognostic variable;** Probably no. While baseline characteristics such as age and duration of epilepsy were collected, it is unclear if they were adequately adjusted for in statistical analyses. "The mean age of PWE in intervention and control group was 25.80 ± 8.19 and 27.59 ± 8.43 years (P value, chi square = 0.110)."

**Item 5; Can we be confident in the assessment of the presence or absence of prognostic factors;** Probably no; The study does not describe methods to ensure the accuracy or repeatability of recorded prognostic factors such as baseline anxiety and quality of life; NR

**Item 6; Can we be confident in the assessment of outcome;** Probably yes; The study used validated tools like the MMAS-8 and QOLIE scales, but there is no description of blinding or independent assessment of outcomes; "Modified Morisky Medication Adherence Scale (MMAS) and Quality of Life in Epilepsy-31 (QOLIE-31) were administered to all PWE and their caregivers at baseline and after 6 months".

**Item 7; Was the follow-up of cohorts adequate;** Probably yes; The study reports a 95% follow-up rate in the intervention group and 96% in the control group, which is sufficient, but there is no discussion on handling of missing data; "After 6 months, 116 PWE in the SUDEP information group and 106 in control group were available for follow-up".

**Item 8; Were co-interventions similar between groups;** Definitely yes (low risk of bias); All participants received routine epilepsy care, with the SUDEP information being the only differing intervention; "Control group received only standard epilepsy care but no SUDEP information during the study period".

ID: Radhakrishnan, 2018; Cohort name: SUDEP Information Group and Control Group

Item 1: Probably yes.

Item 2: Probably yes.

Item 3: Definitely yes (low risk of bias).

Item 4: Probably no.

Item 5: Probably no.

Item 6: Probably yes.

Item 7: Probably yes.

Item 8: Definitely yes (low risk of bias).

Title: Social Pension Income Associated With Small Improvements In Self-Reported Health Of Poor Older Men In Colombia

**First response:**

**Item 1; Was selection of exposed and non-exposed cohorts drawn from the same population;** Definitely yes (low risk of bias); Participants were selected from a nationally representative survey focusing on eligible households. "We used data from four waves (2010–13) of the ENCV, a nationally representative household survey."

**Item 2; Can we be confident in the assessment of exposure;** Definitely yes (low risk of bias); Exposure (receipt of pension) was well defined and linked to clear program eligibility criteria. "Our main independent variable was a binary indicator that captured whether or not any member of a given household received Colombia Mayor benefits."

**Item 3; Can we be confident that the outcome of interest was not present at start of study;** Probably yes; Study excluded ineligible households, but baseline health conditions were not explicitly detailed. NR

**Item 4; Did the study match exposed and unexposed for all variables that are associated with the outcome of interest or did the statistical analysis adjust for these prognostic variables;** Definitely yes (low risk of bias); The study used an instrumental variable approach to adjust for confounders. "An instrumental variable approach... exploited the large variation in rollout rates across municipalities."

**Item 5; Can we be confident in the assessment of the presence or absence of prognostic factors;** Probably yes; Baseline socioeconomic factors were included, but detailed medical profiling was not reported. NR

**Item 6; Can we be confident in the assessment of outcome;** Probably yes; Self-reported health was used as the outcome, which has limitations in reliability but is valid for the study's objectives. "We used the following measures of health and health care use... self-rated general health."

**Item 7; Was the follow-up of cohorts adequate;** Probably yes; Multiple waves of data ensured longitudinal tracking, but the study did not explicitly address individual-level follow-up rates. NR

**Item 8; Were co-interventions similar between groups;** Probably no; Differences in access to healthcare and social services between regions and households likely influenced outcomes. "Access to medicines or health services in poor and rural areas is not universal."

ID: Hessel, 2018; Cohort name: Participants in the Colombia Mayor noncontributory pension program.

Item 1: Definitely yes (low risk of bias).

Item 2: Definitely yes (low risk of bias).

Item 3: Probably yes.

Item 4: Definitely yes (low risk of bias).

Item 5: Probably yes.

Item 6: Probably yes.

Item 7: Probably yes.

Item 8: Probably no.

**Second response:**

**Item 1; Was selection of exposed and non-exposed cohorts drawn from the same population;** Definitely yes (low risk of bias); The study utilized a nationally representative survey and focused on households meeting program eligibility criteria. "We used data from four waves (2010–13) of the ENCV, a nationally representative household survey."

**Item 2; Can we be confident in the assessment of exposure;** Definitely yes (low risk of bias); Exposure (pension receipt) was determined based on program records and household surveys, ensuring reliable classification. "Our main independent variable was a binary indicator that captured whether or not any member of a given household received Colombia Mayor benefits."

**Item 3; Can we be confident that the outcome of interest was not present at start of study;** Probably yes; Households without prior pension benefits were included, but individual baseline health conditions were not explicitly detailed. NR

**Item 4; Did the study match exposed and unexposed for all variables that are associated with the outcome of interest or did the statistical analysis adjust for these prognostic variables;** Definitely yes (low risk of bias); The study used an instrumental variable approach to account for confounding variables such as socioeconomic status. "An instrumental variable approach... exploited the large variation in rollout rates across municipalities."

**Item 5; Can we be confident in the assessment of the presence or absence of prognostic factors;** Probably yes; Demographic and socioeconomic characteristics were considered, but medical conditions were not thoroughly evaluated. NR

**Item 6; Can we be confident in the assessment of outcome;** Probably yes; Self-reported health outcomes were used, which are subject to reporting bias but valid for this type of study. "We used the following measures of health and health care use... self-rated general health."

**Item 7; Was the follow-up of cohorts adequate;** Probably yes; Data were collected over multiple survey waves, but individual-level follow-up rates were not specified. NR

**Item 8; Were co-interventions similar between groups;** Probably no; Access to healthcare and other resources varied across municipalities, potentially influencing the outcomes. "Access to medicines or health services in poor and rural areas is not universal."

ID: Hessel, 2018; Cohort name: Participants in the Colombia Mayor noncontributory pension program

Item 1: Definitely yes (low risk of bias).

Item 2: Definitely yes (low risk of bias).

Item 3: Probably yes.

Item 4: Definitely yes (low risk of bias).

Item 5: Probably yes.

Item 6: Probably yes.

Item 7: Probably yes.

Item 8: Probably no.

Title: Diagnostic value of Xpert MTB/RIF Ultra for osteoarticular tuberculosis

**First response:**

**Item 1; Was selection of exposed and non-exposed cohorts drawn from the same population;** Definitely yes (low risk of bias). The study population was drawn from the same referral center over the same time period, ensuring comparability. "Adults with suspected osteoarticular TB were prospectively and consecutively enrolled during June 2017 to June 2018 at Beijing Chest Hospital."

**Item 2; Can we be confident in the assessment of exposure;** Definitely yes (low risk of bias). Exposure was determined through standardized diagnostic methods, including smear microscopy, culture, Xpert, and Xpert Ultra assays. "Each pus specimen collected during operation from each patient was subjected to smear microscopy, culture, Xpert and Xpert Ultra."

**Item 3; Can we be confident that the outcome of interest was not present at start of study;** Probably yes. Patients with osteoarticular TB were prospectively followed, but the study did not explicitly report measures to ensure the absence of the outcome at baseline. NR.

**Item 4; Did the study match exposed and unexposed for all variables that are associated with the outcome of interest or did the statistical analysis adjust for these prognostic variables;** Definitely yes (low risk of bias). The study conducted stratified analyses based on smear and culture status, ensuring adjustment for key prognostic variables. "Sensitivity of Xpert Ultra was higher than Xpert among both smear-negative cases (89.38%...vs. 75.22%) and culture-negative cases (87.5%...vs. 67.5%)."

**Item 5; Can we be confident in the assessment of the presence or absence of prognostic factors;** Probably no. Key factors such as age, gender, comorbidities, and clinical features were systematically assessed and reported; however, the quality and repeatability of data collection were not explicitly validated. "High proportion of osteoarticular TB patients (25.00%) also combined with pulmonary TB in contrast with the non-osteoarticular TB patients (6.06%, P=0.015)."

**Item 6; Can we be confident in the assessment of outcome;** Definitely yes (low risk of bias). Outcomes were assessed through standardized diagnostic tests and follow-up data. "All the patients were followed up for a minimum of 6 months."

**Item 7; Was the follow-up of cohorts adequate;** Definitely yes (low risk of bias). All patients were followed up for at least 6 months, ensuring completeness of data. "All the patients were followed up for a minimum of 6 months."

**Item 8; Were co-interventions similar between groups;** Probably yes. Co-interventions were not explicitly reported, but patients were managed in a consistent clinical setting. NR.

ID: Sun, 2019; Cohort name: Patients suspected of osteoarticular tuberculosis from Beijing Chest Hospital.

Item 1: Definitely yes (low risk of bias).

Item 2: Definitely yes (low risk of bias).

Item 3: Probably yes.

Item 4: Definitely yes (low risk of bias).

Item 5: Probably no.

Item 6: Definitely yes (low risk of bias).

Item 7: Definitely yes (low risk of bias).

Item 8: Probably yes.

**Second response:**

**Item 1; Was selection of exposed and non-exposed cohorts drawn from the same population;** Definitely yes (low risk of bias). Patients were enrolled prospectively from the same referral center during the same period, ensuring consistency in the population. "Adults with suspected osteoarticular TB were prospectively and consecutively enrolled during June 2017 to June 2018 at Beijing Chest Hospital."

**Item 2; Can we be confident in the assessment of exposure;** Definitely yes (low risk of bias). Exposure to Mycobacterium tuberculosis was confirmed using standardized diagnostic methods, including smear microscopy, culture, Xpert, and Xpert Ultra. "Each pus specimen collected during operation from each patient was subjected to smear microscopy, culture, Xpert, and Xpert Ultra."

**Item 3; Can we be confident that the outcome of interest was not present at start of study;** Probably yes. The study included patients suspected of TB, but it did not explicitly confirm that none had advanced TB at baseline. NR.

**Item 4; Did the study match exposed and unexposed for all variables that are associated with the outcome of interest or did the statistical analysis adjust for these prognostic variables;** Definitely yes (low risk of bias). The study adjusted for key diagnostic statuses, including smear-positive and culture-positive conditions, and stratified performance analyses were conducted. "Sensitivity of Xpert Ultra was higher than Xpert among both smear-negative cases (89.38% vs. 75.22%) and culture-negative cases (87.5% vs. 67.5%)."

**Item 5; Can we be confident in the assessment of the presence or absence of prognostic factors;** Probably no. Prognostic factors such as age, gender, and comorbidities were systematically reported, but no explicit validation of the accuracy or repeatability of these measures was provided. "High proportion of osteoarticular TB patients (25.00%) also combined with pulmonary TB in contrast with the non-osteoarticular TB patients (6.06%, P=0.015)."​

**Item 6; Can we be confident in the assessment of outcome;** Definitely yes (low risk of bias). The outcomes were evaluated based on structured diagnostic tests and follow-up data collection over six months. "All the patients were followed up for a minimum of 6 months."

**Item 7; Was the follow-up of cohorts adequate;** Definitely yes (low risk of bias). No significant loss to follow-up was reported, and all patients were followed up for a sufficient duration. "All the patients were followed up for a minimum of 6 months."

**Item 8; Were co-interventions similar between groups;** Probably yes. While co-interventions were not explicitly described, the diagnostic and treatment processes likely followed consistent protocols in the single hospital setting. NR.

ID: Sun, 2019; Cohort name: Patients suspected of osteoarticular tuberculosis from Beijing Chest Hospital.

Item 1: Definitely yes (low risk of bias).

Item 2: Definitely yes (low risk of bias).

Item 3: Probably yes.

Item 4: Definitely yes (low risk of bias).

Item 5: Probably no.

Item 6: Definitely yes (low risk of bias).

Item 7: Definitely yes (low risk of bias).

Item 8: Probably yes.

**KimiChat**

Title: Anticholinergic Drug Use and Negative Outcomes Among the Frail Elderly Population Living in a Nursing Home

**First response:**

**Item 1; Was selection of exposed and non-exposed cohorts drawn from the same population;** Probably yes; "From February to December 2005, 1904 long-term nursing home residents, aged 65 years or older, living in 31 facilities distributed across Italy, were enrolled in the U.L.I.S.S.E. project."

**Item 2; Can we be confident in the assessment of exposure;** Probably no; The study does not provide a clear method for assessing anticholinergic drug exposure.

**Item 3; Can we be confident that the outcome of interest was not present at start of study;** Definitely yes (low risk of bias); "All participants underwent a standardized comprehensive evaluation using the Italian version of the inter Resident Assessment Instrument Minimum Data Set (version 2.0) for Nursing Home (MDSNH)..."

**Item 4; Did the study match exposed and unexposed for all variables that are associated with the outcome of interest or did the statistical analysis adjust for these prognostic variables;** Probably yes; "In multivariate analysis, a higher score in the ARS scale was associated with a greater likelihood of functional decline..."

**Item 5; Can we be confident in the assessment of the presence or absence of prognostic factors;** Probably yes; "The MDS-NH contains more than 350 data elements, including sociodemographic variables, numerous clinical items about both physical and cognitive status, as well as all clinical diagnoses..."

**Item 6; Can we be confident in the assessment of outcome;** Definitely yes (low risk of bias); "Functional decline was defined as a reduction of at least 1 point in the summary ADL score, including all the activities of daily life during follow-up..."

**Item 7; Was the follow up of cohorts adequate;** Probably yes; "Patients from 31 facilities in Italy were assessed at baseline and at 6 and 12 months..."

**Item 8; Were co-interventions similar between groups;** NR; There is no information provided about any co-interventions.

ID: Landi, 2014; Cohort name: Nursing home residents.

Item 1: Probably yes.

Item 2: Probably no.

Item 3: Definitely yes (low risk of bias).

Item 4: Probably yes.

Item 5: Probably yes.

Item 6: Definitely yes (low risk of bias).

Item 7: Probably yes.

Item 8:Probably no.

**Second response:**

**Item 1; Was selection of exposed and non-exposed cohorts drawn from the same population;** Definitely yes (low risk of bias); "From February to December 2005, 1904 long-term nursing home residents, aged 65 years or older, living in 31 facilities distributed across Italy, were enrolled in the U.L.I.S.S.E. project."

**Item 2; Can we be confident in the assessment of exposure;** Probably no; The study does not provide enough information to determine the accuracy of exposure assessment.

**Item 3; Can we be confident that the outcome of interest was not present at start of study;** Definitely yes (low risk of bias); "All participants underwent a standardized comprehensive evaluation using the Italian version of the inter Resident Assessment Instrument Minimum Data Set (version 2.0) for Nursing Home (MDSNH)..."

**Item 4; Did the study match exposed and unexposed for all variables that are associated with the outcome of interest or did the statistical analysis adjust for these prognostic variables;** Probably yes; "In multivariate analysis, a higher score in the ARS scale was associated with a greater likelihood of functional decline..."

**Item 5; Can we be confident in the assessment of the presence or absence of prognostic factors;** Probably yes; "The MDS-NH contains more than 350 data elements, including sociodemographic variables, numerous clinical items about both physical and cognitive status, as well as all clinical diagnoses."

**Item 6; Can we be confident in the assessment of outcome;** Definitely yes (low risk of bias); "Functional decline was defined as a reduction of at least 1 point in the summary ADL score, including all the activities of daily life during follow-up."

**Item 7; Was the follow up of cohorts adequate;** Probably yes; "Patients from 31 facilities in Italy were assessed at baseline and at 6 and 12 months by trained personnel."

**Item 8; Were co-interventions similar between groups;** NR; There is no information provided about co-interventions in the study.

ID: Landi, 2014; Cohort name: Nursing home residents.

Item 1: Definitely yes (low risk of bias).

Item 2: Probably no.

Item 3: Definitely yes (low risk of bias).

Item 4: Probably yes.

Item 5: Probably yes.

Item 6: Definitely yes (low risk of bias).

Item 7: Probably yes.

Item 8: Probably no.

Title: Chronic Exposure to Anticholinergic Medications Adversely Affects the Course of Alzheimer Disease

**First response:**

**Item 1; Was selection of exposed and non-exposed cohorts drawn from the same population;** Probably yes; "The study population (N=69) was divided into two groups: those receiving one or more concomitant medications with significant anticholinergic properties (N=16) and those receiving no concomitant medications with anticholinergic properties (N=53)."

**Item 2; Can we be confident in the assessment of exposure;** Probably yes; "All patients were examined annually with standard neuropsychologic tests and received the cholinesterase inhibitor donepezil hydrochloride at a dose of 10 mg/day."

**Item 3; Can we be confident that the outcome of interest was not present at start of study;** Definitely yes (low risk of bias); "There were no significant differences between the individuals receiving no anticholinergic medications and those receiving one or more anticholinergic medications with regard to baseline MMSE scores."

**Item 4; Did the study match exposed and unexposed for all variables that are associated with the outcome of interest or did the statistical analysis adjust for these prognostic variables;** Probably no; The study did not match patients for all variables associated with the outcome of interest; "There were no significant differences between the individuals receiving no anticholinergic medications and those receiving one or more anticholinergic medications..."

**Item 5; Can we be confident in the assessment of the presence or absence of prognostic factors;** Probably yes; "The primary measure in this study is the MMSE scale, which is used to determine the severity of the patient’s cognitive impairment."

**Item 6; Can we be confident in the assessment of outcome;** Definitely yes (low risk of bias); "MMSE scores were collected at the baseline visit into the ADC program and at the subsequent two follow-up visits."

**Item 7; Was the follow up of cohorts adequate;** Probably yes; "Patients are then followed annually with standard neuropsychological tests."

**Item 8; Were co-interventions similar between groups;** NR; No information provided about co-interventions.

ID: Lu, 2003; Cohort name: Alzheimer's disease patients.

Item 1: Probably yes.

Item 2: Probably yes.

Item 3: Definitely yes (low risk of bias).

Item 4: Probably no.

Item 5: Probably yes.

Item 6: Definitely yes (low risk of bias).

Item 7: Probably yes.

Item 8: Probably no.

**Second response:**

**Item 1; Was selection of exposed and non-exposed cohorts drawn from the same population;** Probably yes; "The study population (N=69) was divided into two groups: those receiving one or more concomitant medications with significant anticholinergic properties (N=16) and those receiving no concomitant medications with anticholinergic properties (N=53)."

**Item 2; Can we be confident in the assessment of exposure;** Probably yes; The study used medication lists to determine exposure to anticholinergic medications; "Anticholinergic medications were identified from previously published lists, on the basis of antimuscarinic activity in a radioreceptor assay."

**Item 3; Can we be confident that the outcome of interest was not present at start of study;** Definitely yes (low risk of bias); The study assessed patients annually with neuropsychologic tests, implying the outcome was not present at the start; "All patients were examined annually with standard neuropsychologic tests..."

**Item 4; Did the study match exposed and unexposed for all variables that are associated with the outcome of interest or did the statistical analysis adjust for these prognostic variables;** Probably no; The study did not match patients for all variables associated with the outcome of interest; "There were no significant differences between the individuals receiving no anticholinergic medications and those receiving one or more anticholinergic medications..."

**Item 5; Can we be confident in the assessment of the presence or absence of prognostic factors;** Probably no; The study does not provide enough information to assess the presence or absence of prognostic factors; NR

**Item 6; Can we be confident in the assessment of outcome;** Definitely yes (low risk of bias); The study used the MMSE scale, a validated measure of cognitive impairment, to assess outcomes; "The primary measure in this study is the MMSE scale."

**Item 7; Was the follow up of cohorts adequate;** Probably yes; The study does not provide enough information to assess the adequacy of follow-up; "MMSE scores were collected at the baseline visit into the ADC program and at the subsequent two follow-up visits."

**Item 8; Were co-interventions similar between groups;** Probably no; The study does not provide information on co-interventions; NR

ID: Lu, 2003; Cohort name: Alzheimer's disease patients with and without anticholinergic medications.

Item 1: Probably yes.

Item 2: Probably yes.

Item 3: Definitely yes (low risk of bias).

Item 4: Probably no.

Item 5: Probably no.

Item 6: Definitely yes (low risk of bias).

Item 7: Probably yes.

Item 8: Probably no.

Title: Prognostic factors associated with mortality risk and disease progression in 639 critically ill patients with COVID-19 in Europe: Initial report of the international RISC-19-ICU prospective observational cohort

**First response:**

**Item 1：Was selection of exposed and non-exposed cohorts drawn from the same population;** Probably yes; The study populations appear to be drawn from the same population of critically ill COVID-19 patients in Europe; “Following the critical developments in ICUs in regions experiencing early inception of the pandemic, the European-based, international RIsk Stratification in COVID-19 patients in the Intensive Care Unit (RISC-19-ICU) registry was created to provide near real-time assessment of patients developing critical illness due to COVID-19.”

**Item 2; Can we be confident in the assessment of exposure;** Probably no; The study does not describe how exposure information was obtained; NR.

**Item 3; Can we be confident that the outcome of interest was not present at start of study;** Definitely yes (low risk of bias); The study excluded patients who had the outcome of interest at baseline; “Inclusion criteria for the RISC-19-ICU registry were (I) a laboratory confirmed SARS-CoV-2 infection by nucleic acid amplification according to the WHO-issued testing guidelines, and (II) severe manifestation of COVID-19 requiring treatment in an ICU or intermediate care unit.”

**Item 4; Did the study match exposed and unexposed for all variables that are associated with the outcome of interest or did the statistical analysis adjust for these prognostic variables;** Probably yes; The study attempted to adjust for prognostic variables; “On a multivariable Cox proportional-hazard regression model at admission, creatinine, D-dimer, lactate, potassium, P/F-ratio, alveolar-arterial gradient, and ischemic heart disease were independently associated with ICU-mortality.”

**Item 5; Can we be confident in the assessment of the presence or absence of prognostic factors;** Probably yes; Prognostic factors were obtained through a standardized dataset; “A standardized dataset was prospectively collected during the ongoing COVID-19 pandemic for all critically ill COVID-19 patients admitted to the collaborating centres.”

**Item 6; Can we be confident in the assessment of outcome;** Definitely yes (low risk of bias); The outcome indicator is based on objective data; “As of April 22, 2020, 639 critically ill patients with confirmed SARS-CoV-2 infection were included in the RISC-19-ICU registry.”

**Item 7; Was the follow up of cohorts adequate;** Probably yes; The study followed patients for a defined period; “Data were collected on the day of ICU admission, and on days one, two, three, five and seven thereafter.”

**Item 8; Were co-interventions similar between groups;** Probably yes; The study does not suggest that co-interventions differed between groups; “Off-label therapies were prescribed in 265 (67%) patients...”

ID: Wendel Garcia, 2020; Cohort name: RISC-19-ICU registry cohort.

Item 1: Probably yes.

Item 2: Probably no.

Item 3: Definitely yes (low risk of bias).

Item 4: Probably yes.

Item 5: Probably yes.

Item 6: Definitely yes (low risk of bias).

Item 7: Probably yes.

Item 8: Probably yes.

**Second response:**

**Item 1; Was selection of exposed and non-exposed cohorts drawn from the same population;** Probably yes; "Following the critical developments in ICUs in regions experiencing early inception of the pandemic, the European-based, international RIsk Stratification in COVID-19 patients in the Intensive Care Unit (RISC-19-ICU) registry was created to provide near real-time assessment of patients developing critical illness due to COVID-19."

**Item 2; Can we be confident in the assessment of exposure;** Probably no; The study does not provide enough information to determine how exposure was assessed.

**Item 3; Can we be confident that the outcome of interest was not present at start of study;** Definitely yes (low risk of bias); "Inclusion criteria for the RISC-19-ICU registry were (I) a laboratory confirmed SARS-CoV-2 infection by nucleic acid amplification according to the WHO-issued testing guidelines [10], and (II) severe manifestation of COVID-19 requiring treatment in an ICU or intermediate care unit."

**Item 4; Did the study match exposed and unexposed for all variables that are associated with the outcome of interest or did the statistical analysis adjust for these prognostic variables;** Probably yes; "On a multivariable Cox proportional-hazard regression model at admission, creatinine, D-dimer, lactate, potassium, P/F-ratio, alveolar-arterial gradient, and ischemic heart disease were independently associated with ICU-mortality."

**Item 5; Can we be confident in the assessment of the presence or absence of prognostic factors;** Probably yes; "A standardized dataset was prospectively collected during the ongoing COVID-19 pandemic for all critically ill COVID-19 patients admitted to the collaborating centres."

**Item 6; Can we be confident in the assessment of outcome;** Definitely yes (low risk of bias); "As of April 22, 2020, 639 critically ill patients with confirmed SARS-CoV-2 infection were included in the RISC-19-ICU registry."

**Item 7; Was the follow up of cohorts adequate;** Probably yes; "Data were collected on the day of ICU admission, and on days one, two, three, five and seven thereafter."

**Item 8; Were co-interventions similar between groups;** Probably yes; The study does not provide enough information to determine whether co-interventions were similar between groups.

ID: Wendel Garcia, 2020; Cohort name: RISC-19-ICU registry cohort.

Item 1: Probably yes.

Item 2: Probably no.

Item 3: Definitely yes (low risk of bias).

Item 4: Probably yes.

Item 5: Probably yes.

Item 6: Definitely yes (low risk of bias).

Item 7: Probably yes.

Item 8: Probably yes.

Title: Clinical characteristics and risk factors associated with COVID-19 disease severity in patients with cancer in Wuhan, China: a multicentre, retrospective, cohort study

**First response:**

**Item 1; Was selection of exposed and non-exposed cohorts drawn from the same population;** Probably no; The text does not provide enough information to determine if the cohorts were drawn from the same population; "Enrolled patients were statistically matched (2:1) with patients admitted with COVID-19 who did not have cancer with propensity score on the basis of age, sex, and comorbidities."

**Item 2; Can we be confident in the assessment of exposure;** Probably no; The study does not describe how exposure to COVID-19 was determined; NR

**Item 3; Can we be confident that the outcome of interest was not present at start of study;** Definitely yes (low risk of bias); The study included patients with laboratory-confirmed COVID-19, implying the outcome was not present at the start; "we included all adult patients (aged ≥18 years) with any type of malignant solid tumours and haematological malignancy who were admitted to nine hospitals in Wuhan, China, with laboratory-confirmed COVID-19 between Jan 13 and March 18, 2020."

**Item 4; Did the study match exposed and unexposed for all variables that are associated with the outcome of interest or did the statistical analysis adjust for these prognostic variables;** Probably yes; The study matched patients on age, sex, and comorbidities, but it is unclear if all variables associated with the outcome were considered; "Enrolled patients were statistically matched (2:1) with patients admitted with COVID-19 who did not have cancer with propensity score on the basis of age, sex, and comorbidities."

**Item 5; Can we be confident in the assessment of the presence or absence of prognostic factors;** Probably yes; Data was collected from electronic medical records and verified by physicians, suggesting a reliable assessment of prognostic factors; "Epidemiological, clinical, laboratory, and radiological data of all patients with laboratory-confirmed SARS-CoV-2 were obtained with data collection forms from the electronic medical records..."

**Item 6; Can we be confident in the assessment of outcome;** Probably yes; Outcomes were defined based on WHO guidelines and the Seventh Revised Trial Version of the COVID-19 Diagnosis and Treatment Guidance of China; "We defined disease severity according to WHO guidelines..."

**Item 7; Was the follow up of cohorts adequate;** Probably no; The study does not provide enough information to assess the adequacy of follow-up; NR

**Item 8; Were co-interventions similar between groups;** Probably no; The study does not provide information on co-interventions; NR

ID: Tian, 2020; Cohort name: COVID-19 patients with cancer and without cancer in Wuhan.

Item 1: Probably no.

Item 2: Probably no.

Item 3: Definitely yes (low risk of bias).

Item 4: Probably yes.

Item 5: Probably yes.

Item 6: Probably yes.

Item 7: Probably no.

Item 8: Probably no.

**Second response:**

**Item 1; Was selection of exposed and non-exposed cohorts drawn from the same population;** Probably no; The text does not provide explicit evidence that the cohorts were drawn from the exact same population or administrative database; "Enrolled patients were statistically matched (2:1) with patients admitted with COVID-19 who did not have cancer with propensity score on the basis of age, sex, and comorbidities."

**Item 2; Can we be confident in the assessment of exposure;** Probably yes; The study used laboratory-confirmed COVID-19 for inclusion, indicating a reliable method of determining exposure; "we included all adult patients (aged ≥18 years) with any type of malignant solid tumours and haematological malignancy who were admitted to nine hospitals in Wuhan, China, with laboratory-confirmed COVID-19."

**Item 3; Can we be confident that the outcome of interest was not present at start of study;** Definitely yes (low risk of bias); The study included patients with confirmed COVID-19, implying the outcome (COVID-19) was not present at baseline; "In this multicentre, retrospective, cohort study, we included all adult patients (aged ≥18 years)..."

**Item 4; Did the study match exposed and unexposed for all variables that are associated with the outcome of interest or did the statistical analysis adjust for these prognostic variables;** Probably yes; The study used propensity score matching to adjust for potential confounders; "Enrolled patients were statistically matched (2:1)..."

**Item 5; Can we be confident in the assessment of the presence or absence of prognostic factors;** Probably yes; Data was collected from electronic medical records and verified by physicians, suggesting a reliable assessment of prognostic factors; "Epidemiological, clinical, laboratory, and radiological data of all patients with laboratory-confirmed SARS-CoV-2 were obtained with data collection forms from the electronic medical records..."

**Item 6; Can we be confident in the assessment of outcome;** Probably yes; Outcomes were defined based on WHO guidelines and the Seventh Revised Trial Version of the COVID-19 Diagnosis and Treatment Guidance of China; "We defined disease severity according to WHO guidelines..."

**Item 7; Was the follow up of cohorts adequate;** Probably no; The study does not provide explicit information about the follow-up rate or completeness of follow-up; NR.

**Item 8; Were co-interventions similar between groups;** Probably yes; The study does not mention any differences in co-interventions between groups, implying they were similar; "These permissions are granted for free by Elsevier for as long as the COVID-19 resource centre remains active."

ID: Tian, 2020; Cohort name: Cancer patients with COVID-19 and cancer-free patients with COVID-19 in Wuhan.

Item 1: Probably no.

Item 2: Probably yes.

Item 3: Definitely yes (low risk of bias).

Item 4: Probably yes.

Item 5: Probably yes.

Item 6: Probably yes.

Item 7: Probably no.

Item 8: Probably yes.

Title: Cardiac Troponin I Is an Independent Predictor for Mortality in Hospitalized Patients With COVID-19

**First response:**

**Item 1; Was selection of exposed and non-exposed cohorts drawn from the same population;** Definitely yes (low risk of bias); "Patients with laboratory-confirmed COVID-19 admitted to Union Hospital (West Campus), Huazhong University of Science and Technology from January 12 to March 12, 2020, were enrolled..."

**Item 2; Can we be confident in the assessment of exposure;** Definitely yes (low risk of bias); Exposure was determined by laboratory confirmation of COVID-19; "laboratory-confirmed COVID-19..."

**Item 3; Can we be confident that the outcome of interest was not present at start of study;** Definitely yes (low risk of bias); The study defined the outcome of interest (mortality) at the beginning of the study; "The primary composite end point was all-cause death..."

**Item 4; Did the study match exposed and unexposed for all variables that are associated with the outcome of interest or did the statistical analysis adjust for these prognostic variables;** Probably yes; The study used multivariable logistic regression to adjust for potential confounders; "multivariable logistic regression models (backward elimination) were applied..."

**Item 5; Can we be confident in the assessment of the presence or absence of prognostic factors;** Probably yes; Prognostic factors were obtained from laboratory tests and medical records; "The data of laboratory and imaging tests performed for the first time after admission were used for analysis..."

**Item 6; Can we be confident in the assessment of outcome;** Definitely yes (low risk of bias); The outcome assessment was based on objective measures (discharge or death); "the included patients were assigned to 1 of 2 groups according to clinical outcomes: the discharged group and the nonsurvivor group..."

**Item 7; Was the follow up of cohorts adequate;** Probably no; The study did not provide information on the follow-up rate or completeness; NR.

**Item 8; Were co-interventions similar between groups;** Probably yes; The study did not mention any differences in co-interventions between groups; "This study was approved by the ethics committee of Union Hospital..."

ID: Nie, 2020; Cohort name: Hospitalized COVID-19 patients with and without cardiac injury.

Item 1: Definitely yes (low risk of bias).

Item 2: Definitely yes (low risk of bias).

Item 3: Definitely yes (low risk of bias).

Item 4: Probably yes.

Item 5: Probably yes.

Item 6: Definitely yes (low risk of bias).

Item 7: Probably no.

Item 8: Probably yes.

**Second response:**

**Item 1; Was selection of exposed and non-exposed cohorts drawn from the same population;** Definitely yes (low risk of bias); "Patients with laboratory-confirmed COVID-19 admitted to Union Hospital (West Campus), Huazhong University of Science and Technology from January 12 to March 12, 2020, were enrolled..."

**Item 2; Can we be confident in the assessment of exposure;** Definitely yes (low risk of bias); Exposure was determined by laboratory confirmation of COVID-19; "laboratory-confirmed COVID-19..."

**Item 3; Can we be confident that the outcome of interest was not present at start of study;** Definitely yes (low risk of bias); The study defined the outcome of interest (mortality) at the beginning of the study; "The primary composite end point was all-cause death..."

**Item 4; Did the study match exposed and unexposed for all variables that are associated with the outcome of interest or did the statistical analysis adjust for these prognostic variables;** Probably yes; The study used multivariable logistic regression to adjust for potential confounders; "multivariable logistic regression models (backward elimination) were applied..."

**Item 5; Can we be confident in the assessment of the presence or absence of prognostic factors;** Probably yes; Prognostic factors were obtained from medical records and laboratory tests at admission; "The data of laboratory and imaging tests performed for the first time after admission were used for analysis..."

**Item 6; Can we be confident in the assessment of outcome;** Definitely yes (low risk of bias); The outcome assessment was based on hospital records and confirmed by the treating physicians; "One hundred eleven patients died during hospitalization and 200 were discharged."

**Item 7; Was the follow up of cohorts adequate;** Probably no; The study did not provide information on the follow-up rate or completeness; NR.

**Item 8; Were co-interventions similar between groups;** Probably yes; The study did not mention any differences in co-interventions between groups; "This study is limited by selection bias based on cTnI measurement..."

ID: Nie, 2020; Cohort name: Hospitalized COVID-19 patients with and without cardiac injury.

Item 1:Definitely yes (low risk of bias).

Item 2: Definitely yes (low risk of bias).

Item 3: Definitely yes (low risk of bias).

Item 4: Probably yes.

Item 5: Probably yes.

Item 6: Definitely yes (low risk of bias).

Item 7: Probably no.

Item 8: Probably yes.

Title: Clinical impact of pre-admission antithrombotic therapy in hospitalized patients with COVID-19: A multicenter observational study

**First response:**

**Item 1; Was selection of exposed and non-exposed cohorts drawn from the same population;** Probably no; The study included patients from five Italian hospitals, but it is unclear if they are from the same population; "We enrolled 192 consecutive patients with laboratory-conﬁrmed COVID-19 admitted to emergency department of ﬁve Italian hospitals."

**Item 2; Can we be confident in the assessment of exposure;** Definitely yes (low risk of bias); Exposure was determined by medical history at admission; "At admission, all patients underwent medical history, physical examination and laboratory evaluation."

**Item 3; Can we be confident that the outcome of interest was not present at start of study;** Definitely yes (low risk of bias); The study defined ARDS according to the Berlin definition at admission; "ARDS diagnosis was deﬁned according to the Berlin deﬁnition."

**Item 4; Did the study match exposed and unexposed for all variables that are associated with the outcome of interest or did the statistical analysis adjust for these prognostic variables;** Probably yes; The study used propensity score weighting to adjust for potential selection bias; "We used propensity score weighting to account for potential selection bias in treatment assignment between the two study groups."

**Item 5; Can we be confident in the assessment of the presence or absence of prognostic factors;** Probably yes; Prognostic factors were obtained from medical records and laboratory tests at admission; "At admission, all patients underwent medical history, physical examination and laboratory evaluation."

**Item 6; Can we be confident in the assessment of outcome;** Definitely yes (low risk of bias); The outcome assessment was based on hospital records and confirmed by the treating physicians; "Thirty-ﬁve patients (18.5 %) died during the hospitalization."

**Item 7; Was the follow up of cohorts adequate;** Probably no; The study did not provide information on the follow-up rate or completeness; NR.

**Item 8; Were co-interventions similar between groups;** Probably yes; The study did not mention any differences in co-interventions between groups; "Pre-admission antithrombotic therapy, both antiplatelet and anticoagulant, does not seem to show a protective eﬀect in severe forms of COVID-19..."

ID: Russo, 2020; Cohort name: COVID-19 patients with and without ARDS at admission.

Item 1: Probably no.

Item 2: Definitely yes (low risk of bias).

Item 3: Definitely yes (low risk of bias).

Item 4: Probably yes.

Item 5: Probably yes.

Item 6: Definitely yes (low risk of bias).

Item 7: Probably no.

Item 8: Probably yes.

**Second response:**

**Item 1; Was selection of exposed and non-exposed cohorts drawn from the same population;** Probably no; The study included patients from five different hospitals, but it is unclear if they are from the same population; "We enrolled 192 consecutive patients with laboratory-conﬁrmed COVID-19 admitted to emergency department of ﬁve Italian hospitals."

**Item 2; Can we be confident in the assessment of exposure;** Definitely yes (low risk of bias); Exposure was determined by medical history at admission; "At admission, all patients underwent medical history, physical examination and laboratory evaluation."

**Item 3; Can we be confident that the outcome of interest was not present at start of study;** Definitely yes (low risk of bias); The study defined ARDS according to the Berlin definition at admission; "ARDS diagnosis was deﬁned according to the Berlin deﬁnition."

**Item 4; Did the study match exposed and unexposed for all variables that are associated with the outcome of interest or did the statistical analysis adjust for these prognostic variables;** Probably yes; The study used propensity score weighting to adjust for potential confounders; "Propensity score weighting adjusted regression analysis was performed to assess the risk ARDS at admission, and death during hospitalization..."

**Item 5; Can we be confident in the assessment of the presence or absence of prognostic factors;** Probably yes; Prognostic factors were obtained from medical records and laboratory tests at admission; "The data of laboratory and imaging tests performed for the first time after admission were used for analysis..."

**Item 6; Can we be confident in the assessment of outcome;** Definitely yes (low risk of bias); The outcome assessment was based on hospital records and confirmed by the treating physicians; "Thirty-ﬁve patients (18.5 %) died during the hospitalization."

**Item 7; Was the follow up of cohorts adequate;** Probably no; The study did not provide information on the follow-up rate or completeness; NR.

**Item 8; Were co-interventions similar between groups;** Probably yes; The study did not mention any differences in co-interventions between groups; "Pre-admission antithrombotic therapy, both antiplatelet and anticoagulant, does not seem to show a protective eﬀect in severe forms of COVID-19..."

ID: Russo, 2020; Cohort name: COVID-19 patients with and without ARDS at admission.

Item 1: Probably no.

Item 2: Definitely yes (low risk of bias).

Item 3: Definitely yes (low risk of bias).

Item 4: Probably yes.

Item 5: Probably yes.

Item 6: Definitely yes (low risk of bias).

Item 7: Probably no.

Item 8: Probably yes.

Title: The Impact of a School-Based Water, Sanitation, and Hygiene Program on Absenteeism, Diarrhea, and Respiratory Infection: A Matched–Control Trial in Mali

**First response:**

**Item 1; Was selection of exposed and non-exposed cohorts drawn from the same population;** Probably yes; The study aimed to match beneficiary schools with comparison schools based on enrollment size and WASH characteristics; "Beneficiary schools (N = 100) were selected for the evaluation from DCIM program records and matched with 100 comparison schools with similar enrollment size and WASH characteristics within the same educational districts."

**Item 2; Can we be confident in the assessment of exposure;** Definitely yes (low risk of bias); Exposure was determined by the implementation of the Dubai Cares Initiative in Mali (DCIM) program; "We evaluated a school-based WASH program, known as the Dubai Cares Initiative in Mali (DCIM) for WASH in schools..."

**Item 3; Can we be confident that the outcome of interest was not present at start of study;** Probably yes; The study did not provide explicit evidence that the outcomes were absent at baseline, but it was a controlled trial implying baseline equivalence; "We used a matched–control, longitudinal design."

**Item 4; Did the study match exposed and unexposed for all variables that are associated with the outcome of interest or did the statistical analysis adjust for these prognostic variables;** Probably yes; The study used propensity score matching to adjust for potential confounders; "We used propensity score matching to account for potential selection bias in treatment assignment between the two study groups..."

**Item 5; Can we be confident in the assessment of the presence or absence of prognostic factors;** Probably yes; Prognostic factors were obtained from school records and pupil interviews; "Data collection included roll calls to assess absenteeism and interviews with a subset of pupils to assess recent absence and disease symptoms."

**Item 6; Can we be confident in the assessment of outcome;** Definitely yes (low risk of bias); The outcome assessment was based on school records and pupil interviews; "The odds of pupils being absent at roll call were 23% higher in beneficiary schools than in comparison schools..."

**Item 7; Was the follow up of cohorts adequate;** Probably yes; The study involved multiple follow-up visits to the schools; "Enumerators visited each school every 6–8 weeks between January 2013 and May 2014..."

**Item 8; Were co-interventions similar between groups;** Probably yes; The study did not mention any differences in co-interventions between groups; "No further financial or technical assistance was provided to schools by the program or the government once the intervention activities were completed..."

ID: Trinies, 2016; Cohort name: Students in beneficiary schools and matched comparison schools in Mali.

Item 1: Probably yes.

Item 2: Definitely yes (low risk of bias).

Item 3: Probably yes.

Item 4: Probably yes.

Item 5: Probably yes.

Item 6: Definitely yes (low risk of bias).

Item 7: Probably yes.

Item 8: Probably yes.

**Second response:**

**Item 1; Was selection of exposed and non-exposed cohorts drawn from the same population;** Probably yes; The study used a matched–control design, suggesting that the cohorts were drawn from the same population; "Beneficiary schools (N = 100) were selected for the evaluation from DCIM program records and matched with 100 comparison schools with similar enrollment size and WASH characteristics within the same educational districts."

**Item 2; Can we be confident in the assessment of exposure;** Definitely yes (low risk of bias); Exposure was clearly defined by participation in the WASH program; "We evaluated a school-based WASH program, known as the Dubai Cares Initiative in Mali (DCIM) for WASH in schools, implemented by..."

**Item 3; Can we be confident that the outcome of interest was not present at start of study;** Probably no; The study did not provide baseline data on the outcomes of interest; "Baseline data on attendance and health outcomes were not collected, as intervention activities had begun before the evaluation period."

**Item 4; Did the study match exposed and unexposed for all variables that are associated with the outcome of interest or did the statistical analysis adjust for these prognostic variables;** Definitely yes (low risk of bias); The study used multivariate logistic regression to adjust for potential confounders; "We used intention-to-treat analyses utilizing mixed-effects logistic regression models that compared beneficiary schools to the matched comparison schools..."

**Item 5; Can we be confident in the assessment of the presence or absence of prognostic factors;** Probably yes; The study involved training of enumerators and used standardized data collection tools; "Enumerators participated in a 1-week training that included data collection procedures, ethics, minimization of bias, and use of android devices and the ODK data collection software."

**Item 6; Can we be confident in the assessment of outcome;** Definitely yes (low risk of bias); Outcomes were objectively measured through roll call and pupil interviews; "Data were collected electronically on Android-enabled devices using Open Data Kit (ODK) by a team of experienced enumerators."

**Item 7; Was the follow up of cohorts adequate;** Probably yes; The study involved multiple follow-up visits to the schools; "Enumerators visited each school every 6–8 weeks between January 2013 and May 2014..."

**Item 8; Were co-interventions similar between groups;** Probably yes; The study design aimed to ensure that any co-interventions were similar between groups; "The DCIM program delivered a comprehensive WASH package to 916 primary and secondary schools..."

ID: Trinies, 2016; Cohort name: beneficiary schools and comparison schools.

Item 1: Probably yes.

Item 2: Definitely yes (low risk of bias).

Item 3: Probably no.

Item 4: Definitely yes (low risk of bias).

Item 5: Probably yes.

Item 6: Definitely yes (low risk of bias).

Item 7: Probably yes.

Item 8: Probably yes.

Title: The Use of Magnetic Resonance Imaging in the Diagnosis of Suspected Appendicitis in Pregnancy Shortened Length of Stay Without Increase in Hospital Charges

**First response:**

**Item 1; Was selection of exposed and non-exposed cohorts drawn from the same population;** Probably yes; The study included all pregnant patients with suspected appendicitis from a single center; "Retrospective review at a university tertiary referral center of all pregnant patients seen with abdominal pain and suspected appendicitis who were followed up through delivery during an 11-year period."

**Item 2; Can we be confident in the assessment of exposure;** Definitely yes (low risk of bias); Exposure was determined by whether patients underwent MR imaging; "Thirty-one patients underwent MR imaging."

**Item 3; Can we be confident that the outcome of interest was not present at start of study;** Definitely yes (low risk of bias); The study outcome was defined as length of hospital stay, complications, and hospital charges; "To determine whether magnetic resonance (MR) imaging in pregnant patients with suspected appendicitis improves outcomes, minimizes length of stay (LOS), and lowers hospital charges."

**Item 4; Did the study match exposed and unexposed for all variables that are associated with the outcome of interest or did the statistical analysis adjust for these prognostic variables;** Probably yes; The study used multivariable analysis to adjust for potential confounders; "On multivariable analysis, the receipt of MR imaging (P < .001) and the absence of operative intervention (P = .001) were associated with shorter LOS."

**Item 5; Can we be confident in the assessment of the presence or absence of prognostic factors;** Probably yes; Prognostic factors were obtained from medical records; "Data were analyzed using commercially available software (SAS version 9.3; SAS Institute Inc)."

**Item 6; Can we be confident in the assessment of outcome;** Definitely yes (low risk of bias); The outcome assessment was based on objective hospital records and charges; "Patients in the MR imaging group were more frequently discharged from the emergency department (OR, 0.35; 95% CI, 0.13-0.94; P = .04) and had shorter LOS (33.7 vs 64.8 hours, P < .001)."

**Item 7; Was the follow up of cohorts adequate;** Probably yes; The study followed patients from the time of presentation through delivery; "who were followed up through delivery during an 11-year period."

**Item 8; Were co-interventions similar between groups;** Probably yes; The study did not mention any differences in co-interventions between groups; "Magnetic resonance imaging in pregnant patients with suspected appendicitis does not affect clinical outcomes or hospital charges."

ID: Fonseca, 2014; Cohort name: Pregnant patients with suspected appendicitis who underwent MR imaging vs. those who did not.

Item 1: Probably yes.

Item 2: Definitely yes (low risk of bias).

Item 3: Definitely yes (low risk of bias).

Item 4: Probably yes.

Item 5: Probably yes.

Item 6: Definitely yes (low risk of bias).

Item 7: Probably yes.

Item 8: Probably yes.

**Second response:**

**Item 1; Was selection of exposed and non-exposed cohorts drawn from the same population;** Probably yes; The study included all pregnant patients with suspected appendicitis from a single tertiary care center, suggesting a single population source; "We conducted a retrospective review at a university tertiary referral center of all pregnant patients seen with abdominal pain and suspected appendicitis..."

**Item 2; Can we be confident in the assessment of exposure;** Definitely yes (low risk of bias); The use of MR imaging as an exposure was clearly defined and consistently applied; "Thirty-one patients underwent MR imaging."

**Item 3; Can we be confident that the outcome of interest was not present at start of study;** Probably yes; The study design implies that the outcomes (operations, hospital stays, etc.) were measured from the start of the clinical encounter; "Retrospective review at a university tertiary referral center..."

**Item 4; Did the study match exposed and unexposed for all variables that are associated with the outcome of interest or did the statistical analysis adjust for these prognostic variables;** Probably yes; The study used multivariable analysis to adjust for potential confounders; "On multivariable analysis, the receipt of MR imaging (P < .001) and the absence of operative intervention (P = .001) were associated with shorter LOS."

**Item 5; Can we be confident in the assessment of the presence or absence of prognostic factors;** Probably yes; The study used medical records and imaging results to determine the presence or absence of appendicitis, suggesting reliable data; "The diagnosis of appendicitis was made by surgical impression in the operative report and pathology confirmation."

**Item 6; Can we be confident in the assessment of outcome;** Definitely yes (low risk of bias); Outcomes were objectively assessed through hospital records and standardized measures; "MAIN OUTCOMES AND MEASURES Time to operation, LOS, complications, nontherapeutic exploration, fetal outcomes, and hospital charges."

**Item 7; Was the follow up of cohorts adequate;** Definitely yes (low risk of bias); The study followed patients through delivery, indicating complete follow-up; "followed up through delivery during an 11-year period."

**Item 8; Were co-interventions similar between groups;** Probably yes; The study suggests that management protocols were consistent across groups; "This is especially true in the presence of negative or inconclusive ultrasonography(US)."

ID: Fonseca, 2014; Cohort name: Pregnant patients with suspected appendicitis. Overall: Low risk

Item 1: Probably yes.

Item 2: Definitely yes (low risk of bias).

Item 3: Probably yes.

Item 4: Probably yes.

Item 5: Probably yes.

Item 6: Definitely yes (low risk of bias).

Item 7: Definitely yes (low risk of bias).

Item 8: Probably yes.

Title: MRI vs. Ultrasound for Suspected Appendicitis During Pregnancy

**First response:**

**Item 1; Was selection of exposed and non-exposed cohorts drawn from the same population;** Probably yes; The study included pregnant patients with suspected appendicitis who underwent both US and MRI, suggesting a single population source; "A total of 33 pregnant patients with suspected appendicitis underwent US and MRI."

**Item 2; Can we be confident in the assessment of exposure;** Definitely yes (low risk of bias); The use of MRI and US as diagnostic tools was clearly defined and applied; "A total of 33 pregnant patients with suspected appendicitis underwent US and MRI."

**Item 3; Can we be confident that the outcome of interest was not present at start of study;** Definitely yes (low risk of bias); The study design implies that the outcomes were measured from the start of the clinical encounter; "The original imaging reports generated at the time of presentation were used for data analysis."

**Item 4; Did the study match exposed and unexposed for all variables that are associated with the outcome of interest or did the statistical analysis adjust for these prognostic variables;** Probably yes; The study used medical record review to confirm the absence or presence of appendicitis, suggesting an effort to control for confounding factors; "When surgery was not performed, a medical record review was performed."

**Item 5; Can we be confident in the assessment of the presence or absence of prognostic factors;** Probably yes; The study used pathology reports and medical record reviews for confirmation, suggesting reliable data; "Pathology reports were used for disease confirmation in patients who underwent appendectomy."

**Item 6; Can we be confident in the assessment of outcome;** Definitely yes (low risk of bias); Outcomes were objectively assessed through pathology reports and medical record reviews; "The sensitivity, speciﬁcity, PPV, and NPV were calculated for US and MRI in the diagnosis of appendicitis."

**Item 7; Was the follow up of cohorts adequate;** Probably yes; The study followed patients through definitive diagnoses, suggesting adequate follow-up; "In 13 patients, a normal appendix was diagnosed at MRI, none of whom had appendicitis."

**Item 8; Were co-interventions similar between groups;** Probably yes; The study suggests that imaging techniques were applied similarly across groups; "A total of 33 pregnant patients with suspected appendicitis underwent US and MRI."

ID: Israel, 2008; Cohort name: Pregnant patients with suspected appendicitis who underwent both US and MRI.

Item 1: Probably yes.

Item 2: Definitely yes (low risk of bias).

Item 3: Definitely yes (low risk of bias).

Item 4: Probably yes.

Item 5: Probably yes.

Item 6: Definitely yes (low risk of bias).

Item 7: Probably yes.

Item 8: Probably yes.

**Second response:**

**Item 1; Was selection of exposed and non-exposed cohorts drawn from the same population;** Probably yes; The study included pregnant patients with suspected appendicitis who underwent both US and MRI, suggesting a single population source; "A total of 33 pregnant patients with suspected appendicitis underwent US and MRI."

**Item 2; Can we be confident in the assessment of exposure;** Definitely yes (low risk of bias); The use of MRI and US as diagnostic tools was clearly defined and applied; "The original imaging reports generated at the time of presentation were used for data analysis."

**Item 3; Can we be confident that the outcome of interest was not present at start of study;** Probably yes; The study design implies that the outcomes were measured from the start of the clinical encounter; "Pathology reports were used for disease confirmation in patients who underwent appendectomy."

**Item 4; Did the study match exposed and unexposed for all variables that are associated with the outcome of interest or did the statistical analysis adjust for these prognostic variables;** Probably yes; The study used medical record review to confirm the absence or presence of appendicitis, suggesting an effort to control for confounding factors; "When surgery was not performed, a medical record review was performed."

**Item 5; Can we be confident in the assessment of the presence or absence of prognostic factors;** Probably yes; The study used a combination of imaging techniques and clinical follow-up for confirmation, suggesting reliable data; "The sensitivity, speciﬁcity, PPV, and NPV were calculated for US and MRI in the diagnosis of appendicitis."

**Item 6; Can we be confident in the assessment of outcome;** Definitely yes (low risk of bias); Outcomes were objectively assessed through pathology reports and medical record reviews; "Final pathology reports were used for disease conﬁrmation in patients who underwent appendectomy."

**Item 7; Was the follow up of cohorts adequate;** Probably yes; The study followed patients through definitive diagnoses, suggesting adequate follow-up; "In 13 patients, a normal appendix was diagnosed at MRI, none of whom had appendicitis."

**Item 8; Were co-interventions similar between groups;** Probably yes; The study suggests that imaging techniques were applied similarly across groups; "A total of 33 pregnant patients with suspected appendicitis underwent US and MRI."

ID: Israel, 2008; Cohort name: Pregnant patients with suspected appendicitis who underwent both US and MRI.

Item 1: Probably yes.

Item 2: Definitely yes (low risk of bias).

Item 3: Probably yes.

Item 4: Probably yes.

Item 5: Probably yes.

Item 6: Definitely yes (low risk of bias).

Item 7: Probably yes.

Item 8: Probably yes.

Title: Ultrafast 3-T MRI in the Evaluation of Children With Acute Lower Abdominal Pain for the Detection of Appendicitis

**First response:**

**Item 1; Was selection of exposed and non-exposed cohorts drawn from the same population;** Probably yes; The study included pediatric patients with acute abdominal pain suspicious for acute appendicitis recruited from the emergency department, suggesting a single population source; "42 patients 4–17 years old with acute abdominal pain suspicious for acute appendicitis were recruited in the emergency department setting to undergo unenhanced abdominal MRI."

**Item 2; Can we be confident in the assessment of exposure;** Definitely yes (low risk of bias); The use of MRI as a diagnostic tool was clearly defined and applied; "All scans were performed without sedation or oral or IV contrast agent."

**Item 3; Can we be confident that the outcome of interest was not present at start of study;** Definitely yes (low risk of bias); The study design implies that the outcomes were measured from the start of the clinical encounter; "Patients underwent CT or ultrasound or both as a comparison study to the MRI examination."

**Item 4; Did the study match exposed and unexposed for all variables that are associated with the outcome of interest or did the statistical analysis adjust for these prognostic variables;** Probably yes; The study used clinical follow-up and pathology results to confirm the diagnosis, suggesting an effort to control for confounding factors; "Clinical follow-up was obtained for all patients for at least 6 months."

**Item 5; Can we be confident in the assessment of the presence or absence of prognostic factors;** Probably yes; The study used a combination of imaging techniques and clinical follow-up for confirmation, suggesting reliable data; "Pathology results were obtained for all patients who underwent surgery."

**Item 6; Can we be confident in the assessment of outcome;** Definitely yes (low risk of bias); Outcomes were objectively assessed through pathology reports and clinical follow-up; "A diagnosis of acute appendicitis was made when the following features were present: markedly hyperintense thickened wall, markedly hyperintense periappendiceal tissue, and dilated appendix (6 mm)."

**Item 7; Was the follow up of cohorts adequate;** Definitely yes (low risk of bias); The study followed patients for at least 6 months, suggesting adequate follow-up; "Clinical follow-up was obtained for all patients for at least 6 months."

**Item 8; Were co-interventions similar between groups;** Probably yes; The study suggests that imaging techniques were applied similarly across groups; "Patients underwent CT or ultrasound or both as a comparison study to the MRI examination."

ID: Johnson, 2012; Cohort name: Pediatric patients with suspected appendicitis who underwent MRI.

Item 1: Probably yes.

Item 2: Definitely yes (low risk of bias).

Item 3: Definitely yes (low risk of bias).

Item 4: Probably yes.

Item 5: Probably yes.

Item 6: Definitely yes (low risk of bias).

Item 7: Definitely yes (low risk of bias).

Item 8: Probably yes.

**Second response:**

**Item 1; Was selection of exposed and non-exposed cohorts drawn from the same population;** Probably yes; The study included pediatric patients with acute abdominal pain suspicious for acute appendicitis recruited from the emergency department, suggesting a single population source; "42 patients 4–17 years old with acute abdominal pain suspicious for acute appendicitis were recruited in the emergency department setting to undergo unenhanced abdominal MRI."

**Item 2; Can we be confident in the assessment of exposure;** Definitely yes (low risk of bias); The use of MRI as a diagnostic tool was clearly defined and applied; "All scans were performed without sedation or oral or IV contrast agent."

**Item 3; Can we be confident that the outcome of interest was not present at start of study;** Probably yes; The study design implies that the outcomes were measured from the start of the clinical encounter; "Patients underwent CT or ultrasound or both as a comparison study to the MRI examination."

**Item 4; Did the study match exposed and unexposed for all variables that are associated with the outcome of interest or did the statistical analysis adjust for these prognostic variables;** Probably yes; The study used clinical follow-up and pathology results to confirm the absence or presence of appendicitis, suggesting an effort to control for confounding factors; "Clinical follow-up was obtained for all patients for at least 6 months."

**Item 5; Can we be confident in the assessment of the presence or absence of prognostic factors;** Probably yes; The study used a combination of imaging techniques and clinical follow-up for confirmation, suggesting reliable data; "The sensitivity, speciﬁcity, PPV, and NPV were calculated for US and MRI in the diagnosis of appendicitis."

**Item 6; Can we be confident in the assessment of outcome;** Definitely yes (low risk of bias); Outcomes were objectively assessed through pathology reports and clinical follow-up; "Final pathology reports were used for disease conﬁrmation in patients who underwent appendectomy."

**Item 7; Was the follow up of cohorts adequate;** Definitely yes (low risk of bias); The study followed patients for at least 6 months, suggesting adequate follow-up; "Clinical follow-up was obtained for all patients for at least 6 months."

**Item 8; Were co-interventions similar between groups;** Probably yes; The study suggests that imaging techniques were applied similarly across groups; "Patients underwent CT or ultrasound or both as a comparison study to the MRI examination."

ID: Johnson, 2012; Cohort name: Pediatric patients with suspected appendicitis who underwent MRI.

Item 1: Probably yes.

Item 2: Definitely yes (low risk of bias).

Item 3: Probably yes.

Item 4: Probably yes.

Item 5: Probably yes.

Item 6: Definitely yes (low risk of bias).

Item 7: Definitely yes (low risk of bias).

Item 8: Probably yes.

Title: Young and Middle-Aged Adults Hospitalized with COVID-19 Pneumonia (COVID-UNDER50)

**First response:**

**Item 1; Was selection of exposed and non-exposed cohorts drawn from the same population;** Probably no; The article does not provide information on whether the exposed and non-exposed cohorts were drawn from the same population. NR

**Item 2; Can we be confident in the assessment of exposure;** Probably yes; The exposure assessment seems to rely on confirmed or probable diagnosis of SARS-CoV2 infection, which suggests a reasonable method of exposure assessment. "Consecutive patients, aged 18–50 years, hospitalized for confirmed or probable diagnosis of SARS-CoV2 infection..."

**Item 3; Can we be confident that the outcome of interest was not present at start of study;** Definitely yes (low risk of bias); The study includes patients hospitalized for COVID-19, implying the outcome (hospitalization) was not present at the start. "Consecutive patients... hospitalized..."

**Item 4; Did the study match exposed and unexposed for all variables that are associated with the outcome of interest or did the statistical analysis adjust for these prognostic variables;** Probably no; While the study collected data on various factors, it is unclear if all potential confounders were adjusted for in the analysis. NR

**Item 5; Can we be confident in the assessment of the presence or absence of prognostic factors;** Probably no; The article does not provide enough detail on the methods used to assess prognostic factors such as hypertension, diabetes, etc. NR

**Item 6; Can we be confident in the assessment of outcome;** Probably yes; The study appears to have clear criteria for determining outcomes such as mechanical ventilation and death. "The follow-up period was the time from admission to the date of discharge or death."

**Item 7; Was the follow up of cohorts adequate;** Probably yes; The article does not mention any significant loss to follow-up; NR

**Item 8; Were co-interventions similar between groups;** Probably no; The article does not provide information on whether co-interventions were similar between groups, increasing the risk of bias. NR

ID: Bonifazi, 2021; Cohort name: Young and Middle-Aged Adults Hospitalized with COVID-19 Pneumonia (COVID-UNDER50)

Item 1: Probably no.

Item 2: Probably yes.

Item 3: Definitely yes (low risk of bias).

Item 4: Probably no.

Item 5: Probably no.

Item 6: Probably yes.

Item 7: Probably yes.

Item 8: Probably no.

**Second response:**

**Item 1; Was selection of exposed and non-exposed cohorts drawn from the same population;** Probably no; The article does not clearly describe whether the cohorts were drawn from the same population or not; NR

**Item 2; Can we be confident in the assessment of exposure;** Probably yes; The exposure assessment seems to rely on confirmed or probable diagnosis of SARS-CoV2 infection; "Consecutive patients, aged 18–50 years, hospitalized for confirmed or probable diagnosis of SARS-CoV2 infection between 28 February and 15 May, were included in the present study."

**Item 3; Can we be confident that the outcome of interest was not present at start of study;** Definitely yes (low risk of bias); The study included patients with confirmed or probable diagnosis of COVID-19, implying the outcome was not present at the start; "Consecutive patients, aged 18–50 years, hospitalized for confirmed or probable diagnosis of SARS-CoV2 infection between 28 February and 15 May, were included in the present study."

**Item 4; Did the study match exposed and unexposed for all variables that are associated with the outcome of interest or did the statistical analysis adjust for these prognostic variables;** Probably no; The article does not mention matching or adjustment for all prognostic variables; NR

**Item 5; Can we be confident in the assessment of the presence or absence of prognostic factors;** Probably no; The article does not provide information on the accuracy or repeatability of data quality for prognostic factors; NR

**Item 6; Can we be confident in the assessment of outcome;** Definitely yes (low risk of bias); The outcome measures were clearly defined and included mechanical ventilation and death; "Main clinical outcomes (death, discharge), were collected during the follow-up period."

**Item 7; Was the follow up of cohorts adequate;** Probably yes; The article does not mention any significant loss to follow-up; NR

**Item 8; Were co-interventions similar between groups;** Probably yes; The article does not mention any co-interventions that might affect the outcome of interest; NR

ID: Bonifazi, 2021; Cohort name: Young and Middle-Aged Adults with COVID-19 Pneumonia.

Item 1: Probably no.

Item 2: Probably yes.

Item 3: Definitely yes (low risk of bias).

Item 4: Probably no.

Item 5: Probably no.

Item 6: Definitely yes (low risk of bias).

Item 7: Probably yes.

Item 8: Probably yes.

Title: Overweight/obesity as the potentially most important lifestyle factor associated with signs of pneumonia in COVID-19

**First response:**

**Item 1; Was selection of exposed and non-exposed cohorts drawn from the same population;** Probably no; The study recruited participants through online means, which may not represent the entire population; "Participants were recruited through medical information web sites, social media postings and general news media."

**Item 2; Can we be confident in the assessment of exposure;** Probably yes; The exposure assessment was based on PCR tests or antibody testing, indicating a reliable method of determining exposure; "Infection with SARS-CoV-2 had to be diagnosed by PCR from a nasopharyngeal swab or, in retrospect, by antibody testing."

**Item 3; Can we be confident that the outcome of interest was not present at start of study;** Definitely yes (low risk of bias); The outcome measures were clearly defined and would not be present at the start of the study; "We defined the main outcome as the presence vs. the absence of signs of pneumonia..."

**Item 4; Did the study match exposed and unexposed for all variables that are associated with the outcome of interest or did the statistical analysis adjust for these prognostic variables;** Probably yes; “Overweight/obesity remained significantly associated with signs of pneumonia in a multivariate analysis adjusted for these two disease groups, age and sex”

**Item 5; Can we be confident in the assessment of the presence or absence of prognostic factors;** Probably no; The assessment of prognostic factors relied on self-report, which may be subject to bias; "The survey covered demographic information, eight lifestyle factors, comorbidities and medication use."

**Item 6; Can we be confident in the assessment of outcome;** Probably yes; The study had clear criteria for outcome assessment, reducing the risk of bias; "The first outcome variable was self-reported severity of COVID-19..."

**Item 7; Was the follow up of cohorts adequate;** Probably yes; The article does not mention any significant loss to follow-up; “no data were missing.”

**Item 8; Were co-interventions similar between groups;** Probably yes; The article does not mention any co-interventions that might affect the outcome of interest; NR

ID: Sacco, 2020; Cohort name: German Adults with COVID-19

Item 1: Probably no.

Item 2: Probably yes.

Item 3: Definitely yes (low risk of bias).

Item 4: Probably yes.

Item 5: Probably no.

Item 6: Probably yes.

Item 7: Definitely yes (low risk of bias).

Item 8: Probably yes.

**Second response:**

**Item 1; Was selection of exposed and non-exposed cohorts drawn from the same population;** Probably no; The study does not provide information on whether exposed and non-exposed cohorts were drawn from the same population; NR

**Item 2; Can we be confident in the assessment of exposure;** Probably yes; The exposure assessment seems to rely on PCR-based diagnosis and retrospective antibody testing; "Infection with SARS-CoV-2 had to be diagnosed by PCR from a nasopharyngeal swab or, in retrospect, by antibody testing."

**Item 3; Can we be confident that the outcome of interest was not present at start of study;** Definitely yes (low risk of bias); The study defined the outcome as the presence vs. the absence of signs of pneumonia; "We defined the main outcome as the presence vs. the absence of signs of pneumonia, represented by dyspnea, the requirement for oxygen therapy or intubation."

**Item 4; Did the study match exposed and unexposed for all variables that are associated with the outcome of interest or did the statistical analysis adjust for these prognostic variables;** Probably yes; “Overweight/obesity remained significantly associated with signs of pneumonia in a multivariate analysis adjusted for these two disease groups, age and sex”

**Item 5; Can we be confident in the assessment of the presence or absence of prognostic factors;** Probably no; The article does not provide information on the accuracy or repeatability of data quality for prognostic factors; NR

**Item 6; Can we be confident in the assessment of outcome;** Definitely yes (low risk of bias); The outcome measures were clearly defined; "The first outcome variable was self-reported severity of COVID-19, derived from a survey question with 5 possible answers."

**Item 7; Was the follow up of cohorts adequate;** Probably yes; The article does not mention any significant loss to follow-up; NR

**Item 8; Were co-interventions similar between groups;** Probably yes; The article does not mention any co-interventions that might affect the outcome of interest; NR

ID: Sacco, 2020; Cohort name: German Adults with COVID-19.

Item 1: Probably no.

Item 2: Probably yes.

Item 3: Definitely yes (low risk of bias).

Item 4: Probably yes.

Item 5: Probably no.

Item 6: Definitely yes (low risk of bias).

Item 7: Probably yes.

Item 8: Probably yes.

Title: Oncoplastic Breast-Conserving Surgery Reduces Mastectomy and Postoperative Re-excision Rates

**First response:**

**Item 1; Was selection of exposed and non-exposed cohorts drawn from the same population;** Definitely yes (low risk of bias); "This was a single-institution, retrospective study"

**Item 2; Can we be confident in the assessment of exposure;**Definitely yes (low risk of bias)

**Item 3; Can we be confident that the outcome of interest was not present at start of study;** Definitely yes (low risk of bias)

**Item 4; Did the study match exposed and unexposed for all variables that are associated with the outcome of interest or did the statistical analysis adjust for these prognostic variables;** Probably no; NR

**Item 5; Can we be confident in the assessment of the presence or absence of prognostic factors;** Probably no; NR

**Item 6; Can we be confident in the assessment of outcome;** Definitely yes (low risk of bias); "There were no differences in patient age or tumor characteristics between the two groups"

**Item 7; Was the follow up of cohorts adequate;** Probably yes; NR

**Item 8; Were co-interventions similar between groups;** Probably no; NR

ID: Crown, 2015; Cohort name: Standard Surgery group (SS) and Oncoplastic Surgery group (OS)

Item 1: Definitely yes (low risk of bias).

Item 2: Definitely yes (low risk of bias).

Item 3: Definitely yes (low risk of bias).

Item 4: Probably no

Item 5: Probably no

Item 6: Definitely yes (low risk of bias).

Item 7: Probably yes

Item 8: Probably no

**Second response:**

**Item 1; Was selection of exposed and non-exposed cohorts drawn from the same population;** Definitely yes (low risk of bias); "This was a single-institution, retrospective study"

**Item 2; Can we be confident in the assessment of exposure;**Definitely yes (low risk of bias)

**Item 3; Can we be confident that the outcome of interest was not present at start of study;** Definitely yes (low risk of bias); "Patients diagnosed with invasive or noninvasive breast carcinoma who underwent operative intervention were included in the study."

**Item 4; Did the study match exposed and unexposed for all variables that are associated with the outcome of interest or did the statistical analysis adjust for these prognostic variables;** Probably no; "The two groups were similar with respect to age and tumor characteristics."

**Item 5; Can we be confident in the assessment of the presence or absence of prognostic factors;** Probably no; "Specimen margins were inked by the operating surgeon at the time of resection..."

**Item 6; Can we be confident in the assessment of outcome;** Definitely yes (low risk of bias); "The threshold for returning to the operating room for postoperative reexcision for both groups was a margin <2 mm on final pathology."

**Item 7; Was the follow up of cohorts adequate;** Probably yes; "A total of 812 patients were evaluated; 425 patients were treated in the SS group compared with 387 patients in the OS group."

**Item 8; Were co-interventions similar between groups;** Probably no; "Oncoplastic BCS combines the traditional oncologic approach of wide local resection with plastic surgery incisions and tissue transfer techniques..."

ID: Crown, 2015; Cohort name: Standard Surgery group (SS) and Oncoplastic Surgery group (OS)

Item 1: Definitely yes (low risk of bias)

Item 2: Definitely yes (low risk of bias)

Item 3: Definitely yes (low risk of bias)

Item 4: Probably no

Item 5: Probably no

Item 6: Definitely yes (low risk of bias)

Item 7: Probably yes

Item 8: Probably no

Title: Comparison of Outcomes of Standard and Oncoplastic Breast-Conserving Surgery

**First response:**

**Item 1; Was selection of exposed and non-exposed cohorts drawn from the same population;** Definitely yes (low risk of bias); "One hundred and sixty-two patients who received a quadrantectomy because of breast cancer (group 1) and 106 breast cancer patients with macromastia who underwent breast-conserving surgery via bilateral reduction mammoplasty (group 2) between 2003 and 2010 were enrolled in this study."

**Item 2; Can we be confident in the assessment of exposure;** Probably no; NR

**Item 3; Can we be confident that the outcome of interest was not present at start of study;** Definitely yes (low risk of bias); "The study was approved by the local ethics committee. All the patients were informed with written explanations on both techniques."

**Item 4; Did the study match exposed and unexposed for all variables that are associated with the outcome of interest or did the statistical analysis adjust for these prognostic variables;** Probably no; "The groups were homogenous when age and tumor size were considered."

**Item 5; Can we be confident in the assessment of the presence or absence of prognostic factors;** Probably no; "Mammography and breast ultrasonography were routinely applied on the patients in order to display the tumor."

**Item 6; Can we be confident in the assessment of outcome;** Definitely yes (low risk of bias); "Three patients (1.8%) in group 1 and one patient (0.9%) in group 2 had local recurrences of the disease and received a mastectomy."

**Item 7; Was the follow up of cohorts adequate;** Probably yes; "The mean follow-up time was 37 months for group 1 and 33 months for group 2."

**Item 8; Were co-interventions similar between groups;** Probably no; "In group 1, 82.7% of the patients had a surgical margin wider than 2 mm, and 76.5% of the patients in the same group had a surgical margin wider than 10 mm, where 11% patients had positive surgical margin. When compared to group 2, the rates were 89%, 84%, and 8.4%, respectively."

ID: Gulcelik, 2013; Cohort name: Group 1 (Quadrantectomy) and Group 2 (Bilateral Reduction Mammoplasty - BRM)

Item 1: Definitely yes (low risk of bias)

Item 2: Probably no

Item 3: Definitely yes (low risk of bias)

Item 4: Probably no

Item 5: Probably no

Item 6: Definitely yes (low risk of bias)

Item 7: Probably yes

Item 8: Probably no

**Second response:**

**Item 1; Was selection of exposed and non-exposed cohorts drawn from the same population;** Definitely yes (low risk of bias); "One hundred and sixty-two patients who received a quadrantectomy because of breast cancer (group 1) and 106 breast cancer patients with macromastia who underwent breast-conserving surgery via bilateral reduction mammoplasty (group 2) between 2003 and 2010 were enrolled in this study."

**Item 2; Can we be confident in the assessment of exposure;** Probably no; The article does not provide information on how exposure was determined; NR

**Item 3; Can we be confident that the outcome of interest was not present at start of study;** Definitely yes (low risk of bias); The study excluded patients with in situ carcinoma and multicentric or multifocal diseases, indicating that the outcome was not present at the start; “The patients who have had invasive breast cancer were enrolled in the study, where the patients who had in situ carcinoma and multicentric or multifocal diseases were excluded.”

**Item 4; Did the study match exposed and unexposed for all variables that are associated with the outcome of interest or did the statistical analysis adjust for these prognostic variables;** Probably no; The article does not mention matching or adjustment for prognostic variables; NR

**Item 5; Can we be confident in the assessment of the presence or absence of prognostic factors;** Probably no; The article does not provide information on the assessment of prognostic factors; NR

**Item 6; Can we be confident in the assessment of outcome;** Probably yes; The study followed patients postoperatively with regular controls and used imaging and tumor markers for surveillance, suggesting a reliable assessment of outcomes; “In the first 2 years of the postoperative period, the patients came in every 3 months for controls...Mammography and breast ultrasonography were applied once a year.”

**Item 7; Was the follow up of cohorts adequate;** Probably yes; The median follow-up time was 37 months for group 1 and 33 months for group 2, suggesting adequate follow-up; “The median follow-up time was 37 months (range, 20-124 months) for group 1 and 33 months (range, 16-88 months) for group 2.”

**Item 8; Were co-interventions similar between groups;** Probably no; The article does not provide information on whether co-interventions were similar between the two groups; NR

ID: Gulcelik, 2013; Cohort name: Group 1 (Quadrantectomy patients) and Group 2 (Bilateral Reduction Mammoplasty patients).

Item 1: Definitely yes (low risk of bias)

Item 2: Probably no

Item 3: Definitely yes (low risk of bias)

Item 4: Probably no

Item 5: Probably no

Item 6: Probably yes

Item 7: Probably yes

Item 8: Probably no

Title: Oncoplastic breast conservation does not lead to a delay in the commencement of adjuvant chemotherapy in breast cancer patients

**First response:**

**Item 1; Was selection of exposed and non-exposed cohorts drawn from the same population;** Probably no; It is unclear whether cohorts were drawn from the same population as the study did not provide this information; NR

**Item 2; Can we be confident in the assessment of exposure;** Probably no; The article does not provide information on how exposure was determined; NR

**Item 3; Can we be confident that the outcome of interest was not present at start of study;** Definitely yes (low risk of bias); The study excluded patients who received neoadjuvant chemotherapy, indicating that the outcome was not present at the start; “Patients treated with neoadjuvant chemotherapy were excluded from the analysis.”

**Item 4; Did the study match exposed and unexposed for all variables that are associated with the outcome of interest or did the statistical analysis adjust for these prognostic variables;** Probably no; The article does not mention matching or adjustment for prognostic variables; NR

**Item 5; Can we be confident in the assessment of the presence or absence of prognostic factors;** Probably no; The article does not provide information on the assessment of prognostic factors; NR

**Item 6; Can we be confident in the assessment of outcome;** Definitely yes (low risk of bias); The study used multidisciplinary team decisions and pharmacy records to measure the time to chemotherapy, indicating a reliable assessment of outcomes; “Time between multidisciplinary team decision to offer chemotherapy and delivery of first cycle of chemotherapy was measured and compared among the four groups of patients.”

**Item 7; Was the follow up of cohorts adequate;** Probably yes; The study measured the time from decision to chemotherapy which serves as a proxy for follow-up; “Time to chemotherapy of breast cancer patients (n = 169) treated with OBCS (n = 31) were 29 [16e58] days, while it was 29.5 [15e105] days after WLE (n = 66), 29 [15e57] days after Ms (n = 56) and 31 [15e58] days after MsIR (n = 16).”

**Item 8; Were co-interventions similar between groups;** Probably no; The article does not provide information on whether co-interventions were similar between the two groups; NR

ID: Kahn, 2013; Cohort name: OBCS patients and control groups (WLE, Ms, MsIR patients).

Item 1: Probably no

Item 2: Probably no

Item 3: Definitely yes (low risk of bias)

Item 4: Probably no

Item 5: Probably no

Item 6: Definitely yes (low risk of bias)

Item 7: Probably yes

Item 8: Probably no

**Second response:**

**Item 1; Was selection of exposed and non-exposed cohorts drawn from the same population;** Probably no; Does not clearly describe whether it is the same or different population; “Breast cancer patients who required adjuvant chemotherapy after OBCS, WLE, Ms and MsIR were identified from prospectively maintained institutional databases.”

**Item 2; Can we be confident in the assessment of exposure;** Probably no; If not described how exposure information was obtained; NR.

**Item 3; Can we be confident that the outcome of interest was not present at start of study;** Definitely yes (low risk of bias); If study participants did not have the outcome of interest at baseline; “Patients treated with neoadjuvant chemotherapy were excluded from the analysis.”

**Item 4; Did the study match exposed and unexposed for all variables that are associated with the outcome of interest or did the statistical analysis adjust for these prognostic variables;** Probably no; If the article only matched or adjusted for a few or no reasonable prognostic variables; “A combined analysis involving all four groups demonstrated no statistically significant difference (p = 0.524).”

**Item 5; Can we be confident in the assessment of the presence or absence of prognostic factors;** Probably no; If the article states that prognostic factors were obtained through retrospective charts but repeatability could not be demonstrated or from a database where prognostic information quality is uncertain; NR.

**Item 6; Can we be confident in the assessment of outcome;** Probably yes; If the article states that the outcome indicator is non-blind independent evaluation, self-report; “Time between multidisciplinary team decision to offer chemotherapy and delivery of first cycle of chemotherapy was measured and compared among the four groups of patients.”

**Item 7; Was the follow up of cohorts adequate;** Probably no; If follow-up rate during the study is 80-89%, losses to follow-up may be related to outcomes; “169 breast cancer patients were identified from the databases altogether, who received adjuvant chemotherapy after surgical treatment.”

**Item 8; Were co-interventions similar between groups;** Probably no; If few or none of the co-interventions that may affect the outcome of interest are similar in the exposed and unexposed cohorts of the queue; “Chemotherapy was administered according to evidence-based guidelines of the Beatson West of Scotland Cancer Centre of the given time period.”

ID: Kahn, 2013; Cohort name: OBCS patients, WLE patients, Ms patients, MsIR patients.

Item 1: Probably no.

Item 2: Probably no.

Item 3: Definitely yes (low risk of bias).

Item 4: Probably no.

Item 5: Probably no.

Item 6: Probably yes.

Item 7: Probably no.

Item 8: Probably no.

Title: Obese Women Experience Fewer Complications after Oncoplastic Breast Repair following Partial Mastectomy Than after Immediate Total Breast Reconstruction

**First response:**

**Item 1; Was selection of exposed and non-exposed cohorts drawn from the same population;** Definitely yes (low risk of bias); The study compared two different groups of patients undergoing different types of breast reconstruction, it is not clear if both cohorts were drawn from the same population; "We retrospectively searched a prospectively maintained departmental database for consecutive obese patients (body mass index ≥30 kg/m2) who had undergone oncoplastic breast reconstruction or implant-based or abdominally based free flap immediate breast reconstruction at a single center between January of 2005 and April of 2013."

**Item 2; Can we be confident in the assessment of exposure;** Probably no; Study does not describe how exposure information was obtained; NR

**Item 3; Can we be confident that the outcome of interest was not present at start of study;** Definitely yes (low risk of bias); Participants did not have the outcome of interest at baseline; “Patients were excluded if they were not obese”

**Item 4; Did the study match exposed and unexposed for all variables that are associated with the outcome of interest or did the statistical analysis adjust for these prognostic variables;** Definitely yes (low risk of bias); “Logistic regression was used to analyze the associations between patient and surgical characteristics and postoperative outcomes.”

**Item 5; Can we be confident in the assessment of the presence or absence of prognostic factors;** Probably no; Not obtained through secure methods; NR

**Item 6; Can we be confident in the assessment of outcome;** Probably yes; Non-blind independent evaluation; “The study included 408 patients: 131 oncoplastic breast reconstruction and 277 immediate breast reconstruction patients.”

**Item 7; Was the follow up of cohorts adequate;** Probably yes; Follow-up rate during the study is 80-89%

**Item 8; Were co-interventions similar between groups;** Probably no; Few of the co-interventions that may affect the outcome of interest are similar; “Nevertheless, the oncoplastic breast reconstruction group experienced fewer major complications requiring operative management (3.8 percent versus 28.5 percent; p < 0.001),”

ID: Tong, 2016; Cohort name: Obese patients undergoing oncoplastic breast reconstruction and immediate breast reconstruction.

Item 1: Definitely yes (low risk of bias).

Item 2: Probably no.

Item 3: Definitely yes (low risk of bias).

Item 4: Definitely yes (low risk of bias).

Item 5: Probably no.

Item 6: Probably yes.

Item 7: Probably yes.

Item 8: Probably no.

**Second response:**

**Item 1; Was selection of exposed and non-exposed cohorts drawn from the same population;** Definitely yes (low risk of bias); The study compared two different groups of patients undergoing different types of breast reconstruction, it is not clear if both cohorts were drawn from the same population; "We retrospectively searched a prospectively maintained departmental database for consecutive obese patients (body mass index ≥30 kg/m2) who had undergone oncoplastic breast reconstruction or implant-based or abdominally based free flap immediate breast reconstruction at a single center between January of 2005 and April of 2013."

**Item 2; Can we be confident in the assessment of exposure;** Probably no; The article does not provide information on how the exposure was determined; NR

**Item 3; Can we be confident that the outcome of interest was not present at start of study;** Definitely yes (low risk of bias); The study included patients who underwent breast reconstruction, implying that the outcome of interest, complications, were not present at the start; "Complication rates were compared for oncoplastic breast reconstruction versus immediate breast reconstruction (with either implants or autologous tissue) in consecutive obese patients (body mass index ≥30 kg/m2) treated at a single center between January of 2005 and April of 2013."

**Item 4; Did the study match exposed and unexposed for all variables that are associated with the outcome of interest or did the statistical analysis adjust for these prognostic variables;** Definitely yes (low risk of bias); “Logistic regression was used to analyze the associations between patient and surgical characteristics and postoperative outcomes.”

**Item 5; Can we be confident in the assessment of the presence or absence of prognostic factors;** Probably yes.

**Item 6; Can we be confident in the assessment of outcome;** Probably no; The article does not provide detail on how outcomes were assessed; "The overall complication rate was defined as the incidence of patients who experienced at least one postoperative surgical complication, irrespective of the relative severity of that complication."

**Item 7; Was the follow up of cohorts adequate;** **Item 7; Was the follow up of cohorts adequate;** Probably yes; Follow-up rate during the study is 80-89%

**Item 8; Were co-interventions similar between groups;** Probably no; The article does not provide information on whether co-interventions were similar between groups; "Oncoplastic breast reconstruction was offered by our surgeons, even for patients with T1 tumors, based on several factors, including the following: (1) facilitating optimal treatment fields for radiation therapy, (2) optimizing the aesthetic outcome, and (3) patient preference in women with symptoms of macromastia such as back discomfort."

ID: Tong, 2016; Cohort name: Obese breast cancer patients undergoing oncoplastic breast reconstruction

Item 1: Definitely yes (low risk of bias).

Item 2: Probably no.

Item 3: Definitely yes (low risk of bias).

Item 4: Definitely yes (low risk of bias).

Item 5: Probably yes.

Item 6: Probably no.

Item 7: Probably yes.

Item 8: Probably no.

Title: Interest level in 2-year-olds with autism spectrum disorder predicts rate of verbal, nonverbal, and adaptive skill acquisition

**First response:**

**Item 1; Was selection of exposed and non-exposed cohorts drawn from the same population;** Probably no; NR

**Item 2; Can we be confident in the assessment of exposure;** Probably no; NR

**Item 3; Can we be confident that the outcome of interest was not present at start of study;** "Definitely yes (low risk of bias)."; The study assessed skills at Time 1 (around 22 months of age) and again at Time 2 (around 38 months of age), indicating the outcomes were not present at the start.

**Item 4; Did the study match exposed and unexposed for all variables that are associated with the outcome of interest or did the statistical analysis adjust for these prognostic variables;** "Probably yes."; The study entered multiple variables into regression models, suggesting an attempt to control for confounding factors.

**Item 5; Can we be confident in the assessment of the presence or absence of prognostic factors;** "Probably yes."; The study used standardized assessments such as the ADOS and MSEL, which are reliable and valid measures.

**Item 6; Can we be confident in the assessment of outcome;** "Definitely yes (low risk of bias)."; The study used validated instruments such as the VABS and MSEL to assess outcomes.

**Item 7; Was the follow up of cohorts adequate;** "Probably yes."; The study followed up with the children after a mean of 16.3 months, suggesting adequate follow-up.

**Item 8; Were co-interventions similar between groups;** Probably no; "A total of 94% of children received speech-therapy (average 2 h/week), 77% received ABA (e.g. EIBI; average 10 h/week), and 36% received developmental interventions (e.g. ESDM; average 7 h/week). A total of 84% of children received more than one type of intervention"

ID: Klintwall, 2015; Cohort name: 2-year-olds with autism spectrum disorder.

Item 1: Probably no.

Item 2: Probably no.

Item 3: Definitely yes (low risk of bias).

Item 4: Probably yes.

Item 5: Probably yes.

Item 6: Definitely yes (low risk of bias).

Item 7: Probably yes.

Item 8: Probably no.

**Second response:**

**Item 1; Was selection of exposed and non-exposed cohorts drawn from the same population;** Probably no; Unclear if exposed and non-exposed cohorts were drawn from the same population; NR

**Item 2; Can we be confident in the assessment of exposure;** Probably no; Unclear how exposure was determined; NR

**Item 3; Can we be confident that the outcome of interest was not present at start of study;** Definitely yes (low risk of bias); Participants did not have the outcome of interest at baseline; "A total of 70 toddlers with autism spectrum disorder, mean age of 21.9 months, were scored using Interest Level Scoring for Autism, quantifying toddlers’ interest in toys, social routines, and activities that could serve as reinforcers in an intervention."

**Item 4; Did the study match exposed and unexposed for all variables that are associated with the outcome of interest or did the statistical analysis adjust for these prognostic variables;** "Probably yes."; The study used regression models that included several predictors, suggesting an attempt to control for confounding variables.

**Item 5; Can we be confident in the assessment of the presence or absence of prognostic factors;** "Probably yes."; Prognostic factors were assessed using standardized instruments like ADOS and MSEL.

**Item 6; Can we be confident in the assessment of outcome;** "Definitely yes (low risk of bias)."; Outcomes were measured using validated tools such as VABS and MSEL.

**Item 7; Was the follow up of cohorts adequate;** "Probably yes."; The study followed up with the children after a mean of 16.3 months, suggesting adequate follow-up.

**Item 8; Were co-interventions similar between groups;** Probably no; Unclear if co-interventions were similar; NR

ID: Klintwall, 2015; Cohort name: 2-year-olds with autism spectrum disorder.

Item 1: Probably no.

Item 2: Probably no.

Item 3: Definitely yes (low risk of bias).

Item 4: Probably yes.

Item 5: Probably yes.

Item 6: Definitely yes (low risk of bias).

Item 7: Probably yes.

Item 8: Probably no.

Title: Detecting interictal discharges in first seizure patients Ambulatory EEG or EEG after sleep deprivation?

**First response:**

**Item 1; Was selection of exposed and non-exposed cohorts drawn from the same population;** Probably no; The study included patients with an unprovoked first seizure and a normal routine EEG who subsequently underwent an sdEEG or aEEG, it is unclear if they were drawn from the same population; NR.

**Item 2; Can we be confident in the assessment of exposure;** Probably no; The method of exposure assessment is not detailed in the text; NR.

**Item 3; Can we be confident that the outcome of interest was not present at start of study;** Definitely yes (low risk of bias); "We included 104 patients. The majority of patients presented with either a primary or secondary generalized seizure, 46% in the aEEG group, 62% in the sdEEG group" indicating that seizures were not present at the start; "We included 104 patients. The majority of patients presented with either a primary or secondary generalized seizure, 46% in the aEEG group, 62% in the sdEEG group".

**Item 4; Did the study match exposed and unexposed for all variables that are associated with the outcome of interest or did the statistical analysis adjust for these prognostic variables;** Probably no; The text does not provide information on matching or adjustment for prognostic variables; NR.

**Item 5; Can we be confident in the assessment of the presence or absence of prognostic factors;** Probably no; The presence or absence of prognostic factors is not clearly assessed in the text; NR.

**Item 6; Can we be confident in the assessment of outcome;** Probably yes; "All patients had a follow-up of one year" suggests a clear assessment of outcome; "All patients had a follow-up of one year".

**Item 7; Was the follow up of cohorts adequate;** Probably yes; "All patients had a follow-up of one year" suggests adequate follow-up; "All patients had a follow-up of one year".

**Item 8; Were co-interventions similar between groups;** Probably no; The text does not provide information on whether co-interventions were similar between groups; NR.

ID: Geut, 2017; Cohort name: First seizure patients with normal routine EEG.

Item 1: Probably no.

Item 2: Probably no.

Item 3: Definitely yes (low risk of bias).

Item 4: Probably no.

Item 5: Probably no.

Item 6: Probably yes.

Item 7: Probably yes.

Item 8: Probably no.

**Second response:**

**Item 1; Was selection of exposed and non-exposed cohorts drawn from the same population;** Probably no; Unclear if drawn from same population; NR

**Item 2; Can we be confident in the assessment of exposure;** Probably no; Unclear how exposure information was obtained; NR

**Item 3; Can we be confident that the outcome of interest was not present at start of study;** Definitely yes (low risk of bias); Patients had a normal routine EEG at baseline; "We included patients with an unprovoked first seizure and a normal routine EEG who subsequently underwent an sdEEG or aEEG."

**Item 4; Did the study match exposed and unexposed for all variables that are associated with the outcome of interest or did the statistical analysis adjust for these prognostic variables;** Probably no; Unclear if all prognostic variables were matched or adjusted; NR

**Item 5; Can we be confident in the assessment of the presence or absence of prognostic factors;** Probably no; Unclear how prognostic factors were assessed; NR

**Item 6; Can we be confident in the assessment of outcome;** Probably yes; Outcome assessment mentioned but not detailed; "We calculated specificity and sensitivity of sdEEG and aEEG, using the clinical diagnosis of epilepsy as golden standard."

**Item 7; Was the follow up of cohorts adequate;** Probably yes; One year follow-up mentioned; "All patients had a follow-up of one year."

**Item 8; Were co-interventions similar between groups;** Probably no; Unclear if co-interventions were similar; NR

ID: Geut, 2017; Cohort name: First seizure patients with normal routine EEG.

Item 1: Probably no.

Item 2: Probably no.

Item 3: Definitely yes (low risk of bias).

Item 4: Probably no.

Item 5: Probably no.

Item 6: Probably yes.

Item 7: Probably yes.

Item 8: Probably no.

Title: Is the first seizure epilepsy—and when?

**First response:**

**Item 1; Was selection of exposed and non-exposed cohorts drawn from the same population;** Probably no; Unclear if exposed and non-exposed drawn from same population; NR

**Item 2; Can we be confident in the assessment of exposure;** Definitely yes (low risk of bias); Exposure determined by EEG and neuroimaging; "EEG was performed in 98% of patients, and the findings were classified as normal or abnormal (epileptiform or nonspecific). Neuroimaging (computed tomography [CT], magnetic resonance imaging [MRI], or both, undertaken in 98% of patients) abnormalities identified were defined as epileptogenic if a lesion likely relevant to the cause of the seizure was found."

**Item 3; Can we be confident that the outcome of interest was not present at start of study;** Definitely yes (low risk of bias); Patients did not have outcome at baseline; "The likelihood of seizure recurrence at 10 years was ≥60% in patients with epileptiform abnormalities on EEG or neuroimaging abnormalities, therefore, meeting the new definition of epilepsy."

**Item 4; Did the study match exposed and unexposed for all variables that are associated with the outcome of interest or did the statistical analysis adjust for these prognostic variables;** Probably yes; Adjusted for multiple variables; "The risk factors for seizure recurrence consistently identified in prior studies (remote symptomatic etiology or the presence of an epileptogenic abnormality on neuroimaging and epileptiform abnormalities) were examined using Cox proportional hazards models."

**Item 5; Can we be confident in the assessment of the presence or absence of prognostic factors;** Definitely yes (low risk of bias); Prognostic factors obtained by measurements; "Seizure type and electroclinical syndrome (focal, generalized, or unclassified) were categorized for the presenting seizure according to published guidelines."

**Item 6; Can we be confident in the assessment of outcome;** Probably yes; Outcome indicator is non-blind independent evaluation; "A seizure occurring during follow-up at least 24 h after the first was considered a recurrence."

**Item 7; Was the follow up of cohorts adequate;** Probably no; Follow-up rate during the study is 80-89%; "Patients were reviewed in clinic 3–9 months after the index seizure, and if there had been no further seizures they were contacted thereafter by phone every 1–2 years until seizure recurrence occurred or death."

**Item 8; Were co-interventions similar between groups;** Probably no; Few relevant co-interventions documented to be similar; NR

ID: Lawn, 2015; Cohort name: 798 adults with a first-ever unprovoked seizure.

Item 1: Probably no.

Item 2: Definitely yes (low risk of bias).

Item 3: Definitely yes (low risk of bias).

Item 4: Probably yes.

Item 5: Definitely yes (low risk of bias).

Item 6: Probably yes.

Item 7: Probably no.

Item 8: Probably no.

**Second response:**

**Item 1; Was selection of exposed and non-exposed cohorts drawn from the same population;** Definitely yes (low risk of bias); From the same hospital-based first seizure clinic; "Prospective analysis of 798 adults with a first-ever unprovoked seizure seen at a hospital-based first seizure clinic between 2000 and 2011."

**Item 2; Can we be confident in the assessment of exposure;** Definitely yes (low risk of bias); Exposure determined by EEG and neuroimaging; "EEG was performed in 98% of patients, and the findings were classified as normal or abnormal (epileptiform or nonspecific). Neuroimaging (computed tomography [CT], magnetic resonance imaging [MRI], or both, undertaken in 98% of patients) abnormalities identified were defined as epileptogenic if a lesion likely relevant to the cause of the seizure was found."

**Item 3; Can we be confident that the outcome of interest was not present at start of study;** Definitely yes (low risk of bias); Outcome of interest not present at baseline; "Exclusion of patients with an early seizure recurrence while awaiting assessment may underestimate seizure recurrence, whereas their inclusion may overestimate seizure recurrence."

**Item 4; Did the study match exposed and unexposed for all variables that are associated with the outcome of interest or did the statistical analysis adjust for these prognostic variables;** Probably yes; Adjusted for multiple variables; "The risk factors for seizure recurrence consistently identified in prior studies (remote symptomatic etiology or the presence of an epileptogenic abnormality on neuroimaging and epileptiform abnormalities) were examined using Cox proportional hazards models."

**Item 5; Can we be confident in the assessment of the presence or absence of prognostic factors;** Definitely yes (low risk of bias); Prognostic factors obtained by measurements; "Seizure type and electroclinical syndrome (focal, generalized, or unclassified) were categorized for the presenting seizure according to published guidelines."

**Item 6; Can we be confident in the assessment of outcome;** Definitely yes (low risk of bias); Outcome assessment by clinical follow-up; "Patients were reviewed in clinic 3–9 months after the index seizure, and if there had been no further seizures they were contacted thereafter by phone every 1–2 years until seizure recurrence occurred or death."

**Item 7; Was the follow up of cohorts adequate;** Probably yes; Follow-up was regular and losses were censored; "Patients were reviewed in clinic 3–9 months after the index seizure, and if there had been no further seizures they were contacted thereafter by phone every 1–2 years until seizure recurrence occurred or death."

**Item 8; Were co-interventions similar between groups;** Probably no; Unclear if co-interventions were similar; NR

ID: Lawn, 2015; Cohort name: 798 adults with a first-ever unprovoked seizure.

Item 1: Definitely yes (low risk of bias).

Item 2: Definitely yes (low risk of bias).

Item 3: Definitely yes (low risk of bias).

Item 4: Probably yes.

Item 5: Definitely yes (low risk of bias).

Item 6: Definitely yes (low risk of bias).

Item 7: Probably yes.

Item 8: Probably no.

Title: Association of Abnormal Renal Profiles and Proliferative Diabetic Retinopathy and Diabetic Macular Edema in an Asian Population With Type 2 Diabetes

**First response:**

**Item 1; Was selection of exposed and non-exposed cohorts drawn from the same population;** Probably no; Unclear if exposed and non-exposed drawn from same population; NR

**Item 2; Can we be confident in the assessment of exposure;** Probably yes; Exposure determined by measurements; "The baseline and mean follow-up renal profiles including serum creatinine level, estimated glomerular filtration rate (eGFR), and urinary albumin/creatinine ratio (ACR)."

**Item 3; Can we be confident that the outcome of interest was not present at start of study;** Definitely yes (low risk of bias); Outcome not present at baseline; "Of the 1345 patients without DR at baseline, 685 patients (50.9%) developed DR during the follow-up period."

**Item 4; Did the study match exposed and unexposed for all variables that are associated with the outcome of interest or did the statistical analysis adjust for these prognostic variables;** Probably yes; Adjusted for multiple variables; "While analyzing the baseline renal profile, the following covariates were adjusted in the regression models using forward selection: age, sex, duration of diabetes, baseline body mass index, SBP, fasting glucose levels, HbA1c, total cholesterol levels, high-density lipoprotein cholesterol levels, low-density lipoprotein cholesterol levels, and triglyceride levels."

**Item 5; Can we be confident in the assessment of the presence or absence of prognostic factors;** Definitely yes (low risk of bias); Prognostic factors obtained by measurements; "All patients had their blood samples obtained at 8 AM after an overnight fast and before taking their morning medication; overnight first-void urine samples were also obtained."

**Item 6; Can we be confident in the assessment of outcome;** Definitely yes (low risk of bias); Outcome assessment by trained graders; "The images were then graded by well-trained graders according to the international clinical diabetic retinopathy and diabetic macular edema disease severity scales."

**Item 7; Was the follow up of cohorts adequate;** Probably yes; Follow-up was regular and losses were censored; "All patients obtained regular follow-up for systemic checkups every 2 to 6 months during the follow-up period."

**Item 8; Were co-interventions similar between groups;** Probably no; Unclear if co-interventions were similar; NR

ID: Hsieh, 2017; Cohort name: 2135 patients with type 2 diabetes.

Item 1: Probably no.

Item 2: Probably yes.

Item 3: Definitely yes (low risk of bias).

Item 4: Probably yes.

Item 5: Definitely yes (low risk of bias).

Item 6: Definitely yes (low risk of bias).

Item 7: Probably yes.

Item 8: Probably no.

**Second response:**

**Item 1; Was selection of exposed and non-exposed cohorts drawn from the same population;** Probably no; The article does not describe the source of the population clearly; NR

**Item 2; Can we be confident in the assessment of exposure;** Probably yes; The article mentions that renal profiles including serum creatinine level, eGFR, and urinary ACR were measured at baseline; "This 8-year prospective cohort study...included 2135 patients with type 2 diabetes."

**Item 3; Can we be confident that the outcome of interest was not present at start of study;** Definitely yes (low risk of bias); The study excluded patients who had DR/PDR/DME at baseline; "Those who were lost to follow-up within 6 months were excluded from the study."

**Item 4; Did the study match exposed and unexposed for all variables that are associated with the outcome of interest or did the statistical analysis adjust for these prognostic variables;** Probably yes; The article states that Cox regression analyses were used to evaluate the hazard ratios (HRs) and adjusted for several covariates; "Cox regression models were used to calculate the hazard ratios (HRs) for the baseline and mean follow-up renal profiles for any new-onset DR/PDR/DME."

**Item 5; Can we be confident in the assessment of the presence or absence of prognostic factors;** Probably yes; The article states that prognostic factors were obtained by measuring blood samples and urinary samples; "All patients had their blood samples obtained at 8 AM after an overnight fast and before taking their morning medication; overnight first-void urine samples were also obtained."

**Item 6; Can we be confident in the assessment of outcome;** Definitely yes (low risk of bias); The article states that DR and DME were detected with nonmydriatic fundus photography and graded by well-trained graders; "Allparticipantsunderwentfundusphotographywitha45°nonmydriatic fundus camera...The images were then graded by well-trained graders."

**Item 7; Was the follow up of cohorts adequate;** Probably yes; The article states that patients obtained regular follow-up every 2 to 6 months and those who were lost to follow-up within 6 months were excluded; "All patients obtained regular follow-up for systemic checkups every 2 to 6 months during the follow-up period."

**Item 8; Were co-interventions similar between groups;** Probably no; The article does not provide enough information to determine whether co-interventions between groups were similar; NR

ID: Hsieh, 2017; Cohort name: Type 2 diabetes patients in Taiwan.

Item 1: Probably no.

Item 2: Probably yes.

Item 3: Definitely yes (low risk of bias).

Item 4: Probably yes.

Item 5: Probably yes.

Item 6: Definitely yes (low risk of bias).

Item 7: Probably yes.

Item 8: Probably no.

Title: Five-year incidence of diabetic retinopathy in the Melbourne Visual Impairment Project

**First response:**

**Item 1; Was selection of exposed and non-exposed cohorts drawn from the same population;** Probably no; The article does not provide enough information to determine whether the cohorts were drawn from the same population; "Eligible residents were then invited to attend locally established test sites for the complete eye examination and interview."

**Item 2; Can we be confident in the assessment of exposure;** Probably no; If the study is uncertain how exposure information was obtained; NR

**Item 3; Can we be confident that the outcome of interest was not present at start of study;** Definitely yes (low risk of bias); The study defined incidence as newly detected retinopathy at the follow-up examination for eyes with no retinopathy detected at the baseline examination; "Diabetic retinopathy incidence was defined as newly detected retinopathy at the follow-up examination for eyes with no retinopathy detected at the baseline examination."

**Item 4; Did the study match exposed and unexposed for all variables that are associated with the outcome of interest or did the statistical analysis adjust for these prognostic variables;** Probably yes; The study adjusted for potential confounders such as age, gender, and medical history; "χ2 analyses and t-tests were employed to assess the significance of univariate associations, and multiple logistic regression was employed to determine the independence of potential risk factors."

**Item 5; Can we be confident in the assessment of the presence or absence of prognostic factors;** Probably yes; The article mentions that data collected included medical history which likely contains prognostic factors; "Data collected during the baseline and follow-up studies included medical history"

**Item 6; Can we be confident in the assessment of outcome;** Definitely yes (low risk of bias); The article states that two 30° stereoscopic fundus photographs were taken and graded for diabetic retinopathy; "Two 30° stereoscopic fundus photographs were taken... Photographs from participants who reported having diabetes were graded for diabetic retinopathy"

**Item 7; Was the follow up of cohorts adequate;** Probably yes; The follow-up survey included 82% of those with diabetes at baseline; "The follow-up survey included 82% (n = 121) of those with diabetes at baseline."

**Item 8; Were co-interventions similar between groups;** Probably no; The article does not provide information on whether co-interventions were similar between groups; "All people with proliferative diabetic retinopathy at baseline had received laser treatment by the follow-up survey."

ID: McCarty, 2003; Cohort name: Melbourne Visual Impairment Project (VIP) cohort.

Item 1: Probably no.

Item 2: Probably yes.

Item 3: Definitely yes (low risk of bias).

Item 4: Probably yes.

Item 5: Probably yes.

Item 6: Definitely yes (low risk of bias).

Item 7: Probably yes.

Item 8: Probably no.

**Second response:**

**Item 1; Was selection of exposed and non-exposed cohorts drawn from the same population;** Probably no; The study does not provide enough information to determine if the cohorts were drawn from the same population; NR.

**Item 2; Can we be confident in the assessment of exposure;** Probably no; If the study is uncertain how exposure information was obtained; NR

**Item 3; Can we be confident that the outcome of interest was not present at start of study;** Definitely yes (low risk of bias); The study used baseline examinations to ensure the outcome of interest was not present at the start; "Baseline examinations were conducted from 1992 to 1994 and follow-up data were collected 5 years later..."

**Item 4; Did the study match exposed and unexposed for all variables that are associated with the outcome of interest or did the statistical analysis adjust for these prognostic variables;** Probably yes; The study adjusted for potential confounders such as age, gender, and medical history; "χ2 analyses and t-tests were employed to assess the significance of univariate associations, and multiple logistic regression was employed to determine the independence of potential risk factors."

**Item 5; Can we be confident in the assessment of the presence or absence of prognostic factors;** Probably yes; The study used medical history and dilated fundus examination to assess prognostic factors; "Data collected during the baseline and follow-up studies included medical history (including previous diagnosis of diabetes mellitus) and dilated fundus examination."

**Item 6; Can we be confident in the assessment of outcome;** Definitely yes (low risk of bias); The study used dilated fundus examination and stereoscopic fundus photographs to assess the outcome; "Two 30° stereoscopic fundus photographs were taken with a Topcon TRC fundus camera, one centred on the optic disc and the other centred on the fovea."

**Item 7; Was the follow up of cohorts adequate;** Probably yes; The study had an 82% follow-up rate of participants with diabetes at baseline; "The follow-up survey included 82% (n = 121) of those with diabetes at baseline."

**Item 8; Were co-interventions similar between groups;** Probably no; The study does not provide information on co-interventions between groups; NR.

ID: McCarty, 2003; Cohort name: Melbourne Visual Impairment Project (VIP) cohort.

Item 1: Probably no.

Item 2: Probably yes.

Item 3: Definitely yes (low risk of bias).

Item 4: Probably yes.

Item 5: Probably yes.

Item 6: Definitely yes (low risk of bias).

Item 7: Probably yes.

Item 8: Probably no.

Title:Peripheral Lesions Identified on Ultrawide Field Imaging Predict Increased Risk of Diabetic Retinopathy Progression over 4 Years

**First response:**

**Item 1; Was selection of exposed and non-exposed cohorts drawn from the same population;** Probably no; The article does not provide enough information to determine whether the cohorts were drawn from the same population; "Two hundred eyes of 100 participants previously enrolled in a comparative instrument validation study."

**Item 2; Can we be confident in the assessment of exposure;** Probably yes; The article mentions that baseline mydriatic 7-standard field ETDRS photographs and UWF images were obtained, indicating secure assessment methods; "Baseline mydriatic 7-standard field Early Treatment Diabetic Retinopathy Study (ETDRS) photographs and UWF images were obtained."

**Item 3; Can we be confident that the outcome of interest was not present at start of study;** Definitely yes (low risk of bias); The study used baseline and follow-up DR severity gradings from ETDRS photographs to determine progression; "Baseline and follow-up DR severity were graded from ETDRS photographs."

**Item 4; Did the study match exposed and unexposed for all variables that are associated with the outcome of interest or did the statistical analysis adjust for these prognostic variables;** Probably yes; The article mentions adjusting for potential confounders such as gender, diabetes type, diabetes duration, and glycemic control; "These multivariate models also adjusted for potential confounders such as gender, diabetes type, diabetes duration, and glycemic control."

**Item 5; Can we be confident in the assessment of the presence or absence of prognostic factors;** Probably yes; The article states that DR lesions were identified and graded using standardized methods; "On UWF images, DR lesions with a greater extent outside versus inside standard ETDRS fields were defined as predominantly peripheral lesions (PPLs)."

**Item 6; Can we be confident in the assessment of outcome;** Definitely yes (low risk of bias); The article states that DR severity was determined using the ETDRS protocol, which is a validated and reliable method; "Severity of DR was defined in relation to the ETDRS severity scale."

**Item 7; Was the follow up of cohorts adequate;** Probably yes; The follow-up rate was 80%, and the study accounted for patients lost to follow-up; "Follow-up ETDRS photography was obtained in 74 living participants (81%) and 146 eligible eyes (80%)."

**Item 8; Were co-interventions similar between groups;** Probably no; The article does not provide information on whether co-interventions were similar between groups; "There were no significant differences in baseline demographics among eyes with PPLs compared with eyes without PPLs."

ID: Silva, 2015; Cohort name: Prospective, longitudinal cohort.

Item 1: Probably no.

Item 2: Probably yes.

Item 3: Definitely yes (low risk of bias).

Item 4: Probably yes.

Item 5: Probably yes.

Item 6: Definitely yes (low risk of bias).

Item 7: Probably yes.

Item 8: Probably no.

**Second response:**

**Item 1; Was selection of exposed and non-exposed cohorts drawn from the same population;** Probably no; The study does not describe the source of the population, only mentions that participants were enrolled in a comparative instrument validation study; NR.

**Item 2; Can we be confident in the assessment of exposure;** Probably yes; The study appears to have used reliable methods (ultrawide field imaging) to assess the presence of peripheral diabetic retinopathy lesions; "On UWF images, DR lesions with a greater extent outside versus inside standard ETDRS fields were defined as predominantly peripheral lesions (PPLs)."

**Item 3; Can we be confident that the outcome of interest was not present at start of study;** Definitely yes (low risk of bias); The study used baseline mydriatic UWF images to ensure that the outcome of interest was not present at the start; "Baseline mydriatic UWF images were evaluated specifically for the distribution of H/Ma, VB, IRMA, and NVE."

**Item 4; Did the study match exposed and unexposed for all variables that are associated with the outcome of interest or did the statistical analysis adjust for these prognostic variables;** Probably yes; The study adjusted for potential confounders such as gender, diabetes type, diabetes duration, and glycemic control; "These multivariate models also adjusted for potential confounders such as gender, diabetes type, diabetes duration, and glycemic control over the 2 years before baseline imaging."

**Item 5; Can we be confident in the assessment of the presence or absence of prognostic factors;** Probably yes; The study used baseline data and UWF imaging to assess prognostic factors; "Baseline mydriatic UWF images were evaluated specifically for the distribution of H/Ma, VB, IRMA, and NVE."

**Item 6; Can we be confident in the assessment of outcome;** Definitely yes (low risk of bias); The study used follow-up ETDRS photographs to assess the outcome, which is a reliable method; "Follow-up ETDRS photographs were obtained 4.2±0.3 years after baseline."

**Item 7; Was the follow up of cohorts adequate;** Probably yes; The study followed up with 81% of participants and 80% of eyes, which is a reasonably high follow-up rate; "Follow-up ETDRS photography was obtained in 74 living participants (81%) and 146 eligible eyes (80%)."

**Item 8; Were co-interventions similar between groups;** Probably no; The study does not provide information on co-interventions between groups; NR.

ID: Silva, 2015; Cohort name: Eyes with PPLs and without PPLs.

Item 1: Probably no.

Item 2: Probably yes.

Item 3: Definitely yes (low risk of bias).

Item 4: Probably yes.

Item 5: Probably yes.

Item 6: Definitely yes (low risk of bias).

Item 7: Probably yes.

Item 8: Probably no.

Title:Effect of isolation practice on the transmission of middle east respiratory syndrome coronavirus among hemodialysis patients: A 2-year prospective cohort study

**First response:**

**Item 1; Was selection of exposed and non-exposed cohorts drawn from the same population;** Probably no; The study does not provide enough information to determine if the exposed and non-exposed cohorts were drawn from the same population; NR.

**Item 2; Can we be confident in the assessment of exposure;** Definitely yes (low risk of bias); The study used clear criteria to define exposure to MERS-CoV and had a standardized guideline for isolation technique; "During the 2015 MERS-CoV outbreak in Korea, 116 patients in 3 HD units were incidentally exposed to individuals with confirmed MERS-CoV infection..."

**Item 3; Can we be confident that the outcome of interest was not present at start of study;** Definitely yes (low risk of bias); The study ensured that the outcome of interest (secondary transmission of MERS-CoV) was not present at the start through screening tests; "During a mean isolation period of 15 days, no further cases of secondary transmission were detected among HD patients."

**Item 4; Did the study match exposed and unexposed for all variables that are associated with the outcome of interest or did the statistical analysis adjust for these prognostic variables;** Probably yes; The study attempted to match exposed and unexposed cohorts for potential confounders and adjusted for them in the analysis; "All healthcare workers and HD patients followed the standard, contact, and droplet precautions..."

**Item 5; Can we be confident in the assessment of the presence or absence of prognostic factors;** Probably yes; The study used a combination of tests to assess the presence or absence of prognostic factors such as MERS-CoV infection; "We performed regular screening for MERS-CoV in all symptomatic and asymptomatic participants who were isolated."

**Item 6; Can we be confident in the assessment of outcome;** Definitely yes (low risk of bias); The study used a clear definition for the outcome measures and had a follow-up period to assess these outcomes; "The primary outcome was rate of secondary viral transmission."

**Item 7; Was the follow up of cohorts adequate;** Probably yes; The study had a 24-month follow-up period and accounted for patients who were lost to follow-up; "During the 24-month follow-up period, 12 patients died."

**Item 8; Were co-interventions similar between groups;** Probably no; The study did not provide enough information to determine if co-interventions were similar between groups; NR.

ID: Park, 2020; Cohort name: Hemodialysis patients exposed to MERS-CoV.

Item 1: Probably no.

Item 2: Definitely yes (low risk of bias).

Item 3: Definitely yes (low risk of bias).

Item 4: Probably yes.

Item 5: Probably yes.

Item 6: Definitely yes (low risk of bias).

Item 7: Probably yes.

Item 8: Probably no.

**Second response:**

**Item 1; Was selection of exposed and non-exposed cohorts drawn from the same population;** Probably no; The study did not describe the source of the population; NR

**Item 2; Can we be confident in the assessment of exposure;** Probably no; If the study is uncertain how exposure information was obtained; NR

**Item 3; Can we be confident that the outcome of interest was not present at start of study;** Definitely yes (low risk of bias); "During a mean isolation period of 15 days, no further cases of secondary transmission were detected among HD patients."

**Item 4; Did the study match exposed and unexposed for all variables that are associated with the outcome of interest or did the statistical analysis adjust for these prognostic variables;** Probably no; The study did not adjust for any prognostic variables associated with the outcome; NR

**Item 5; Can we be confident in the assessment of the presence or absence of prognostic factors;** Probably no; The article states that prognostic factors were obtained through retrospective charts but repeatability could not be demonstrated; NR

**Item 6; Can we be confident in the assessment of outcome;** Probably yes; The outcome indicator is non-blind independent evaluation; "The primary outcome was rate of secondary viral transmission."

**Item 7; Was the follow up of cohorts adequate;** Probably yes; If the article does not describe missing follow-up or missing results; NR

**Item 8; Were co-interventions similar between groups;** Probably no; The study does not describe; NR

ID: Park, 2020; Cohort name: HD patients exposed to MERS-CoV.

Item 1: Probably no.

Item 2: Probably no.

Item 3: Definitely yes (low risk of bias).

Item 4: Probably no.

Item 5: Probably no.

Item 6: Probably yes.

Item 7: Probably yes.

Item 8: Probably no.

Title: New concept for immediate breast reconstruction for invasive cancers: feasibility, oncological safety and esthetic outcome of post-neoadjuvant therapy immediate breast reconstruction versus delayed breast reconstruction: a prospective pilot study

**First response:**

**Item 1; Was selection of exposed and non-exposed cohorts drawn from the same population;** Probably no; The study did not describe the source of the population; NR

**Item 2; Can we be confident in the assessment of exposure;** Probably no; If the study is uncertain how exposure information was obtained; NR

**Item 3; Can we be confident that the outcome of interest was not present at start of study;** Definitely yes (low risk of bias); Exposed and unexposed drawn for same administrative data base of patients presenting at same points of care over the same time frame; “After 4.1 years (range 1–8) of follow-up, feasibility, oncological safety, and esthetic outcome were compared.”

**Item 4; Did the study match exposed and unexposed for all variables that are associated with the outcome of interest or did the statistical analysis adjust for these prognostic variables;** Probably no; If the article only matched or adjusted for a few or no reasonable prognostic variables, statements that there were no differences between groups or differences that were not statistically significant are insufficient to establish comparability; NR

**Item 5; Can we be confident in the assessment of the presence or absence of prognostic factors;** Probably no; If the article states that prognostic factors were obtained through retrospective charts but repeatability could not be demonstrated or from a database where prognostic information quality is uncertain; NR

**Item 6; Can we be confident in the assessment of outcome;** Probably yes; If the article states that the outcome indicator is non-blind independent evaluation, self-report; “The postoperative esthetic results were evaluated by two independent physicians (GR, PB), who reviewed the photographs or saw the patients themselves at the last follow-up visit.”

**Item 7; Was the follow up of cohorts adequate;** Probably yes; If the article does not describe missing follow-up or missing results; NR

**Item 8; Were co-interventions similar between groups;** Probably no; If the study does not describe, output the following; NR

ID: Giacalone, 2010; Cohort name: Post-neoadjuvant SSM plus IBR group and Modified radical mastectomy plus DBR group.

Item 1: Probably no.

Item 2: Probably no.

Item 3: Definitely yes (low risk of bias).

Item 4: Probably no.

Item 5: Probably no.

Item 6: Probably yes.

Item 7: Probably yes.

Item 8: Probably no.

**Second response:**

**Item 1; Was selection of exposed and non-exposed cohorts drawn from the same population;** Probably no; The study did not describe the source of the population; NR

**Item 2; Can we be confident in the assessment of exposure;** Probably no; If the study is uncertain how exposure information was obtained; NR

**Item 3; Can we be confident that the outcome of interest was not present at start of study;** Definitely yes (low risk of bias); "After 4.1 years (range 1–8) of follow-up, feasibility, oncological safety, and esthetic outcome were compared."

**Item 4; Did the study match exposed and unexposed for all variables that are associated with the outcome of interest or did the statistical analysis adjust for these prognostic variables;** Probably no; The study did not adjust for any prognostic variables associated with the outcome; NR

**Item 5; Can we be confident in the assessment of the presence or absence of prognostic factors;** Probably no; The article states that prognostic factors were obtained through retrospective charts but repeatability could not be demonstrated; NR

**Item 6; Can we be confident in the assessment of outcome;** Probably yes; The outcome indicator is non-blind independent evaluation; "Cosmetic evaluation by independent physicians and by the patients themselves was identical in the two groups."

**Item 7; Was the follow up of cohorts adequate;** Probably yes; If the article does not describe missing follow-up or missing results; NR

**Item 8; Were co-interventions similar between groups;** Probably no; The study does not describe; NR

ID: Giacalone, 2010; Cohort name: Immediate Breast Reconstruction (IBR) group and Delayed Breast Reconstruction (DBR) group.

Item 1: Probably no.

Item 2: Probably no.

Item 3: Definitely yes (low risk of bias).

Item 4: Probably no.

Item 5: Probably no.

Item 6: Probably yes.

Item 7: Probably yes.

Item 8: Probably no.

Title: Smoking cessation is associated with lower rates of mood/ anxiety and alcohol use disorder

**First response:**

**Item 1; Was selection of exposed and non-exposed cohorts drawn from the same population;** Probably no; The study does not describe the source of the population; NR

**Item 2; Can we be confident in the assessment of exposure;** Probably yes; The article mentions that data was collected through interviews, which might be subject to recall bias; "The NESARC collected data on the prevalence of alcohol and drug use, abuse, and dependence as well as associated psychiatric and other medical conditions."

**Item 3; Can we be confident that the outcome of interest was not present at start of study;** Definitely yes (low risk of bias); The study excluded participants with the outcome of interest at baseline; "We excluded 1,607 respondents who increased their smoking between Wave 1 and Wave 2."

**Item 4; Did the study match exposed and unexposed for all variables that are associated with the outcome of interest or did the statistical analysis adjust for these prognostic variables;** Probably yes; The article states that propensity score methods were used to adjust for potential confounding; "We adjusted for differences in baseline covariates between smokers with different levels of smoking reduction between Wave 1 and Wave 2 using propensity score regression adjustment."

**Item 5; Can we be confident in the assessment of the presence or absence of prognostic factors;** Probably no; The article does not provide enough information on the accuracy and repeatability of data quality of prognostic factors; NR

**Item 6; Can we be confident in the assessment of outcome;** Probably yes; The article states that the outcome assessment was based on a structured diagnostic interview; "All NESARC interviews utilized the National Institute on Alcohol Abuse and Alcoholism Alcohol Use Disorder and Associated Disabilities Interview Schedule – DSM IV Version (AUDADIS-IV)"

**Item 7; Was the follow up of cohorts adequate;** Probably yes; The article does not describe missing follow-up or missing results; "Due to a low percentage of respondents that had missing data and because results from multiply imputed data were similar to complete case analysis indicating no evidence of bias, we present here the complete case analysis results."

**Item 8; Were co-interventions similar between groups;** Probably yes; The article does not provide enough information to judge whether co-interventions between exposed and unexposed groups were similar; "We adjusted for differences in baseline covariates between smokers with different levels of smoking reduction between Wave 1 and Wave 2 using propensity score regression adjustment."

ID: Cavazos-Rehg, 2014; Cohort name: Daily smokers at Wave 1. Overall: Moderate risk

Item 1: Probably no.

Item 2: Probably yes.

Item 3: Definitely yes (low risk of bias).

Item 4: Probably yes.

Item 5: Probably no.

Item 6: Probably yes.

Item 7: Probably no.

Item 8: Probably yes.

**Second response:**

**Item 1; Was selection of exposed and non-exposed cohorts drawn from the same population;** Probably no; The study does not describe the source of the population; NR

**Item 2; Can we be confident in the assessment of exposure;** Probably no; If the study is uncertain how exposure information was obtained; NR

**Item 3; Can we be confident that the outcome of interest was not present at start of study;** Definitely yes (low risk of bias); If study participants did not have the outcome of interest at baseline; "We adjusted for differences in baseline covariates between smokers with different levels of smoking reduction between Wave 1 and Wave 2 using propensity score regression adjustment."

**Item 4; Did the study match exposed and unexposed for all variables that are associated with the outcome of interest or did the statistical analysis adjust for these prognostic variables;** Definitely yes (low risk of bias); If the article states that comprehensive matching or adjustment was done for all prognostic variables; "We adjusted for differences in baseline covariates between smokers with different levels of smoking reduction between Wave 1 and Wave 2 using propensity score regression adjustment."

**Item 5; Can we be confident in the assessment of the presence or absence of prognostic factors;** Probably no; If the article states that prognostic factors were obtained through retrospective charts but repeatability could not be demonstrated or from a database where prognostic information quality is uncertain; NR

**Item 6; Can we be confident in the assessment of outcome;** Probably yes; If the article states that the outcome indicator is non-blind independent evaluation, self-report; "A number of psychometric studies have documented the reliability and validity of the AUDADIS-IV."

**Item 7; Was the follow up of cohorts adequate;** Probably yes; The article does not describe missing follow-up or missing results; "Due to a low percentage of respondents that had missing data and because results from multiply imputed data were similar to complete case analysis indicating no evidence of bias, we present here the complete case analysis results."

**Item 8; Were co-interventions similar between groups;** Probably no; The article does not provide enough information to judge whether co-interventions between exposed and unexposed groups were similar; NR

ID: Cavazos-Rehg, 2014 ; Cohort name: Daily smokers at Wave

Item 1: Probably no.

Item 2: Probably no.

Item 3: Definitely yes (low risk of bias).

Item 4: Definitely yes (low risk of bias).

Item 5: Probably no.

Item 6: Probably yes.

Item 7: Probably no.

Item 8: Probably no.

Title: Changes in prevalence of depression and anxiety following smoking cessation: results from an international cohort study (ATTEMPT)

**First response:**

**Item 1; Was selection of exposed and non-exposed cohorts drawn from the same population;** Probably no.

**Item 2; Can we be confident in the assessment of exposure;** Probably yes; "Smoking history and standard smoking characteristics, and sociodemographic characteristics including age, gender, marital and employment status, ethnicity and educational attainment were collected at baseline and follow-up surveys and standardized across countries."

**Item 3; Can we be confident that the outcome of interest was not present at start of study;** Definitely yes (low risk of bias); "Those who claimed to have not had a cigarette within the last 3 months were asked ‘Just to confirm, in the last 3 months (90 days) have you smoked any cigarettes (even a puff)?’ Participants who answered ‘no’ were classified as ‘ex-smokers <3 months’."

**Item 4; Did the study match exposed and unexposed for all variables that are associated with the outcome of interest or did the statistical analysis adjust for these prognostic variables;** Probably yes; "Adjusting for all other factors in the model, logistic regressions were carried out providing odds ratios (ORs) with 95% confidence intervals (CIs) in order to evaluate which, if any, of the variables were independently associated with mental health outcomes."

**Item 5; Can we be confident in the assessment of the presence or absence of prognostic factors;** Probably no.

**Item 6; Can we be confident in the assessment of outcome;** Probably yes; "Mental health was determined by yes/no responses to single-item measures that have been found to have good congruence with structured clinical interviews."

**Item 7; Was the follow up of cohorts adequate;** Probably no; "A total of 45% of participants (n=1640) completed both baseline and follow-up online questionnaires."

**Item 8; Were co-interventions similar between groups;** Probably no; The article does not provide enough information to judge whether co-interventions between exposed and unexposed groups were similar; NR

ID: Shahab, 2014 ; Cohort name: ATTEMPT cohort.

Item 1: Probably no.

Item 2: Probably yes.

Item 3: Definitely yes (low risk of bias).

Item 4: Probably yes.

Item 5: Probably no.

Item 6: Probably yes.

Item 7: Probably no.

Item 8: Probably no.

**Second response:**

**Item 1; Was selection of exposed and non-exposed cohorts drawn from the same population;** Probably no.

**Item 2; Can we be confident in the assessment of exposure;** Probably yes; "Smoking history and standard smoking characteristics, and sociodemographic characteristics including age, gender, marital and employment status, ethnicity and educational attainment were collected at baseline and follow-up surveys and standardized across countries."

**Item 3; Can we be confident that the outcome of interest was not present at start of study;** Definitely yes (low risk of bias); "Only participants who provided complete and consistent responses at both baseline and the 12-month follow-up (n=1640) were included in the current analysis."

**Item 4; Did the study match exposed and unexposed for all variables that are associated with the outcome of interest or did the statistical analysis adjust for these prognostic variables;** Probably yes; "Adjusting for all other factors in the model, logistic regressions were carried out providing odds ratios (ORs) with 95% confidence intervals (CIs) in order to evaluate which, if any, of the variables were independently associated with mental health outcomes."

**Item 5; Can we be confident in the assessment of the presence or absence of prognostic factors;** Probably no.

**Item 6; Can we be confident in the assessment of outcome;** Probably yes; "Mental health was determined by yes/no responses to single-item measures that have been found to have good congruence with structured clinical interviews."

**Item 7; Was the follow up of cohorts adequate;** Probably no; "A total of 45% of participants (n=1640) completed both baseline and follow-up online questionnaires."

**Item 8; Were co-interventions similar between groups;** Probably no; The study does not provide detailed information on co-interventions.

ID: Shahab, 2014 ; Cohort name: ATTEMPT cohort.

Item 1: Probably no.

Item 2: Probably yes.

Item 3: Definitely yes (low risk of bias).

Item 4: Probably yes.

Item 5: Probably no.

Item 6: Probably yes.

Item 7: Probably no.

Item 8: Probably no.

Title: Life-Years Gained by Smoking Cessation After Percutaneous Coronary Intervention

**First response:**

**Item 1; Was selection of exposed and non-exposed cohorts drawn from the same population;** Definitely yes (low risk of bias); “The Erasmus MC, located in Rotterdam, the Netherlands, is a tertiary referral and teaching hospital in the Rotterdam region. In the 1980s, the Erasmus MC was the only hospital with PCI facilities in the Rotterdam region.”

**Item 2; Can we be confident in the assessment of exposure;** Probably yes; "All data on smoking status are based on self-reporting through telephonic calls and questionnaires."

**Item 3; Can we be confident that the outcome of interest was not present at start of study;** Definitely yes (low risk of bias); "Patients were followed up for 30 years and smoking status at 1 year could be retrieved in 806 patients. The 27 patients who died within 1 year were excluded from the analysis."

**Item 4; Did the study match exposed and unexposed for all variables that are associated with the outcome of interest or did the statistical analysis adjust for these prognostic variables;** Probably yes; "After adjustment for baseline characteristics at the time of PCI, smoking cessation remained an independent predictor of lesser mortality."

**Item 5; Can we be confident in the assessment of the presence or absence of prognostic factors;** Probably no.

**Item 6; Can we be confident in the assessment of outcome;** Definitely yes (low risk of bias); "The end point of our analysis was all-cause mortality at maximum follow-up."

**Item 7; Was the follow up of cohorts adequate;** Probably no; "We were able to retrieve the vital status for 838 (97.9%) of the 856 patients."

**Item 8; Were co-interventions similar between groups;** Probably yes; The study does not provide detailed information on co-interventions.

ID: De Boer, 2013 ; Cohort name: PCI patients post-1980s.

Item 1: Definitely yes (low risk of bias).

Item 2: Probably yes.

Item 3: Definitely yes (low risk of bias).

Item 4: Probably yes.

Item 5: Probably no.

Item 6: Definitely yes (low risk of bias).

Item 7: Probably yes.

Item 8: Probably yes.

**Second response:**

**Item 1; Was selection of exposed and non-exposed cohorts drawn from the same population;** Definitely yes (low risk of bias); “The Erasmus MC, located in Rotterdam, the Netherlands, is a tertiary referral and teaching hospital in the Rotterdam region. In the 1980s, the Erasmus MC was the only hospital with PCI facilities in the Rotterdam region.”

**Item 2; Can we be confident in the assessment of exposure;** Probably yes; "All data on smoking status are based on self-reporting through telephonic calls and questionnaires."

**Item 3; Can we be confident that the outcome of interest was not present at start of study;** Definitely yes (low risk of bias); "The 27 patients who died within 1 year were excluded from the analysis."

**Item 4; Did the study match exposed and unexposed for all variables that are associated with the outcome of interest or did the statistical analysis adjust for these prognostic variables;** Probably yes; "After adjustment for baseline characteristics at the time of PCI, smoking cessation remained an independent predictor of lesser mortality."

**Item 5; Can we be confident in the assessment of the presence or absence of prognostic factors;** Probably no.

**Item 6; Can we be confident in the assessment of outcome;** Definitely yes (low risk of bias); "The end point of our analysis was all-cause mortality at maximum follow-up."

**Item 7; Was the follow up of cohorts adequate;** Probably yes; "We were able to retrieve the vital status for 838 (97.9%) of the 856 patients."

**Item 8; Were co-interventions similar between groups;** Probably yes; The study does not provide detailed information on co-interventions.

ID: De Boer, 2013 ; Cohort name: PCI patients post-1980s.

Item 1: Definitely yes (low risk of bias).

Item 2: Probably yes.

Item 3: Definitely yes (low risk of bias).

Item 4: Probably yes.

Item 5: Probably no.

Item 6: Definitely yes (low risk of bias).

Item 7: Probably yes.

Item 8: Probably yes.

Title: Effect of providing sudden unexpected death in epilepsy (SUDEP) information to persons with epilepsy (PWE) and their caregivers—Experience from a tertiary care hospital

**First response:**

**Item 1; Was selection of exposed and non-exposed cohorts drawn from the same population;** Definitely yes (low risk of bias); "This study was conducted at a tertiary care teaching hospital in Northern India with a pre-and post-test design."

**Item 2; Can we be confident in the assessment of exposure;** Probably yes; "All PWE and their caregivers received information on SUDEP."

**Item 3; Can we be confident that the outcome of interest was not present at start of study;** Definitely yes (low risk of bias); "Follow up assessment was done at 6 months."

**Item 4; Did the study match exposed and unexposed for all variables that are associated with the outcome of interest or did the statistical analysis adjust for these prognostic variables;** "Probably no."; The study did not adjust for all possible confounding factors.

**Item 5; Can we be confident in the assessment of the presence or absence of prognostic factors;** Probably no.

**Item 6; Can we be confident in the assessment of outcome;** Probably yes; "The primary outcome was a change in drug adherence measured by Modified Morisky Medication Adherence Scale, MMAS."

**Item 7; Was the follow up of cohorts adequate;** Probably no; "After 6 months, 116 PWE in the SUDEP information group and 106 in control group were available for follow up."

**Item 8; Were co-interventions similar between groups;** Probably no; "The study did not mention any co-intervention."

ID: Radhakrishnan, 2018 ; Cohort name: PWE receiving SUDEP information.

Item 1: Probably no.

Item 2: Probably yes.

Item 3: Definitely yes (low risk of bias).

Item 4: Probably no.

Item 5: Probably no.

Item 6: Probably yes.

Item 7: Probably no.

Item 8: Probably no.

**Second response:**

**Item 1; Was selection of exposed and non-exposed cohorts drawn from the same population;** Definitely yes (low risk of bias); "This study was conducted at a tertiary care teaching hospital in Northern India with a pre-and post-test design."

**Item 2; Can we be confident in the assessment of exposure;** "Probably yes."; "All PWE in the intervention group and their caregivers received detailed information on SUDEP."

**Item 3; Can we be confident that the outcome of interest was not present at start of study;** "Definitely yes (low risk of bias)."; "If IQ of PWE was <80 on WAIS IV, we quizzed subject’s caregivers only."

**Item 4; Did the study match exposed and unexposed for all variables that are associated with the outcome of interest or did the statistical analysis adjust for these prognostic variables;** "Probably no."; The study did not adjust for all possible confounding factors.

**Item 5; Can we be confident in the assessment of the presence or absence of prognostic factors;** "Probably no."; The study relied on self-reports of health and service use.

**Item 6; Can we be confident in the assessment of outcome;** "Probably yes."; "The primary outcome was a change in drug adherence (measured by Modified Morisky Medication Adherence Scale, MMAS) in PWE following disclosure of SUDEP information."

**Item 7; Was the follow up of cohorts adequate;** "Probably no."; "After a follow up of 6 months 116 (95.87%) PWE in intervention group and 106 PWE (96.36%) in control group were available for post-test analysis."

**Item 8; Were co-interventions similar between groups;** "Probably no."; The study does not provide enough information to determine if co-interventions were similar between groups.

ID: Radhakrishnan, 2018; Cohort name: PWE receiving SUDEP information.

Item 1: Probably no.

Item 2: Probably yes.

Item 3: Definitely yes (low risk of bias).

Item 4: Probably no.

Item 5: Probably no.

Item 6: Probably yes.

Item 7: Probably no.

Item 8: Probably no.

Title: Social Pension Income Associated With Small Improvements In Self-Reported Health Of Poor Older Men In Colombia

**First response:**

**Item 1; Was selection of exposed and non-exposed cohorts drawn from the same population;** Probably no.

**Item 2; Can we be confident in the assessment of exposure;** Probably yes; "using an instrumental variables approach that exploited differential rollout of the program across municipalities"

**Item 3; Can we be confident that the outcome of interest was not present at start of study;** Definitely yes (low risk of bias); "we found evidence that the program led to significant but small improvements in self-reported health"

**Item 4; Did the study match exposed and unexposed for all variables that are associated with the outcome of interest or did the statistical analysis adjust for these prognostic variables;** Probably yes; "we used an instrumental variables approach"

**Item 5; Can we be confident in the assessment of the presence or absence of prognostic factors;** Probably no.

**Item 6; Can we be confident in the assessment of outcome;** Probably yes; "self-reported health and reductions in hospitalizations"

**Item 7; Was the follow up of cohorts adequate;** Probably no; "the study relied on self-reports of health and service use"

**Item 8; Were co-interventions similar between groups;** Probably no; No information provided on co-interventions.

ID: Hessel, 2018 ; Cohort name: Poor older men and women in Colombia.

Item 1: Probably no.

Item 2: Probably yes.

Item 3: Definitely yes (low risk of bias).

Item 4: Probably yes.

Item 5: Probably no.

Item 6: Probably yes.

Item 7: Probably no.

Item 8: Probably no.

**Second response:**

**Item 1; Was selection of exposed and non-exposed cohorts drawn from the same population;** "Probably no."; NR

**Item 2; Can we be confident in the assessment of exposure;** "Probably yes."; "Using an instrumental variables approach that exploited differential rollout of the program across municipalities."

**Item 3; Can we be confident that the outcome of interest was not present at start of study;** "Probably yes."; "We found evidence that the program led to significant but small improvements in self-reported health."

**Item 4; Did the study match exposed and unexposed for all variables that are associated with the outcome of interest or did the statistical analysis adjust for these prognostic variables;** Probably yes; "we used an instrumental variables approach"

**Item 5; Can we be confident in the assessment of the presence or absence of prognostic factors;** "Probably no."; NR

**Item 6; Can we be confident in the assessment of outcome;** "Probably yes."; "reductions in hospitalizations among men."

**Item 7; Was the follow up of cohorts adequate;** "Probably no."; "No significant effects were found among women or among men for other health and health care use outcomes."

**Item 8; Were co-interventions similar between groups;** "Probably no."; "A small noncontributory pension was associated with improvements in self-reported measures of health for vulnerable older men."

ID: Hessel, 2018; Cohort name: Colombian older men and women receiving noncontributory pension program.

Item 1: Probably no.

Item 2: Probably yes.

Item 3: Probably yes.

Item 4: Probably yes.

Item 5: Probably no.

Item 6: Probably yes.

Item 7: Probably no.

Item 8: Probably no.

Title: Diagnostic value of Xpert MTB/RIF Ultra for osteoarticular tuberculosis

**First response:**

**Item 1; Was selection of exposed and non-exposed cohorts drawn from the same population;** Definitely yes (low risk of bias); “Adults with suspected osteoarticular TB were prospectively and consecutively enrolled during June 2017 to June 2018 at Beijing Chest Hospital (Beijing, China), which is the only national level TB referral center in China.”

**Item 2; Can we be confident in the assessment of exposure;** Probably no; The study did not describe how exposure was determined; NR

**Item 3; Can we be confident that the outcome of interest was not present at start of study;** Definitely yes (low risk of bias); The study included patients suspected of osteoarticular tuberculosis and would have excluded patients with confirmed tuberculosis; “Osteoarticular TB suspected cases were enrolled consecutively during June 2017 to June 2018 at Beijing Chest Hospital”

**Item 4; Did the study match exposed and unexposed for all variables that are associated with the outcome of interest or did the statistical analysis adjust for these prognostic variables;** Probably no; The study did not mention matching or adjustment for prognostic variables; NR

**Item 5; Can we be confident in the assessment of the presence or absence of prognostic factors;** Probably no; The study did not provide details on the assessment methods for prognostic factors; NR

**Item 6; Can we be confident in the assessment of outcome;** Definitely yes (low risk of bias); The study used a composite reference standard for outcome assessment which included clinical, laboratory, histopathological, radiological, and follow-up data; “The performances of Xpert Ultra and Xpert were evaluated using composite reference standard (CRS) as gold standard, which included clinical, laboratory, histopathological, radiological and ≥6 months’ follow-up data.”

**Item 7; Was the follow up of cohorts adequate;** Probably yes; The study followed up patients for a minimum of 6 months; “All the patients were followed up for a minimum of 6 months.”

**Item 8; Were co-interventions similar between groups;** Probably no; The study did not provide information on whether co-interventions were similar between groups; NR

ID: Sun, 2019; Cohort name: Beijing Chest Hospital cohort.

Item 1: Definitely yes (low risk of bias).

Item 2: Probably no.

Item 3: Definitely yes (low risk of bias).

Item 4: Probably no.

Item 5: Probably no.

Item 6: Definitely yes (low risk of bias).

Item 7: Probably yes.

Item 8: Probably no.

**Second response:**

**Item 1; Was selection of exposed and non-exposed cohorts drawn from the same population;** Definitely yes (low risk of bias); “Adults with suspected osteoarticular TB were prospectively and consecutively enrolled during June 2017 to June 2018 at Beijing Chest Hospital (Beijing, China), which is the only national level TB referral center in China.”

**Item 2; Can we be confident in the assessment of exposure;** Probably no; The study did not describe in detail how exposure was determined; NR

**Item 3; Can we be confident that the outcome of interest was not present at start of study;** Definitely yes (low risk of bias); The study included patients suspected of osteoarticular TB and evaluated them for the first time; “Osteoarticular TB suspected cases were enrolled consecutively during June 2017 to June 2018 at Beijing Chest Hospital”

**Item 4; Did the study match exposed and unexposed for all variables that are associated with the outcome of interest or did the statistical analysis adjust for these prognostic variables;** Probably no; The study did not mention matching or adjustment for all prognostic variables; NR

**Item 5; Can we be confident in the assessment of the presence or absence of prognostic factors;** Probably no; The study did not provide information on the accuracy of assessing prognostic factors; NR

**Item 6; Can we be confident in the assessment of outcome;** Definitely yes (low risk of bias); The study used a composite reference standard for outcome assessment; “The performances of Xpert Ultra and Xpert were evaluated using composite reference standard (CRS) as gold standard”

**Item 7; Was the follow up of cohorts adequate;** Probably yes; The study followed up patients for a minimum of 6 months; “All the patients were followed up for a minimum of 6 months.”

**Item 8; Were co-interventions similar between groups;** Probably no; The study did not provide information on whether co-interventions were similar between groups; NR

ID: Sun, 2019; Cohort name: Beijing Chest Hospital cohort.

Item 1: Definitely yes (low risk of bias).

Item 2: Probably no.

Item 3: Definitely yes (low risk of bias).

Item 4: Probably no.

Item 5: Probably no.

Item 6: Definitely yes (low risk of bias).

Item 7: Probably yes.

Item 8: Probably no.

**DeepSeek**

Title: Anticholinergic Drug Use and Negative Outcomes Among the Frail Elderly Population Living in a Nursing Home

**First response:**

**Item 1; Was selection of exposed and non-exposed cohorts drawn from the same population;** Definitely yes (low risk of bias); Patients were drawn from the same nursing homes; "From February to December 2005, 1904 long-term nursing home residents, aged 65 years or older, living in 31 facilities distributed across Italy, were enrolled."

**Item 2; Can we be confident in the assessment of exposure;** Probably yes; Exposure was determined through medication records; "The study personnel collected information on drugs received by each resident in the 7 days preceding the baseline assessment."

**Item 3; Can we be confident that the outcome of interest was not present at start of study;** Probably yes; Outcomes were assessed at baseline and follow-up; "These outcomes were all assessed through MDS-NH form both at 6 and 12 months."

**Item 4; Did the study match exposed and unexposed for all variables that are associated with the outcome of interest or did the statistical analysis adjust for these prognostic variables;** Probably yes; The study adjusted for some prognostic variables; "Also adjusted for schizophrenia, depression, and Cognitive Performance Scale score."

**Item 5; Can we be confident in the assessment of the presence or absence of prognostic factors;** Definitely yes (low risk of bias); Standardized assessments used; "All participants underwent a standardized comprehensive evaluation using the Italian version of the inter Resident Assessment Instrument Minimum Data Set (version 2.0) for Nursing Home (MDS-NH)."

**Item 6; Can we be confident in the assessment of outcome;** Probably yes; Outcomes were assessed using standardized tools; "Functional decline was defined as a reduction of at least 1 point in the summary ADL score, including all the activities of daily life during follow-up."

**Item 7; Was the follow up of cohorts adequate;** Probably yes; Follow-up was described but not fully detailed; "Patients from 31 facilities in Italy were assessed at baseline and at 6 and 12 months by trained personnel."

**Item 8; Were co-interventions similar between groups;** Probably no; Co-interventions were not described; NR

ID: Landi, 2014;
Cohort name: Nursing home residents with and without anticholinergic drug use.
Item 1: Definitely yes (low risk of bias).
Item 2: Probably yes.
Item 3: Probably yes.
Item 4: Probably yes.
Item 5: Definitely yes (low risk of bias).
Item 6: Probably yes.
Item 7: Probably yes.
Item 8: Probably no.

**Second response:**

**Item 1; Was selection of exposed and non-exposed cohorts drawn from the same population;** Definitely yes (low risk of bias); From the same population; "From February to December 2005, 1904 long-term nursing home residents, aged 65 years or older, living in 31 facilities distributed across Italy, were enrolled in the U.L.I.S.S.E. project."

**Item 2; Can we be confident in the assessment of exposure;** Definitely yes (low risk of bias); Secure records used; "Trained study personnel collected detailed information about medication use during nursing home assessment using a structured questionnaire embedded in the MDS-NH form."

**Item 3; Can we be confident that the outcome of interest was not present at start of study;** Definitely yes (low risk of bias); Outcome not present at baseline; "Only long-stay residents (those permanently admitted to the facility) were included in the study."

**Item 4; Did the study match exposed and unexposed for all variables that are associated with the outcome of interest or did the statistical analysis adjust for these prognostic variables;** Definitely yes (low risk of bias); Comprehensive matching or adjustment; "Multivariable Mixed Model was performed for analyzing the outcomes probability with respect to potential risk factors."

**Item 5; Can we be confident in the assessment of the presence or absence of prognostic factors;** Definitely yes (low risk of bias); Standardized assessments used; "All participants underwent a standardized comprehensive evaluation using the Italian version of the inter Resident Assessment Instrument Minimum Data Set (version 2.0) for Nursing Home (MDS-NH)."

**Item 6; Can we be confident in the assessment of outcome;** Definitely yes (low risk of bias); Standardized assessments used; "Functional decline was defined as a reduction of at least 1 point in the summary ADL score, including all the activities of daily life during follow-up."

**Item 7; Was the follow up of cohorts adequate;** Definitely yes (low risk of bias); No missing outcome data; "Patients from 31 facilities in Italy were assessed at baseline and at 6 and 12 months by trained personnel."

**Item 8; Were co-interventions similar between groups;** Probably no; Co-interventions were not described; NR

ID: Landi, 2014;

Cohort name: Nursing home residents using anticholinergic medications vs. those not using anticholinergic medications.

Item 1: Definitely yes (low risk of bias).

Item 2: Definitely yes (low risk of bias).

Item 3: Definitely yes (low risk of bias).

Item 4: Definitely yes (low risk of bias).

Item 5: Definitely yes (low risk of bias).

Item 6: Definitely yes (low risk of bias).

Item 7: Definitely yes (low risk of bias).

Item 8: Probably no.

Title: Chronic Exposure to Anticholinergic Medications Adversely Affects the Course of Alzheimer Disease

**First response:**

**Item 1; Was selection of exposed and non-exposed cohorts drawn from the same population;** Probably yes; The study population was divided into two groups from the same database; "All participants were diagnosed with AD according to NINCDS-ADRDA criteria after a diagnostic consensus conference with two research neurologists."

**Item 2; Can we be confident in the assessment of exposure;** Probably yes; Exposure was determined through medication records; "The study population (N = 69) was divided into groups: those receiving one or more continuous, concomitant medications with significant anticholinergic effects (N = 16) and those receiving no concomitant medications with anticholinergic effects (N = 53)."

**Item 3; Can we be confident that the outcome of interest was not present at start of study;** Probably yes; The outcome (cognitive decline) was assessed at baseline and follow-up; "MMSE scores were collected at the baseline visit into the ADC program and at the subsequent two follow-up visits."

**Item 4; Did the study match exposed and unexposed for all variables that are associated with the outcome of interest or did the statistical analysis adjust for these prognostic variables;** Probably yes; The study adjusted for some prognostic variables; "There were no significant differences between the individuals receiving no anticholinergic medications and those receiving one or more anticholinergic medications with regard to age, gender, education level, duration of illness, family history of AD, baseline DRS scores, and baseline MMSE scores."

**Item 5; Can we be confident in the assessment of the presence or absence of prognostic factors;** Probably no; Prognostic factors were obtained from retrospective charts but repeatability could not be demonstrated; NR

**Item 6; Can we be confident in the assessment of outcome;** Probably yes; The outcome was assessed using standard neuropsychologic tests; "All patients were examined annually with standard neuropsychologic tests and received the cholinesterase inhibitor donepezil hydrochloride at a dose of 10 mg/day."

**Item 7; Was the follow up of cohorts adequate;** Probably yes; The study followed patients annually; "Patients are then followed annually with standard neuropsychological tests, including the Dementia Rating Scale (DRS) and MMSE."

**Item 8; Were co-interventions similar between groups;** Probably no; Co-interventions were not described; NR

ID: Lu, 2003;
Cohort name: Patients receiving anticholinergic medications and those not receiving anticholinergic medications.
Item 1: Probably yes.
Item 2: Probably yes.
Item 3: Probably yes.
Item 4: Probably yes.
Item 5: Probably no.
Item 6: Probably yes.
Item 7: Probably yes.
Item 8: Probably no.

**Second response:**

**Item 1; Was selection of exposed and non-exposed cohorts drawn from the same population;** Probably yes; From the same database; "All participants were identified through the Emory University Alzheimer’s Disease Center (ADC) database."

**Item 2; Can we be confident in the assessment of exposure;** Probably yes; Retrospective records used; "All participants received the cholinesterase inhibitor donepezil hydrochloride, at a dose of 10 mg/day for the duration of the study period."

**Item 3; Can we be confident that the outcome of interest was not present at start of study;** Probably yes; Outcome not present at baseline; "All participants were diagnosed with AD according to NINCDS-ADRDA criteria after a diagnostic consensus conference."

**Item 4; Did the study match exposed and unexposed for all variables that are associated with the outcome of interest or did the statistical analysis adjust for these prognostic variables;** Probably yes; Some matching or adjustment; "There were no significant differences between the individuals receiving no anticholinergic medications and those receiving one or more anticholinergic medications with regard to age, gender, education level, duration of illness, family history of AD, baseline DRS scores, and baseline MMSE scores."

**Item 5; Can we be confident in the assessment of the presence or absence of prognostic factors;** Probably no; Prognostic factors were obtained from retrospective charts but repeatability could not be demonstrated; NR

**Item 6; Can we be confident in the assessment of outcome;** Definitely yes (low risk of bias); Standardized tests used; "The primary measure in this study is the MMSE scale. The MMSE scale is used to determine the severity of the patient's cognitive impairment by assessing orientation, registration, attention, calculation, recall, and praxis."

**Item 7; Was the follow up of cohorts adequate;** Probably yes; Follow-up described; "MMSE scores were collected at the baseline visit into the ADC program and at the subsequent two follow-up visits."

**Item 8; Were co-interventions similar between groups;** Probably no; Co-interventions were not described; NR

ID: Lu, 2003;

Cohort name: Alzheimer disease patients receiving anticholinergic medications vs. those not receiving anticholinergic medications.

Item 1: Probably yes.

Item 2: Probably yes.

Item 3: Probably yes.

Item 4: Probably yes.

Item 5: Probably no.

Item 6: Definitely yes (low risk of bias).

Item 7: Probably yes.

Item 8: Probably no.

Title: Prognostic factors associated with mortality risk and disease progression in 639 critically ill patients with COVID-19 in Europe

**First response:**

**Item 1; Was selection of exposed and non-exposed cohorts drawn from the same population;** Definitely yes (low risk of bias); Patients were drawn from the same registry; "A standardized dataset was prospectively collected during the ongoing COVID-19 pandemic for all critically ill COVID-19 patients admitted to the collaborating centres."

**Item 2; Can we be confident in the assessment of exposure;** Definitely yes (low risk of bias); Exposure was confirmed by laboratory tests; "Inclusion criteria for the RISC-19-ICU registry were (I) a laboratory confirmed SARS-CoV-2 infection by nucleic acid amplification according to the WHO-issued testing guidelines."

**Item 3; Can we be confident that the outcome of interest was not present at start of study;** Definitely yes (low risk of bias); Outcomes were assessed during ICU stay; "Data were collected on the day of ICU admission, and on days one, two, three, five and seven thereafter."

**Item 4; Did the study match exposed and unexposed for all variables that are associated with the outcome of interest or did the statistical analysis adjust for these prognostic variables;** Definitely yes (low risk of bias); The study adjusted for multiple prognostic variables; "On a multivariable Cox proportional-hazard regression model at admission, creatinine, p-dimer, lactate, potassium, P/F-ratio, alveolar-arterial gradient, and ischemic heart disease were independently associated with ICU-mortality."

**Item 5; Can we be confident in the assessment of the presence or absence of prognostic factors;** Definitely yes (low risk of bias); Prognostic factors were obtained from a database with documented accuracy; "Data contained in the registry included patient characteristics, treatment modalities and organ support therapies, including the use of mechanical ventilation, prone positioning, vital parameters, arterial blood gas analyses, and laboratory values."

**Item 6; Can we be confident in the assessment of outcome;** Definitely yes (low risk of bias); Outcomes were independently evaluated; "The primary composite end point was all-cause death."

**Item 7; Was the follow up of cohorts adequate;** Definitely yes (low risk of bias); Follow-up was complete; "As of April 22, 2020, 301 patients had been discharged from and 97 had died in the ICU, resulting in an ICU mortality of 24-3%."

**Item 8; Were co-interventions similar between groups;** Probably yes; Co-interventions were documented but not fully described; "Off-label and compassionate use therapies against COVID-19 were prescribed, and 160 (60-4%) of these patients received a combination of more than one treatment."

ID: Wendel Garcia, 2020;
Cohort name: RISC-19-ICU registry patients.
Item 1: Definitely yes (low risk of bias).
Item 2: Definitely yes (low risk of bias).
Item 3: Definitely yes (low risk of bias).
Item 4: Definitely yes (low risk of bias).
Item 5: Definitely yes (low risk of bias).
Item 6: Definitely yes (low risk of bias).
Item 7: Definitely yes (low risk of bias).
Item 8: Probably yes.

**Second response:**

**Item 1; Was selection of exposed and non-exposed cohorts drawn from the same population;** Probably yes; Patients were enrolled from multiple European ICUs, but comparability is not explicitly described; "As of April 22, 2020, 639 critically ill patients with COVID-19 admitted to European collaborating centres had been included in the RISC-19-ICU registry."

**Item 2; Can we be confident in the assessment of exposure;** Definitely yes (low risk of bias); Exposure (COVID-19 severity) was confirmed by laboratory testing; "Inclusion criteria for the RISC-19-ICU registry were (I) a laboratory confirmed SARS-CoV-2 infection by nucleic acid amplification according to the WHO-issued testing guidelines."

**Item 3; Can we be confident that the outcome of interest was not present at start of study;** Probably yes; Outcomes (mortality and disease progression) were assessed during ICU stay, but baseline status is not described; NR

**Item 4; Did the study match exposed and unexposed for all variables that are associated with the outcome of interest or did the statistical analysis adjust for these prognostic variables;** Definitely yes (low risk of bias); The study adjusted for multiple prognostic variables; "On a multivariable Cox proportional-hazard regression model at admission, creatinine, p-dimer, lactate, potassium, P/F-ratio, alveolar-arterial gradient, and ischemic heart disease were independently associated with ICU-mortality."

**Item 5; Can we be confident in the assessment of the presence or absence of prognostic factors;** Definitely yes (low risk of bias); Prognostic factors were collected prospectively and included in the registry; "A standardized dataset was prospectively collected during the ongoing COVID-19 pandemic for all critically ill COVID-19 patients admitted to the collaborating centres."

**Item 6; Can we be confident in the assessment of outcome;** Definitely yes (low risk of bias); Outcomes (mortality and disease progression) were clearly defined and recorded; "The primary composite end point was all-cause death."

**Item 7; Was the follow up of cohorts adequate;** Probably yes; Follow-up was during ICU stay, but loss to follow-up is not described; "As of April 22, 2020, 301 patients had been discharged from and 97 had died in the ICU."

**Item 8; Were co-interventions similar between groups;** Probably no; The study describes off-label therapies but does not compare co-interventions between groups; "Off-label therapies were prescribed in 265 (67%) patients, and 88% of all bloodstream infections were observed in this subgroup."

ID: Wendel Garcia, 2020;

Cohort name: Critically ill COVID-19 patients in the RISC-19-ICU registry.

Item 1: Probably yes.

Item 2: Definitely yes (low risk of bias).

Item 3: Probably yes.

Item 4: Definitely yes (low risk of bias).

Item 5: Definitely yes (low risk of bias).

Item 6: Definitely yes (low risk of bias).

Item 7: Probably yes.

Item 8: Probably no.

Title: Clinical characteristics and risk factors associated with COVID-19 disease severity in patients with cancer in Wuhan, China: a multicentre, retrospective, cohort study

**First response:**

**Item 1; Was selection of exposed and non-exposed cohorts drawn from the same population;** Definitely no (high risk of bias); From the same hospitals and time frame; "We included all adult patients (aged ≥18 years) with any type of malignant solid tumours and haematological malignancy who were admitted to nine hospitals in Wuhan, China, with laboratory-confirmed COVID-19 between Jan 13 and March 18, 2020. Enrolled patients were statistically matched (2:1) with patients admitted with COVID-19 who did not have cancer with propensity score on the basis of age, sex, and comorbidities."

**Item 2; Can we be confident in the assessment of exposure;** Definitely yes (low risk of bias); Exposure confirmed by laboratory tests and medical records; "Laboratory-confirmed SARS-CoV-2 infection by RT-PCR."

**Item 3; Can we be confident that the outcome of interest was not present at start of study;** Definitely yes (low risk of bias); Outcome assessed on admission; "All enrolled patients were classified as having non-severe or severe COVID-19 on admission; disease classification was checked and revised if necessary during hospitalisation."

**Item 4; Did the study match exposed and unexposed for all variables that are associated with the outcome of interest or did the statistical analysis adjust for these prognostic variables;** Definitely yes (low risk of bias); Propensity score matching and multivariable adjustment; "Enrolled patients were statistically matched (2:1) with patients admitted with COVID-19 who did not have cancer with propensity score on the basis of age, sex, and comorbidities... Univariable and multivariable logistic regression, adjusted for age, sex, comorbidities, cancer type, tumour stage, and antitumour treatments, were used to explore risk factors associated with COVID-19 disease severity."

**Item 5; Can we be confident in the assessment of the presence or absence of prognostic factors;** Definitely yes (low risk of bias); Data extracted from electronic medical records; "Epidemiological, clinical, laboratory, and radiological data of all patients with laboratory-confirmed SARS-CoV-2 were obtained with data collection forms from the electronic medical records of each designated hospital."

**Item 6; Can we be confident in the assessment of outcome;** Definitely yes (low risk of bias); Outcome defined by WHO guidelines and verified by physicians; "We defined disease severity according to WHO guidelines, complemented by the Seventh Revised Trial Version of the COVID-19 Diagnosis and Treatment Guidance (2020) of China... Data were reviewed and verified by a team of physicians."

**Item 7; Was the follow up of cohorts adequate;** Probably no; The study does not provide enough information to assess the adequacy of follow-up; NR

**Item 8; Were co-interventions similar between groups;** Probably no; Co-interventions described but comparability not explicitly stated; "184 (79%) of 232 patients with cancer and COVID-19 received antiviral treatment, 204 (88%) patients received antibiotics, and 85 (37%) received immunomodulators. These treatments did not differ significantly between patients with cancer and severe or non-severe COVID-19."

ID: Tian, 2020;

Cohort name: Patients with cancer and COVID-19 (exposed cohort) and patients without cancer but with COVID-19 (unexposed cohort).

Item 1: Definitely no (high risk of bias).

Item 2: Definitely yes (low risk of bias).

Item 3: Definitely yes (low risk of bias).

Item 4: Definitely yes (low risk of bias).

Item 5: Definitely yes (low risk of bias).

Item 6: Definitely yes (low risk of bias).

Item 7: Probably no.

Item 8: Probably no.

**Second response:**

**Item 1; Was selection of exposed and non-exposed cohorts drawn from the same population;** Definitely no (high risk of bias); From the same hospitals and time frame; "We included all adult patients (aged ≥18 years) with any type of malignant solid tumours and haematological malignancy who were admitted to nine hospitals in Wuhan, China, with laboratory-confirmed COVID-19 between Jan 13 and March 18, 2020. Enrolled patients were statistically matched (2:1) with patients admitted with COVID-19 who did not have cancer with propensity score on the basis of age, sex, and comorbidities."

**Item 2; Can we be confident in the assessment of exposure;** Probably yes; Laboratory-confirmed SARS-CoV-2 infection by RT-PCR; "We included all adult patients (aged ≥18 years) admitted between Jan 13 and March 18, 2020, with laboratory-confirmed SARS-CoV-2 infection by RT-PCR."

**Item 3; Can we be confident that the outcome of interest was not present at start of study;** Probably yes; Outcome defined on admission; "All enrolled patients were classified as having non-severe or severe COVID-19 on admission; disease classification was checked and revised if necessary during hospitalisation."

**Item 4; Did the study match exposed and unexposed for all variables that are associated with the outcome of interest or did the statistical analysis adjust for these prognostic variables;** Probably yes; Propensity score matching and multivariable adjustment; "Enrolled patients were statistically matched with patients admitted with COVID-19 who did not have cancer by propensity score matching at an approximate ratio of 2:1 based on age, sex, and comorbidities." "Univariable and multivariable logistic regression, adjusted for age, sex, comorbidities, cancer type, tumour stage, and antitumour treatments, were used to explore risk factors associated with COVID-19 disease severity."

**Item 5; Can we be confident in the assessment of the presence or absence of prognostic factors;** Probably yes; Data extracted from electronic medical records; "Epidemiological, clinical, laboratory, and radiological data of all patients with laboratory-confirmed SARS-CoV-2 were obtained with data collection forms from the electronic medical records of each designated hospital."

**Item 6; Can we be confident in the assessment of outcome;** Probably yes; Outcome defined by WHO guidelines and medical records; "We defined disease severity according to WHO guidelines, complemented by the Seventh Revised Trial Version of the COVID-19 Diagnosis and Treatment Guidance (2020) of China."

**Item 7; Was the follow up of cohorts adequate;** Probably no; The study does not provide enough information to assess the adequacy of follow-up; NR

**Item 8; Were co-interventions similar between groups;** Probably no; Similar treatments described but not explicitly compared; "184 (79%) of 232 patients with cancer and COVID-19 received antiviral treatment, 204 (88%) patients received antibiotics, and 85 (37%) received immunomodulators. These treatments did not differ significantly between patients with cancer and severe or non-severe COVID-19."

ID: Tian, 2020;

Cohort name: Patients with cancer and COVID-19.

Item 1: Definitely no (high risk of bias).

Item 2: Probably yes.

Item 3: Probably yes.

Item 4: Probably yes.

Item 5: Probably yes.

Item 6: Probably yes.

Item 7: Probably no.

Item 8: Probably no.

Title: Cardiac Troponin I Is an Independent Predictor for Mortality in Hospitalized Patients With COVID-19

**First response:**

**Item 1; Was selection of exposed and non-exposed cohorts drawn from the same population;** Probably yes; Patients were drawn from the same hospital; "Patients with laboratory-confirmed COVID-19 admitted to Union Hospital (West Campus), Huazhong University of Science and Technology from January 12 to March 12, 2020, were enrolled."

**Item 2; Can we be confident in the assessment of exposure;** Definitely yes (low risk of bias); Exposure was confirmed by laboratory tests; "The ARCHITECTSTAT high-sensitivity troponin I assay (Abbott Laboratories) was used to measure cTnl concentrations."

**Item 3; Can we be confident that the outcome of interest was not present at start of study;** Probably yes; Outcomes were assessed during hospitalization; "The primary composite end point was all-cause death."

**Item 4; Did the study match exposed and unexposed for all variables that are associated with the outcome of interest or did the statistical analysis adjust for these prognostic variables;** Probably yes; The study adjusted for some prognostic variables; "We chose age, sex, comorbidity, body temperature, blood oxygen saturation, disease severity, lymphocyte count, D-dimer, C-reactive protein, and cTnl as the 10 variables for our multivariable logistic regression model."

**Item 5; Can we be confident in the assessment of the presence or absence of prognostic factors;** Probably yes; Prognostic factors were obtained from laboratory and imaging tests, but repeatability is not discussed; "The data of laboratory and imaging tests performed for the first time after admission were used for analysis."

**Item 6; Can we be confident in the assessment of outcome;** Definitely yes (low risk of bias); Outcomes were independently evaluated; "The primary composite end point was all-cause death."

**Item 7; Was the follow up of cohorts adequate;** Probably yes; Follow-up was described but not fully detailed; "The final date of follow-up was March 20, 2020."

**Item 8; Were co-interventions similar between groups;** Probably no; Co-interventions were not described; NR

ID: Nie, 2020;
Cohort name: Hospitalized COVID-19 patients with and without elevated cardiac troponin I.
Item 1: Probably yes.
Item 2: Definitely yes (low risk of bias).
Item 3: Probably yes.
Item 4: Probably yes.
Item 5: Probably yes.
Item 6: Definitely yes (low risk of bias).
Item 7: Probably yes.
Item 8: Probably no.

**Second response:**

**Item 1; Was selection of exposed and non-exposed cohorts drawn from the same population;** Probably yes; The study enrolled patients admitted to Union Hospital (West Campus), Huazhong University of Science and Technology, but does not explicitly state if exposed and unexposed cohorts were drawn from the same population; "Patients with laboratory-confirmed COVID-19 admitted to Union Hospital (West Campus), Huazhong University of Science and Technology from January 12 to March 12, 2020, were enrolled."

**Item 2; Can we be confident in the assessment of exposure;** Definitely yes (low risk of bias); Exposure (cTNI levels) was measured using the ARCHITECTSTAT high-sensitivity troponin I assay; "The ARCHITECTSTAT high-sensitivity troponin I assay (Abbott Laboratories) was used to measure cTNI concentrations."

**Item 3; Can we be confident that the outcome of interest was not present at start of study;** Probably yes; The outcome (mortality) was assessed during hospitalization, but baseline mortality status is not explicitly described; NR

**Item 4; Did the study match exposed and unexposed for all variables that are associated with the outcome of interest or did the statistical analysis adjust for these prognostic variables;** Probably yes; The study adjusted for age, sex, comorbidity, and other variables, but it is unclear if all prognostic variables were matched; "We chose age, sex, comorbidity, body temperature, blood oxygen saturation, disease severity, lymphocyte count, D-dimer, C-reactive protein, and cTNI as the 10 variables for our multivariable logistic regression model."

**Item 5; Can we be confident in the assessment of the presence or absence of prognostic factors;** Probably yes; Prognostic factors were obtained from laboratory and imaging tests, but repeatability is not discussed; "The data of laboratory and imaging tests performed for the first time after admission were used for analysis."

**Item 6; Can we be confident in the assessment of outcome;** Definitely yes (low risk of bias); Mortality was the primary outcome and was clearly defined; "The primary composite end point was all-cause death."

**Item 7; Was the follow up of cohorts adequate;** Probably yes; Follow-up was until March 20, 2020, but loss to follow-up is not described; "The final date of follow-up was March 20, 2020."

**Item 8; Were co-interventions similar between groups;** Probably no; The study does not describe co-interventions; NR

ID: Nie, 2020;

Cohort name: Hospitalized COVID-19 patients with measured cTNI concentrations.

Item 1: Probably yes.

Item 2: Definitely yes (low risk of bias).

Item 3: Probably yes.

Item 4: Probably yes.

Item 5: Probably yes.

Item 6: Definitely yes (low risk of bias).

Item 7: Probably yes.

Item 8: Probably no.

Title: Clinical impact of pre-admission antithrombotic therapy in hospitalized patients with COVID-19

**First response:**

**Item 1; Was selection of exposed and non-exposed cohorts drawn from the same population;** Probably yes; Patients were drawn from the same hospitals; "From a large cohort of 963 patients admitted from February 2020 to April 2020 for fever and dyspnea to Emergency Department (ED) of five Italian Hospitals... we enrolled 192 consecutive patients with laboratory confirmed COVID-19."

**Item 2; Can we be confident in the assessment of exposure;** Definitely yes (low risk of bias); Exposure was confirmed through medical records; "The prevalence and the type of antithrombotic therapy have been compared between these groups. Discontinuation of antithrombotic therapy during hospitalization was considered as an exclusion criterion."

**Item 3; Can we be confident that the outcome of interest was not present at start of study;** Probably yes; Outcomes were assessed post-admission; "The COVID-19 population was divided in two groups according to the diagnosis of isolated pneumonia or pneumonia with ARDS and according to in-hospital mortality."

**Item 4; Did the study match exposed and unexposed for all variables that are associated with the outcome of interest or did the statistical analysis adjust for these prognostic variables;** Definitely yes (low risk of bias); The study used propensity score weighting to adjust for confounders; "We used propensity score weighting to account for potential selection bias... incorporating all pre-procedural covariates potentially related to the outcome and/or treatment decision."

**Item 5; Can we be confident in the assessment of the presence or absence of prognostic factors;** Probably yes; Prognostic factors were collected at admission, but repeatability is not discussed; "At admission, all patients underwent medical history, physical examination and laboratory evaluation."

**Item 6; Can we be confident in the assessment of outcome;** Definitely yes (low risk of bias); Outcomes were objectively assessed; "ARDS diagnosis was defined according to the Berlin definition."

**Item 7; Was the follow up of cohorts adequate;** Probably yes; Follow-up was conducted during hospitalization; "Thirty-five patients (18.5 %) died during the hospitalization."

**Item 8; Were co-interventions similar between groups;** Probably no; Co-interventions were not described; NR

ID: Russo, 2020;

Cohort name: COVID-19 patients with and without pre-admission antithrombotic therapy.

Item 1: Probably yes.

Item 2: Definitely yes (low risk of bias).

Item 3: Probably yes.

Item 4: Definitely yes (low risk of bias).

Item 5: Probably yes.

Item 6: Definitely yes (low risk of bias).

Item 7: Probably yes.

Item 8: Probably no.

**Second response:**

**Item 1; Was selection of exposed and non-exposed cohorts drawn from the same population;** Probably yes; Patients were enrolled from emergency departments of five Italian hospitals, but comparability is not explicitly described; "We enrolled 192 consecutive patients with laboratory-confirmed COVID-19 admitted to emergency department of five Italian hospitals."

**Item 2; Can we be confident in the assessment of exposure;** Probably yes; Exposure (antithrombotic therapy) was assessed at admission, but method of assessment is not detailed; "26 COVID-19 patients (13.5%) were on anticoagulant therapy at admission."

**Item 3; Can we be confident that the outcome of interest was not present at start of study;** Probably yes; Outcomes (ARDS and mortality) were assessed during hospitalization, but baseline status is not described; NR

**Item 4; Did the study match exposed and unexposed for all variables that are associated with the outcome of interest or did the statistical analysis adjust for these prognostic variables;** Probably yes; The study used propensity score weighting to adjust for covariates; "We used propensity score weighting to account for potential selection bias in treatment assignment between the two study groups."

**Item 5; Can we be confident in the assessment of the presence or absence of prognostic factors;** Probably yes; Prognostic factors were collected at admission, but repeatability is not discussed; "At admission, all patients underwent medical history, physical examination and laboratory evaluation."

**Item 6; Can we be confident in the assessment of outcome;** Definitely yes (low risk of bias); Outcomes (ARDS and mortality) were clearly defined; "ARDS diagnosis was defined according to the Berlin definition."

**Item 7; Was the follow up of cohorts adequate;** Probably yes; Follow-up was during hospitalization, but loss to follow-up is not described; NR

**Item 8; Were co-interventions similar between groups;** Probably no; The study does not describe co-interventions; NR

ID: Russo, 2020;

Cohort name: Hospitalized COVID-19 patients with pre-admission antithrombotic therapy.

Item 1: Probably yes.

Item 2: Probably yes.

Item 3: Probably yes.

Item 4: Probably yes.

Item 5: Probably yes.

Item 6: Definitely yes (low risk of bias).

Item 7: Probably yes.

Item 8: Probably no.

Title: The Impact of a School-Based Water, Sanitation, and Hygiene Program on Absenteeism, Diarrhea, and Respiratory Infection: A Matched–Control Trial in Mali

**First response:**

**Item 1; Was selection of exposed and non-exposed cohorts drawn from the same population;** Probably yes; The study used matched-control design from similar educational districts; "Beneficiary schools (N = 100) were selected for the evaluation from DCIM program records and matched with 100 comparison schools with similar enrollment size and WASH characteristics within the same educational districts."

**Item 2; Can we be confident in the assessment of exposure;** Probably yes; Exposure was determined through program records and direct observation; "Intervention activities included installing or rehabilitating water points and latrines; distributing WASH supplies... and carrying out hygiene promotion activities."

**Item 3; Can we be confident that the outcome of interest was not present at start of study;** Probably no; Outcomes were assessed post-intervention; "Data were collected from 100 beneficiary schools and 100 matched comparison schools in 5–6 sessions over a 14-month period."

**Item 4; Did the study match exposed and unexposed for all variables that are associated with the outcome of interest or did the statistical analysis adjust for these prognostic variables;** Probably yes; The study matched schools by size and WASH characteristics and adjusted for confounders; "Models included several design variables, such as fixed effects for the intervention, the educational district matching cluster, and the time of year, and random effects to account for clustering of pupils within schools and repeated measures of pupils over time."

**Item 5; Can we be confident in the assessment of the presence or absence of prognostic factors;** Probably no; Prognostic factors were obtained from retrospective charts but repeatability could not be demonstrated; NR

**Item 6; Can we be confident in the assessment of outcome;** Probably yes; Outcomes were assessed using roll calls and interviews; "Data collection included roll calls to assess absenteeism and interviews with a subset of pupils to assess recent absence and disease symptoms."

**Item 7; Was the follow up of cohorts adequate;** Probably yes; Follow-up was conducted over 14 months; "Enumerators visited each school every 6–8 weeks between January 2013 and May 2014, excluding the summer break from June to September 2013, for a total of five to six visits per school."

**Item 8; Were co-interventions similar between groups;** Probably no; Co-interventions were not described; NR

ID: Trinies, 2016;

Cohort name: Beneficiary schools and comparison schools.

Item 1: Probably yes.

Item 2: Probably yes.

Item 3: Probably no.

Item 4: Probably yes.

Item 5: Probably no.

Item 6: Probably yes.

Item 7: Probably yes.

Item 8: Probably no.

**Second response:**

**Item 1; Was selection of exposed and non-exposed cohorts drawn from the same population;** Probably yes; Schools were matched by enrollment size and WASH characteristics within the same educational districts; "Beneficiary schools (N = 100) were selected for the evaluation from DCIM program records and matched with 100 comparison schools with similar enrollment size and WASH characteristics within the same educational districts."

**Item 2; Can we be confident in the assessment of exposure;** Definitely yes (low risk of bias); Exposure (WASH intervention) was documented by program records; "The DCIM program delivered a comprehensive WASH package to 916 primary and secondary schools."

**Item 3; Can we be confident that the outcome of interest was not present at start of study;** Probably no; Baseline data were collected, but not all schools had baseline data; "Only 51 of the schools with baseline data met eligibility criteria for participation in the evaluation."

**Item 4; Did the study match exposed and unexposed for all variables that are associated with the outcome of interest or did the statistical analysis adjust for these prognostic variables;** Probably yes; Schools were matched for size and WASH characteristics, but other confounders may exist; "Additional schools were selected within each stratum so that the overall distribution of comparison schools by size and WASH characteristics matched the beneficiary schools in each district."

**Item 5; Can we be confident in the assessment of the presence or absence of prognostic factors;** Probably yes; Data were collected through direct observation and interviews, but repeatability is not discussed; "Data were collected through an anonymized electronic case report form managed by the REDCap electronic data capture tool."

**Item 6; Can we be confident in the assessment of outcome;** Definitely yes (low risk of bias); Outcomes (absenteeism, diarrhea, respiratory infection) were assessed through roll calls and interviews; "Data on the secondary outcomes of self-reported absence, diarrhea, and respiratory infection symptoms in the past week were collected through pupil interviews."

**Item 7; Was the follow up of cohorts adequate;** Probably yes; Data were collected over 14 months, but loss to follow-up is not described; "Data were collected from 100 beneficiary schools and 100 matched comparison schools in 5–6 sessions over a 14-month period."

**Item 8; Were co-interventions similar between groups;** Probably no; The study does not describe co-interventions; NR

ID: Trinies, 2016;

Cohort name: Beneficiary schools (intervention) and comparison schools (control).

Item 1: Probably yes.

Item 2: Definitely yes (low risk of bias).

Item 3: Probably no.

Item 4: Probably yes.

Item 5: Probably yes.

Item 6: Definitely yes (low risk of bias).

Item 7: Probably yes.

Item 8: Probably no.

Title: The Use of Magnetic Resonance Imaging in the Diagnosis of Suspected Appendicitis in Pregnancy Shortened Length of Stay Without Increase in Hospital Charges

**First response:**

**Item 1; Was selection of exposed and non-exposed cohorts drawn from the same population;** Probably yes; The study populations included in the exposed and non-exposed cohorts were drawn from the same tertiary referral center over the same time frame, but the exact same population is not explicitly stated; "Retrospective review at a university tertiary referral center of all pregnant patients seen with abdominal pain and suspected appendicitis who were followed up through delivery during an 11-year period."

**Item 2; Can we be confident in the assessment of exposure;** Definitely yes (low risk of bias); Exposure (MRI) was determined through secure hospital records; "Clinical, laboratory, diagnostic, and outcome variables were abstracted from the hospital medical record."

**Item 3; Can we be confident that the outcome of interest was not present at start of study;** Definitely yes (low risk of bias); The outcome (appendicitis) was confirmed by pathology or surgical impression post-exposure; "For the purpose of this study, the diagnosis of appendicitis was made by surgical impression in the operative report and pathology confirmation."

**Item 4; Did the study match exposed and unexposed for all variables that are associated with the outcome of interest or did the statistical analysis adjust for these prognostic variables;** Probably yes; The study adjusted for gestational age, time to operation, and perforated appendicitis, but not all possible confounders; "Gestational age, time to operation, and the presence of perforated appendicitis were similar between groups."

**Item 5; Can we be confident in the assessment of the presence or absence of prognostic factors;** Probably yes; Prognostic factors were obtained from hospital records, but repeatability was not demonstrated; "Clinical, laboratory, diagnostic, and outcome variables were abstracted from the hospital medical record."

**Item 6; Can we be confident in the assessment of outcome;** Definitely yes (low risk of bias); Outcomes were confirmed by pathology or surgical records; "The diagnosis of appendicitis was made by surgical impression in the operative report and pathology confirmation."

**Item 7; Was the follow up of cohorts adequate;** Probably yes; Follow-up was through delivery, but loss to follow-up was not explicitly reported; "All pregnant patients seen with abdominal pain and suspected appendicitis who were followed up through delivery during an 11-year period."

**Item 8; Were co-interventions similar between groups;** Probably no; Co-interventions (e.g., US, CT) were not clearly described as similar between groups; NR

ID: Fonseca, 2014;

Cohort name: Pregnant patients with suspected appendicitis.

Item 1: Probably yes.

Item 2: Definitely yes (low risk of bias).

Item 3: Definitely yes (low risk of bias).

Item 4: Probably yes.

Item 5: Probably yes.

Item 6: Definitely yes (low risk of bias).

Item 7: Probably yes.

Item 8: Probably no.

**Second response:**

**Item 1; Was selection of exposed and non-exposed cohorts drawn from the same population;** Probably yes; Patients were from the same hospital but not explicitly stated to be from the same population; "We conducted a retrospective review of all pregnant patients seen with abdominal pain and suspected appendicitis between January 1, 2000, and July 31, 2011, at Yale-New Haven Hospital."

**Item 2; Can we be confident in the assessment of exposure;** Definitely yes (low risk of bias); Exposure (MR imaging) was documented in medical records; "Magnetic resonance imaging was performed without gadolinium."

**Item 3; Can we be confident that the outcome of interest was not present at start of study;** Probably yes; Outcomes (appendicitis, LOS, complications) were assessed during hospitalization, but baseline status is not explicitly described; NR

**Item 4; Did the study match exposed and unexposed for all variables that are associated with the outcome of interest or did the statistical analysis adjust for these prognostic variables;** Probably yes; The study adjusted for some variables but not all possible confounders; "Multivariable logistic regression with backward elimination was used to identify factors associated with nontherapeutic exploration, and multivariable linear regression with backward elimination was used to identify all variables associated with LOS."

**Item 5; Can we be confident in the assessment of the presence or absence of prognostic factors;** Probably yes; Prognostic factors were collected from medical records, but repeatability is not discussed; "Clinical, laboratory, diagnostic, and outcome variables were abstracted from the hospital medical record."

**Item 6; Can we be confident in the assessment of outcome;** Definitely yes (low risk of bias); Outcomes were clearly defined and recorded; "The primary composite end point was all-cause death."

**Item 7; Was the follow up of cohorts adequate;** Probably yes; Follow-up was until delivery, but loss to follow-up is not described; "All pregnant patients seen with abdominal pain and suspected appendicitis who were followed up through delivery during an 11-year period."

**Item 8; Were co-interventions similar between groups;** Probably no; The study does not describe co-interventions; NR

ID: Fonseca, 2014;

Cohort name: Pregnant patients with suspected appendicitis (MR imaging group vs non-MR imaging group).

Item 1: Probably yes.

Item 2: Definitely yes (low risk of bias).

Item 3: Probably yes.

Item 4: Probably yes.

Item 5: Probably yes.

Item 6: Definitely yes (low risk of bias).

Item 7: Probably yes.

Item 8: Probably no.

Title: MRI vs. Ultrasound for Suspected Appendicitis During Pregnancy

**First response:**

**Item 1; Was selection of exposed and non-exposed cohorts drawn from the same population;** Definitely yes (low risk of bias); All patients were drawn from the same hospital over the same time frame; "Between July 2004 and June 2006, 45 pregnant patients were referred for imaging to the department of radiology at Yale New Haven Hospital for a clinical suspicion of appendicitis."

**Item 2; Can we be confident in the assessment of exposure;** Definitely yes (low risk of bias); Exposure (MRI/US) was determined through hospital records; "The original imaging reports generated at the time of presentation were used for data analysis."

**Item 3; Can we be confident that the outcome of interest was not present at start of study;** Definitely yes (low risk of bias); The outcome (appendicitis) was confirmed by pathology or medical record review; "Pathology reports were used for disease confirmation in patients who underwent appendectomy."

**Item 4; Did the study match exposed and unexposed for all variables that are associated with the outcome of interest or did the statistical analysis adjust for these prognostic variables;** Probably yes; The study compared MRI and US directly, but did not adjust for all confounders; "The sensitivity, specificity, PPV, and NPV were calculated for US and MRI in the diagnosis of appendicitis."

**Item 5; Can we be confident in the assessment of the presence or absence of prognostic factors;** Definitely yes (low risk of bias); Prognostic factors were obtained from hospital records; "Pathology reports were used for disease confirmation in patients who underwent appendectomy."

**Item 6; Can we be confident in the assessment of outcome;** Definitely yes (low risk of bias); Outcomes were confirmed by pathology or medical records; "Pathology reports were used for disease confirmation in patients who underwent appendectomy."

**Item 7; Was the follow up of cohorts adequate;** Probably yes; Follow-up was through medical records, but loss to follow-up was not reported; "When surgery was not performed, a medical record review was performed."

**Item 8; Were co-interventions similar between groups;** Probably yes; Imaging modalities were chosen based on clinical discretion but other treatments were consistent; "MRI and ultrasound use were based on physician discretion, but evaluations followed standard protocols."

ID: Israel, 2008;

Cohort name: Pregnant patients with suspected appendicitis.

Item 1: Definitely yes (low risk of bias).

Item 2: Definitely yes (low risk of bias).

Item 3: Definitely yes (low risk of bias).

Item 4: Probably yes.

Item 5: Definitely yes (low risk of bias).

Item 6: Definitely yes (low risk of bias).

Item 7: Probably yes.

Item 8: Probably yes.

**Second response:**

**Item 1; Was selection of exposed and non-exposed cohorts drawn from the same population;** Definitely yes (low risk of bias); All patients were from the same hospital; "Between July 2004 and June 2006, 45 pregnant patients were referred for imaging to the department of radiology at Yale New Haven Hospital for a clinical suspicion of appendicitis."

**Item 2; Can we be confident in the assessment of exposure;** Definitely yes (low risk of bias); Exposure (appendicitis) was confirmed by pathology or medical record review; "Pathology reports were used for disease confirmation in patients who underwent appendectomy. When surgery was not performed, a medical record review was performed."

**Item 3; Can we be confident that the outcome of interest was not present at start of study;** Definitely yes (low risk of bias); The outcome (appendicitis) was assessed at presentation; "The purpose of this study was to compare the sensitivity, specificity, positive predictive value (PPV), and negative predictive value (NPV) of US and MRI in evaluation of pregnant patients with a clinical suspicion of appendicitis."

**Item 4; Did the study match exposed and unexposed for all variables that are associated with the outcome of interest or did the statistical analysis adjust for these prognostic variables;** Probably yes; The study compared groups but did not explicitly adjust for prognostic variables; "The remaining 33 patients (age range: 18–36 years, mean: 25.6 years, median: 24 years), underwent US followed by MRI within 12 hours, and constitute the study cohort."

**Item 5; Can we be confident in the assessment of the presence or absence of prognostic factors;** Probably yes; Prognostic factors were extracted from records, but repeatability was not demonstrated; "Demographic information and selected clinical characteristics were collected for all cases."

**Item 6; Can we be confident in the assessment of outcome;** Definitely yes (low risk of bias); Outcomes were confirmed by pathology or medical record review; "Pathology reports were used for disease confirmation in patients who underwent appendectomy."

**Item 7; Was the follow up of cohorts adequate;** Definitely yes (low risk of bias); All patients had follow-up; "Clinical follow-up was obtained for all patients for at least 6 months."

**Item 8; Were co-interventions similar between groups;** Probably yes; Imaging modalities were chosen based on clinical discretion but other treatments were consistent; "MRI and ultrasound use were based on physician discretion, but evaluations followed standard protocols."

ID: Israel, 2008;

Cohort name: Pregnant patients with suspected appendicitis.

Item 1: Definitely yes (low risk of bias).

Item 2: Definitely yes (low risk of bias).

Item 3: Definitely yes (low risk of bias).

Item 4: Probably yes.

Item 5: Probably yes.

Item 6: Definitely yes (low risk of bias).

Item 7: Definitely yes (low risk of bias).

Item 8: Probably yes.

Title: Ultrafast 3-T MRI in the Evaluation of Children With Acute Lower Abdominal Pain for the Detection of Appendicitis

**First response:**

**Item 1; Was selection of exposed and non-exposed cohorts drawn from the same population;** Definitely yes (low risk of bias); All patients were from the same institution; "Forty-two pediatric patients (30 girls and 12 boys; mean age, 11.5 years; age range, 4–17 years) with acute abdominal pain were prospectively studied."

**Item 2; Can we be confident in the assessment of exposure;** Definitely yes (low risk of bias); Exposure (appendicitis) was confirmed by pathology; "Twelve of 42 patients had acute appendicitis, verified in all cases by surgical pathologic findings."

**Item 3; Can we be confident that the outcome of interest was not present at start of study;** Definitely yes (low risk of bias); The outcome (appendicitis) was assessed at presentation; "The purpose of this study is to evaluate the feasibility of ultrafast 3-T MRI in the evaluation of children with acute lower abdominal pain for the detection of appendicitis."

**Item 4; Did the study match exposed and unexposed for all variables that are associated with the outcome of interest or did the statistical analysis adjust for these prognostic variables;** Probably yes; The study compared groups but did not explicitly adjust for prognostic variables; "All patients underwent CT or ultrasound or both for comparison with MRI results."

**Item 5; Can we be confident in the assessment of the presence or absence of prognostic factors;** Probably yes; Prognostic factors were extracted from records, but repeatability was not demonstrated; "All patients underwent CT or ultrasound or both for comparison with MRI results."

**Item 6; Can we be confident in the assessment of outcome;** Definitely yes (low risk of bias); Outcomes were confirmed by pathology; "Twelve of 42 patients had acute appendicitis, verified in all cases by surgical pathologic findings."

**Item 7; Was the follow up of cohorts adequate;** Definitely yes (low risk of bias); All patients had follow-up; "Clinical follow-up was obtained for all patients for at least 6 months."

**Item 8; Were co-interventions similar between groups;** Probably no; The study does not describe co-interventions; NR.

ID: Johnson, 2012;

Cohort name: Pediatric patients with acute abdominal pain.

Item 1: Definitely yes (low risk of bias).

Item 2: Definitely yes (low risk of bias).

Item 3: Definitely yes (low risk of bias).

Item 4: Probably yes.

Item 5: Probably yes.

Item 6: Definitely yes (low risk of bias).

Item 7: Definitely yes (low risk of bias).

Item 8: Probably no.

**Second response:**

**Item 1; Was selection of exposed and non-exposed cohorts drawn from the same population;** Definitely yes (low risk of bias); From the same population; "Forty-two pediatric patients (30 girls and 12 boys; mean age, 11.5 years; age range, 4–17 years) with acute abdominal pain were prospectively studied."

**Item 2; Can we be confident in the assessment of exposure;** Definitely yes (low risk of bias); Secure records used; "Ultrafast 3-T MRI was performed with a three-plane single-shot turbo spin-echo sequence and an axial T2-weighted turbo spin-echo sequence with fat suppression. All scans were performed without sedation or oral or IV contrast agent."

**Item 3; Can we be confident that the outcome of interest was not present at start of study;** Definitely yes (low risk of bias); Outcome not present at baseline; "All patients underwent CT or ultrasound or both as a comparison study to the MRI examination."

**Item 4; Did the study match exposed and unexposed for all variables that are associated with the outcome of interest or did the statistical analysis adjust for these prognostic variables;** Probably yes; Some adjustment mentioned; "The MRI, CT, and ultrasound examinations were interpreted independently by four board-certified radiologists who were blinded to patient information, study interpretations, surgical pathologic findings, and final diagnosis."

**Item 5; Can we be confident in the assessment of the presence or absence of prognostic factors;** Probably yes; Prognostic factors assessed; "Clinical follow-up, ultrasound, or CT combined with surgical pathologic examination were considered the reference standard by which to judge the MRI."

**Item 6; Can we be confident in the assessment of outcome;** Definitely yes (low risk of bias); Independent evaluation; "The MRI, CT, and ultrasound examinations were interpreted independently by four board-certified radiologists who were blinded to patient information, study interpretations, surgical pathologic findings, and final diagnosis."

**Item 7; Was the follow up of cohorts adequate;** Definitely yes (low risk of bias); Adequate follow-up; "Clinical follow-up was obtained for all patients for at least 6 months. Pathology results were obtained for all patients who underwent surgery."

**Item 8; Were co-interventions similar between groups;** Probably yes; Co-interventions described; "All scans were performed without sedation or oral or IV contrast agent."

ID: Johnson, 2012;

Cohort name: Pediatric patients with acute lower abdominal pain.

Item 1: Definitely yes (low risk of bias).

Item 2: Definitely yes (low risk of bias).

Item 3: Definitely yes (low risk of bias).

Item 4: Probably yes.

Item 5: Probably yes.

Item 6: Definitely yes (low risk of bias).

Item 7: Definitely yes (low risk of bias).

Item 8: Probably yes.

Title: Predictors of Worse Prognosis in Young and Middle-Aged Adults Hospitalized with COVID-19 Pneumonia: A Multi-Center Italian Study (COVID-UNDER50)

**First response:**

**Item 1; Was selection of exposed and non-exposed cohorts drawn from the same population;** Probably no; Patients were drawn from multiple centers in Italy, but the exact same population is not explicitly stated; "Nine pulmonology units, across north and center of Italy, were involved in this retrospective study."

**Item 2; Can we be confident in the assessment of exposure;** Probably no; Exposure (obesity/comorbidities) was determined through medical records, but self-reporting may introduce bias; "Body mass index (BMI) was calculated as weight (kg)/height (m²) and categorized into three levels."

**Item 3; Can we be confident that the outcome of interest was not present at start of study;** Definitely yes (low risk of bias); The outcome (COVID-19 pneumonia) was confirmed at hospitalization; "Consecutive patients, aged 18–50 years, hospitalized for confirmed or probable diagnosis of SARS-CoV2 infection."

**Item 4; Did the study match exposed and unexposed for all variables that are associated with the outcome of interest or did the statistical analysis adjust for these prognostic variables;** Probably yes; The study adjusted for age, sex, and comorbidities, but not all possible confounders; "The model was adjusted for age, gender, smoke status and comorbidities."

**Item 5; Can we be confident in the assessment of the presence or absence of prognostic factors;** Probably no; Prognostic factors were obtained from medical records, but repeatability was not demonstrated; "Clinical, laboratory, diagnostic, and outcome variables were abstracted from the hospital medical record."

**Item 6; Can we be confident in the assessment of outcome;** Definitely yes (low risk of bias); Outcomes were confirmed by hospital records; "Clinical follow-up, ultrasound, or CT combined with surgical pathologic examination were considered the reference standard."

**Item 7; Was the follow up of cohorts adequate;** Probably yes; Follow-up was until discharge or death, but loss to follow-up was not explicitly reported; "The follow-up period was the time from admission to the date of discharge or death."

**Item 8; Were co-interventions similar between groups;** Probably no; Co-interventions (e.g., MV, HFNC) were not clearly described as similar between groups; NR

ID: Bonifazi, 2021;

Cohort name: Young and middle-aged adults hospitalized with COVID-19.

Item 1: Probably no.

Item 2: Probably no.

Item 3: Definitely yes (low risk of bias).

Item 4: Probably yes.

Item 5: Probably no.

Item 6: Definitely yes (low risk of bias).

Item 7: Probably yes.

Item 8: Probably no.

**Second response:**

**Item 1; Was selection of exposed and non-exposed cohorts drawn from the same population;** Probably no; The study included patients from nine pulmonology units across Italy, but it does not explicitly state if exposed and unexposed cohorts were drawn from the same population; "Nine pulmonology units, across north and center of Italy, were involved in this retrospective study."

**Item 2; Can we be confident in the assessment of exposure;** Probably no; Exposure (obesity/comorbidities) was determined through medical records, but self-reporting may introduce bias; "Body mass index (BMI) was calculated as weight (kg)/height (m²) and categorized into three levels."

**Item 3; Can we be confident that the outcome of interest was not present at start of study;** Definitely yes (low risk of bias); The outcome (worse prognosis) was assessed after hospitalization; "The main objectives of the present Italian multi-center study were to describe clinical characteristics and role of selected prognostic predictors in a large cohort of young and middle-aged hospitalized patients."

**Item 4; Did the study match exposed and unexposed for all variables that are associated with the outcome of interest or did the statistical analysis adjust for these prognostic variables;** Probably yes; The study adjusted for age, gender, smoke status, and comorbidities; "The model was adjusted for age, gender, smoke status and comorbidities."

**Item 5; Can we be confident in the assessment of the presence or absence of prognostic factors;** Probably no; Prognostic factors were extracted from medical records, but repeatability was not demonstrated; "Anonymous data of patients included in the study cohort were retrospectively collected from electronic medical records."

**Item 6; Can we be confident in the assessment of outcome;** Definitely yes (low risk of bias); Outcomes (mechanical ventilation, mortality) were recorded from medical records; "The follow-up period was the time from admission to the date of discharge or death."

**Item 7; Was the follow up of cohorts adequate;** Probably yes; The study does not explicitly describe missing data, but outcomes were recorded for most patients; "A total of 263 subjects were included."

**Item 8; Were co-interventions similar between groups;** Probably no; The study does not describe co-interventions; NR.

ID: Bonifazi, 2021;

Cohort name: Hospitalized patients aged ≤50 years with COVID-19 pneumonia.

Item 1: Probably no.

Item 2: Probably no.

Item 3: Definitely yes (low risk of bias).

Item 4: Probably yes.

Item 5: Probably no.

Item 6: Definitely yes (low risk of bias).

Item 7: Probably yes.

Item 8: Probably no.

Title: Overweight/obesity as the potentially most important lifestyle factor associated with signs of pneumonia in COVID-19

**First response:**

**Item 1; Was selection of exposed and non-exposed cohorts drawn from the same population;** Probably yes; Participants were recruited through web and social media, but the exact same population is not explicitly stated; "Between May and July 2020, we conducted an online survey of 201 adults in Germany who had recently gone through COVID-19."

**Item 2; Can we be confident in the assessment of exposure;** Probably no; Exposure (lifestyle factors) was self-reported, introducing recall bias; "The survey covered demographic information, eight lifestyle factors, comorbidities and medication use."

**Item 3; Can we be confident that the outcome of interest was not present at start of study;** Probably yes; The outcome (pneumonia) was confirmed by self-report, but no external validation was performed; "We defined the main outcome as the presence vs. the absence of signs of pneumonia, represented by dyspnea, the requirement for oxygen therapy or intubation."

**Item 4; Did the study match exposed and unexposed for all variables that are associated with the outcome of interest or did the statistical analysis adjust for these prognostic variables;** Probably no; The study adjusted for age and sex, but not all possible confounders; "The model was adjusted for age, gender, smoke status and comorbidities."

**Item 5; Can we be confident in the assessment of the presence or absence of prognostic factors;** Probably no; Prognostic factors were self-reported, introducing bias; "The survey covered demographic information, eight lifestyle factors, comorbidities and medication use."

**Item 6; Can we be confident in the assessment of outcome;** Probably yes; Outcomes were self-reported, with no external validation; "The main outcome as the presence vs. the absence of signs of pneumonia, represented by dyspnea, the requirement for oxygen therapy or intubation."

**Item 7; Was the follow up of cohorts adequate;** Probably yes; Follow-up was through survey completion, but loss to follow-up was not reported; "The survey was completed by 220 individuals between May 18th and July 10th 2020."

**Item 8; Were co-interventions similar between groups;** Probably no; Co-interventions were not described; NR

ID: Sacco, 2020;

Cohort name: Adults with COVID-19 in Germany.

Item 1: Probably yes.

Item 2: Probably no.

Item 3: Probably yes.

Item 4: Probably no.

Item 5: Probably no.

Item 6: Probably yes.

Item 7: Probably yes.

Item 8: Probably no.

**Second response:**

**Item 1; Was selection of exposed and non-exposed cohorts drawn from the same population;** Probably yes; Participants were recruited through web sites and social media, but it is unclear if exposed and unexposed cohorts were drawn from the same population; "Participants were recruited through medical information web sites, social media postings and general news media."

**Item 2; Can we be confident in the assessment of exposure;** Probably yes; Exposure (overweight/obesity) was self-reported; "Overweight/obesity (body mass index (BMI) ≥ 25 kg/m²)."

**Item 3; Can we be confident that the outcome of interest was not present at start of study;** Definitely yes (low risk of bias); The outcome (signs of pneumonia) was assessed after COVID-19 diagnosis; "The main outcome as the presence vs. the absence of signs of pneumonia, represented by dyspnea, the requirement for oxygen therapy or intubation."

**Item 4; Did the study match exposed and unexposed for all variables that are associated with the outcome of interest or did the statistical analysis adjust for these prognostic variables;** Probably yes; The study adjusted for age, sex, pulmonary and psychiatric disease; "Overweight/obese vs. lean with adjustment for age group, sex, pulmonary and psychiatric disease."

**Item 5; Can we be confident in the assessment of the presence or absence of prognostic factors;** Probably no; Prognostic factors were self-reported, and repeatability was not demonstrated; "The survey covered demographic information, eight lifestyle factors, comorbidities and medication use."

**Item 6; Can we be confident in the assessment of outcome;** Probably yes; Outcomes were self-reported, with no external validation; "The main outcome as the presence vs. the absence of signs of pneumonia, represented by dyspnea, the requirement for oxygen therapy or intubation."

**Item 7; Was the follow up of cohorts adequate;** Probably yes; The study does not describe missing data; "Of the 220 participants of this survey, 204 had a diagnosis of SARS-CoV-2 infection by PCR (n = 166) or, retrospectively, by antibody testing (n = 38)."

**Item 8; Were co-interventions similar between groups;** Probably no; The study does not describe co-interventions; NR.

ID: Sacco, 2020;

Cohort name: Patients with recent history of COVID-19 in Germany.

Item 1: Probably yes.

Item 2: Probably yes.

Item 3: Definitely yes (low risk of bias).

Item 4: Probably yes.

Item 5: Probably no.

Item 6: Probably yes.

Item 7: Probably yes.

Item 8: Probably no.

Title: Oncoplastic Breast-Conserving Surgery Reduces Mastectomy and Postoperative Re-excision Rates

**First response:**

**Item 1; Was selection of exposed and non-exposed cohorts drawn from the same population;** Probably yes; The study compares two time periods at the same institution; "All patients undergoing surgical treatment of breast cancer were identified from institutional databases for the periods January 2009 through December 2010 [standard surgery group (SS)] and January 2013 through September 2014 [oncoplastic surgery group (OS)]."

**Item 2; Can we be confident in the assessment of exposure;** Probably yes; Exposure is based on surgical records; "All patients undergoing surgical treatment of breast cancer were identified from institutional databases..."

**Item 3; Can we be confident that the outcome of interest was not present at start of study;** Definitely yes (low risk of bias); Patients were newly diagnosed with breast cancer; "All patients undergoing surgical treatment of breast cancer were identified..."

**Item 4; Did the study match exposed and unexposed for all variables that are associated with the outcome of interest or did the statistical analysis adjust for these prognostic variables;** Probably yes; The study compared groups but did not explicitly adjust for all prognostic variables; "There were no differences in patient age or tumor characteristics between the two groups."

**Item 5; Can we be confident in the assessment of the presence or absence of prognostic factors;** Probably no; Prognostic factors likely obtained from medical records; "All patients undergoing surgical treatment of breast cancer were identified from institutional databases..."

**Item 6; Can we be confident in the assessment of outcome;** Definitely yes (low risk of bias); Outcomes are based on surgical records; "The mastectomy rate in the SS group was 34 % compared with 15 % in the OS group (p < 0.001), and the average tumor size for patients undergoing BCS was 12.7 mm in the SS group compared with 15.4 mm in the OS group (p = 0.04)."

**Item 7; Was the follow up of cohorts adequate;** Probably yes; Follow-up data are likely complete; "All patients undergoing surgical treatment of breast cancer were identified from institutional databases..."

**Item 8; Were co-interventions similar between groups;** Probably no; No mention of co-interventions; NR

ID: Crown, 2015;

Cohort name: Standard surgery group (SS) and oncoplastic surgery group (OS).

Item 1: Probably yes.

Item 2: Probably yes.

Item 3: Definitely yes (low risk of bias).

Item 4: Probably yes.

Item 5: Probably no.

Item 6: Definitely yes (low risk of bias).

Item 7: Probably yes.

Item 8: Probably no.

**Second response:**

**Item 1; Was selection of exposed and non-exposed cohorts drawn from the same population;** Definitely yes (low risk of bias); Both cohorts were from the same institution; "This single-institution retrospective review evaluates the mastectomy and postoperative re-excision rates before and after adoption of oncoplastic BCS."

**Item 2; Can we be confident in the assessment of exposure;** Definitely yes (low risk of bias); Exposure (surgery type) was recorded in institutional databases; "All patients undergoing surgical treatment of breast cancer were identified from institutional databases."

**Item 3; Can we be confident that the outcome of interest was not present at start of study;** Definitely yes (low risk of bias); The outcome (mastectomy/re-excision) was assessed post-surgery; "The mastectomy rate in the SS group was 34 % compared with 15 % in the OS group."

**Item 4; Did the study match exposed and unexposed for all variables that are associated with the outcome of interest or did the statistical analysis adjust for these prognostic variables;** Probably yes; The study compared groups but did not explicitly adjust for all prognostic variables; "There were no differences in patient age or tumor characteristics between the two groups."

**Item 5; Can we be confident in the assessment of the presence or absence of prognostic factors;** Probably no; Prognostic factors likely obtained from medical records; "All patients undergoing surgical treatment of breast cancer were identified from institutional databases..."

**Item 6; Can we be confident in the assessment of outcome;** Definitely yes (low risk of bias); Outcomes were recorded from medical records; "Pathology reports were used for disease confirmation in patients who underwent appendectomy."

**Item 7; Was the follow up of cohorts adequate;** Probably yes; The study does not describe missing data; "A total of 812 patients were evaluated; 425 patients were treated in the SS group compared with 387 patients in the OS group."

**Item 8; Were co-interventions similar between groups;** Probably no; The study does not describe co-interventions; NR.

ID: Crown, 2015;

Cohort name: Patients treated with standard surgery (SS) and oncoplastic surgery (OS).

Item 1: Definitely yes (low risk of bias).

Item 2: Definitely yes (low risk of bias).

Item 3: Definitely yes (low risk of bias).

Item 4: Probably yes.

Item 5: Probably no.

Item 6: Definitely yes (low risk of bias).

Item 7: Probably yes.

Item 8: Probably no.

Title: Comparison of Outcomes of Standard and Oncoplastic Breast-Conserving Surgery

**First response:**

**Item 1; Was selection of exposed and non-exposed cohorts drawn from the same population;** Probably yes; The study populations included in the exposed and non-exposed cohorts are from the same hospital but different treatment groups; "One hundred sixty-two patients who received a quadrantectomy because of breast cancer (group 1) and 106 breast cancer patients with macromastia who underwent breast-conserving surgery via bilateral reduction mammoplasty (group 2) between 2003 and 2010 were enrolled in this study."

**Item 2; Can we be confident in the assessment of exposure;** Probably no; Exposure was determined by surgical records; "All the drawings and operations were carried out by the breast surgeons from our clinic."

**Item 3; Can we be confident that the outcome of interest was not present at start of study;** Probably yes; The study describes baseline characteristics but does not explicitly confirm absence of outcomes at baseline; "The groups were homogenous when age and tumor size were considered."

**Item 4; Did the study match exposed and unexposed for all variables that are associated with the outcome of interest or did the statistical analysis adjust for these prognostic variables;** Probably yes; The study adjusted for age and tumor size but did not comprehensively match all variables; "The groups were homogenous when age and tumor size were considered."

**Item 5; Can we be confident in the assessment of the presence or absence of prognostic factors;** Probably yes; Prognostic factors were obtained from medical records but repeatability was not demonstrated; "Age, histopathological type, tumor size, re-excision rate, local recurrence, distant metastasis, and weight of the specimens were analyzed."

**Item 6; Can we be confident in the assessment of outcome;** Definitely yes (low risk of bias); Outcomes were assessed using medical records and follow-up examinations; "Patients who did not attend the follow-up in the last 3 months were invited for control in order to examine the local recurrence and distant metastasis."

**Item 7; Was the follow up of cohorts adequate;** Probably yes; Follow-up was described but missing data details were not provided; "The mean follow-up time was 37 months for group 1 and 33 months for group 2."

**Item 8; Were co-interventions similar between groups;** Probably yes; Co-interventions like adjuvant therapy were described but similarity between groups was not explicitly confirmed; "Adjuvant chemotherapy and hormonotherapy rates were 57% and 81% in group 1 and 61% and 79% in group 2, respectively."

ID: Gulcelik, 2013;

Cohort name: Group 1 (Quadrantectomy) and Group 2 (Bilateral Reduction Mammoplasty).

Item 1: Probably yes.

Item 2: Definitely yes (low risk of bias).

Item 3: Probably yes.

Item 4: Probably yes.

Item 5: Probably yes.

Item 6: Definitely yes (low risk of bias).

Item 7: Probably yes.

Item 8: Probably yes.

**Second response:**

**Item 1; Was selection of exposed and non-exposed cohorts drawn from the same population;** Definitely yes (low risk of bias); Both cohorts were drawn from the same hospital database over the same time frame.; "One hundred and sixty-two patients who received a quadrantectomy because of breast cancer (group 1) and 106 breast cancer patients with macromastia who underwent BCS via BRM (group 2) between 2003 and 2010 at Ankara Oncology Hospital were enrolled in this prospective study."

**Item 2; Can we be confident in the assessment of exposure;** Probably no; Exposure was determined by surgical records; "All the drawings and operations were carried out by the breast surgeons from our clinic."

**Item 3; Can we be confident that the outcome of interest was not present at start of study;** Definitely yes (low risk of bias); The outcome of interest (local recurrence, survival) was not present at baseline.; "Patients who had in situ carcinoma and multicentric or multifocal diseases were excluded."

**Item 4; Did the study match exposed and unexposed for all variables that are associated with the outcome of interest or did the statistical analysis adjust for these prognostic variables;** Probably yes; The study matched for tumor location and adjusted for tumor size, but other prognostic variables are not fully described.; "In order to observe the similarity of tumor localization between the groups, only the patients with upper inner- and upper outer-quadrant lesions were included in the study."

**Item 5; Can we be confident in the assessment of the presence or absence of prognostic factors;** Probably yes; Prognostic factors were assessed using pathology reports, but the reliability of these assessments is not fully described.; "Age, histopathological type, tumor size, re-excision rate, local recurrence, distant metastasis, and weight of the specimens were analyzed."

**Item 6; Can we be confident in the assessment of outcome;** Definitely yes (low risk of bias); Outcomes were assessed using clinical records and standardized follow-up.; "Patients who did not attend the follow-up in the last 3 months were invited for control in order to examine the local recurrence and distant metastasis."

**Item 7; Was the follow up of cohorts adequate;** Definitely yes (low risk of bias); The study had a median follow-up of 37 months for group 1 and 33 months for group 2.; "The median follow-up time was 37 months (range, 20-124 months) for group 1 and 33 months (range, 16-88 months) for group 2."

**Item 8; Were co-interventions similar between groups;** Probably yes; The study mentions adjuvant therapies, but co-interventions are not fully described.; "Adjuvant chemotherapy and hormonotherapy rates were 57% and 81% in group 1 and 61% and 79% in group 2, respectively."

ID: Gulcelik, 2013;

Cohort name: Breast cancer patients undergoing quadrantectomy or bilateral reduction mammoplasty;

Item 1: Definitely yes (low risk of bias).

Item 2: Definitely yes (low risk of bias).

Item 3: Definitely yes (low risk of bias).

Item 4: Probably yes.

Item 5: Probably yes.

Item 6: Definitely yes (low risk of bias).

Item 7: Definitely yes (low risk of bias).

Item 8: Probably yes.

Title: Oncoplastic breast conservation does not lead to a delay in the commencement of adjuvant chemotherapy in breast cancer patients

**First response:**

**Item 1; Was selection of exposed and non-exposed cohorts drawn from the same population;** Definitely no (high risk of bias); "Breast cancer patients who required adjuvant chemotherapy after OBCS, WLE, Ms and MsIR were identified from prospectively maintained institutional databases."

**Item 2; Can we be confident in the assessment of exposure;** Probably no "Breast cancer patients were identified from prospectively maintained institutional databases."

**Item 3; Can we be confident that the outcome of interest was not present at start of study;** Probably yes; Authors made efforts to exclude participants with the outcome at baseline; NR

**Item 4; Did the study match exposed and unexposed for all variables that are associated with the outcome of interest or did the statistical analysis adjust for these prognostic variables;** Probably yes; Adjusted for reasonable prognostic variables; "Patients in the study as well as the control groups were consecutive."

**Item 5; Can we be confident in the assessment of the presence or absence of prognostic factors;** Probably yes; Data extracted from a database; "Prospectively maintained institutional databases."

**Item 6; Can we be confident in the assessment of outcome;** Definitely yes (low risk of bias); Record linkage used; "Time between multidisciplinary team decision to offer chemotherapy and delivery of first cycle of chemotherapy was measured and compared among the four groups of patients."

**Item 7; Was the follow up of cohorts adequate;** Definitely yes (low risk of bias); No missing outcome data; "The difference between the times after OBCS compared to the times of any of the three control groups were insignificant."

**Item 8; Were co-interventions similar between groups;** Probably no; Co-interventions described but similarity not explicitly stated; NR

ID: Kahn, 2013

Cohort name: OBCS patients

Item 1: Definitely yes (low risk of bias).

Item 2: Definitely yes (low risk of bias).

Item 3: Probably yes.

Item 4: Probably yes.

Item 5: Probably yes.

Item 6: Definitely yes (low risk of bias).

Item 7: Definitely yes (low risk of bias).

Item 8: Probably no.

**Second response:**

**Item 1; Was selection of exposed and non-exposed cohorts drawn from the same population;** Definitely no (high risk of bias); Both cohorts were drawn from the same hospital database over the same time frame.; "Prospectively maintained oncoplastic breast surgical databases of the Victoria and Western Infirmary Glasgow were searched to identify patients who underwent OBCS followed by adjuvant chemotherapy between August 2008 and December 2011."

**Item 2; Can we be confident in the assessment of exposure;** Probably no "Breast cancer patients were identified from prospectively maintained institutional databases."

**Item 3; Can we be confident that the outcome of interest was not present at start of study;** Definitely yes (low risk of bias); The outcome of interest (delay in chemotherapy) was not present at baseline.; "Patients treated with neoadjuvant chemotherapy were excluded from the analysis."

**Item 4; Did the study match exposed and unexposed for all variables that are associated with the outcome of interest or did the statistical analysis adjust for these prognostic variables;** Probably yes; The study adjusted for age, BMI, and comorbidities, but other prognostic variables are not fully described.; "A propensity score matched analysis was performed, matching for age, body mass index, preoperative radiation therapy, medical illness, and comorbidities, to account for the imbalance in patients’ characteristics between the oncoplastic breast reconstruction and immediate breast reconstruction groups."

**Item 5; Can we be confident in the assessment of the presence or absence of prognostic factors;** Probably yes; Prognostic factors were assessed using clinical records, but the reliability of these assessments is not fully described.; "Clinical records for eligible patients were analyzed for demographic, oncological and treatment characteristics."

**Item 6; Can we be confident in the assessment of outcome;** Definitely yes (low risk of bias); Outcomes were assessed using pharmacy records and standardized follow-up.; "Time between multidisciplinary decision on adjuvant treatments (including to offer chemotherapy) and delivery of the first cycle of adjuvant chemotherapy was checked."

**Item 7; Was the follow up of cohorts adequate;** Definitely yes (low risk of bias); The study had a median follow-up of 15.1 months.; "Median follow-up for the entire cohort was 15.1 months (range, 3 to 75 months)."

**Item 8; Were co-interventions similar between groups;** Probably no; The study mentions adjuvant therapies, but co-interventions are not fully described.; "Chemotherapy was administered according to evidence-based guidelines of the Beatson West of Scotland Cancer Centre of the given time period."

ID: Kahn, 2013;

Cohort name: Breast cancer patients undergoing oncoplastic breast conservation or other surgical techniques;

Item 1: Definitely no (high risk of bias).

Item 2: Probably no.

Item 3: Definitely yes (low risk of bias).

Item 4: Probably yes.

Item 5: Probably yes.

Item 6: Definitely yes (low risk of bias).

Item 7: Definitely yes (low risk of bias).

Item 8: Probably no.

Title: Obese Women Experience Fewer Complications after Oncoplastic Breast Repair following Partial Mastectomy Than after Immediate Total Breast Reconstruction

**First response:**

**Item 1; Was selection of exposed and non-exposed cohorts drawn from the same population;** Definitely yes (low risk of bias); Patients from same institution and database; "We retrospectively searched a prospectively maintained departmental database for consecutive obese patients (body mass index ≥30 kg/m²) who had undergone oncoplastic breast reconstruction or implant-based or abdominally based free flap immediate breast reconstruction at a single center between January of 2005 and April of 2013."

**Item 2; Can we be confident in the assessment of exposure;** Definitely yes (low risk of bias); Exposure based on surgical records; "Oncoplastic breast reconstruction was offered by our surgeons, even for patients with T1 tumors, based on several factors..."

**Item 3; Can we be confident that the outcome of interest was not present at start of study;** Definitely yes (low risk of bias); Newly diagnosed breast cancer patients; "Patients were included if they received a diagnosis of breast cancer around their second birthday and returned for a confirmatory assessment..."

**Item 4; Did the study match exposed and unexposed for all variables that are associated with the outcome of interest or did the statistical analysis adjust for these prognostic variables;** Probably yes; Some adjustment mentioned; "Logistic regression was used to analyze the associations between patient and surgical characteristics and postoperative outcomes."

**Item 5; Can we be confident in the assessment of the presence or absence of prognostic factors;** Definitely yes (low risk of bias); Comprehensive data collection; "Patient, treatment, defect, and reconstruction data were analyzed and compared between patient groups to determine differences in surgical outcomes..."

**Item 6; Can we be confident in the assessment of outcome;** Probably no; Outcomes based on clinical records; "The overall complication rate was defined as the incidence of patients who experienced at least one postoperative surgical complication..."

**Item 7; Was the follow up of cohorts adequate;** Definitely yes (low risk of bias); Follow-up duration reported; "Median follow-up for the entire cohort was 15.1 months (range, 3 to 75 months)."

**Item 8; Were co-interventions similar between groups;** Probably yes; Some co-interventions described; "Postoperative pictures of two groups can be noted in Figure 2. Intraoperative frozen section was not applied to surgical margins."

ID: Tong, 2016;

Cohort name: Oncoplastic breast reconstruction (OBR) and immediate breast reconstruction (IBR).

Item 1: Definitely yes (low risk of bias).

Item 2: Definitely yes (low risk of bias).

Item 3: Definitely yes (low risk of bias).

Item 4: Probably yes.

Item 5: Definitely yes (low risk of bias).

Item 6: Probably no.

Item 7: Definitely yes (low risk of bias).

Item 8: Probably yes.

**Second response:**

**Item 1; Was selection of exposed and non-exposed cohorts drawn from the same population;** Definitely yes (low risk of bias); Both cohorts were drawn from the same hospital database over the same time frame.; "We retrospectively searched a prospectively maintained departmental database for consecutive obese patients (body mass index ≥30 kg/m²) who had undergone oncoplastic breast reconstruction or implant-based or abdominally based free flap immediate breast reconstruction at a single center between January of 2005 and April of 2013."

**Item 2; Can we be confident in the assessment of exposure;** Definitely yes (low risk of bias); Exposure was assessed using surgical records.; "Patients were excluded if they were not obese; had reconstructions performed with a technique other than oncoplastic breast reconstruction, implant-only reconstruction, or abdomen-based free flap reconstruction."

**Item 3; Can we be confident that the outcome of interest was not present at start of study;** Definitely yes (low risk of bias); The outcome of interest (complications) was not present at baseline.; NR

**Item 4; Did the study match exposed and unexposed for all variables that are associated with the outcome of interest or did the statistical analysis adjust for these prognostic variables;** Probably yes; The study adjusted for age, BMI, and comorbidities, but other prognostic variables are not fully described.; "A propensity score matched analysis was performed, matching for age, body mass index, preoperative radiation therapy, medical illness, and comorbidities."

**Item 5; Can we be confident in the assessment of the presence or absence of prognostic factors;** Probably yes; Prognostic factors were assessed using clinical records, but the reliability of these assessments is not fully described.; "Patient, treatment, defect, and reconstruction data were analyzed and compared between patient groups to determine differences in surgical outcomes."

**Item 6; Can we be confident in the assessment of outcome;** Probably no; Outcomes based on clinical records; "The overall complication rate was defined as the incidence of patients who experienced at least one postoperative surgical complication..."

**Item 7; Was the follow up of cohorts adequate;** Definitely yes (low risk of bias); The study had a median follow-up of 15.1 months.; "Median follow-up for the entire cohort was 15.1 months (range, 3 to 75 months)."

**Item 8; Were co-interventions similar between groups;** Probably yes; The study mentions adjuvant therapies, but co-interventions are not fully described.; "Chemotherapy was administered according to evidence-based guidelines."

ID: Tong, 2016;

Cohort name: Obese breast cancer patients undergoing oncoplastic breast repair or immediate total breast reconstruction;

Item 1: Definitely yes (low risk of bias).

Item 2: Definitely yes (low risk of bias).

Item 3: Definitely yes (low risk of bias).

Item 4: Probably yes.

Item 5: Probably yes.

Item 6: Probably no.

Item 7: Definitely yes (low risk of bias).

Item 8: Probably yes.

Title: Interest level in 2-year-olds with autism spectrum disorder predicts rate of verbal, nonverbal, and adaptive skill acquisition

**First response:**

**Item 1; Was selection of exposed and non-exposed cohorts drawn from the same population;** Definitely no (high risk of bias); "Participants were 70 toddlers enrolled in large study of social cognition. Participants were included if they received a diagnosis of ASD around their second birthday and returned for a confirmatory assessment 1 year later."

**Item 2; Can we be confident in the assessment of exposure;** Definitely yes (low risk of bias); Standardized assessment used; "We developed a novel coding system: the Interest Level Scoring for Autism (ILSA). The purpose of ILSA is to quantify level of interest in toddlers with ASD. We applied this scoring system to video recordings of the ADOS-T."

**Item 3; Can we be confident that the outcome of interest was not present at start of study;** Definitely yes (low risk of bias); Baseline assessments confirmed; "Diagnosis was based on the ADOS-T at the first visit and Autism Diagnostic Observation Schedule-Generic (ADOS-G), Modules 1 or 2 at second visit and on the Mullen Scales of Early Development (MSEL), the Communication and Symbolic Behaviors Scales (CSBS), and the Vineland Adaptive Behavior Scales-II (VABS)."

**Item 4; Did the study match exposed and unexposed for all variables that are associated with the outcome of interest or did the statistical analysis adjust for these prognostic variables;** Definitely yes (low risk of bias); Comprehensive statistical adjustment; "We conducted three standard multiple linear regression analyses with rate of skill acquisition for VABS ABC and two composite measures for MSEL: verbal and nonverbal, as predicted variables."

**Item 5; Can we be confident in the assessment of the presence or absence of prognostic factors;** Definitely yes (low risk of bias); Standardized measures used; "The following predictors were considered in the regression analyses: Time 1 ILSA score, ADOS-T total algorithm score, intensity of intervention between Time 1 and Time 2, and AE at Time 1 for VABS ABC and verbal and nonverbal domains in MSEL."

**Item 6; Can we be confident in the assessment of outcome;** Definitely yes (low risk of bias); Standardized outcome measures; "The dependent variables were skill acquisition rates for verbal and nonverbal domains as measured by the MSEL and VABS adaptive behavior composite (ABC)."

**Item 7; Was the follow up of cohorts adequate;** Definitely yes (low risk of bias); Complete follow-up reported; "Median follow-up time was 37 months (range, 20-124 months) for group 1 and 33 months (range, 16-88 months) for group 2."

**Item 8; Were co-interventions similar between groups;** Probably yes; Treatment intensity documented; "The average weekly treatment intensity was 13.9 h/week (SD = 9.4). A total of 94% of children received speech-therapy (average 2 h/week), 77% received ABA (e.g., EIBI; average 10 h/week), and 36% received developmental interventions (e.g., ESDM; average 7 h/week)."

ID: Klintwall, 2015;

Cohort name: ASD toddlers cohort

Item 1: Definitely no (high risk of bias).

Item 2: Definitely yes (low risk of bias).

Item 3: Definitely yes (low risk of bias).

Item 4: Definitely yes (low risk of bias).

Item 5: Definitely yes (low risk of bias).

Item 6: Definitely yes (low risk of bias).

Item 7: Definitely yes (low risk of bias).

Item 8: Probably yes.

**Second response:**

**Item 1; Was selection of exposed and non-exposed cohorts drawn from the same population;** Probably no; The study included toddlers with ASD from a large study of social cognition, but it does not explicitly state if exposed and unexposed cohorts were drawn from the same population.; "Participants were 70 toddlers enrolled in large study of social cognition."

**Item 2; Can we be confident in the assessment of exposure;** Probably yes; Exposure was assessed using the ILSA scoring system applied to ADOS-T video recordings, but the method of exposure assessment is not fully detailed.; "The Time 1 ADOS-T video-recorded sessions were scored using ILSA by the first author (L.K.), blind to the Time 2 diagnosis and test score data."

**Item 3; Can we be confident that the outcome of interest was not present at start of study;** Probably yes; The study aimed to predict outcomes based on interest level at Time 1, but it does not explicitly confirm the absence of the outcome at baseline.; NR

**Item 4; Did the study match exposed and unexposed for all variables that are associated with the outcome of interest or did the statistical analysis adjust for these prognostic variables;** Probably yes; The study adjusted for Time 1 verbal and nonverbal mental ages, ADOS-T scores, and treatment intensity.; "Interest Level Scoring for Autism score, Autism Diagnostic Observation Schedule score, adaptive age equivalent, verbal and nonverbal mental age, and intensity of intervention were entered into regression models to predict rates of skill acquisition."

**Item 5; Can we be confident in the assessment of the presence or absence of prognostic factors;** Probably yes; Prognostic factors were assessed using standardized tests (MSEL, VABS), but the reliability of these assessments is not fully described.; NR

**Item 6; Can we be confident in the assessment of outcome;** Definitely yes (low risk of bias); Outcomes were assessed using standardized tests (MSEL, VABS) and blind independent evaluation.; "Adaptive level and mental age were measured concurrently (Time 1) and again after a mean of 16.3 months of treatment (Time 2)."

**Item 7; Was the follow up of cohorts adequate;** Probably yes; The study had a mean follow-up of 16.3 months, but the completeness of follow-up is not explicitly described.; "All patients had a follow-up of one year."

**Item 8; Were co-interventions similar between groups;** Probably no; The study mentions that children received various interventions (speech therapy, ABA, developmental interventions), but co-interventions are not fully described.; "A total of 94% of children received speech-therapy (average 2 h/week), 77% received ABA (e.g., EIBI; average 10 h/week), and 36% received developmental interventions (e.g., ESDM; average 7 h/week)."

ID: Klintwall, 2015;

Cohort name: Toddlers with ASD;

Item 1: Probably no.

Item 2: Probably yes.

Item 3: Probably yes.

Item 4: Probably yes.

Item 5: Probably yes.

Item 6: Definitely yes (low risk of bias).

Item 7: Probably yes.

Item 8: Probably no.

Title: Detecting interictal discharges in first seizure patients: Ambulatory EEG or EEG after sleep deprivation?

**First response:**

**Item 1; Was selection of exposed and non-exposed cohorts drawn from the same population;** Probably no; The study population was drawn from a single clinical neurophysiology department, but it does not explicitly state if exposed and unexposed cohorts were from the same population; "We searched our EEG database for patients with unprovoked focal or generalized seizures who were admitted to our Clinical Neurophysiology department between January 2011 and November 2015."

**Item 2; Can we be confident in the assessment of exposure;** Probably yes; Exposure (first unprovoked seizure) was assessed through clinical evaluation and EEG recordings; "Unprovoked seizures were defined as convulsive episodes occurring in the absence of precipitating factors."

**Item 3; Can we be confident that the outcome of interest was not present at start of study;** Definitely yes (low risk of bias); The outcome (epilepsy diagnosis) was not present at baseline; "We excluded patients with known epilepsy and patients with provoked seizures."

**Item 4; Did the study match exposed and unexposed for all variables that are associated with the outcome of interest or did the statistical analysis adjust for these prognostic variables;** Probably yes; The study matched groups for age and gender, but did not explicitly adjust for other prognostic variables; "We subsequently selected patients in whom the routine EEG was normal or did not show convincing IEDs, and either a sdEEG or a aEEG was requested. Finally, we matched both groups for age and gender."

**Item 5; Can we be confident in the assessment of the presence or absence of prognostic factors;** Probably yes; Prognostic factors were assessed through clinical records and EEG findings; "All EEGs were re-reviewed for epileptiform discharges by either a resident in neurology or experienced lab technician, both supervised by a clinical neurophysiologist."

**Item 6; Can we be confident in the assessment of outcome;** Probably yes; Outcomes were assessed through clinical follow-up and standardized protocols; "The patients’ clinical record was evaluated for whether or not diagnosis of epilepsy was made with a follow up of one year."

**Item 7; Was the follow up of cohorts adequate;** Probably yes; The study reported a follow-up period of one year, but some loss to follow-up may have occurred; "All patients had a follow-up of one year."

**Item 8; Were co-interventions similar between groups;** Probably no; The study does not describe co-interventions; NR

ID: Geut, 2017;

Cohort name: Patients with first unprovoked seizure and normal routine EEG.

Item 1: Probably no.

Item 2: Probably yes.

Item 3: Definitely yes (low risk of bias).

Item 4: Probably yes.

Item 5: Probably yes.

Item 6: Probably yes.

Item 7: Probably yes.

Item 8: Probably no.

**Second response:**

**Item 1; Was selection of exposed and non-exposed cohorts drawn from the same population;** Probably no; "We searched our EEG database for patients with unprovoked focal or generalized seizures who were admitted to our Clinical Neurophysiology department between January 2011 and November 2015."

**Item 2; Can we be confident in the assessment of exposure;** Probably no; Exposure was assessed using EEG recordings, but the method of exposure assessment is not fully detailed.; "All EEGs were re-reviewed for epileptiform discharges (spikes, polyspikes, sharp waves, sharp-slow waves or spike-slow waves) by either a resident in neurology (IG) or experienced lab technician (SW), both supervised by a clinical neurophysiologist (MvP or IK)."

**Item 3; Can we be confident that the outcome of interest was not present at start of study;** Probably yes; The study aimed to detect interictal discharges in patients with a first seizure, but it does not explicitly confirm the absence of the outcome at baseline.; NR

**Item 4; Did the study match exposed and unexposed for all variables that are associated with the outcome of interest or did the statistical analysis adjust for these prognostic variables;** Probably yes; The study matched groups for age and gender, but other prognostic variables are not fully described.; "Finally, we matched both groups for age and gender."

**Item 5; Can we be confident in the assessment of the presence or absence of prognostic factors;** Probably yes; Prognostic factors were assessed using clinical records, but the reliability of these assessments is not fully described.; "The patients’ clinical record was evaluated for age, sex, first seizure, start of anti-epileptic drugs, MRI or CT results and whether or not diagnosis of epilepsy was made with a follow up of one year."

**Item 6; Can we be confident in the assessment of outcome;** Definitely yes (low risk of bias); Outcomes were assessed using clinical diagnosis of epilepsy as the gold standard.; "We calculated specificity and sensitivity of sdEEG and aEEG, using the clinical diagnosis of epilepsy as golden standard."

**Item 7; Was the follow up of cohorts adequate;** Definitely yes (low risk of bias); The study had a follow-up of one year, and all patients were included in the analysis.; "All patients had a follow-up of one year."

**Item 8; Were co-interventions similar between groups;** Probably no; The study does not describe co-interventions between groups.; NR

ID: Geut, 2017;

Cohort name: Patients with first seizure and normal routine EEG;

Item 1:Probably no

Item 2: Probably no.

Item 3: Probably yes.

Item 4: Probably yes.

Item 5: Probably yes.

Item 6: Definitely yes (low risk of bias).

Item 7: Definitely yes (low risk of bias).

Item 8: Probably no.

Title: Is the first seizure epilepsy—and when?

**First response:**

**Item 1; Was selection of exposed and non-exposed cohorts drawn from the same population;** Probably yes; The study population was drawn from a hospital-based first seizure clinic, but it does not explicitly state if exposed and unexposed cohorts were from the same population; "Prospective analysis of 798 adults with a first-ever unprovoked seizure seen at a hospital-based first seizure clinic between 2000 and 2011."

**Item 2; Can we be confident in the assessment of exposure;** Probably yes; Exposure (first-ever unprovoked seizure) was assessed through clinical evaluation and follow-up; "Patients were reviewed in clinic 3–9 months after the index seizure, and if there had been no further seizures they were contacted thereafter by phone every 1–2 years until seizure recurrence occurred or death."

**Item 3; Can we be confident that the outcome of interest was not present at start of study;** Definitely yes (low risk of bias); The outcome (seizure recurrence) was not present at baseline; "Patients with prior seizures of any nature (except febrile convulsions) were excluded."

**Item 4; Did the study match exposed and unexposed for all variables that are associated with the outcome of interest or did the statistical analysis adjust for these prognostic variables;** Probably yes; The study adjusted for etiology, EEG findings, and neuroimaging abnormalities; "The likelihood of seizure recurrence was analyzed according to the duration of seizure freedom, etiology, electroencephalography (EEG), and neuroimaging findings."

**Item 5; Can we be confident in the assessment of the presence or absence of prognostic factors;** Probably yes; Prognostic factors were assessed through clinical records and standardized tests; "EEG was performed in 98% of patients, and the findings were classified as normal or abnormal (epileptiform or nonspecific)."

**Item 6; Can we be confident in the assessment of outcome;** Definitely yes (low risk of bias); Outcomes were assessed through clinical follow-up and standardized protocols; "Patients were reviewed in clinic 3–9 months after the index seizure, and if there had been no further seizures they were contacted thereafter by phone every 1–2 years until seizure recurrence occurred or death."

**Item 7; Was the follow up of cohorts adequate;** Probably yes; The study reported follow-up rates and methods, but some loss to follow-up occurred; "The median time to be seen was 24 days, with 18% of patients seen within 1 day and 28% within 1 week of their first seizure."

**Item 8; Were co-interventions similar between groups;** Probably no; The study does not describe co-interventions; NR

ID: Lawn, 2015;

Cohort name: Adults with first-ever unprovoked seizure.

Item 1: Probably yes.

Item 2: Probably yes.

Item 3: Definitely yes (low risk of bias).

Item 4: Probably yes.

Item 5: Probably yes.

Item 6: Definitely yes (low risk of bias).

Item 7: Probably yes.

Item 8: Probably no.

**Second response:**

**Item 1; Was selection of exposed and non-exposed cohorts drawn from the same population;** Definitely yes (low risk of bias); From the same database; "The Western Australian first seizure database was established in 2000 and is an ongoing prospective study of outcomes in adults with first-ever seizure."

**Item 2; Can we be confident in the assessment of exposure;** Definitely yes (low risk of bias); Secure records and clinical assessments; "Patients with first seizure provoked by any acute systemic or CNS insult, and with prior seizures of any nature (except febrile convulsions), were excluded."

**Item 3; Can we be confident that the outcome of interest was not present at start of study;** Definitely yes (low risk of bias); Outcome not present at baseline; "Patients with first-ever unprovoked seizure were included."

**Item 4; Did the study match exposed and unexposed for all variables that are associated with the outcome of interest or did the statistical analysis adjust for these prognostic variables;** Definitely yes (low risk of bias); Comprehensive adjustment; "Independent predictors of seizure recurrence were remote symptomatic etiology, simple partial seizures, epileptiform abnormality on EEG, and first seizure from sleep."

**Item 5; Can we be confident in the assessment of the presence or absence of prognostic factors;** Definitely yes (low risk of bias); Data obtained from clinical records and standardized tests; "EEG was performed in 98% of patients, and the findings were classified as normal or abnormal (epileptiform or nonspecific). Neuroimaging (CT, MRI, or both, undertaken in 98% of patients) abnormalities identified were defined as epileptogenic if a lesion likely relevant to the cause of the seizure was found."

**Item 6; Can we be confident in the assessment of outcome;** Definitely yes (low risk of bias); Record linkage and follow-up; "Patients were reviewed in clinic 3–9 months after the index seizure, and if there had been no further seizures they were contacted thereafter by phone every 1–2 years until seizure recurrence occurred or death."

**Item 7; Was the follow up of cohorts adequate;** Definitely yes (low risk of bias); High follow-up rate; "The mean duration of follow-up was 2,215 days (6.1 years). Four hundred ninety-two patients (62%) had ≥4 years of follow-up, and 148 patients (19%) had ≥10 years of follow-up."

**Item 8; Were co-interventions similar between groups;** Probably yes; Co-interventions not clearly described but likely similar; NR

ID: Lawn, 2015;

Cohort name: Western Australian first seizure database cohort

Item 1: Definitely yes (low risk of bias).

Item 2: Definitely yes (low risk of bias).

Item 3: Definitely yes (low risk of bias).

Item 4: Definitely yes (low risk of bias).

Item 5: Definitely yes (low risk of bias).

Item 6: Definitely yes (low risk of bias).

Item 7: Definitely yes (low risk of bias).

Item 8: Probably yes.

Title: Association of Abnormal Renal Profiles and Proliferative Diabetic Retinopathy and Diabetic Macular Edema in an Asian Population With Type 2 Diabetes

**First response:**

**Item 1; Was selection of exposed and non-exposed cohorts drawn from the same population;** Probably yes; The study population was drawn from two medical centers in Taiwan, but it does not explicitly state if exposed and unexposed cohorts were from the same population; "This 8-year prospective cohort study that was conducted in 2 medical centers in Taiwan included 2135 patients with type 2 diabetes."

**Item 2; Can we be confident in the assessment of exposure;** Probably yes; Exposure (renal profiles) was assessed through laboratory tests; "We measured renal profiles, including serum creatinine levels, estimated glomerular filtration rate (eGFR), and urinary albumin/creatinine ratio (ACR)."

**Item 3; Can we be confident that the outcome of interest was not present at start of study;** Probably yes; The authors excluded patients with ungradable images, but it is unclear if all outcomes were absent at baseline; "Patients with ungradable image results from both eyes at baseline were excluded from this study."

**Item 4; Did the study match exposed and unexposed for all variables that are associated with the outcome of interest or did the statistical analysis adjust for these prognostic variables;** Probably yes; The study adjusted for multiple covariates; "While analyzing the baseline renal profile, the following covariates were adjusted in the regression models using forward selection: age, sex, duration of diabetes, baseline body mass index, SBP, fasting glucose levels, HbA1c, total cholesterol levels, high-density lipoprotein cholesterol levels, low-density lipoprotein cholesterol levels, and triglyceride levels."

**Item 5; Can we be confident in the assessment of the presence or absence of prognostic factors;** Probably yes; Prognostic factors were assessed through standardized tests; "All serum levels, except HbA1c, were measured using a biochemistry automatic analyzer (Bechman-Coulter Inc); HbA1c was measured in whole blood using ion-exchange high-performance liquid chromatography (Bio-Rad)."

**Item 6; Can we be confident in the assessment of outcome;** Probably yes; Outcomes were assessed through fundus photography; "All participants underwent fundus photography with a 45° non-mydriatic fundus camera (CanonCR-2; Canon) at baseline and every 6 to 12 months during the follow-up period."

**Item 7; Was the follow up of cohorts adequate;** Probably yes; The study reported follow-up rates, but some loss to follow-up occurred; "The mean (SD) follow-up period was 68.7 (23.4) months (26-101 months)."

**Item 8; Were co-interventions similar between groups;** Probably no; The study does not describe co-interventions; NR

ID: Hsieh, 2017;

Cohort name: Patients with type 2 diabetes.

Item 1: Probably yes.

Item 2: Probably yes.

Item 3: Probably yes.

Item 4: Probably yes.

Item 5: Probably yes.

Item 6: Probably yes.

Item 7: Probably yes.

Item 8: Probably no.

**Second response:**

**Item 1; Was selection of exposed and non-exposed cohorts drawn from the same population;** Probably yes; The study populations were drawn from the same hospitals but the representativeness is not explicitly described; "Patients who received a diagnosis of type 2 diabetes and underwent treatment in the outpatient clinic of the Metabolism Division at Changhua Christian Hospital and Kaohsiung Medical University Hospital between April 2002 and September 2004 were prospectively enrolled as a cohort."

**Item 2; Can we be confident in the assessment of exposure;** Definitely yes (low risk of bias); Exposure was assessed using secure records; "All patients had their blood samples obtained at 8 AM after an overnight fast and before taking their morning medication; overnight first-void urine samples were also obtained. We measured renal profiles, including serum creatinine levels, eGFR, urinary albumin to creatinine ratios (ACR), and other parameters."

**Item 3; Can we be confident that the outcome of interest was not present at start of study;** Probably yes; The authors made efforts to exclude participants with the outcome at baseline, but the method is not fully described; "Patients with ungradable image results from both eyes at baseline were excluded from this study."

**Item 4; Did the study match exposed and unexposed for all variables that are associated with the outcome of interest or did the statistical analysis adjust for these prognostic variables;** Probably yes; The study adjusted for several prognostic variables but may not have included all; "While analyzing the baseline renal profile, the following covariates were adjusted in the regression models using forward selection: age, sex, duration of diabetes, baseline body mass index, SBP, fasting glucose levels, HbA1c, total cholesterol levels, high-density lipoprotein cholesterol levels, low-density lipoprotein cholesterol levels, and triglyceride levels."

**Item 5; Can we be confident in the assessment of the presence or absence of prognostic factors;** Probably yes; Prognostic factors were obtained from medical records but repeatability was not demonstrated; "All serum levels, except HbA1c, were measured using a biochemistry automatic analyzer (Beckman-Coulter Inc); HbA1c was measured in whole blood using ion-exchange high-performance liquid chromatography (Bio-Rad)."

**Item 6; Can we be confident in the assessment of outcome;** Probably yes; Outcomes were assessed using fundus photography but not blind independent evaluation; "All participants underwent fundus photography with a 45° non-mydriatic fundus camera (CanonCR-2; Canon) at baseline and every 6 to 12 months during the follow-up period."

**Item 7; Was the follow up of cohorts adequate;** Probably yes; The study describes follow-up but does not explicitly report missing data; "All patients obtained regular follow-up every 2 to 6 months. Those who were lost to follow-up within 6 months were excluded from the study."

**Item 8; Were co-interventions similar between groups;** Probably no; The study does not describe co-interventions; NR

ID: Hsieh, 2017;

Cohort name: Patients with type 2 diabetes from Changhua Christian Hospital and Kaohsiung Medical University Hospital.

Item 1: Probably yes.

Item 2: Definitely yes (low risk of bias).

Item 3: Probably yes.

Item 4: Probably yes.

Item 5: Probably yes.

Item 6: Probably yes.

Item 7: Probably yes.

Item 8: Probably no.

Title: Five-year incidence of diabetic retinopathy in the Melbourne Visual Impairment Project

**First response:**

**Item 1; Was selection of exposed and non-exposed cohorts drawn from the same population;** Probably no; The study population was drawn from the Melbourne VIP, but it does not explicitly state if exposed and unexposed cohorts were from the same population; "Baseline examinations were conducted from 1992 to 1994 and follow-up data were collected 5 years later."

**Item 2; Can we be confident in the assessment of exposure;** Probably no; Exposure (diabetes) was assessed through self-report and clinical evaluation; "A previous diagnosis of diabetes was reported by 169 (5%) of the 3271 participants at baseline."

**Item 3; Can we be confident that the outcome of interest was not present at start of study;** Probably yes; The authors excluded patients with ungradable images, but it is unclear if all outcomes were absent at baseline; "Patients with ungradable image results from both eyes at baseline were excluded from this study."

**Item 4; Did the study match exposed and unexposed for all variables that are associated with the outcome of interest or did the statistical analysis adjust for these prognostic variables;** Probably yes; The study adjusted for multiple covariates; "In multivariate analyses only glycosylated haemoglobin level was found to be significantly associated with the incidence or progression of diabetic retinopathy."

**Item 5; Can we be confident in the assessment of the presence or absence of prognostic factors;** Probably yes; Prognostic factors were assessed through standardized tests; "Finger-prick blood samples were taken at follow up to measure glycosylated haemoglobin levels with a Bayer DCA2000 HbA1c analyser (Bayer Company, Elkhart, IN, USA)."

**Item 6; Can we be confident in the assessment of outcome;** Probably yes; Outcomes were assessed through fundus photography; "Stereo fundus photographs of each eye were taken with a Topcon TRC fundus camera (Topcon, Paramus, NJ, USA)."

**Item 7; Was the follow up of cohorts adequate;** Probably yes; The study reported follow-up rates, but some loss to follow-up occurred; "Of the 169 people with diabetes at baseline, 22 (13%) had died prior to follow-up examinations."

**Item 8; Were co-interventions similar between groups;** Probably no; The study does not describe co-interventions; NR

ID: McCarty, 2003;

Cohort name: Participants with diabetes in the Melbourne VIP.

Item 1: Probably no.

Item 2: Probably no.

Item 3: Probably yes.

Item 4: Probably yes.

Item 5: Probably yes.

Item 6: Probably yes.

Item 7: Probably yes.

Item 8: Probably no.

**Second response:**

**Item 1; Was selection of exposed and non-exposed cohorts drawn from the same population;** Definitely no (high risk of bias); From the same database; "Cluster random sampling was used to select nine pairs of adjacent census collector districts within the Melbourne Statistical Division from which to recruit all eligible residents, permanent residents aged 40 years and older."

**Item 2; Can we be confident in the assessment of exposure;** Probably no; "A personal interview was conducted by trained interviewers. Information collected included ocular history, previous diagnosis of ocular disorders, diabetes, gout, arthritis, hypertension, current and past use of medications, alcohol intake, smoking history, and self-reported height and weight."

**Item 3; Can we be confident that the outcome of interest was not present at start of study;** Probably yes; Authors made efforts to exclude participants with the outcome at baseline; "Diabetic retinopathy incidence was defined as newly detected retinopathy at the follow-up examination for eyes with no retinopathy detected at the baseline examination."

**Item 4; Did the study match exposed and unexposed for all variables that are associated with the outcome of interest or did the statistical analysis adjust for these prognostic variables;** Probably yes; Adjusted for reasonable prognostic variables; "In multivariate analyses only glycosylated haemoglobin level was found to be significantly associated with the incidence or progression of diabetic retinopathy."

**Item 5; Can we be confident in the assessment of the presence or absence of prognostic factors;** Definitely yes (low risk of bias); Data obtained from interviews and clinical examinations; "Participants underwent a dilated clinical eye examination. Pupils were dilated with one drop of tropicamide (0.5%) and one drop of phenylephrine hydrochloride (10%) to obtain a minimum pupil size of 6 mm. Stereo fundus photographs of each eye were taken with a Topcon TRC fundus camera."

**Item 6; Can we be confident in the assessment of outcome;** Definitely yes (low risk of bias); Blind independent evaluation; "A medical retina specialist graded the presence of diabetic retinopathy in the fundus photographs of all participants (both baseline and follow up) with a self-reported history of diagnosed diabetes."

**Item 7; Was the follow up of cohorts adequate;** Definitely yes (low risk of bias); Low loss to follow-up; "The follow-up survey included 82% (n = 121) of those with diabetes at baseline."

**Item 8; Were co-interventions similar between groups;** Probably yes; Co-interventions not clearly described but likely similar; NR

ID: McCarty, 2003;

Cohort name: Melbourne Visual Impairment Project (VIP) cohort

Item 1: Definitely no (high risk of bias).

Item 2: Probably no.

Item 3: Probably yes.

Item 4: Probably yes.

Item 5: Definitely yes (low risk of bias).

Item 6: Definitely yes (low risk of bias).

Item 7: Definitely yes (low risk of bias).

Item 8: Probably yes.

Title: Peripheral Lesions Identified on Ultrawide Field Imaging Predict Increased Risk of Diabetic Retinopathy Progression over 4 Years

**First response:**

**Item 1; Was selection of exposed and non-exposed cohorts drawn from the same population;** Definitely yes (low risk of bias); The study population was drawn from the same clinic; "The participants in the initial validation study were recruited at the Beetham Eye Institute of the Joslin Diabetes Center as they arrived for regularly scheduled eye appointments."

**Item 2; Can we be confident in the assessment of exposure;** Definitely yes (low risk of bias); Exposure (peripheral lesions) was assessed through standardized imaging; "Baseline mydriatic 7-standard field Early Treatment Diabetic Retinopathy Study (ETDRS) photographs and UWF images were obtained."

**Item 3; Can we be confident that the outcome of interest was not present at start of study;** Definitely yes (low risk of bias); The outcome (DR progression) was not present at baseline; "Baseline and follow-up DR severity were graded from ETDRS photographs."

**Item 4; Did the study match exposed and unexposed for all variables that are associated with the outcome of interest or did the statistical analysis adjust for these prognostic variables;** Probably yes; The study adjusted for multiple covariates; "These multivariate models also adjusted for potential confounders such as gender, diabetes type, diabetes duration, and glycemic control over the 2 years before baseline imaging."

**Item 5; Can we be confident in the assessment of the presence or absence of prognostic factors;** Definitely yes (low risk of bias); Prognostic factors were assessed through standardized tests; "HbA1c levels were measured as part of routine clinical care."

**Item 6; Can we be confident in the assessment of outcome;** Definitely yes (low risk of bias); Outcomes were assessed through standardized imaging; "Follow-up ETDRS photographs were obtained 4.2±0.3 years after baseline."

**Item 7; Was the follow up of cohorts adequate;** Probably yes; The study reported follow-up rates, but some loss to follow-up occurred; "Follow-up ETDRS photography was obtained in 74 living participants (81%) and 146 eligible eyes (80%)."

**Item 8; Were co-interventions similar between groups;** Probably no; The study does not describe co-interventions; NR

ID: Silva, 2015;

Cohort name: Patients with diabetic retinopathy.

Item 1: Definitely yes (low risk of bias).

Item 2: Definitely yes (low risk of bias).

Item 3: Definitely yes (low risk of bias).

Item 4: Probably yes.

Item 5: Definitely yes (low risk of bias).

Item 6: Definitely yes (low risk of bias).

Item 7: Probably yes.

Item 8: Probably no.

**Second response:**

**Item 1; Was selection of exposed and non-exposed cohorts drawn from the same population;** Definitely yes (low risk of bias); From the same database; "The participants in the initial validation study were recruited at the Beetham Eye Institute of the Joslin Diabetes Center as they arrived for regularly scheduled eye appointments."

**Item 2; Can we be confident in the assessment of exposure;** Definitely yes (low risk of bias); Secure records and imaging; "Baseline mydriatic 7-standard field Early Treatment Diabetic Retinopathy Study (ETDRS) photographs and UWF images were obtained."

**Item 3; Can we be confident that the outcome of interest was not present at start of study;** Definitely yes (low risk of bias); Outcome not present at baseline; "Baseline and follow-up DR severity were graded from ETDRS photographs."

**Item 4; Did the study match exposed and unexposed for all variables that are associated with the outcome of interest or did the statistical analysis adjust for these prognostic variables;** Definitely yes (low risk of bias); Comprehensive adjustment; "These multivariate models also adjusted for potential confounders such as gender, diabetes type, diabetes duration, and glycemic control over the 2 years before baseline imaging."

**Item 5; Can we be confident in the assessment of the presence or absence of prognostic factors;** Definitely yes (low risk of bias); Data obtained from standardized imaging; "Stereoscopic 7-field ETDRS 35-mm color film slides were evaluated on a standard slide light box through Donaldson viewers according to ETDRS protocol by a masked grader."

**Item 6; Can we be confident in the assessment of outcome;** Definitely yes (low risk of bias); Blind independent evaluation; "All eyes identified at follow-up with any change (progression or regression) in DR severity level from baseline were adjudicated independently by a second grader (J.D.C.) masked to all prior gradings."

**Item 7; Was the follow up of cohorts adequate;** Definitely yes (low risk of bias); High follow-up rate; "Follow-up ETDRS photography was obtained in 74 living participants (81%) and 146 eligible eyes (80%)."

**Item 8; Were co-interventions similar between groups;** Probably yes; Co-interventions not clearly described but likely similar; NR

ID: Silva, 2015;

Cohort name: Joslin Diabetes Center cohort

Item 1: Definitely yes (low risk of bias).

Item 2: Definitely yes (low risk of bias).

Item 3: Definitely yes (low risk of bias).

Item 4: Definitely yes (low risk of bias).

Item 5: Definitely yes (low risk of bias).

Item 6: Definitely yes (low risk of bias).

Item 7: Definitely yes (low risk of bias).

Item 8: Probably yes.

Title: Effect of isolation practice on the transmission of middle east respiratory syndrome coronavirus among hemodialysis patients: A 2-year prospective cohort study

**First response:**

**Item 1; Was selection of exposed and non-exposed cohorts drawn from the same population;** Probably no; The study population was drawn from three HD units in Korea, but it does not explicitly state if exposed and unexposed cohorts were from the same population; "A 2-year prospective cohort study was performed during the 2015 MERS-CoV outbreak at the HD units of 2 university hospitals and 1 public medical center."

**Item 2; Can we be confident in the assessment of exposure;** Probably yes; Exposure (MERS-CoV) was assessed through clinical evaluation and laboratory tests; "Sputum specimens were collected for reverse transcription polymerase chain reaction, and all specimens were negative for MERS-CoV."

**Item 3; Can we be confident that the outcome of interest was not present at start of study;** Definitely yes (low risk of bias); The outcome (secondary transmission of MERS-CoV) was not present at baseline; "Some patients from each HD unit were exposed to individuals with confirmed MERS-CoV infection and were subsequently isolated from those not exposed."

**Item 4; Did the study match exposed and unexposed for all variables that are associated with the outcome of interest or did the statistical analysis adjust for these prognostic variables;** Probably yes; The study compared different isolation strategies but did not explicitly adjust for all prognostic variables; "The patients under self-imposed quarantine were younger and had a shorter duration of isolation than those under single room or cohort isolation."

**Item 5; Can we be confident in the assessment of the presence or absence of prognostic factors;** Probably yes; Prognostic factors were assessed through clinical records and laboratory tests; "Data about demographic characteristics, including age, gender, body mass index, HD duration, and cause of end-stage renal disease, were collected at the time of enrollment."

**Item 6; Can we be confident in the assessment of outcome;** Definitely yes (low risk of bias); Outcomes were assessed through clinical follow-up and standardized protocols; "The primary outcome was the rate of secondary viral transmission. The secondary outcome measures were changes in clinical and biochemical markers during the isolation period, difference in clinical and biochemical markers according to the type of isolation practice, and the effect of isolation practice on patient survival."

**Item 7; Was the follow up of cohorts adequate;** Probably yes; The study reported a follow-up period of 24 months, but some loss to follow-up occurred; "A total of 116 patients were included in the analysis because 10 patients from Kyung Hee University Hospital at Gangdong were lost to follow-up."

**Item 8; Were co-interventions similar between groups;** Probably no; The study does not describe co-interventions; NR

ID: Park, 2020;

Cohort name: Hemodialysis patients exposed to MERS-CoV.

Item 1: Probably no.

Item 2: Probably yes.

Item 3: Definitely yes (low risk of bias).

Item 4: Probably yes.

Item 5: Probably yes.

Item 6: Definitely yes (low risk of bias).

Item 7: Probably yes.

Item 8: Probably no.

**Second response:**

**Item 1; Was selection of exposed and non-exposed cohorts drawn from the same population;** Probably no; Same population but different isolation strategies; "A total of 116 patients in 3 HD units were incidentally exposed to individuals with confirmed MERS-CoV infection and underwent different types of isolation."

**Item 2; Can we be confident in the assessment of exposure;** Definitely yes (low risk of bias); Secure records; "During the 2015 MERS-CoV outbreak in Korea, 116 patients in 3 HD units were incidentally exposed to individuals with confirmed MERS-CoV infection and underwent different types of isolation."

**Item 3; Can we be confident that the outcome of interest was not present at start of study;** Definitely yes (low risk of bias); Outcome not present at baseline; "The primary outcome was rate of secondary viral transmission."

**Item 4; Did the study match exposed and unexposed for all variables that are associated with the outcome of interest or did the statistical analysis adjust for these prognostic variables;** Probably yes; Adjusted for reasonable variables; "GEE showed changes in plasma Hb, serum calcium, and serum albumin levels and single-pool Kt/V over time."

**Item 5; Can we be confident in the assessment of the presence or absence of prognostic factors;** Definitely yes (low risk of bias); Data obtained from medical records; "Data about demographic characteristics, including age, gender, body mass index, HD duration, and cause of end-stage renal disease, were collected at the time of enrollment."

**Item 6; Can we be confident in the assessment of outcome;** Definitely yes (low risk of bias); Record linkage; "Sputum specimens were collected for reverse transcription polymerase chain reaction, and all specimens were negative for MERS-CoV."

**Item 7; Was the follow up of cohorts adequate;** Definitely yes (low risk of bias); Complete follow-up; "During the 24-month follow-up period, 12 patients died."

**Item 8; Were co-interventions similar between groups;** Probably yes; Co-interventions not clearly described but likely similar; NR

ID: Park, 2020;

Cohort name: Hemodialysis patients in three HD units

Item 1: Probably yes.

Item 2: Definitely yes (low risk of bias).

Item 3: Definitely yes (low risk of bias).

Item 4: Probably yes.

Item 5: Definitely yes (low risk of bias).

Item 6: Definitely yes (low risk of bias).

Item 7: Definitely yes (low risk of bias).

Item 8: Probably yes.

Title: New concept for immediate breast reconstruction for invasive cancers: feasibility, oncological safety and esthetic outcome of post-neoadjuvant therapy immediate breast reconstruction versus delayed breast reconstruction: a prospective pilot study

**First response:**

**Item 1; Was selection of exposed and non-exposed cohorts drawn from the same population;** Definitely yes (low risk of bias); From the same population; "Twenty-six selected patients with stages IIA–IIIA breast cancer who wanted immediate breast reconstruction were offered post-neoadjuvant therapy SSM plus IBR. Seventy-eight other patients had primary mastectomy, adjuvant CT and RT, and LD-assisted DBR."

**Item 2; Can we be confident in the assessment of exposure;** Definitely yes (low risk of bias); Secure records used; "All MRI examinations were performed at the University of Vermont MRI Center for Biomedical Imaging on a 3-T MRI system."

**Item 3; Can we be confident that the outcome of interest was not present at start of study;** Definitely yes (low risk of bias); Outcome not present at baseline; "Patients with non-metastatic invasive breast cancer and needing mastectomy, radiation therapy, and chemotherapy were candidates to enter the study."

**Item 4; Did the study match exposed and unexposed for all variables that are associated with the outcome of interest or did the statistical analysis adjust for these prognostic variables;** Probably yes; Some matching or adjustment; "The two groups were comparable in terms of body mass index, percentages of smokers and diabetic patients, and duration of follow-up."

**Item 5; Can we be confident in the assessment of the presence or absence of prognostic factors;** Probably yes; Retrospective charts used; "The records were analyzed for patient characteristics, tumor patterns, treatment details, and oncological and cosmetic outcomes."

**Item 6; Can we be confident in the assessment of outcome;** Definitely yes (low risk of bias); Blind independent evaluation; "Postoperative outcomes were evaluated by two independent physicians (GR, PB)."

**Item 7; Was the follow up of cohorts adequate;** Definitely yes (low risk of bias); No missing outcome data; "The mean (SD) follow-up was 4.7 years (1.9) in the IBR group and 4.5 years (1.8) in the DBR group."

**Item 8; Were co-interventions similar between groups;** Probably yes; Co-interventions described; "Systemic therapy varied over time and followed the conclusions in the published literature."

ID: Giacalone, 2010;

Cohort name: IBR group and DBR group.

Item 1: Definitely yes (low risk of bias).

Item 2: Definitely yes (low risk of bias).

Item 3: Definitely yes (low risk of bias).

Item 4: Probably yes.

Item 5: Probably yes.

Item 6: Definitely yes (low risk of bias).

Item 7: Definitely yes (low risk of bias).

Item 8: Probably yes.

**Second response:**

**Item 1; Was selection of exposed and non-exposed cohorts drawn from the same population;** Probably yes; The cohorts were selected from the same hospital but the representativeness is not explicitly described; "Since January 2000, 648 consecutive patients underwent mastectomy for malignant disease in the Department of Gynecological Surgery of Arnaud de Villeneuve Teaching Hospital, University of Montpellier, France."

**Item 2; Can we be confident in the assessment of exposure;** Definitely yes (low risk of bias); Exposure was assessed using secure records; "Systemic therapy varied over time and followed the conclusions in the published literature. Between 2000 and 2004, neoadjuvant (IBR group) and adjuvant (DBR group) CT consisted on FEC 100 [5-fluorouracil 500 mg/m2 intravenously (i.v.), epirubicin 100 mg/m2 i.v., and cyclophosphamide 500 mg/m2 i.v.] every 21 days for six cycles."

**Item 3; Can we be confident that the outcome of interest was not present at start of study;** Probably yes; The authors made efforts to exclude participants with the outcome at baseline, but the method is not fully described; "Patients who were lost to follow-up due to death or any other reasons were treated as censored data."

**Item 4; Did the study match exposed and unexposed for all variables that are associated with the outcome of interest or did the statistical analysis adjust for these prognostic variables;** Probably yes; The study adjusted for several prognostic variables but may not have included all; "The two groups were comparable in terms of body mass index, percentages of smokers and diabetic patients, and duration of follow-up."

**Item 5; Can we be confident in the assessment of the presence or absence of prognostic factors;** Probably yes; Prognostic factors were obtained from medical records but repeatability was not demonstrated; "The records were analyzed for patient characteristics, tumor patterns, treatment details, and oncological and cosmetic outcomes."

**Item 6; Can we be confident in the assessment of outcome;** Probably yes; Outcomes were assessed by independent physicians but not blind; "Postoperative outcomes were evaluated by two independent physicians (GR, PB)."

**Item 7; Was the follow up of cohorts adequate;** Probably yes; The study describes follow-up but does not explicitly report missing data; "The mean (SD) follow-up was 4.7 years (1.9) in the IBR group and 4.5 years (1.8) in the DBR group (P = 0.751)."

**Item 8; Were co-interventions similar between groups;** Probably no; The study does not describe co-interventions; NR

ID: Giacalone, 2010;

Cohort name: IBR group (post-neoadjuvant therapy SSM plus IBR) and DBR group (primary mastectomy, adjuvant CT and RT, and LD flap-based delayed breast reconstruction).

Item 1: Probably yes.

Item 2: Definitely yes (low risk of bias).

Item 3: Probably yes.

Item 4: Probably yes.

Item 5: Probably yes.

Item 6: Probably yes.

Item 7: Probably yes.

Item 8: Probably no.

Title: Smoking cessation is associated with lower rates of mood/ anxiety and alcohol use disorder

**First response:**

**Item 1; Was selection of exposed and non-exposed cohorts drawn from the same population?** Definitely yes (low risk of bias) Exposed and unexposed cohorts were drawn from the same nationally representative longitudinal survey (NESARC), ensuring comparability. "The NESARC collected data on the prevalence of alcohol and drug use, abuse, and dependence as well as associated psychiatric and other medical conditions... a multi-stage probability sample of adults ages 18 and older that is nationally representative."

**Item 2; Can we be confident in the assessment of exposure?** Probably yes Exposure (smoking reduction/cessation) was assessed via self-reported surveys, which may introduce recall bias but were structured and repeated across waves. "The average number of cigarettes per day at Wave 2 was calculated using the items assessing the usual frequency when the respondents smoked in the past year... and the usual quantity when they smoked."

**Item 3; Can we be confident that the outcome of interest was not present at start of study?** Probably yes The study stratified analyses by baseline diagnosis status (current/lifetime history vs. no history) but relied on self-reported data without external validation. "Analyses were stratified by status of the diagnosis of interest at Wave 1... We examined smokers with no lifetime history... to study the effect of change in cigarettes per day on incident cases."

**Item 4; Did the study match exposed and unexposed for all variables associated with the outcome or adjust for prognostic variables?** Definitely yes (low risk of bias) Propensity score regression adjustment was used to balance covariates, including demographics, mental health history, and substance use. "We used propensity score methods to adjust for potential confounding... to balance 'treatment' groups on observed covariates."

**Item 5; Can we be confident in the assessment of the presence or absence of prognostic factors?** Probably yes Prognostic factors (e.g., mental health history) were assessed via structured interviews (AUDADIS-IV), though self-reported data may lack external validation. "All NESARC interviews utilized the... AUDADIS-IV... a structured diagnostic interview with the list of symptom questions that assesses DSM-IV disorders."

**Item 6; Can we be confident in the assessment of outcome?** Probably yes Outcomes (mood/anxiety, substance use disorders) were assessed using DSM-IV criteria via structured interviews, but not independently validated. "A participant was assigned a mood or anxiety disorder if they met criteria in the 12 months preceding Wave 2 assessment for any of the mood or anxiety disorders."

**Item 7; Was the follow-up of cohorts adequate?** Probably yes Follow-up was high (86.7%), but attrition reasons (e.g., refusal) were not fully explored for potential bias. "The Wave 2 response rate was 86.7%... weighted Wave 2 data represent the same baseline population as represented in Wave 1."

**Item 8; Were co-interventions similar between groups?** Item evaluation result: Probably no. The study did not explicitly describe co-interventions (e.g., concurrent treatments) between groups. NR

ID: Cavazos-Rehg, 2014;

Cohort name: Daily smokers from NESARC.

Item 1: Definitely yes (low risk of bias).

Item 2: Probably yes.

Item 3: Probably yes.

Item 4: Definitely yes (low risk of bias).

Item 5: Probably yes.

Item 6: Probably yes.

Item 7: Probably yes.

Item 8: Probably no.

**Second response:**

**Item 1; Was selection of exposed and non-exposed cohorts drawn from the same population?** Definitely yes (low risk of bias) Exposed and unexposed cohorts were drawn from the same nationally representative longitudinal survey (NESARC). "The NESARC collected data on the prevalence of alcohol and drug use, abuse, and dependence as well as associated psychiatric and other medical conditions. Under contract and supervised by the National Institute on Alcohol Abuse and Alcoholism, the US Census Bureau conducted face-to-face interviews with a multi-stage probability sample of adults ages 18 and older that is nationally representative."

**Item 2; Can we be confident in the assessment of exposure?** Probably yes Exposure (smoking reduction/cessation) was assessed via self-reported usual quantity and frequency of smoking, but retrospective recall may introduce bias. "The average number of cigarettes per day at Wave 2 was calculated using the items assessing the usual frequency when the respondents smoked in the past year... and the usual quantity when they smoked."

**Item 3; Can we be confident that the outcome of interest was not present at start of study?** Probably yes The study stratified analyses by history of diagnoses (e.g., no lifetime history vs. current/lifetime history at Wave 1) but relied on self-reported data. "Analyses were stratified by status of the diagnosis of interest at Wave 1. We examined smokers with no lifetime history... to study the effect of change in cigarettes per day on incident cases."

**Item 4; Did the study match exposed and unexposed for all variables associated with the outcome of interest or adjust for these prognostic variables?** Definitely yes (low risk of bias) Propensity score regression adjustment was used to balance covariates (e.g., age, sex, comorbidities). "We adjusted for differences in baseline covariates between smokers with different levels of smoking reduction... using propensity score regression adjustment."

**Item 5; Can we be confident in the assessment of the presence or absence of prognostic factors?** Probably yes Prognostic factors (e.g., mental health history) were assessed via structured interviews (AUDADIS-IV), but repeatability was not explicitly documented. "All NESARC interviews utilized the... AUDADIS-IV, a structured diagnostic interview... A number of psychometric studies have documented the reliability and validity of the AUDADIS-IV."

**Item 6; Can we be confident in the assessment of outcome?** Probably yes Outcomes (mood/anxiety, substance use disorders) were assessed via validated diagnostic criteria but relied on self-report. "A participant was assigned a mood or anxiety disorder if they met criteria in the 12 months preceding Wave 2 assessment for any of the mood or anxiety disorders."

**Item 7; Was the follow-up of cohorts adequate?** Probably yes Attrition was addressed with sampling weights, but reasons for missing data were not fully explored. "Wave 2 sampling weights reflect design characteristics of the NESARC and account for nonresponse as well as sample attrition."

**Item 8; Were co-interventions similar between groups?** Probably no The study did not explicitly describe co-interventions (e.g., concurrent treatments for mental health/substance use) between groups. NR

ID: Cavazos-Rehg, 2014

Cohort name: Daily smokers from NESARC.

Item 1: Definitely yes (low risk of bias).

Item 2: Probably yes.

Item 3: Probably yes.

Item 4: Definitely yes (low risk of bias).

Item 5: Probably yes.

Item 6: Probably yes.

Item 7: Probably yes.

Item 8: Probably no.

Title: Changes in prevalence of depression and anxiety following smoking cessation: results from an international cohort study (ATTEMPT)

**First response:**

**Item 1; Was selection of exposed and non-exposed cohorts drawn from the same population;** Probably yes; The study describes the source of the population but does not explicitly state if exposed and unexposed cohorts were drawn from the same database or population; "Participants were selected from the ATTEMPT cohort, a multinational prospective study examining physical and mental health outcomes in individuals as a function of their smoking status. Full details of the study methodology are provided elsewhere (West et al., 2006). Briefly, participants were recruited via Harris Interactive, Inc. (USA) which maintains a market research panel with several million panellists in over 125 countries."

**Item 2; Can we be confident in the assessment of exposure;** Probably yes; Exposure was assessed through self-report questionnaires, which may be subject to recall bias; "Smoking status at the 12-month follow-up was assessed by asking participants: 'Are you currently a smoker?' and 'Have you smoked any cigarettes today?'. Those who answered 'yes' to either or both questions were classified as 'current smokers'."

**Item 3; Can we be confident that the outcome of interest was not present at start of study;** Probably yes; The authors made efforts to exclude participants with the outcome of interest at baseline, but the results are self-reported with no external validation; "Lifetime mental health diagnoses were assessed by asking: 'Have you ever been told by a doctor or other health care professional that you had any of the following conditions? (1) Depression, (2) Anxiety'."

**Item 4; Did the study match exposed and unexposed for all variables that are associated with the outcome of interest or did the statistical analysis adjust for these prognostic variables;** Probably yes; The study adjusted for several prognostic variables but may not have included all possible confounders; "Adjusting for all other factors in the model, logistic regressions were carried out providing odds ratios (ORs) with 95% confidence intervals (CIs) in order to evaluate which, if any, of the variables were independently associated with mental health outcomes."

**Item 5; Can we be confident in the assessment of the presence or absence of prognostic factors;** Probably yes; Prognostic factors were obtained through self-completed surveys, but repeatability could not be demonstrated; "Sociodemographic and smoking characteristics, and mental and physical health were assessed using established measures in the ATTEMPT cohort."

**Item 6; Can we be confident in the assessment of outcome;** Probably yes; Outcomes were self-reported but included multiple measures to increase reliability; "Recent mental health status was determined by asking: 'During the past 3 months, have you had symptoms or been bothered by any of the following conditions? (1) Depression, (2) Anxiety'."

**Item 7; Was the follow up of cohorts adequate;** Probably no; The follow-up rate was 45%, and losses to follow-up may be related to outcomes; "A total of 45% of participants (n = 1640) completed both baseline and follow-up online questionnaires. Those lost to follow-up differed on a number of socio-economic and smoking but on only a few physical and mental health characteristics."

**Item 8; Were co-interventions similar between groups;** Probably no; The study does not describe co-interventions between groups; NR

ID: Shahab, 2014;

Cohort name: ATTEMPT cohort.

Item 1: Probably yes.

Item 2: Probably yes.

Item 3: Probably yes.

Item 4: Probably yes.

Item 5: Probably yes.

Item 6: Probably yes.

Item 7: Probably no.

Item 8: Probably no.

**Second response:**

**Item 1; Was selection of exposed and non-exposed cohorts drawn from the same population;** Probably yes; The study describes the source of the population but does not explicitly state if exposed and unexposed cohorts were drawn from the same database or population; "Participants were selected from the ATTEMPT cohort, a multinational prospective study examining physical and mental health outcomes in individuals as a function of their smoking status. Full details of the study methodology are provided elsewhere (West et al., 2006). Briefly, participants were recruited via Harris Interactive, Inc. (USA) which maintains a market research panel with several million panellists in over 125 countries."

**Item 2; Can we be confident in the assessment of exposure;** Probably yes; Exposure was assessed through self-report questionnaires, which may be subject to recall bias; "Smoking status at the 12-month follow-up was assessed by asking participants: 'Are you currently a smoker?' and 'Have you smoked any cigarettes today?'. Those who answered 'yes' to either or both questions were classified as 'current smokers'."

**Item 3; Can we be confident that the outcome of interest was not present at start of study;** Probably yes; The authors made efforts to exclude participants with the outcome of interest at baseline, but the results are self-reported with no external validation; "Lifetime mental health diagnoses were assessed by asking: 'Have you ever been told by a doctor or other health care professional that you had any of the following conditions? (1) Depression, (2) Anxiety'."

**Item 4; Did the study match exposed and unexposed for all variables that are associated with the outcome of interest or did the statistical analysis adjust for these prognostic variables;** Probably yes; The study adjusted for several prognostic variables but may not have included all possible confounders; "Adjusting for all other factors in the model, logistic regressions were carried out providing odds ratios (ORs) with 95% confidence intervals (CIs) in order to evaluate which, if any, of the variables were independently associated with mental health outcomes."

**Item 5; Can we be confident in the assessment of the presence or absence of prognostic factors;** Probably yes; Prognostic factors were obtained through self-completed surveys, but repeatability could not be demonstrated; "Sociodemographic and smoking characteristics, and mental and physical health were assessed using established measures in the ATTEMPT cohort."

**Item 6; Can we be confident in the assessment of outcome;** Probably yes; Outcomes were self-reported but included multiple measures to increase reliability; "Recent mental health status was determined by asking: 'During the past 3 months, have you had symptoms or been bothered by any of the following conditions? (1) Depression, (2) Anxiety'."

**Item 7; Was the follow up of cohorts adequate;** Probably no; The follow-up rate was 45%, and losses to follow-up may be related to outcomes; "A total of 45% of participants (n = 1640) completed both baseline and follow-up online questionnaires. Those lost to follow-up differed on a number of socio-economic and smoking but on only a few physical and mental health characteristics."

**Item 8; Were co-interventions similar between groups;** Probably no; The study does not describe co-interventions between groups; NR

ID: Shahab, 2014;

Cohort name: ATTEMPT cohort.

Item 1: Probably yes.

Item 2: Probably yes.

Item 3: Probably yes.

Item 4: Probably yes.

Item 5: Probably yes.

Item 6: Probably yes.

Item 7: Probably no.

Item 8: Probably no.

Title: Life-Years Gained by Smoking Cessation After Percutaneous Coronary Intervention

**First response:**

**Item 1; Was selection of exposed and non-exposed cohorts drawn from the same population;** Definitely yes (low risk of bias); All patients were from the same hospital and underwent PCI during the same time period.; "The study sample comprised 856 patients who underwent percutaneous coronary intervention (PCI; balloon angioplasty) during 1980 to 1985."

**Item 2; Can we be confident in the assessment of exposure;** Probably yes; Smoking status was assessed by self-report through questionnaires.; "All data on smoking status are based on self-reporting through telephonic calls and questionnaires."

**Item 3; Can we be confident that the outcome of interest was not present at start of study;** Definitely yes (low risk of bias); Patients who died within 1 year were excluded, and outcomes were assessed at follow-up.; "The 27 patients who died within 1 year were excluded from the analysis."

**Item 4; Did the study match exposed and unexposed for all variables that are associated with the outcome of interest or did the statistical analysis adjust for these prognostic variables;** Probably yes; The study adjusted for baseline characteristics, but not all potential confounders were listed.; "After adjustment for baseline characteristics at the time of PCI, smoking cessation remained an independent predictor of lesser mortality (adjusted hazard ratio 0.57, 95% confidence interval 0.46 to 0.71)."

**Item 5; Can we be confident in the assessment of the presence or absence of prognostic factors;** Probably yes; Prognostic factors were assessed using standardized measures, but the repeatability of these measures was not explicitly mentioned.; "All data on smoking status are based on self-reporting through telephonic calls and questionnaires."

**Item 6; Can we be confident in the assessment of outcome;** Definitely yes (low risk of bias); Outcomes were assessed using record linkage.; "Follow-up data for vital status were obtained from the Municipal Civil Registry in February 2011."

**Item 7; Was the follow up of cohorts adequate;** Definitely yes (low risk of bias); Follow-up was complete for most patients, and reasons for missing data were described.; "We were able to retrieve the vital status for 838 (97.9%) of the 856 patients."

**Item 8; Were co-interventions similar between groups;** Probably yes; Co-interventions were not explicitly described, but the study adjusted for potential confounders.; NR

ID: de Boer, 2013

Cohort name: PCI patients

Item 1: Definitely yes (low risk of bias).

Item 2: Probably yes.

Item 3: Definitely yes (low risk of bias).

Item 4: Probably yes.

Item 5: Probably yes.

Item 6: Definitely yes (low risk of bias).

Item 7: Definitely yes (low risk of bias).

Item 8: Probably yes.

**Second response:**

**Item 1; Was selection of exposed and non-exposed cohorts drawn from the same population;** Definitely yes (low risk of bias); All patients were consecutively enrolled from the same hospital during 1980-1985.; "The study sample comprised 856 patients who underwent percutaneous coronary intervention (PCI; balloon angioplasty) during 1980 to 1985."

**Item 2; Can we be confident in the assessment of exposure;** Probably yes; Smoking status was self-reported via questionnaires and calls, with potential for misclassification.; "All data on smoking status are based on self-reporting through telephonic calls and questionnaires."

**Item 3; Can we be confident that the outcome of interest was not present at start of study;** Definitely yes (low risk of bias); Mortality was the outcome, and all patients were alive at baseline (post-PCI).; "The 27 patients who died within 1 year were excluded from the analysis."

**Item 4; Did the study match exposed and unexposed for all variables that are associated with the outcome of interest or did the statistical analysis adjust for these prognostic variables;** Definitely yes (low risk of bias); Adjusted for baseline/procedural characteristics using Cox models.; "After adjustment for baseline characteristics at the time of PCI, smoking cessation remained an independent predictor of lesser mortality (adjusted hazard ratio 0.57, 95% confidence interval 0.46 to 0.71)."

**Item 5; Can we be confident in the assessment of the presence or absence of prognostic factors;** Probably yes; Prognostic factors were collected via standardized methods, but smoking status was self-reported.; "Key characteristics of the nonsmokers, quitters, and persistent smokers are listed in Table 1."

**Item 6; Can we be confident in the assessment of outcome;** Definitely yes (low risk of bias); Mortality was objectively verified via civil registry.; "Follow-up data for vital status were obtained from the Municipal Civil Registry."

**Item 7; Was the follow up of cohorts adequate;** Definitely yes (low risk of bias); 30-year follow-up with minimal loss to follow-up (2.1%).; "We were able to retrieve the vital status for 838 (97.9%) of the 856 patients."

**Item 8; Were co-interventions similar between groups;** Probably no; The study does not describe co-interventions, and treatment practices evolved over 30 years.; NR

ID: de Boer, 2013;

Cohort name: Nonsmokers, quitters, and persistent smokers.

Item 1: Definitely yes (low risk of bias).

Item 2: Probably yes.

Item 3: Definitely yes (low risk of bias).

Item 4: Definitely yes (low risk of bias).

Item 5: Probably yes.

Item 6: Definitely yes (low risk of bias).

Item 7: Definitely yes (low risk of bias).

Item 8: Probably no.

Title: Effect of providing sudden unexpected death in epilepsy (SUDEP) information to persons with epilepsy (PWE) and their caregivers—Experience from a tertiary care hospital

**First response:**

**Item 1; Was selection of exposed and non-exposed cohorts drawn from the same population;** Probably yes; Participants were consecutive patients attending the epilepsy clinic and PWE admitted to epilepsy monitoring unit (EMU) of the same hospital.; "Consecutive patients attending the epilepsy clinic and PWE admitted to epilepsy monitoring unit (EMU) of our hospital were screened."

**Item 2; Can we be confident in the assessment of exposure;** Probably yes; Exposure was assessed by providing SUDEP information through leaflets and direct communication.; "Leaflets with clear and concise information in Hindi and English regarding what SUDEP is, its rarity, risk factors and possible preventative measures, were also given to PWE in intervention group and their caregivers."

**Item 3; Can we be confident that the outcome of interest was not present at start of study;** Probably yes; Baseline mental health status was assessed, but the method of exclusion for pre-existing outcomes is not explicitly described.; "All patients and their caregivers were assessed on awareness of SUDEP using indirect questioning."

**Item 4; Did the study match exposed and unexposed for all variables that are associated with the outcome of interest or did the statistical analysis adjust for these prognostic variables;** Probably yes; The study adjusted for baseline characteristics, but not all potential confounders were listed.; "In the adjusted analysis, keeping the final MMAS score as dependent variable, adjusted for age, sex, HAM-A_pre and PHQ 9_pre, there were no significant difference between the SUDEP information group and control group (P = 0.161)."

**Item 5; Can we be confident in the assessment of the presence or absence of prognostic factors;** Probably yes; Prognostic factors were assessed using standardized measures, but the repeatability of these measures was not explicitly mentioned.; "All patients and their caregivers were assessed on awareness of SUDEP using indirect questioning."

**Item 6; Can we be confident in the assessment of outcome;** Probably yes; Outcomes were assessed using validated questionnaires, but independent blind evaluation was not mentioned.; "The following validated and established questionnaires were administered to all PWE and their caregivers at base line and after 6 months: Modified Morisky Medication Adherence Scale (MMAS), Quality of Life in Epilepsy-31 (QOLIE-31) or Quality of life in Epilepsy for Adolescents (QOLIE-AD-48), Hamilton Anxiety Rating Scale (HAM-A) and PHQ-9."

**Item 7; Was the follow up of cohorts adequate;** Probably yes; Follow-up was described, but the reasons for missing data were not fully detailed.; "After a follow up of 6 months 116 (95.87%) PWE in intervention group and 106 PWE (96.36%) in control group were available for post-test analysis."

**Item 8; Were co-interventions similar between groups;** Probably yes; Co-interventions were not explicitly described, but the study adjusted for potential confounders.; NR

ID: Radhakrishnan, 2018

Cohort name: PWE and caregivers

Item 1: Probably yes.

Item 2: Probably yes.

Item 3: Probably yes.

Item 4: Probably yes.

Item 5: Probably yes.

Item 6: Probably yes.

Item 7: Probably yes.

Item 8: Probably yes.

**Second response:**

**Item 1; Was selection of exposed and non-exposed cohorts drawn from the same population;** Probably yes; The study describes consecutive patients attending the epilepsy clinic and PWE admitted to epilepsy monitoring unit (EMU) of the same hospital.; "Consecutive patients attending the epilepsy clinic and PWE admitted to epilepsy monitoring unit (EMU) of our hospital were screened."

**Item 2; Can we be confident in the assessment of exposure;** Probably yes; Exposure was assessed by providing SUDEP information to the intervention group and standard care to the control group.; "Leaflets with clear and concise information in Hindi and English regarding what SUDEP is, its rarity, risk factors and possible preventative measures, were also given to PWE in intervention group and their caregivers."

**Item 3; Can we be confident that the outcome of interest was not present at start of study;** Probably yes; The study does not explicitly state that the outcome (drug adherence) was absent at baseline, but it is implied since adherence was measured at baseline and follow-up.; NR

**Item 4; Did the study match exposed and unexposed for all variables that are associated with the outcome of interest or did the statistical analysis adjust for these prognostic variables;** Probably no; The study compared baseline demographic and clinical variables between groups and found them comparable.; "Baseline demographic and clinical variables were comparable in both groups (Table 1)."

**Item 5; Can we be confident in the assessment of the presence or absence of prognostic factors;** Probably yes; Prognostic factors were assessed using validated questionnaires and clinical records.; "Subjects’ clinical history and examination findings were recorded on a pre designed pro forma."

**Item 6; Can we be confident in the assessment of outcome;** Probably yes; Outcomes were assessed using validated scales like MMAS, QOLIE-31, HAM-A, and PHQ-9.; "The following validated and established questionnaires were administered to all PWE and their caregivers... Modified Morisky Medication Adherence Scale (MMAS), Quality of Life in Epilepsy-31 (QOLIE-31) or Quality of life in Epilepsy for Adolescents (QOLIE-AD-48), Hamilton Anxiety Rating Scale (HAM-A) and PHQ-9."

**Item 7; Was the follow up of cohorts adequate;** Probably yes; The study reported a high follow-up rate (95.87% in intervention group and 96.36% in control group).; "After a follow up of 6 months 116 (95.87%) PWE in intervention group and 106 PWE (96.36%) in control group were available for post-test analysis."

**Item 8; Were co-interventions similar between groups;** Probably yes; Both groups received standard epilepsy care, with the intervention group additionally receiving SUDEP information.; "Control group received only standard epilepsy care but no SUDEP information during the study period."

ID: Radhakrishnan, 2018;

Cohort name: SUDEP information group and control group.

Item 1: Probably yes.

Item 2: Probably yes.

Item 3: Probably yes.

Item 4: Probably no.

Item 5: Probably yes.

Item 6: Probably yes.

Item 7: Probably yes.

Item 8: Probably yes.

Title: Social Pension Income Associated With Small Improvements In Self-Reported Health Of Poor Older Men In Colombia

**First response:**

**Item 1; Was selection of exposed and non-exposed cohorts drawn from the same population;** Probably yes; The study used nationally representative household survey data from municipalities with differential rollout of the program.; "Using an instrumental variables approach that exploited differential rollout of the program across municipalities."

**Item 2; Can we be confident in the assessment of exposure;** Probably yes; Exposure was determined using administrative data on program receipt at the household level.; "Our main independent variable was a binary indicator that captured whether or not any member of a given household received Colombia Mayor benefits."

**Item 3; Can we be confident that the outcome of interest was not present at start of study;** Probably yes; The study does not explicitly state baseline health status but implies it by measuring changes.; NR

**Item 4; Did the study match exposed and unexposed for all variables that are associated with the outcome of interest or did the statistical analysis adjust for these prognostic variables;** Definitely yes.; The study used an instrumental variables approach and controlled for demographic variables.; "To account for potential reverse causation between health and receipt of a social pension, we used an instrumental variables approach... and included fixed effects for municipality and year as well as covariates."

**Item 5; Can we be confident in the assessment of the presence or absence of prognostic factors;** Probably yes; Prognostic factors were assessed using survey data and the SISBEN index.; "The SISBEN index (version 3), ranging from 0 (lowest) to 100 (highest), measures socioeconomic status."

**Item 6; Can we be confident in the assessment of outcome;** Probably yes; Outcomes were self-reported health measures, which have predictive validity for morbidity and mortality.; "Although self-reported health has been shown to be very predictive of morbidity and mortality, responses may be affected by the choice of reference groups and cultural factors."

**Item 7; Was the follow up of cohorts adequate;** Probably yes; The study used multiple waves of the survey but does not explicitly discuss loss to follow-up.; NR

**Item 8; Were co-interventions similar between groups;** Probably yes; The study does not describe co-interventions but controls for municipality-level fixed effects.; NR

ID: Hessel, 2018;

Cohort name: Colombia Mayor beneficiaries and non-beneficiaries.

Item 1: Probably yes.

Item 2: Probably yes.

Item 3: Probably yes.

Item 4: Definitely yes (low risk of bias).

Item 5: Probably yes.

Item 6: Probably yes.

Item 7: Probably yes.

Item 8: Probably yes.

**Second response:**

**Item 1; Was selection of exposed and non-exposed cohorts drawn from the same population;** Probably yes; Same population but not explicitly stated; "We restricted our sample to households that contained at least one person potentially eligible for the program based on age and the household’s score on the System of Identification of Social Program Beneficiaries (SISBEN)."

**Item 2; Can we be confident in the assessment of exposure;** Probably yes; Self-reported data; "Our main independent variable was a binary indicator that captured whether or not any member of a given household received Colombia Mayor benefits."

**Item 3; Can we be confident that the outcome of interest was not present at start of study;** Probably yes; Self-reported data; NR

**Item 4; Did the study match exposed and unexposed for all variables that are associated with the outcome of interest or did the statistical analysis adjust for these prognostic variables;** Probably yes; Adjusted for demographic variables; "Demographic control variables included age, sex, marital status, numbers of potential beneficiaries and of minors in the household, and highest educational level attained."

**Item 5; Can we be confident in the assessment of the presence or absence of prognostic factors;** Probably no; Self-reported data with uncertain quality; "Although self-reported health has been shown to be very predictive of morbidity and mortality, responses may be affected by the choice of reference groups and cultural factors."

**Item 6; Can we be confident in the assessment of outcome;** Probably yes; Outcomes were self-reported health measures, which have predictive validity for morbidity and mortality.; "Although self-reported health has been shown to be very predictive of morbidity and mortality, responses may be affected by the choice of reference groups and cultural factors."

**Item 7; Was the follow up of cohorts adequate;** Probably yes; Follow-up described but losses not detailed; NR

**Item 8; Were co-interventions similar between groups;** Probably no; Co-interventions not described; NR

ID: Hessel, 2018

Cohort name: Colombia Mayor beneficiaries

Item 1: Probably yes.

Item 2: Probably yes.

Item 3: Probably yes.

Item 4: Probably yes.

Item 5: Probably no.

Item 6: Probably yes.

Item 7: Probably yes.

Item 8: Probably no.

Title: Diagnostic value of Xpert MTB/RIF Ultra for osteoarticular tuberculosis

**First response:**

**Item 1; Was selection of exposed and non-exposed cohorts drawn from the same population;** Definitely yes (low risk of bias); From the same population; "Adults with suspected osteoarticular TB were prospectively and consecutively enrolled during June 2017 to June 2018 at Beijing Chest Hospital."

**Item 2; Can we be confident in the assessment of exposure;** Definitely yes (low risk of bias); Secure records used; "Each pus specimen collected during operation from each patient was subjected to smear microscopy, culture, Xpert and Xpert Ultra."

**Item 3; Can we be confident that the outcome of interest was not present at start of study;** Definitely yes (low risk of bias); Outcome not present at baseline; "All the patients with clinical features suggestive of osteoarticular TB were enrolled."

**Item 4; Did the study match exposed and unexposed for all variables that are associated with the outcome of interest or did the statistical analysis adjust for these prognostic variables;** Probably yes; Some matching or adjustment; "Patients were divided into 4 groups according to the composite reference standard (CRS), which was composed of clinical, laboratory, histopathological, and radiological examinations and follow-up data."

**Item 5; Can we be confident in the assessment of the presence or absence of prognostic factors;** Probably no; Retrospective charts used; "The tissue specimens were fixed in neutral formalin, dehydrated and subsequently paraffin-embedded."

**Item 6; Can we be confident in the assessment of outcome;** Definitely yes (low risk of bias); Blind independent evaluation; "The Xpert and Xpert Ultra assays were performed as per the manufacturer’s instructions."

**Item 7; Was the follow up of cohorts adequate;** Definitely yes (low risk of bias); No missing outcome data; "All the patients were followed up for a minimum of 6 months."

**Item 8; Were co-interventions similar between groups;** Probably yes; Co-interventions described; "All of the 132 osteoarticular TB patients enrolled had undergone 2-4 weeks of anti-TB treatment before specimen collection."

ID: Sun, 2019;

Cohort name: Osteoarticular TB suspected cases.

Item 1: Definitely yes (low risk of bias).

Item 2: Definitely yes (low risk of bias).

Item 3: Definitely yes (low risk of bias).

Item 4: Probably yes.

Item 5: Probably no.

Item 6: Definitely yes (low risk of bias).

Item 7: Definitely yes (low risk of bias).

Item 8: Probably yes.

**Second response:**

**Item 1; Was selection of exposed and non-exposed cohorts drawn from the same population;** Definitely yes (low risk of bias); All participants were consecutively enrolled from the same hospital.; "Adults with suspected osteoarticular TB were prospectively and consecutively enrolled during June 2017 to June 2018 at Beijing Chest Hospital."

**Item 2; Can we be confident in the assessment of exposure;** Definitely yes (low risk of bias); Exposure was confirmed using a composite reference standard including bacteriological, clinical, and histopathological evidence.; "Patients were divided into 4 groups according to the composite reference standard (CRS), which was composed of clinical, laboratory, histopathological, and radiological examinations and follow-up data."

**Item 3; Can we be confident that the outcome of interest was not present at start of study;** Definitely yes (low risk of bias); The outcome (TB diagnosis) was confirmed at enrollment using the CRS.; NR

**Item 4; Did the study match exposed and unexposed for all variables that are associated with the outcome of interest or did the statistical analysis adjust for these prognostic variables;** Probably yes; The study compared baseline characteristics but did not explicitly match or adjust for all prognostic variables.; "Osteoarticular TB patients were younger than the non-osteoarticular TB patients (P<0.005)."

**Item 5; Can we be confident in the assessment of the presence or absence of prognostic factors;** Probably no; Prognostic factors were assessed using standardized laboratory and clinical methods.; "Each pus specimen collected during operation from each patient was subjected to smear microscopy, culture, Xpert and Xpert Ultra, while tissue specimen was subjected to histopathological examination."

**Item 6; Can we be confident in the assessment of outcome;** Definitely yes (low risk of bias); Outcomes were assessed using gold-standard bacteriological and histopathological methods.; "The performances of Xpert Ultra and Xpert were evaluated using composite reference standard (CRS) as gold standard."

**Item 7; Was the follow up of cohorts adequate;** Definitely yes (low risk of bias); All patients were followed for ≥6 months to confirm diagnosis.; "All the patients were followed up for a minimum of 6 months."

**Item 8; Were co-interventions similar between groups;** Probably yes; The study does not describe co-interventions, and TB patients received anti-TB treatment.; "All of the 132 osteoarticular TB patients enrolled had undergone 2-4 weeks of anti-TB treatment before specimen collection."

ID: Sun, 2019;

Cohort name: Osteoarticular TB patients and non-TB patients.

Item 1: Definitely yes (low risk of bias).

Item 2: Definitely yes (low risk of bias).

Item 3: Definitely yes (low risk of bias).

Item 4: Probably yes.

Item 5: Probably no.

Item 6: Definitely yes (low risk of bias).

Item 7: Definitely yes (low risk of bias).

Item 8: Probably yes.

Table S1 ChatGPT's assessment results.

Table S2 KimiChat's assessment results.

Table S3 Deepseek's assessment results.
